# Global, regional, and national burden of cirrhosis and other chronic liver diseases due to alcohol use, 1990–2019: a systematic analysis for the Global Burden of Disease study 2019

**DOI:** 10.1186/s12876-022-02518-0

**Published:** 2022-11-23

**Authors:** Xiansheng Zhang, Xin Zhang, Meilian Liu, Lin Zhu, Zhuokai He

**Affiliations:** 1 Department of medical administration, Affiliated Hospital of Guilin Medical University, 15 Lequn Road, 541001 Guilin, China; 2 Department of Radiation Oncology, Affiliated Hospital of Guilin Medical University, 15 Lequn Road, 541001 Guilin, China

**Keywords:** Cirrhosis, Chronic liver diseases, Alcohol use, Estimated annual percentage change, Disability-adjusted life years

## Abstract

**Background:**

To date, no study has evaluated trends in the burden of alcohol-induced cirrhosis and other chronic liver diseases based on the Global Burden of Diseases, Injuries, and Risk Factors (GBD) 2019 study. Herein, we report on the global burden of alcohol-induced cirrhosis and other chronic liver diseases in terms of age, sex, and sociodemographic index (SDI) from 1990 to 2019, based on analysis of GBD 2019 data.

**Methods:**

The estimated annual percentage change (EAPC) was calculated to determine the trends in the age-standardized incidence and mortality rates and disability-adjusted life years (DALYs) for alcohol-induced cirrhosis and other chronic liver diseases.

**Results:**

From 1990 to 2019, the global age-standardized incidence rate showed an upward trend (EAPC = 0.10), whereas the global age-standardized mortality rate and DALYs showed a downward trend (EAPC = − 0.88 and − 0.89, respectively). Low-(187.08 in 2019) and low-middle (178.11 in 2019)SDI regions had much higher age-standardized DALYs. Eastern Europe saw the largest increases in the age-standardized mortality rate and DALYs. Lithuania had the largest increase in mortalities caused by alcohol-induced cirrhosis and other chronic liver diseases(EAPC = 4.61). The age-standardized mortality rates and DALYs were higher in men than in women.

**Conclusion:**

From 1990 to 2019, the age-standardized incidence rate of alcohol-induced cirrhosis and other chronic liver diseases increased globally; however, both the age-standardized mortality rate and DALYs caused by alcohol-induced cirrhosis and other chronic liver diseases showed decreasing trends. Future studies should devise preventive strategies for low and low-middle SDI regions, Eastern Europe, Lithuania, and other high-risk regions.

**Supplementary Information:**

The online version contains supplementary material available at 10.1186/s12876-022-02518-0.

## Background

Alcohol-induced liver disease is the primary cause of for liver-related morbidity and mortality worldwide. The global prevalence of alcohol-induced compensated and decompensated cirrhosis in 2017 was 23.6 and 2.5 million, respectively [1]. Alcohol-induced liver cirrhosis account for 1% of deaths worldwide, and this trend is projected to increase in the coming years [1]. Moreover, because treatments for viral hepatitis are being devised and continuously improved, alcohol-related liver disease is now becoming the major contributor to the global burden of liver disease [1].

The epidemiological characteristics of alcohol-induced cirrhosis and other chronic liver diseases are unclear. To the best of our knowledge, no studies have evaluated the trends in the burden of alcohol-induced cirrhosis and other chronic liver diseases worldwide. However, the Global Burden of Diseases, Injuries, and Risk Factors (GBD) 2019 study provided accurate estimates of alcohol-induced cirrhosis and other chronic liver diseases through the latest population-based data and analytical methods [2].

Therefore, the current study aimed to generate detailed and accurate estimates of epidemiological trends in the incidence of and mortality and disability-adjusted life years (DALYs) caused by alcohol-related cirrhosis and other chronic liver diseases, stratified by age-, sex-, and sociodemographic index (SDI), from the GBD 2019 study data, which were gathered from 204 countries and territories. By analyzing the characteristics of the global distribution, gender and age, and time trends of incidence, mortality and DALYs, it can not only help us understand alcohol-induced cirrhosis and other chronic liver diseases in different countries and regions around the world, and also to help us to identify high-risk groups, so as to more effectively improve the prevention of alcohol-induced cirrhosis and other chronic liver diseases, and can also help the relevant functional departments in various countries to optimize the allocation of medical resources.

## Methods

### Overview

The GBD 2019 study, estimated the mortality, incidence, and DALYs caused by 369 diseases and injuries among both men and women from 204 countries and territories [2]. The Guidelines for Accurate and Transparent Health Estimates Reporting Checklist was followed in this study [3]. This study was performed in line with the principles of the Declaration of Helsinki. This study was approved by the Institutional Review Board of Affiliated Hospital of Guilin Medical University and Informed consent of this study was waived because no identifiable information was included in the analyses. The data included in the GBD study were obtained primarily from censuses, household surveys, disease registries, health service utilization data, and vital statistics records.

The trends in the incidence of alcohol-induced cirrhosis and other chronic liver diseases were determined by analyzing the data of the GBD study. The data were stratified by age, as follows: 15–19, 20–24, 25–29, 30–34, 35–39, 40–44, 45–49, 50–54, 55–59, 60–64, 65–69, 70–74, 75–79, 80–84, 85–89, 90–94, and ≥ 95 years old.

### Data sources

The data for incidence, mortality and DALYs for alcohol-induced cirrhosis and other chronic liver diseases from 1990 to 2019 were collected from the Global Health Data Exchange (GHDx) query tool (http://ghdx.healthdata.org/gbd-results-tool). The data included in the GBD 2019 study were obtained via various pathways, primarily from censuses, household surveys, disease registries, health service utilization data, and vital statistics records [2]. Alcohol-induced cirrhosis and other chronic liver diseases were identified according to the International Classification of Diseases 10th Revision codes (K70–K70.3).

### Estimation of incidence, mortality, and DALYs

Incidence is the number of new cases of a particular disease during a designated period in a designated population. Age-standardized mortality rates are the age-specific number of deaths per 100,000 persons, whereas age-standardized DALYs are the number of years lived with a disability and the number of life years lost per 100,000 persons after age standardization. In this study, the aggregate of years of life lost and years lived with a disability was regarded as the number of DALYs; thus, DALYs included both fatal and nonfatal disease burdens.

### Statistical analysis

The age-standardized rates of alcohol-induced cirrhosis and other chronic liver diseases (per 100,000 persons) were calculated as follows:

$$Rat{e_{age - standardized}}\, = \,\frac{{\sum _{i\, = \,1}^R{r_{i}p_{i}}}}{{\sum _{i\, = \,1}^Rp_{i}}}$$

where ${\text{r} }_{\text{i}}$ and ${\text{p}}_{\text{i}}$ indicate the age-specific rate and the number of persons in the same age subgroup of the selected reference criterion population, and i represents the i^th^ age group.

The estimated annual percentage change (EAPC) is an indicator of the age-standardized rate trends [4] and was calculated using the following formulas:

$$y = \text{a} + \text{b}x + \in$$

$$EAPC{\rm{ }} = {\rm{ }}100{\rm{ }} \times {\rm{ }}\left[ {exp{\rm{ }}\left(\beta \right){\rm{ }}-{\rm{ }}1} \right],$$

where *y* represents ln (age-standardized rate), *x* is the calendar year, and β is the evaluated value of the slope (b). The formula EAPC = 100 × [exp(β)–1], was then used to estimate the 95% confidence interval (CI), with the standard error determined from the fitted regression line. If EAPC and the lower limit of the 95% CI were both greater than zero, the age-standardized rate was considered to show an increasing trend; by contrast, when these parameters were both less than zero, the age-standardized rate was considered to show a decreasing trend. Otherwise, the age-standardized rate was considered stable during this period.

## Results

### Change in the incidence of alcohol-induced cirrhosis and other chronic liver diseases

Globally, the number of incidences of alcohol-induced cirrhosis and other chronic liver diseases increased from 238767.07 (95% CI, 171651.75–317513.12) in 1990 to 436056.22 (95% CI, 314528.67–579116.38) in 2019. Based on these values, the number of incidences due to these diseases increased by 82.63% from 1990 to 2019.

In 2019, the number of incident cases of alcohol-induced cirrhosis and other chronic liver diseases was highest in the middle SDI region (137128.88,95% CI, 95894.88–8185308.32) and lowest in the low SDI region(27965.31,95% CI, 17184.31–40461.89) (Table 1). Between 1990 and 2019, the incident number of alcohol-induced cirrhosis and other chronic liver diseases in all SDI regions increased. Notably, the low SDI region [EAPC,3.95(95% UI 3.77–4.13)] had a tremendous increase in the number of alcohol-induced cirrhosis and other chronic liver diseases changes from 1990 to 2019 (Table 1).

**Table 1:** Age-standardized incidence rate (ASIR) of alcohol-induced cirrhosis and other chronic liver diseases and its temporal trends in 1990 and 2019

Characteristics	1990		2019			1990–2019	
	**ASIR** ^**†**^ **(per 100000)**		**ASIR** ^**†**^ **(per 100000)**			**EAPC** ^**†**^	**EAPC** ^**†**^
	**No. (95% UI)**	**Male/female ratio**	**No. (95% UI)**	**Male/female ratio**	**Numbers**	**Numbers**	**ASIR**
**Global**	5.11(6.81,3.67)	2.163848249	5.24(6.94,3.78)	2.117627407	436056.22(314528.67,579116.38)	0.83(0.93,0.73)	0.10(0.03,0.18)
**Sex**	-	-	-	-		-	-
Male	6.94(9.30,5.00)	-	7.10(9.39,5.11)	-	294821.67(212463.74,390611.44)	0.77(0.87,0.69)	0.12(0.05,0.19)
Female	3.21(4.31,2.26)	-	3.35(4.51,2.38)	-	141234.55(100237.67,191832.76)	0.95(1.07,0.83)	0.12(0.05,0.19)
**Sociodemographic index**	-	-	-	-		-	-
Low SDI	2.89(4.44,1.59)	1.405028231	3.70(5.42,2.25)	1.536415044	27965.31(17184.31,40461.89)	3.95(3.77,4.13)	0.95(0.82,1.07)
Low-middle SDI	3.50(5.15,2.07)	1.88369469	5.05(7.12,3.26)	1.962767078	85038.25(55729.86,119001.80)	3.93(3.77,4.08)	1.42(1.26,1.58)
Middle SDI	4.12(5.92,2.63)	2.099064202	4.91(6.64,3.46)	1.996287414	137128.88(95894.88,185308.32)	3.23(3.17,3.29)	0.76(0.66,0.86)
High-middle SDI	6.08(7.91,4.53)	2.470377455	5.99(7.69,4.47)	2.799392843	107703.78(80404.64,139469.42)	1.61(1.56,1.65)	0.01(-0.07,0.09)
High SDI	8.01(9.93,6.11)	2.14013889	6.64(8.24,5.12)	1.777364786	77981.04(60445.80,96792.78)	0.07(-0.15,0.28)	-0.76(-0.89,-0.63)
**Region**	-	-	-	-		-	-
Andean Latin America	7.94(10.53,5.70)	1.840028953	13.04(16.73,9.57)	1.54157587	8011.97(5882.02,10287.18)	4.72(4.54,4.90)	1.74(1.57,1.92)
Australasia	1.77(2.54,1.14)	2.528068665	1.68(2.43,1.08)	2.301556592	570.94(358.52,832.10)	1.52(1.34,1.69)	-0.01(-0.21,0.18)
Caribbean	6.45(8.60,4.53)	2.293052765	7.65(10.07,5.36)	2.542985463	3872.72(2700.11,5105.70)	2.37(2.26,2.48)	0.40(0.33,0.47)
Central Asia	9.31(12.44,6.61)	1.550531584	21.15(27.16,15.58)	1.628826439	20847.07(15313.19,26878.64)	5.68(5.48,5.89)	3.20(3.09,3.31)
Central Europe	10.90(13.61,8.24)	2.384052075	11.75(14.35,9.17)	2.794395136	16176.03(12529.59,19820.41)	0.40(0.25,0.56)	0.44(0.28,0.61)
Central Latin America	12.96(17.07,9.29)	1.776326302	13.17(17.37,9.59)	1.409585584	34414.35(25083.76,45444.58)	2.88(2.79,2.97)	0.21(0.16,0.26)
Central sub-Saharan Africa	2.55(4.22,1.33)	1.929339393	3.53(5.66,2.02)	2.400252822	3002.18(1775.58,4744.70)	4.39(4.09,4.68)	0.94(0.71,1.18)
East Asia	3.52(5.15,2.17)	3.085861515	3.61(4.97,2.51)	3.335992611	71957.42(48544.53,100701.55)	2.31(2.24,2.38)	0.25(0.06,0.44)
Eastern Europe	5.77(7.95,3.85)	2.443791771	9.47(13.74,6.01)	2.992069581	22595.81(14106.76,33253.03)	1.71(1.60,1.81)	2.01(1.90,2.12)
Eastern sub-Saharan Africa	3.76(5.94,2.01)	1.175879545	4.58(7.04,2.64)	1.326653922	11026.98(6624.22,16177.73)	3.70(3.46,3.94)	0.67(0.53,0.80)
High-income Asia Pacific	8.50(10.65,6.48)	2.206560694	5.88(7.41,4.46)	1.92415334	13294.79(10046.73,16922.92)	-1.34(-1.52,-1.16)	-1.73(-1.91,-1.54)
High-income North America	6.03(8.05,4.30)	2.139080434	5.45(7.18,4.02)	1.441088721	21764.68(15914.16,28940.01)	0.40(0.17,0.64)	-0.39(-0.48,-0.30)
North Africa and middle East	1.01(1.55,0.60)	1.472407947	1.54(2.34,0.93)	1.617969932	8390.63(5079.31,12891.11)	5.06(4.93,5.20)	1.23(1.08,1.38)
Oceania	1.10(1.77,0.62)	4.203151147	1.04(1.65,0.60)	4.417238558	126.52(73.12,197.85)	2.82(2.72,2.91)	-0.28(-0.35,-0.22)
South Asia	2.83(4.52,1.39)	1.649439554	4.53(6.88,2.61)	1.787620385	79692.91(45485.71,118869.41)	4.56(4.30,4.81)	1.38(1.23,1.52)
Southeast Asia	3.48(5.25,2.02)	1.706206988	4.17(5.83,2.75)	1.895015814	31158.67(20303.33,44006.39)	3.33(3.29,3.36)	0.62(0.54,0.70)
Southern Latin America	7.06(9.99,4.63)	2.864880066	9.09(12.61,6.03)	2.796626778	6733.03(4447.7,9378.51)	2.49(2.45,2.54)	0.81(0.76,0.86)
Southern sub-Saharan Africa	2.42(3.84,1.26)	2.880602084	2.34(3.59,1.31)	2.850940963	1717.03(1000.02,2566.40)	2.00(1.82,2.19)	-0.29(-0.54,-0.04)
Tropical Latin America	8.49(11.98,5.55)	4.553455696	8.25(11.29,5.66)	5.276983843	21311.62(14571.79,29178.72)	2.10(1.97,2.22)	-0.22(-0.28,-0.15)
Western Europe	11.35(13.80,8.81)	2.020918728	9.87(12.00,7.72)	1.966719982	49106.67(38703.50,59916.62)	0.10(-0.14,0.33)	-0.59(-0.72,-0.46)
Western sub-Saharan Africa	3.15(4.95,1.64)	1.412170612	3.71(5.59,2.13)	1.656272712	10284.21(6165.65,15336.59)	3.71(3.51,3.90)	0.45(0.30,0.59)

^**†**^ASIR, age-standardized incidence rate; EAPC, estimated annual percentage change; NA, not available; UI, uncertainty interval

In 2019, the number of alcohol-induced cirrhosis and other chronic liver diseases incident cases was highest in South Asia(79692.91,95% CI, 45485.71–118869.41), East Asia(71957.42,95% CI, 48544.53–100701.55), and Western Europe(49106.67,95% CI, 38703.50–59916.62), while lowest in Oceania(126.52,95% CI, 73.12–197.85), Australasia(570.94,95% CI, 358.52–832.10), Southern sub-Saharan Africa (1717.03,95% CI, 1000.02–2566.40) (Table 1).

In addition, countries with the highest number of incident cases of alcohol-induced cirrhosis and other chronic liver diseases in 2019 were China(69310.00,95% CI, 6617.48–97801.04), India (68961.06,95% CI, 37913.26–104885.51) and the Mexico(24106.77,95% CI, 6809.70–32911.64), in exact order, while the lowest number of incident cases of alcohol-induced cirrhosis and other chronic liver diseases were in the following countries Tokelau(0.02,95% CI, 0.01–0.03), Niue (0.04,95% CI, 0.02–0.06) and Cook Islands (0.16,95% CI, 0.10–0.26) (Supplementary Table S3).

**Fig. 1:**
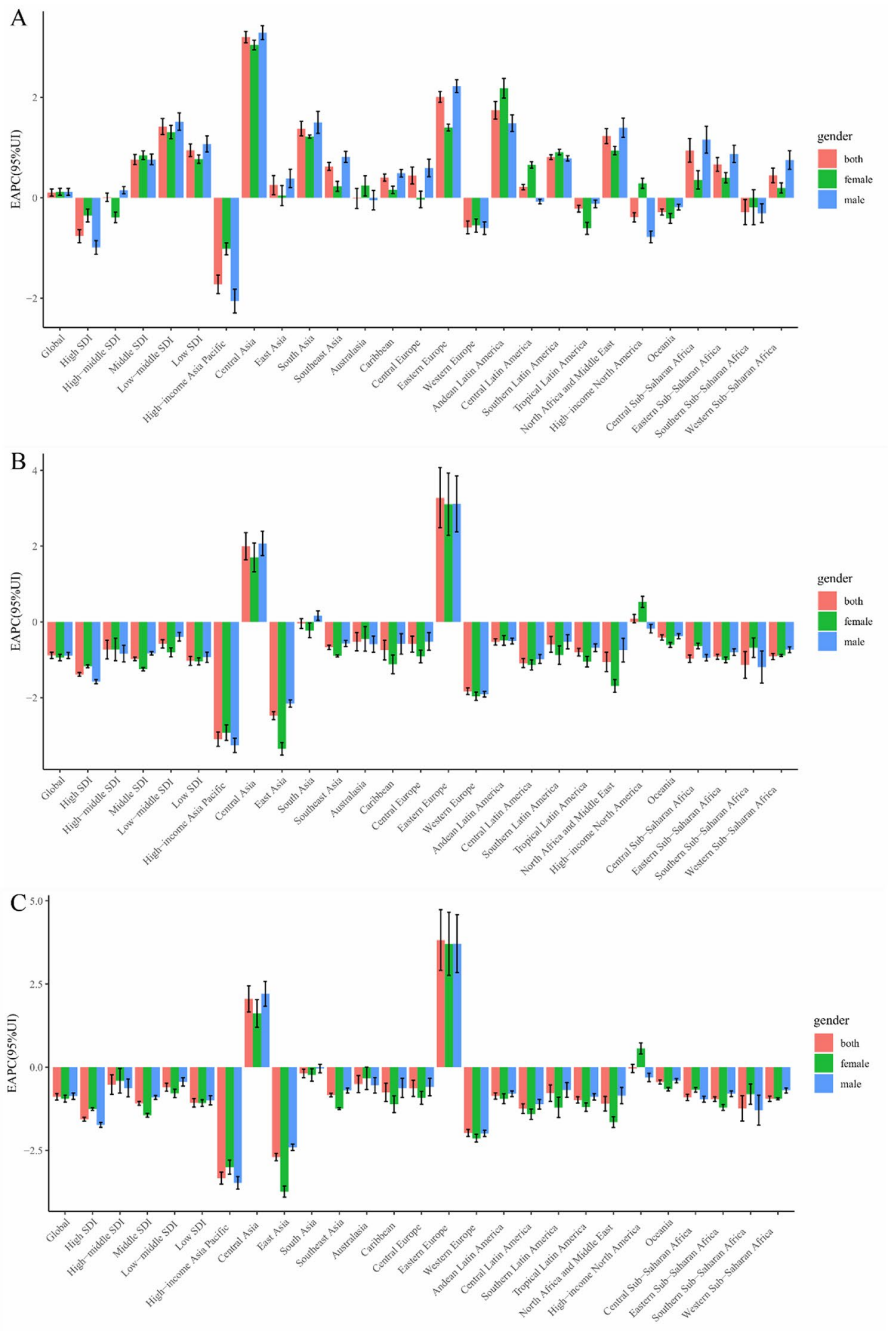
**The EAPC of cirrhosis and other chronic liver diseases due to alcohol use ASRs from 1990 to 2019, by sex and region.** (A) The EAPC of ASIR. (B) The EAPC of ASDR. (C) The EAPC of age-standardized DALY rate. EAPC = estimated annual percentage change. ASRs = age-standardized rates. ASIR = age standardized incidence rate. ASDR = age standardized death rate. DALY = disability adjusted life-year

From 1990 to 2019, the age-standardized incidence rate of alcohol-induced cirrhosis and other chronic liver diseases reflected an ascendant trend globally (EAPC [95% CI], 0.10[0.03, 0.18]; Table 1; Fig. 1A, Supplementary Fig. 1A, Additional file 1). The age-standardized incidence rate for females (EAPC [95% CI], 0.12 [0.05, 0.19]) increased during this period and the age-standardized incidence rate for males (EAPC [95% CI] 0.12 [0.05, 0.19]) showed an upward trend (Table 1; Fig. 1A, Supplementary Fig. 1A, Additional file 1).

The significant growth in the age-standardized incidence of alcohol-induced cirrhosis and other chronic liver diseases from 1990 to 2019 was observed in countries in the low-middle SDI quintile (EAPC, 1.42; 95% CI, 1.26 to 1.58; Table 1). In a high SDI countries, it had the tallest age-standardized incidence (8.01 in 1990 and 6.64 in 2019), whereas the minimum level of age-standardized incidence was reflected in countries with a low SDI (2.89 in 1990 and 3.70 in 2019; Table 1). The age-standardized incidence of these diseases in countries in the low, low-middle, middle and high-middle SDI quintiles continued to increase over time (Table 1).

**Fig. 2:**
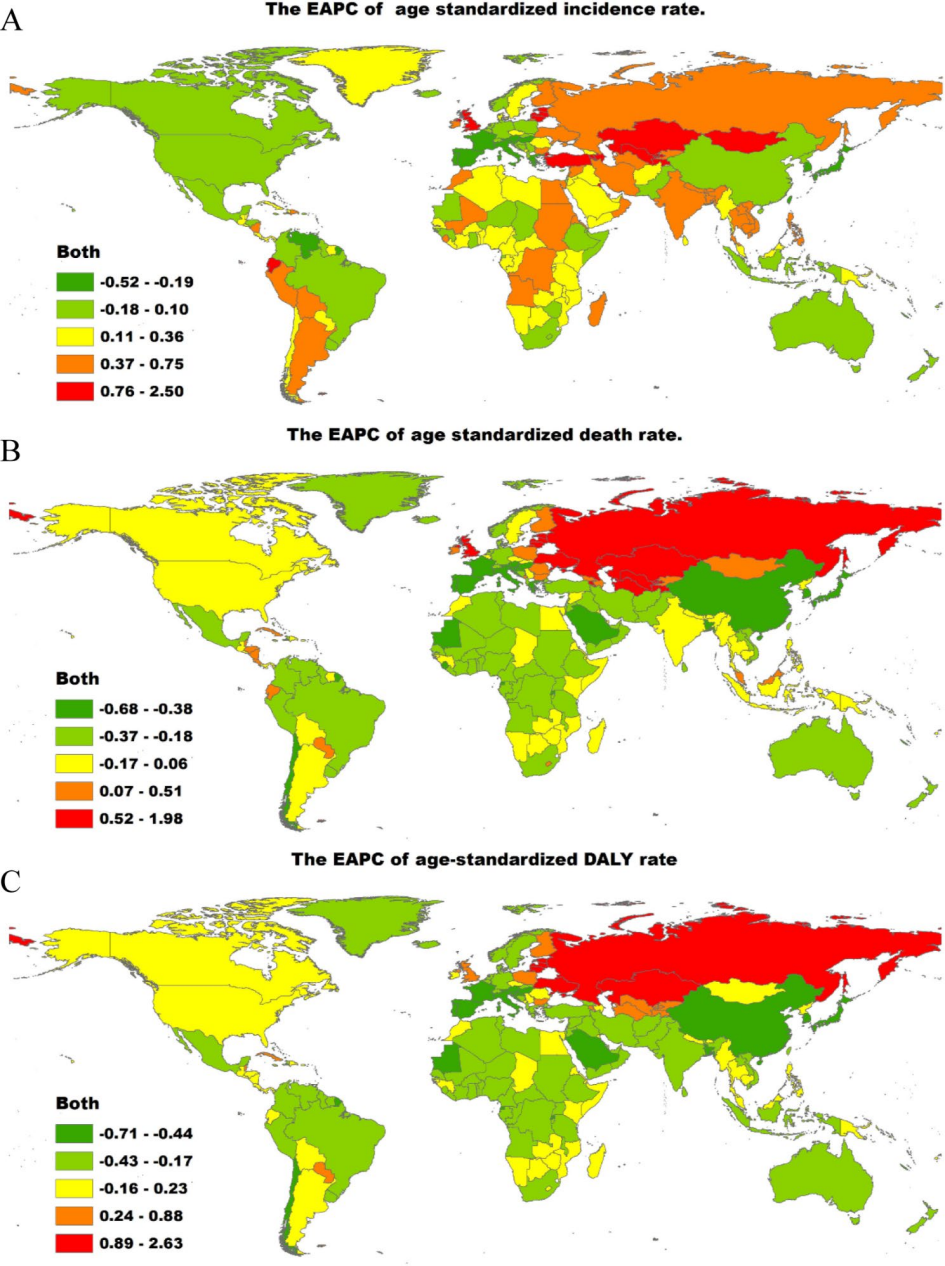
**The global map of EAPC of cirrhosis and other chronic liver diseases due to alcohol use age-standardized rates from 1990 to 2019, by countries.** (A) The EAPC of ASIR. (B) The EAPC of ASDR. (C) The EAPC of age-standardized DALY rate. EAPC = estimated annual percentage change. ASIR = age standardized incidence rate. ASDR = age standardized death rate. DALY = disability adjusted life-year

The region with the most obvious growth in the incidence of alcohol-induced cirrhosis and other chronic liver diseases from 1990 to 2019 was Central Asia (EAPC = 3.20), whereas the region with the smallest increase in the incidence of these diseases was South Asia (EAPC = 1.82, Supplementary Tables 2, Additional file 2). At the global level, the age-standardized incidence rate of alcohol-induced cirrhosis and other chronic liver diseases reported a rising trend in most countries from 1990 to 2019 (Supplementary Tables 3, Additional file 2). As presented in Fig. 2A, Supplementary Tables 1 and 3 (Additional file 2), the largest decrease in the age-standardized incidence of these diseases was in the Republic of Korea (total: EAPC, − 3.55; females: EAPC, − 2.86; males: EAPC, − 3.82), while the largest increase was observed in Kazakhstan (total: EAPC, 5.29; females: EAPC, 4.86; male: EAPC, 5.54).

However, the age-standardized increase in the incidence of alcohol-induced cirrhosis and other chronic liver diseases from 1990 to 2019 was higher for males than for females. Besides, the ratio of male to female was 2.16 and 2.12 in 1990 and 2019, respectively (Table 1). The male-to-female ratio of the incidence of alcohol-induced cirrhosis and other chronic liver diseases among different age groups showed a bimodal distribution globally, with a highest point in the 25–29-year age range (Supplementary Fig. 2A, Additional file 1). The ratio of male to female incidence among different age groups in 2019 among different SDI regions were shown in Supplementary Fig. 2B,C,D,E,F.

Distribution of different ages in cirrhosis and other chronic liver diseases due to alcohol use incidence in global and different SDI regions were shown in Supplementary Fig. 3. The EAPC in the age-standardized incidence of cirrhosis and alcohol-induced other chronic liver diseases was found to be negatively involved with the age-standardized incidence of these diseases (ρ = −0.273, P = 0.000, Supplementary Fig. 4A, Additional file 1) and the SDI of the region (ρ = −0.152, P = 0.030, Supplementary Fig. 4B, Additional file 1). We also noticed that regions with a higher SDI reflected a higher incidence of alcohol-induced cirrhosis and other chronic liver diseases in young people. Moreover, regions in which the SDI increased from 1990 to 2019 had a higher incidence of these diseases in younger adults (Supplementary Fig. 5A and B, Additional file 1). The annual ratios of the incidence of alcohol-induced cirrhosis and other chronic liver diseases between young people and older adults were relatively stable from year to year (Supplementary Fig. 6A, Additional file 1).

### Change in the death of the alcohol-induced cirrhosis and other chronic liver diseases

Globally, the number of deaths of alcohol-induced cirrhosis and other chronic liver diseases decreased from 232949.27 (95% CI, 197746.10-273817.39) in 1990 to 371964.34 (95% CI, 314703.31-438425.29) in 2019. Based on these values, the number of deaths due to these diseases increased by 59.68% from 1990 to 2019.

In 2019, the number of death cases of alcohol-induced cirrhosis and other chronic liver diseases was highest in the middle SDI region(103750.53,95% CI, 86644.14–123232.19) and lowest in the low SDI region(37093.52,95% CI, 28642.20–47467.77) (Table 2 ). Between 1990 and 2019, the deahts number of alcohol-induced cirrhosis and other chronic liver diseases in all SDI regions increased. Notably, the low-middle SDI region [EAPC,2.19 (95% UI 2.08–2.30)] had a tremendous increase in the number of alcohol-induced cirrhosis and other chronic liver diseases changes from 1990 to 2019 (Table 2).

**Table 2:** Age-standardized mortality rates (ASDR) of alcohol-induced cirrhosis and other chronic liver diseases and their time trends in 1990 and 2019

Characteristics	1990		2019			1990–2019	
	**ASDR** ^**†**^ **(per 100000)**		**ASDR** ^**†**^ **(per 100000)**			**EAPC** ^**†**^	**EAPC** ^**†**^
	**No. (95% UI)**	**Male/female ratio**	**No. (95% UI)**	**Male/female ratio**	**Numbers**	**Numbers**	**ASDR**
**Global**	5.67(6.64,4.80)	3.066431238	4.48(5.28,3.81)	3.098623709	371964.34(314703.31,438425.29)	0.60(0.72,0.49)	-0.88(-0.97,-0.80)
**Sex**	-	-	-	-		-	-
Male	8.79(10.23,7.45)	-	6.93(8.09,5.91)	-	274729.01(234217.53,321151.79)	0.60(0.75,0.47)	-0.89(-0.97,-0.81)
Female	2.87(3.44,2.35)	-	2.24(2.73,1.82)	-	97235.33(79336.79,118950.13)	0.59(0.74,0.46)	-0.94(-1.03,-0.85)
**Sociodemographic index**	-	-	-	-		-	-
Low SDI	9.13(11.95,6.92)	2.494870409	6.89(8.86,5.28)	2.528100908	37093.52(28642.20,47467.77)	1.66(1.57,1.75)	-1.03(-1.15,-0.91)
Low-middle SDI	7.06(8.61,5.71)	2.824431718	6.05(7.25,4.97)	3.095427519	86184.83(70719.63,104525.57)	2.19(2.08,2.30)	-0.58(-0.69,-0.47)
Middle SDI	5.27(6.21,4.48)	2.810581164	4.08(4.82,3.44)	3.130496057	103750.53(86644.14,123232.19)	1.99(1.94,2.03)	-0.98(-1.02,-0.93)
High-middle SDI	5.22(5.93,4.53)	3.240862113	4.24(4.82,3.69)	3.186558057	84999.09(73671.62,96976.43)	1.40(1.16,1.64)	-0.73(-0.98,-0.49)
High SDI	5.09(5.81,4.35)	3.263788596	3.54(4.10,3.01)	2.935438559	59697.87(50936.08,69757.94)	0.48(0.45,0.52)	-1.39(-1.44,-1.34)
**Region**	-	-	-	-		-	-
Andean Latin America	11.94(15.25,9.06)	2.732635153	10.19(13.50,7.37)	2.61122218	5730.77(4177.77,7545.05)	2.87(2.79,2.95)	-0.53(-0.61,-0.44)
Australasia	1.33(1.76,0.98)	3.42956826	1.01(1.36,0.72)	3.357314601	454.28(326.34,605.52)	1.93(1.68,2.18)	-0.53(-0.77,-0.28)
Caribbean	8.64(10.54,6.78)	2.886864667	7.47(9.54,5.61)	3.382955955	3876.99(2907.81,4953.88)	1.63(1.39,1.87)	-0.75(-1.00,-0.49)
Central Asia	9.58(11.48,7.63)	2.138630584	17.22(20.69,13.83)	2.20521592	13635.88(10920.70 ,16310.78)	3.67(3.43,3.92)	2.00(1.64,2.35)
Central Europe	9.16(10.41,7.79)	2.829703803	8.47(10.08,7.05)	3.233311763	16263.30(13494.19,19309.86)	0.38(0.18,0.58)	-0.59(-0.80,-0.37)
Central Latin America	12.97(14.46,11.49)	3.345511699	10.31(12.40,8.44)	3.479533903	24929.81(20398.04,29903.97)	2.34(2.23,2.45)	-1.09(-1.21,-0.97)
Central sub-Saharan Africa	9.72(13.92,6.46)	4.836463859	7.66(11.33,4.82)	4.533487715	4396.75(2793.72,6537.65)	2.01(1.82,2.20)	-0.97(-1.07,-0.87)
East Asia	3.13(3.89,2.52)	3.624914329	1.59(2.00,1.23)	4.941444772	33393.96(25686.26,42153.20)	0.34(0.23,0.44)	-2.47(-2.58,-2.37)
Eastern Europe	4.06(4.54,3.57)	2.691339492	10.05(11.75,8.57)	2.793140004	30938.43(26372.11,36115.54)	3.52(2.78,4.27)	3.27(2.48,4.07)
Eastern sub-Saharan Africa	11.98(16.06,8.75)	2.522382847	9.43(12.43,7.12)	2.653851524	15615.89(11873.13,20739.34)	1.74(1.65,1.84)	-0.92(-0.99,-0.85)
High-income Asia Pacific	5.11(5.91,4.33)	4.507051566	2.42(2.84,2.01)	4.309830826	9267.74(7687.65,11015.10)	-0.96(-1.14,-0.78)	-3.10(-3.29,-2.91)
High-income North America	3.51(4.02,3.03)	3.110832171	3.29(3.87,2.78)	2.626792734	19013.41(15971.64,22481.76)	2.15(2.00,2.29)	0.09(-0.02,0.20)
North Africa and middle East	2.22(3.31,1.44)	1.895957585	1.73(2.64,1.05)	2.257974755	7027.43(4297.40,10672.07)	2.55(2.42,2.67)	-1.06(-1.31,-0.80)
Oceania	2.93(4.13,2.02)	4.679495011	2.54(3.68,1.65)	4.988374641	216.29(138.71,313.44)	2.73(2.60,2.85)	-0.41(-0.48,-0.34)
South Asia	6.51(7.90,5.32)	2.599657419	5.16(6.39,4.18)	2.944605545	75580.56(61161.24,94157.34)	2.09(1.88,2.30)	-0.05(-0.18,0.08)
Southeast Asia	6.91(8.77,5.31)	2.337554718	5.73(7.28,4.51)	2.607984139	36506.67(28202.65,46305.19)	2.22(2.15,2.29)	-0.67(-0.73,-0.61)
Southern Latin America	6.72(8.54,4.92)	4.202630872	5.37(6.98,3.86)	4.686285704	4371.79(3127.31 ,5690.57)	1.36(1.11,1.61)	-0.59(-0.80,-0.38)
Southern sub-Saharan Africa	4.81(6.56,3.42)	3.613545808	3.68(4.51,2.96)	3.640255216	2173.99(1751.24 ,2693.28)	1.19(0.80,1.58)	-1.14(-1.49,-0.78)
Tropical Latin America	9.47(10.24,8.75)	5.790308818	7.20(7.89,6.60)	6.368239509	17988.56(16489.10,19662.73)	2.34(2.23,2.45)	-0.80(-0.90,-0.70)
Western Europe	6.68(7.56,5.72)	2.965951346	4.22(4.86,3.59)	2.913718357	32964.64(27987.11,38416.17)	-0.46(-0.52,-0.39)	-1.83(-1.92,-1.74)
Western sub-Saharan Africa	11.91(16.56,8.30)	2.849760704	9.04(11.94,6.60)	2.944361364	17617.22(12608.39,23416.08)	1.73(1.69,1.77)	-0.91(-1.00,-0.83)

^**†**^ASDR, age-standardized death rate; EAPC, estimated annual percentage change; NA, not available; UI, uncertainty interval.

In 2019, the number of alcohol-induced cirrhosis and other chronic liver diseases death cases was highest in South Asia (75580.56,95% CI, 61161.24–94157.34), Southeast Asia(36506.67,95% CI, 28202.65–46305.19), and East Asia(33393.96,95% CI, 25686.26–42153.20), while lowest in Oceania(216.29,95% CI, 138.71–313.44), Australasia(454.28,95% CI, 326.34–605.52), and Southern sub-Saharan Africa (2173.99,95% CI, 1751.24–2693.28) (Table 2).

In addition, countries with the highest number of death cases of alcohol-induced cirrhosis and other chronic liver diseases in 2019 were India(61527.26,95% CI, 48695.18–77858.97), China (30548.39,95% CI,23231.35–38769.91), and Russian Federation(19609.16,95% CI, 16272.40–23478.68), in exact order, while the lowest number of death cases of alcohol-induced cirrhosis and other chronic liver diseases were in the following countries Tokelau(0.04,95% CI,0.02–0.05) ,Niue(0.08,95% CI,0.05–0.11) and Nauru(0.29,95% CI,0.12–0.50), (Supplementary Table S4).

The age-standardized death rate of alcohol-induced cirrhosis and other chronic liver diseases showed a downward trend globally from 1990 to 2019 (EAPC, − 0.88; 95% CI, − 0.97 to − 0.80; Table 2; Fig. 1B, Supplementary Fig. 1B, Additional file 1). The age-standardized death rate for females (EAPC, − 0.94; 95% CI, − 1.03 to − 0.85) declined in this time period and the age-standardized death rate for males (EAPC, − 0.89; 95% CI, − 0.97 to − 0.81) showed a downward trend (Table 2; Fig. 1B, Supplementary Fig. 1B, Additional file 1).

The most obvious decline in the age-standardized death rate of alcohol-induced cirrhosis and other chronic liver diseases from 1990 to 2019 was noticed in countries with the highest SDI quintile (EAPC, − 1.39; 95% CI, − 1.44 to − 1.34; Table 2). Low-SDI countries revealed the tallest age-standardized death rate (9.13 in 1990 and 6.89 in 2019), whereas high-SDI countries revealed the lowest age-standardized death rate (5.09 in 1990 and 3.54 in 2019; Table 2). The age-standardized death rates in countries in all SDI quintiles continued to decrease over time (Table 2).

However, the age-standardized death rate of alcohol-induced cirrhosis and other chronic liver diseases from 1990 to 2019 was higher for males than for females, as demonstrated by the ratio of male to female was 3.07 and 3.10 in 1990 and 2019, respectively (Table 2). The male-to-female ratio of deaths of alcohol-induced cirrhosis and other chronic liver diseases among different age ranges showed a bimodal distribution, globally, with a highest point in the 35–39-year age range (Supplementary Fig. 7A, Additional file 1). The ratio of male to female death among different age groups in 2019 among different SDI regions were shown in Supplementary Fig. 7B,C,D,E,F.

Furthermore, the age-standardized death rates and trends varied between countries. The three countries with the largest decreases in the number of deaths of alcohol-induced cirrhosis and other chronic liver diseases from 1990 to 2019 were the Republic of Korea (EAPC, − 5.07), Portugal (EAPC, − 3.56) and Hungary (EAPC, − 3.49; Supplementary Tables 1, Additional file 2).

At the global level, from 1990 to 2019, the age-standardized death rates of alcohol-induced cirrhosis and other chronic liver diseases reflected a declining trend in most countries (Supplementary Tables 4, Additional file 2). As presented in Fig. 2B, Supplementary Tables 1 and 4 (Additional file 2), the most obvious decline in the age-standardized death rate was in the Republic of Korea (total: EAPC, − 5.07; females: EAPC, − 4.76; males: EAPC, − 5.39), while the largest increase was in Lithuania (total: EAPC, 4.61; females: EAPC, 4.19; male: EAPC, 4.64). Among females, the most pronounced declines and rises in the age-standardized death rates were in Bermuda (EAPC, − 5.07) and Belarus (EAPC, 4.43), respectively. The region with the most obvious change in death rate of alcohol-induced cirrhosis and other chronic liver diseases from 1990 to 2019 was Central Asia (1.96), whereas the smallest change was observed in Western Europe (− 0.07; Supplementary Tables 2, Additional file 2).

Distribution of different ages in cirrhosis and other chronic liver diseases due to alcohol use death in global and different SDI regions were shown in Supplementary Fig. 8. The EAPC in the age-standardized death rate of alcohol-induced cirrhosis and other chronic liver diseases was found to be negatively correlated with the age-standardized death rate owing to these diseases (ρ = −0.160, P = 0.022, Supplementary Fig. 4C, Additional file 1), while the EAPC in the age-standardized death rate of alcohol-induced cirrhosis and other chronic liver diseases was found to be positively related to the SDI of the region (ρ = 0.004, P = 0.955, Supplementary Fig. 4D, Additional file 1). We also found that regions with a higher SDI revealed more proportion of cases of alcohol-induced cirrhosis and other chronic liver diseases in young people, and that regions in which the SDI increased from 1990 to 2019 had a higher proportion of cases of these diseases in older adults (Supplementary Fig. 5C and D, Additional file 1). The annual ratios of deaths of alcohol-induced cirrhosis and other chronic liver diseases in young people and older adults were relatively stable from year to year (Supplementary Fig. 6B, Additional file 1).

### Change in the DALYs of alcohol-induced cirrhosis and other chronic liver diseases

Globally, the number of DALYs of alcohol-induced cirrhosis and other chronic liver diseases decreased from 7312093.40 (95% UI, 6168572.68-8541530.54) in 1990 to 11186601.49 (95% CI, 9447032.51-13125597.63) in 2019. Based on these values, the number of DALYs of alcohol-induced cirrhosis and other chronic liver diseases increased by 52.99% from 1990 to 2019.

In 2019, the number of DALY cases of alcohol-induced cirrhosis and other chronic liver diseases was highest in the middle SDI region(3107518.22,95% CI, 2614771.30–3680878.40) and lowest in the low SDI region(1144560.86,95% CI, 880582.09–1460660.04) (Table 3 ). Between 1990 and 2019, the DALYs number of alcohol-induced cirrhosis and other chronic liver diseases in all SDI regions increased. Notably, the low-middle SDI region [EAPC,2.03 (95% UI 1.90–2.15)] had a tremendous increase in the number of alcohol-induced cirrhosis and other chronic liver diseases changes from 1990 to 2019 (Table 3).

**Table 3:** Age-standardized DALY rates of alcohol-induced cirrhosis and other chronic liver diseases and their time trends in 1990 and 2019

Characteristics	1990		2019			1990–2019	
	**age-standardized DALY** ^**†**^ **rate (per 100000)**		**age-standardized DALY** ^**†**^ **rate (per 100000)**			**EAPC**	**EAPC**
	**No. (95% UI)**	**Male/female ratio**	**No. (95% UI)**	**Male/female ratio**	**Numbers**	**Numbers**	**Age-standardized DALY rates**
**Global**	168.97(197.60,142.05)	3.311659181	133.31(156.17,112.68)	3.390408457	11186601.49(9447032.51,13125597.63)	0.53(0.66,0.42)	-0.89(-0.99,-0.79)
**Sex**	-	-	-	-		-	-
Male	262.45(304.61,221.80)	-	208.08(242.09,177.47)	-	8542105.79(7267110.49,9943860.48)	0.53(0.67,0.42)	-0.87(-0.97,-0.78)
Female	79.25(95.33,64.37)	-	61.37(73.95,50.44)	-	2644495.70(2165566.74,3191927.67)	0.52(0.65,0.40)	-0.95(-1.06,-0.85)
**Sociodemographic index**	-	-	-	-		-	-
Low SDI	252.00(326.34,191.36)	2.752818639	187.08(239.70,144.17)	2.779223086	1144560.86(880582.09,1460660.04)	1.66(1.57,1.76)	-1.07(-1.20,-0.95)
Low-middle SDI	209.81(254.64,170.96)	3.172746092	178.11(217.29,145.56)	3.412846485	2732196.12(2225787.73,3321650.49)	2.03(1.90,2.15)	-0.61(-0.73,-0.48)
Middle SDI	152.94(180.20,128.62)	3.17185458	115.48(136.31,97.40)	3.632013079	3107518.22(2614771.30,3680878.40)	1.70(1.65,1.75)	-1.09(-1.15,-1.03)
High-middle SDI	154.13(175.67,133.63)	3.513546394	132.66(150.02,115.32)	3.41297522	2605011.58(2256045.98,2953295.87)	1.45(1.14,1.76)	-0.53(-0.82,-0.23)
High SDI	155.70(177.93,132.85)	3.365082415	103.31(119.04,87.66)	2.997957205	1590406.86(1340106.25,1836185.46)	0.10(0.04,0.16)	-1.57(-1.63,-1.51)
**Region**	-	-	-	-		-	-
Andean Latin America	335.56(431.11,252.65)	2.920745739	264.39(345.65,190.73)	2.930730013	153515.09(110940.38,200565.64)	2.35(2.26,2.44)	-0.87(-0.96,-0.77)
Australasia	38.96(52.09,28.27)	3.517952027	28.99(39.04,20.10)	3.374222837	12048.80(8499.12,16363.17)	1.72(1.47,1.97)	-0.51(-0.77,-0.26)
Caribbean	247.90(302.59,191.11)	3.133749271	213.61(275.12,157.57)	3.628534387	110702.19(81694.74,142908.75)	1.50(1.26,1.74)	-0.76(-1.04,-0.49)
Central Asia	273.62(328.68,215.81)	2.181216873	505.54(603.58,406.56)	2.380272195	445241.32(354749.56,538226.85)	3.98(3.69,4.27)	2.05(1.66,2.45)
Central Europe	278.57(320.72,237.44)	3.008414368	259.32(308.35,217.76)	3.420029677	461353.24(384272.29,548873.39)	0.10(-0.14,0.34)	-0.64(-0.88,-0.39)
Central Latin America	389.86(435.40,343.51)	3.757892393	297.28(359.02,241.08)	4.070670233	740808.25(598619.10,896311.18)	1.95(1.82,2.07)	-1.25(-1.40,-1.11)
Central sub-Saharan Africa	264.73(374.58,176.38)	4.792392548	211.61(313.51,134.99)	4.541848893	140555.08(90006.03,208070.37)	2.18(2.00,2.37)	-0.91(-1.00,-0.81)
East Asia	93.66(116.40,74.63)	4.194438192	44.80(56.25,34.85)	5.931196672	961802.17(747549.55,1216087.11)	-0.08(-0.18,0.02)	-2.71(-2.81,-2.60)
Eastern Europe	124.86(140.16,110.21)	2.696215012	364.77(427.85,308.53)	2.863648752	1062289.82(901536.25,1244353.12)	3.94(3.07,4.81)	3.82(2.91,4.73)
Eastern sub-Saharan Africa	312.70(419.23,226.35)	2.633082923	243.60(322.39,185.61)	2.902503211	464507.77(350444.84,616490.47)	1.81(1.71,1.90)	-0.96(-1.03,-0.89)
High-income Asia Pacific	154.75(181.14,129.16)	5.293913019	69.31(81.60,56.88)	5.03385982	224872.60(186155.92,263116.24)	-1.84(-2.02,-1.66)	-3.34(-3.52,-3.16)
High-income North America	106.87(123.24,91.69)	3.19508651	95.83(113.39,80.53)	2.604167243	517380.98(432830.05,615645.31)	1.94(1.78,2.11)	-0.04(-0.16,0.08)
North Africa and middle East	50.39(75.01,32.94)	2.078251675	39.03(59.82,23.77)	2.394217365	179033.90(109338.74,274663.58)	2.48(2.36,2.61)	-1.10(-1.32,-0.88)
Oceania	93.77(131.52,63.67)	5.307822603	80.54(116.92,51.95)	5.702118102	7806.33(5052.25,11462.58)	2.73(2.63,2.84)	-0.45(-0.51,-0.39)
South Asia	196.39(236.71,161.05)	3.058238149	153.43(190.68,123.69)	3.299900814	2447028.30(1970290.63, 3056505.82)	1.87(1.63,2.10)	-0.19(-0.32,-0.06)
Southeast Asia	203.94(261.22,157.07)	2.640535668	162.04(205.71,125.51)	3.103166004	1122437.85(867218.59,1447913.35)	1.99(1.95,2.03)	-0.84(-0.90,-0.79)
Southern Latin America	197.63(252.83,143.78)	4.31351478	148.28(193.35,106.88)	5.071380767	117796.66(84867.76,152808.91)	1.07(0.78,1.35)	-0.78(-1.03,-0.54)
Southern sub-Saharan Africa	139.68(189.22,101.41)	3.645606443	103.61(128.08,82.91)	3.820516159	66751.25(53604.17,82609.20)	1.09(0.66,1.52)	-1.24(-1.62,-0.86)
Tropical Latin America	301.72(324.58,279.74)	6.955604594	219.74(239.57,201.70)	7.559922187	559689.18(513129.08,610186.70)	1.88(1.77,1.99)	-0.99(-1.08,-0.89)
Western Europe	201.13(228.10,170.99)	2.930954464	123.15(140.82,104.32)	2.966100139	853109.40(720659.68,975413.55)	-0.78(-0.87,-0.70)	-1.98(-2.08,-1.88)
Western sub-Saharan Africa	318.32(442.61,219.97)	3.05891016	238.20(317.24,170.77)	3.238501701	537871.31(383196.77,719722.07)	1.88(1.83,1.92)	-0.95(-1.03,-0.87)

^**†**^DALY, disability adjusted life-years; NA, not available; UI, uncertainty interval.

In 2019, the number of alcohol-induced cirrhosis and other chronic liver diseases DALY cases was highest in South Asia(2447028.30,95% CI, 1970290.63–3056505.82), Southeast Asia(1122437.85,95% CI, 867218.59–1447913.35), and Eastern Europe(1062289.82,95% CI, 901536.25–1244353.12), while lowest in Oceania(7806.33,95% CI, 5052.25–11462.58), Australasia(12048.80,95% CI, 8499.12–16363.17), and Southern sub-Saharan (66751.25,95% CI, 53604.17–82609.20) (Table 3).

In addition, countries with the highest number of DALY cases of alcohol-induced cirrhosis and other chronic liver diseases in 2019 were India(2025620.81,95% CI, 1589227.01–2565144.45) ,China(877551.34,95% CI, 671826.49–1114226.90) and Russian Federation(666445.50,95% CI, 550271.06–798352.19), in exact order, while the lowest number of DALY cases of alcohol-induced cirrhosis and other chronic liver diseases were in the following countries Tokelau(1.06,95% CI, 0.64–1.60), Niue(2.26,95% CI, 1.37–3.40), and Nauru (10.94,95% CI, 4.59–19.57) (Supplementary Table S5).

From 1990 to 2019, the age-standardized number of DALYs (EAPC, − 0.89; 95% CI, − 0.99 to − 0.79) revealed a downward trend (Table 3; Fig. 1C, Supplementary Fig. 1C, Additional file 1). In addition, the age-standardized number of DALYs (EAPC, − 0.95; 95% CI, − 1.06 to − 0.85) for females continued to decrease, while the age-standardized number of DALYs (EAPC, − 0.87; 95% CI, − 0.97 to − 0.78) for males also showed a decreasing trend (Table 3; Fig. 1C, Supplementary Fig. 1C, Additional file 1).

The tallest number of age-standardized DALYs of alcohol-induced cirrhosis and other chronic liver diseases was observed in low-SDI regions in 1990 (252.00) and 2019 (187.08). The lowest number of age-standardized DALYs was in the middle-SDI regions in 1990 (152.94) and in the high-SDI regions in 2019 (103.31, Table 3).

The three regions with the highest age-standardized number of DALYs of alcohol-induced cirrhosis and other chronic liver diseases in 2019 were Central Asia (505.54), Eastern Europe (364.77) and Central Latin America (297.28) (Supplementary Tables 2, Additional file 2), whereas those with the lowest age-standardized number of DALYs were Australasia (28.99), North Africa and Middle East (39.03) and East Asia (44.80; Supplementary Tables 2, Additional file 2). The largest increase in the age-standardized number of DALYs was in Eastern Europe (total: EAPC, 3.82; females: EAPC, 3.71; males: EAPC, 3.71), while the largest decrease was in the High-income Asia Pacific (total: EAPC, − 3.34; females: EAPC, − 3.01; males: EAPC, − 3.48). Among females, the largest decrease in the age-standardized number of DALYs was in East Asia (EAPC, − 3.74; Supplementary Tables 2, Additional file 2).

As shown in Fig. 2C, Supplementary Tables 1 and 5 (Additional file 2), females in Mongolia (519.95) and males in Turkmenistan (1043.21) revealed the tallest number of DALYs in 2019. The most obvious decline in the age-standardized number of DALYs was in the Republic of Korea (total: EAPC, − 5.33; females: EAPC, − 4.97; males: EAPC, − 5.54), while the largest increase was in Lithuania (total: EAPC, 4.82; females: EAPC, 4.38; males: EAPC, 4.88). Among females, the most pronounced drop and rise in the age-standardized number of DALYs were in Bermuda (EAPC, − 5.06) and Belarus (EAPC, 4.82), respectively.

However, the age-standardized number of DALYs of alcohol-induced cirrhosis and other chronic liver diseases from 1990 to 2019 was higher for males than for females, as demonstrated by the male-to-female ratios of 3.31 and 3.39 in 1990 and 2019, respectively (Table 3). The male-to-female ratio of the age-standardized number of DALYs of alcohol-induced cirrhosis and other chronic liver diseases across different age groups showed a bimodal distribution, globally, with a highest point in the 35–39-year age range (Supplementary Fig. 9A, Additional file 1). The ratio of male to female age standardized DALY rate among different age groups in 2019 among different SDI regions were shown in Supplementary Fig. 9B,C,D,E,F.

Distribution of different ages in cirrhosis and other chronic liver diseases due to alcohol use DALYs in global and different SDI regions were shown in Supplementary Fig. 10. The EAPC in the age-standardized number of DALYs of alcohol-induced cirrhosis and other chronic liver diseases was found to be negatively correlated with the age-standardized number of DALYs due to these diseases (ρ = −0.159, P = 0.023, Supplementary Fig. 4E, Additional file 1) and the SDI of the region (ρ = −0.018, P = 0.803, Supplementary Fig. 4F, Additional file 1). In 2019, low-SDI regions had the lowest proportion of DALYs of alcohol-induced cirrhosis and other chronic liver diseases in young people (15–49 years), whereas regions in which the SDI increased from 1990 to 2019 revealed more proportion of DALYs in older adults than other regions (Supplementary Fig. 5E and F, Additional file 1). The annual ratios of DALYs of alcohol-induced cirrhosis and other chronic liver diseases in young people and older adults were relatively stable from year to year (Supplementary Fig. 6C. Additional file 1).

## Discussion

Our study indicated that the global age-standardized incidence rate increased; however, both the age-standardized death rate and DALYs rate alcohol-induced cirrhosis and other chronic liver diseases showed decreasing trends. Low and low-middle SDI regions had a much higher age-standardized DALYs rate. Eastern Europe, the region saw the largest increases in age-standardized death rate and the number of DALYs. The country with the largest increase in deaths of alcohol-induced cirrhosis and other chronic liver diseases was Lithuania. The age-standardized death and DALYs rates were higher for males than for females.

. In consistent with our findings, the website of GBD 2019 also reported the global incidence, prevalence, DAaLYs numbers and age-standardized rates for cirrhosis and other chronic liver diseases due to alcohol use in 2019 [5], however, they focused on the total percentage change between 1990 and 2019, our study calculated the global EAPCs by performing linear regression of data from the GBD 2019 datasets. The EAPCs provide annual change about the temporal trends rather than the total percentage change during the study period, which offers more accurate information.

The global age-standardized incidence of these diseases is increasing, and this is expected to continue owing to increasing alcohol consumption, increasing population age, and changes in diet [6]. High-SDI regions had the highest incidence of alcohol-induced cirrhosis and other chronic liver diseases, which may be attributable to the better healthcare systems in these regions leading to a higher detection rate of these disease than in other regions. Low-SDI regions had the highest age-standardized mortality rates and DALYs associated with alcohol-induced cirrhosis and other chronic liver diseases, perhaps owing to the poorer medical facilities in these regions, leading to higher death rates and an increased disease burden than in other regions. Moreover, in low-SDI regions, alcohol products may be illegally manufactured at home; thus, the real alcohol intake in people from these regions may be undetectable [7]. Therefore, alcohol-related deaths and DALYs may be underestimated in low-SDI regions [7].

The greatest increase in the age-standardized mortality rate was observed in Lithuania, perhaps owing to the high alcohol consumption in this region [8]. Since the early 1990s, Lithuania has not implemented any control policies for the production and sale of alcohol [9]. The lack of legislation, weak border controls, and huge personal economic gains from alcohol sales have led to increasing levels of alcohol consumption in Lithuania [9]. According to data from the WHO, Lithuania has one of the world’s highest levels of alcohol consumption, with an estimated per capita consumption of 15 L in 2016 [10]. Therefore, Lithuania ranks highest in the European Union in terms of alcohol-related mortalities and DALYs. From January 1, 2016 to January 1, 2018, Lithuania implemented three effective alcohol-control measures, all of which are recommended by the WHO. Alcohol procurement is now regulated and taxes have been increased, thereby increasing the price and decreasing the supply of alcoholic beverages, and advertising of alcoholic beverages has been prohibited [11].

The incidence of alcohol-induced cirrhosis and other chronic liver diseases significantly differs based on age and sex. Because men generally consume more alcohol than women [12], in most age ranges analyzed the incidence, mortality, and DALYs associated with these diseases were higher in men than in women. However, women are reportedly more often affected by alcohol-induced liver injury than men [13]. Based on global data and data from low-SDI regions, 44–50-year-old men and 50–54-year-old women had the highest incidence of alcohol-induced cirrhosis and other chronic liver diseases, whereas men and women aged 60–64 had the highest mortality rate and DALYs.

Alcohol consumption is one of the major contributors to death and disease and is reportedly one of the top 10 avoidable risk factors in every global comparative risk assessment performed thus far [14]. The WHO has adopted a series of initiatives at the global level to reduce the effects of alcohol [15]. Three of these initiatives––taxation, reduction in utilization capacity, and restrictions on promotion––are highlighted as the most cost-effective measures to lessen the per capita consumption of alcohol. It is essential to obtain an accurate estimate of alcohol intake and the potential of liver disease in patients with heavy alcohol consumption.

The main risk factors for alcohol-induced cirrhosis and other chronic liver diseases are related to the level and method of alcohol consumption. For example, the risks associated with consuming large amounts of alcohol at once are greater than those associated with consuming smaller amounts at once. A recent study in 195 countries conducted from 1990 to 2016 on alcohol consumption and the associated mortality risk reported that, except for ischemic heart disease, the permissible level of alcohol consumption limit is 0 units/week; that is, any level of alcohol consumption can increase the overall risk of mortality [16]. Therefore, the general population should minimize their alcohol intake. Abstinence is the most important and fundamental strategy to treat alcohol-induced cirrhosis and other chronic liver diseases. To detect and treat these diseases as early as possible, patients with heavy alcohol consumption should be screened at alcohol withdrawal clinics.

We used the latest epidemiological data to study the burden of alcohol-induced cirrhosis and other chronic liver diseases and examined the association between SDI and these diseases. Moreover, our dataset covered was from 204 countries and regions, and used more data sources than previous studies. This analysis of epidemiological data will guide the effective use of resources and national health data for the treatment of alcohol-related cirrhosis and other chronic liver diseases.

Our study used data and methods from the GBD 2019 study and has certain limitations. First, the GBD 2019 study data were not obtained from all countries or regions across the world. Second, owing to differences between the data sources and the measures evaluated in the GBD 2019 study and those in other studies, our estimates may be higher than those of other studies. Third, the GBD data were obtained from various sites and systems; thus, underreporting might have occurred. Fourth, because the GBD 2019 study mainly included country- and regional-level data, we could not assess differences in terms of race or ethnicity. Fifth, the linear trends assumption in our study is problematic, as in many countries, such as Lithuania, the trends are not linear. Sixth, the uncertainty intervals for the EAPCs might be severely underestimated, as they disregarded the uncertainty of the underlying GBD estimates. Despite the abovementioned limitations, our findings are valuable because they provide a basis for formulating clinical policies and public health guidelines regarding the prevention and treatment of alcohol-induced cirrhosis and other chronic liver diseases.

## Conclusion

Our results highlight the burden of alcohol-induced cirrhosis and other chronic liver diseases in different countries and regions worldwide. Globally, both the age-standardized mortality rate and DALYs caused by alcohol-induced cirrhosis and other chronic liver diseases showed decreasing trends from 1990 to 2019. Future preventive strategies should focus on the male population, particularly in Eastern Europe, Lithuania, and the other high-risk regions. Efforts should also be made to increase awareness and promote education, change unhealthy lifestyles, reduce alcohol intake, and detect and treat alcohol-induced cirrhosis and other chronic liver diseases as early as possible.

## Electronic supplementary material

Below is the link to the electronic supplementary material.

Additional file 1. Supplementary figures.

Additional file 2. Supplementary tables.

## Data Availability

The datasets generated during and/or analyzed during the current study are available from the corresponding author on reasonable request. All data were collected from the Global Burden of Diseases database. The Global Burden of Diseases database is publicly available and considered.
