# Supplementary material for: Global, regional, and national burden of cirrhosis and other chronic liver diseases due to alcohol use, 1990–2019: a systematic analysis for the Global Burden of Disease study 2019
Source: BMC Gastroenterol. 2022 Nov 23;22:484. doi: 10.1186/s12876-022-02518-0 (PMC9685909; doi:10.1186/s12876-022-02518-0)
Supplement: Supplementary file 1 — Additional file 1. Supplementary figures. [file 12876_2022_2518_MOESM1_ESM.doc]

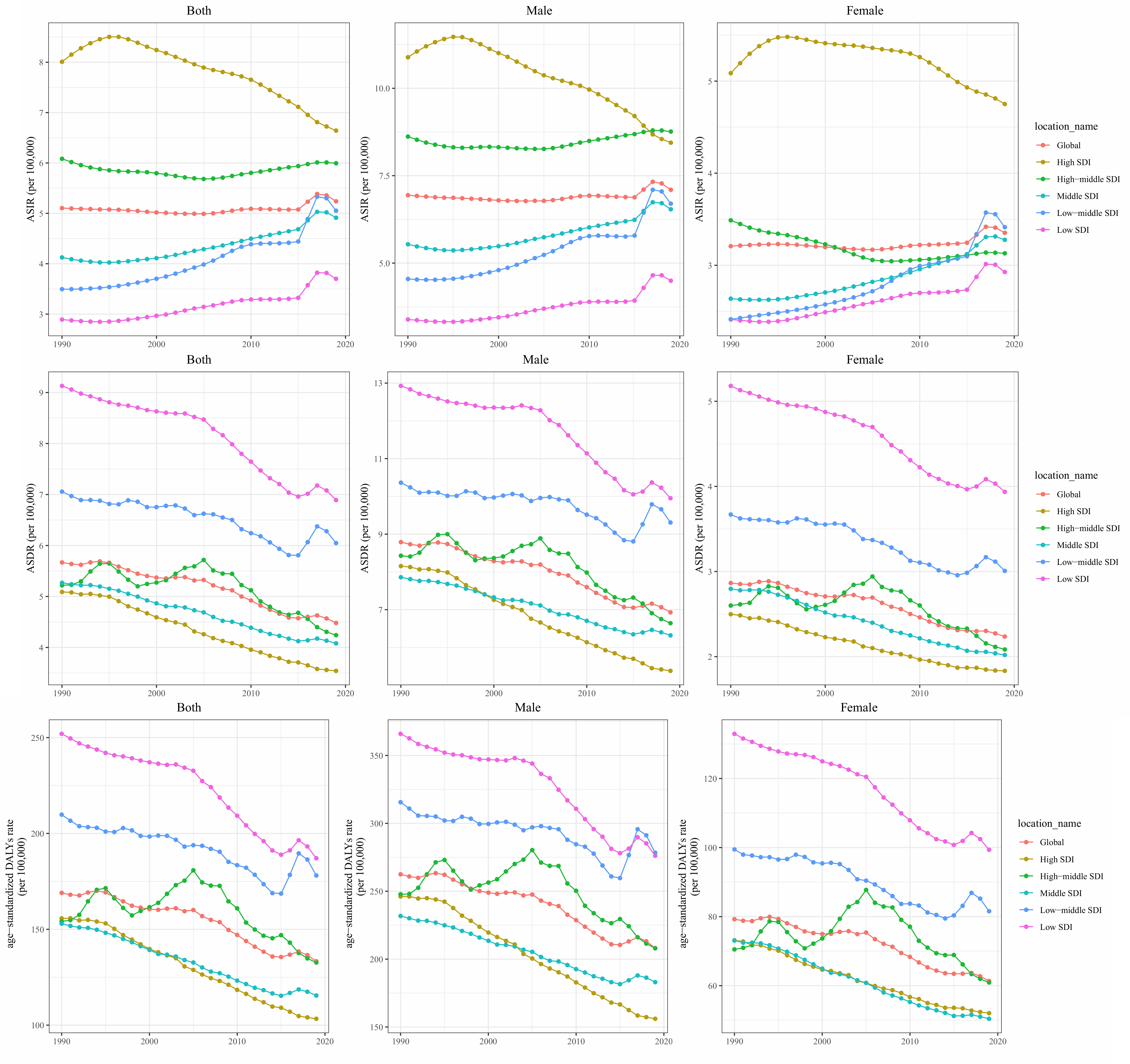


**Supplemental Fig 1: The change trends of age-standardized cirrhosis and other chronic liver diseases due to alcohol use incidence, death, and DALY rate among different SDI quintiles and gender from 1990 to 2019.** (A) ASIR, age standardized incidence rate. (B) ASDR, age standardized death rate. (C) age-standardized DALY rate. Abbreviations: DALY = disability adjusted life-year.


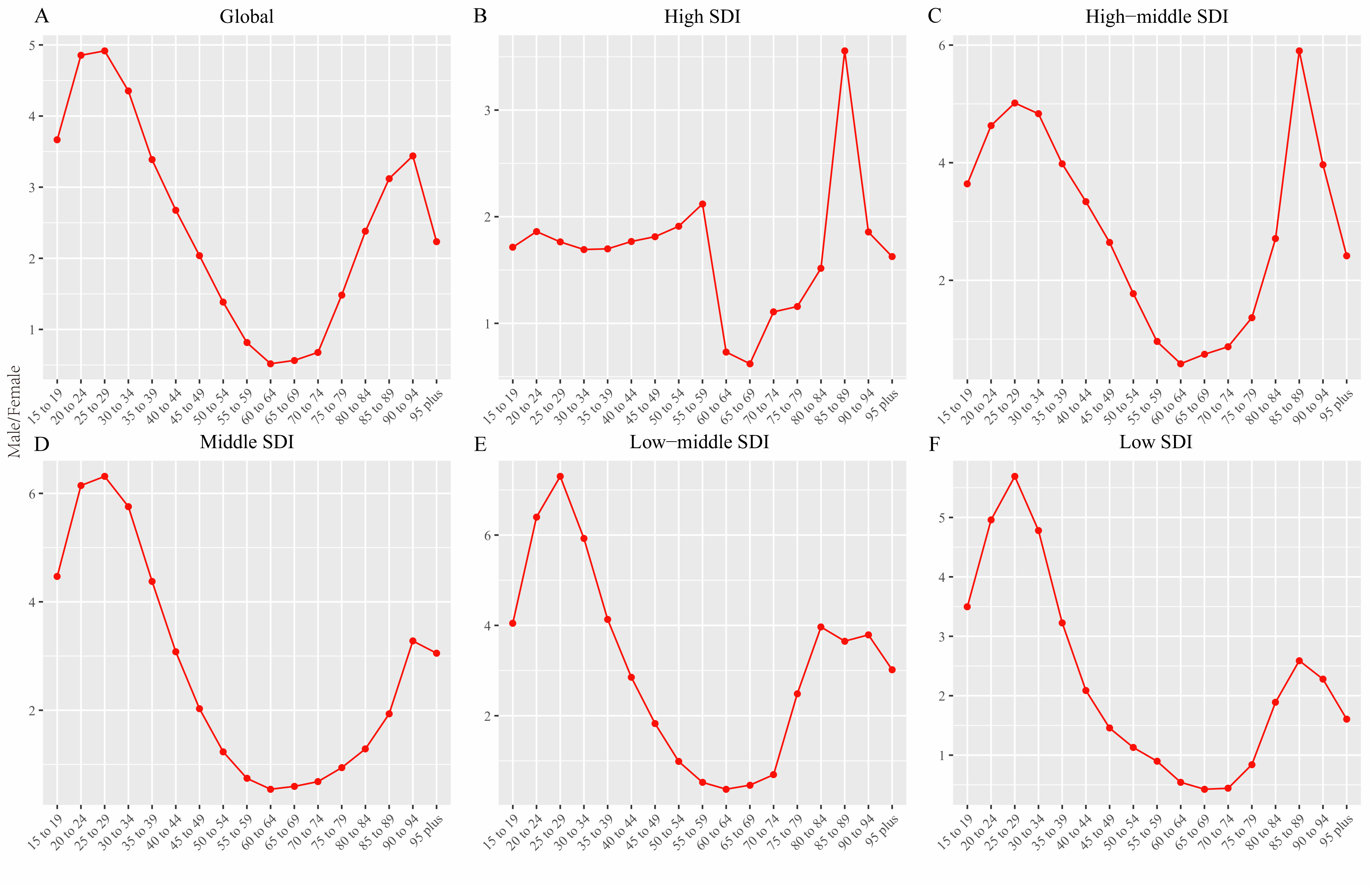


**Supplemental Fig 2: The ratio of male to female incidence among different age groups in 2019.** (A) Global. (B) High SDI. (C) High-middle SDI. (D) Middle SDI. (E) Middle-low SDI. (F) Low SDI. Abbreviations: SDI = socio-demographic index.

**
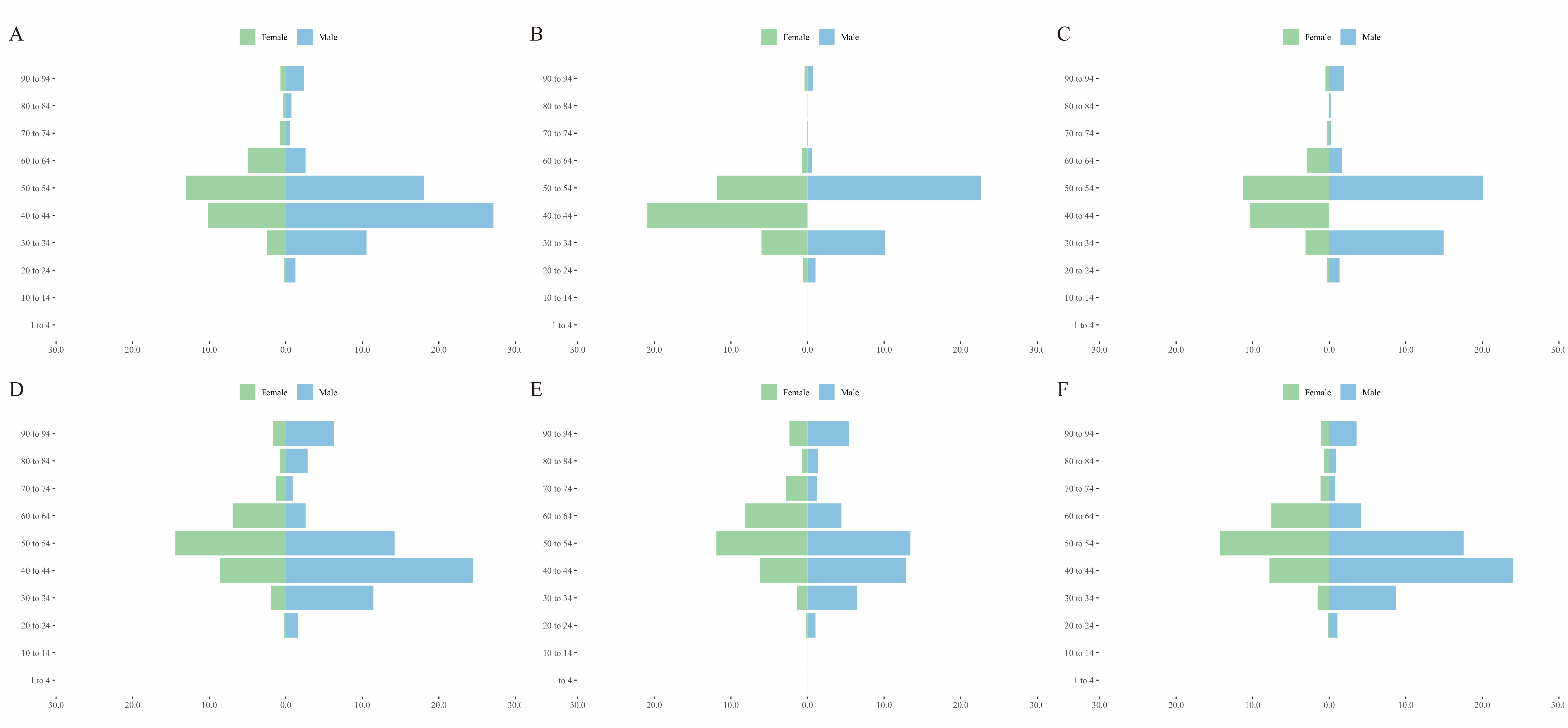
**

**Supplemental Fig 3. Distribution of different ages in cirrhosis and other chronic liver diseases due to alcohol use incidence in global** (A), high SDI (B), high-middle SDI (C), middle SDI (D), middle-low SDI (E), low SDI (F). Abbreviations: SDI, socio-demographic index.


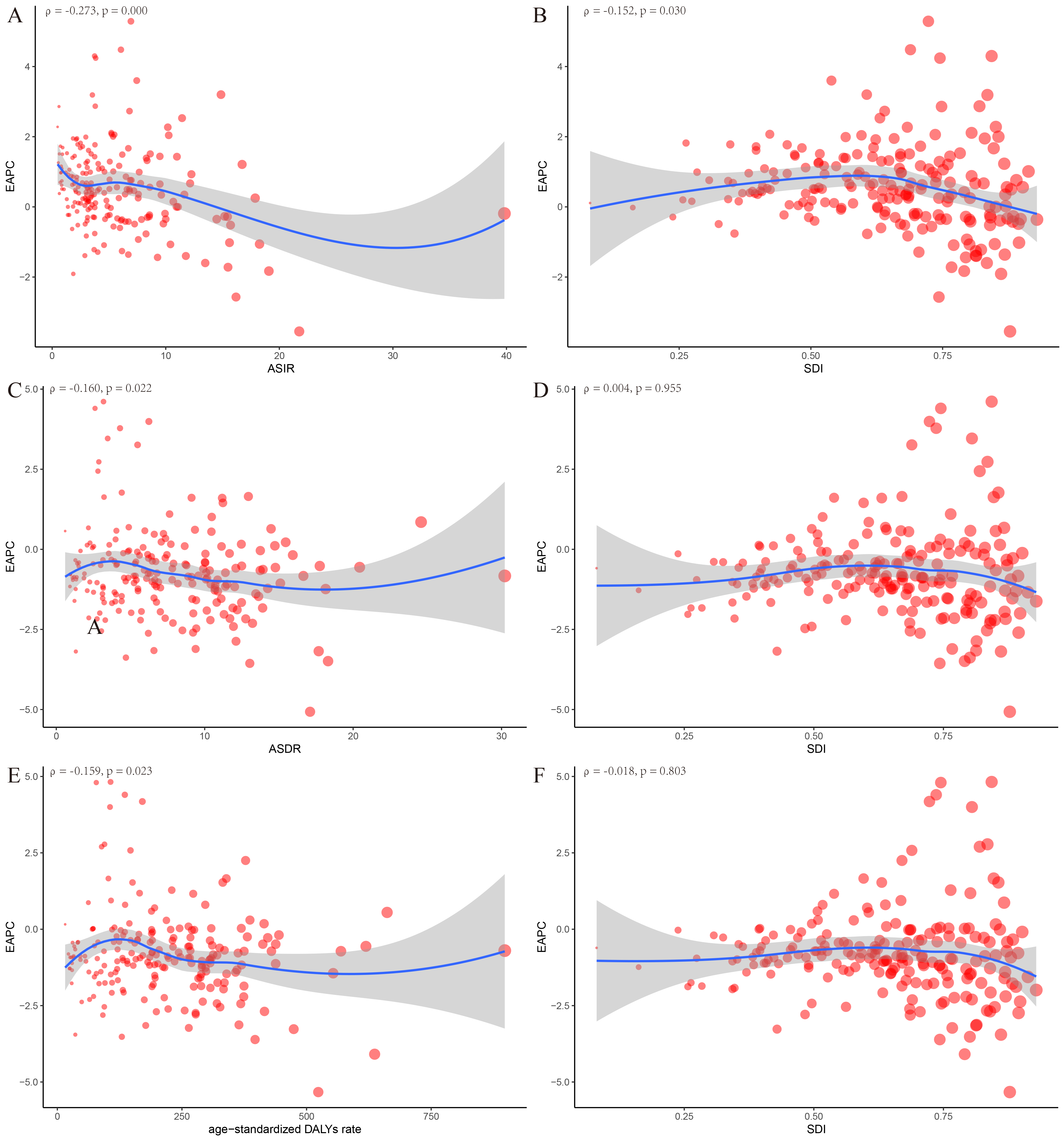


**Supplemental Fig 4: The correlation between EAPC and cirrhosis and other chronic liver diseases due to alcohol use age-standardized rates in 1990 and SDI in 2019. The circles represent countries that were available on SDI data. The size of circle is increased with the cases of cirrhosis and other chronic liver diseases due to alcohol use. The ρ indices Pearson’s correlation coefficient and p values were derived from Pearson’s correlation analysis.** (A) EAPC and ASIR. (B) EAPC and SDI in incidence. (C) EAPC and ASDR. (D) EAPC and SDI in death. (E) EAPC and age-standardized DALY rate. (F) EAPC and SDI in DALYs. Abbreviations: EAPC = estimated annual percentage change. SDI = socio-demographic index. ASIR = age standardized incidence rate. ASDR = age standardized death rate. DALY = disability adjusted life-year.


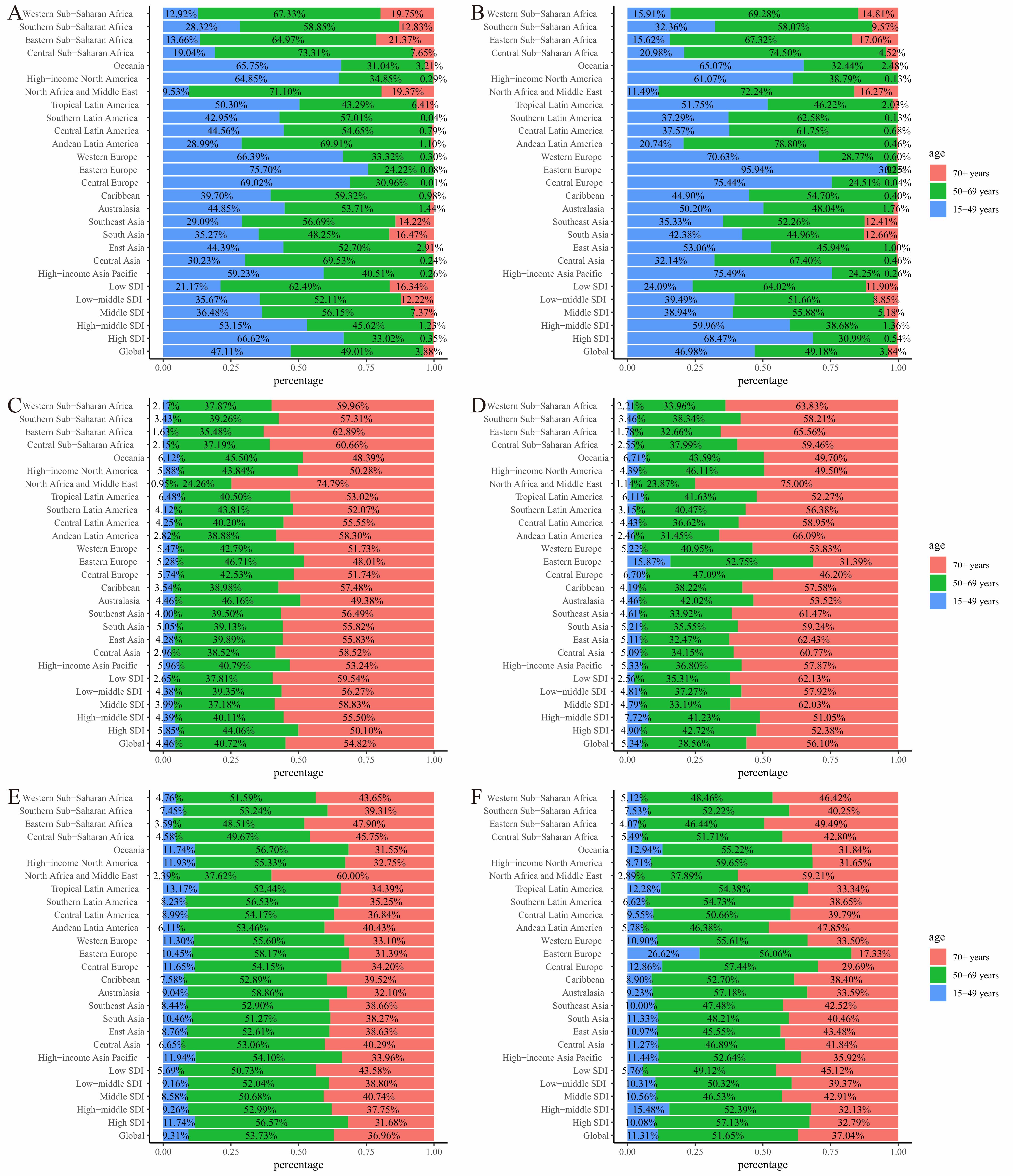


**Supplemental Fig 5: The incidence, death, and DALY rates of cirrhosis and other chronic liver diseases due to alcohol use in different age groups.** (A) incidence in 1990. (B) incidence in 2019. (C) Death rate in 1990. (D) Death rate in 2019. (E) DALY rate in 1990. (F) DALY rate in 2019. Abbreviations: DALY = disability adjusted life-year.


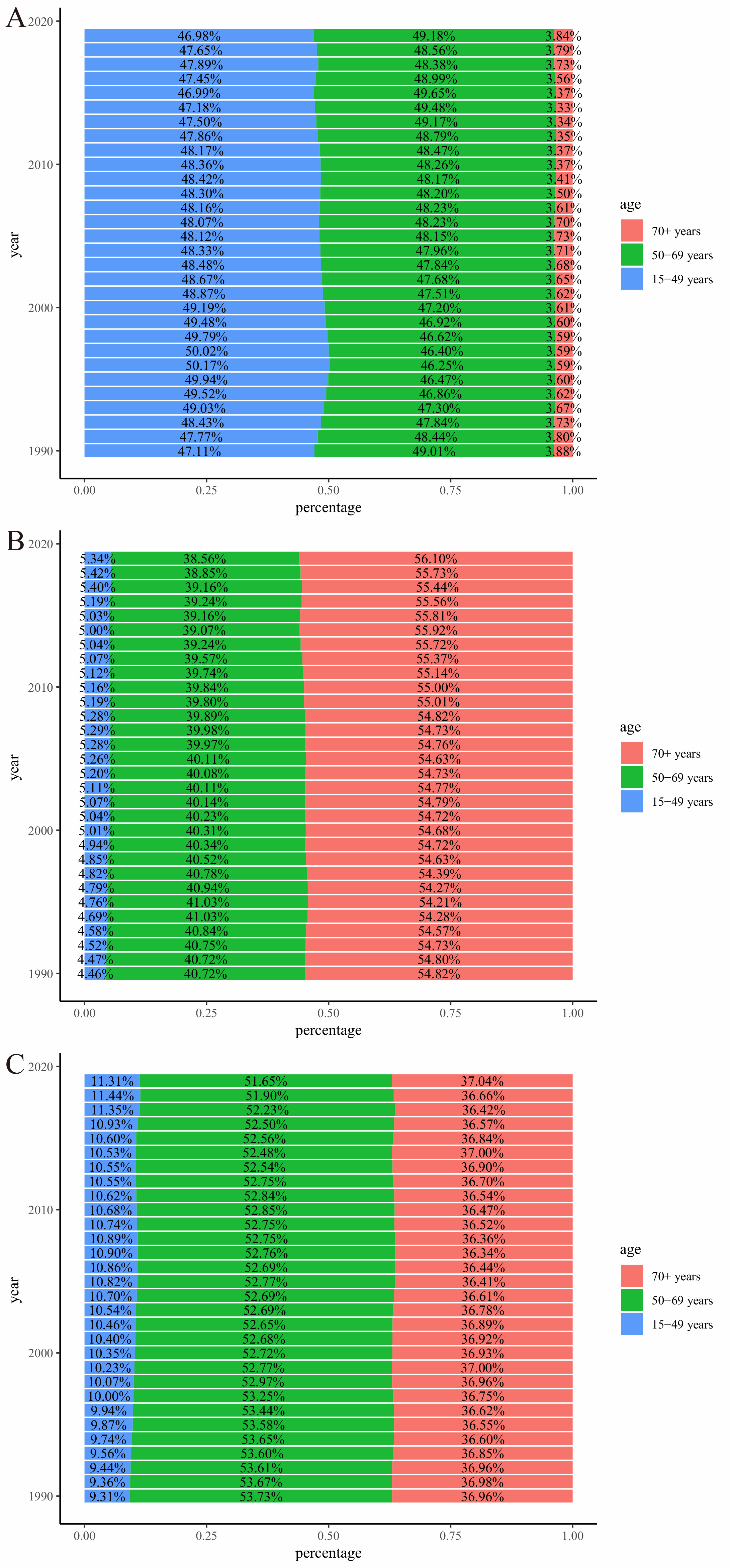


**Supplemental Fig 6: The proportion of different ages in cirrhosis and other chronic liver diseases due to alcohol use incidence** (A) and death (B) and age-standardized DALY (C) by years. Abbreviations: DALY = disability adjusted life-year.


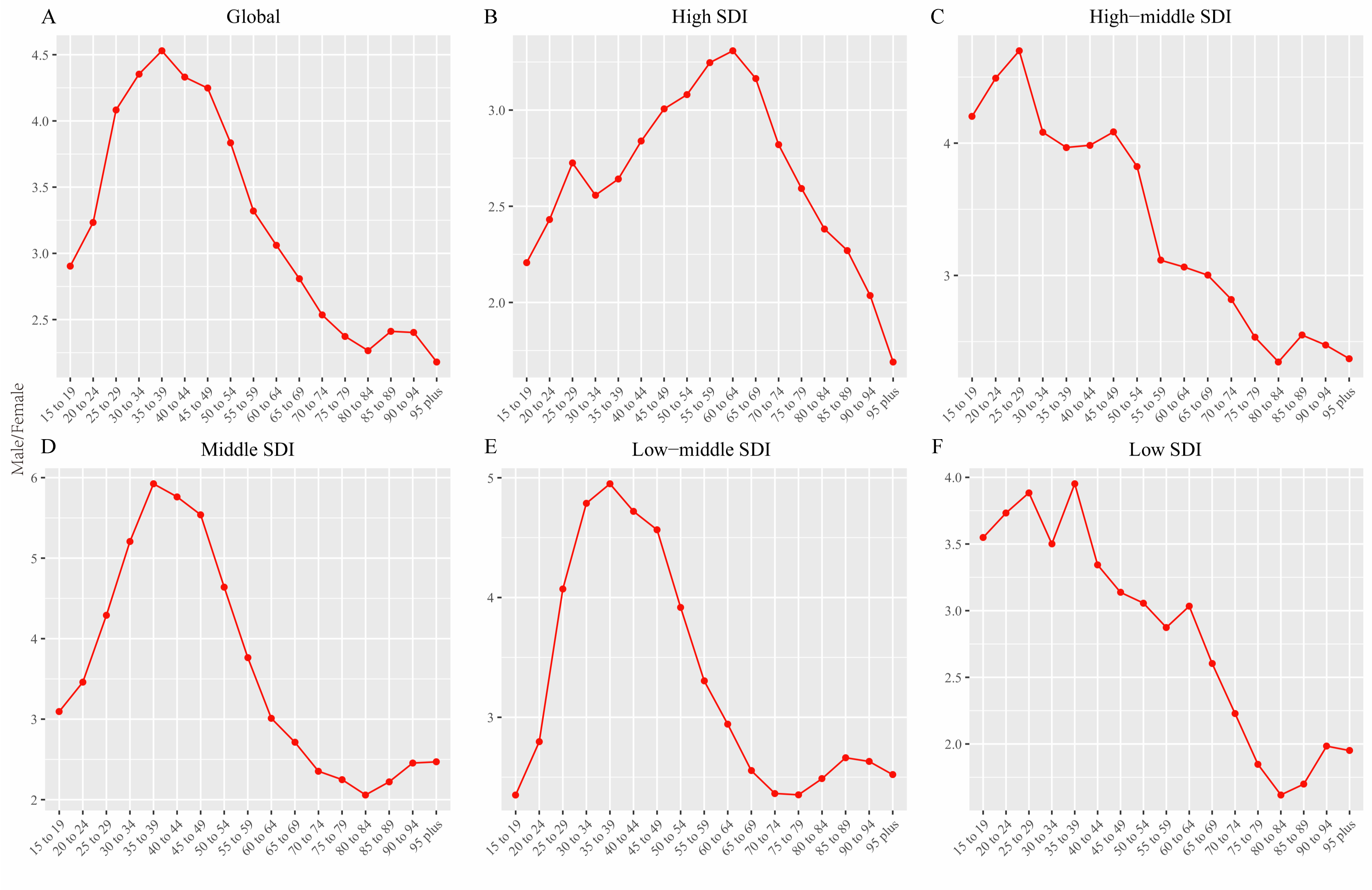


**Supplemental Fig 7: The ratio of male to female death among different age groups in 2019**. (A) Global. (B) High SDI. (C) High-middle SDI. (D) Middle SDI. (E) Middle-low SDI. (F) Low SDI. Abbreviations: SDI = socio-demographic index.


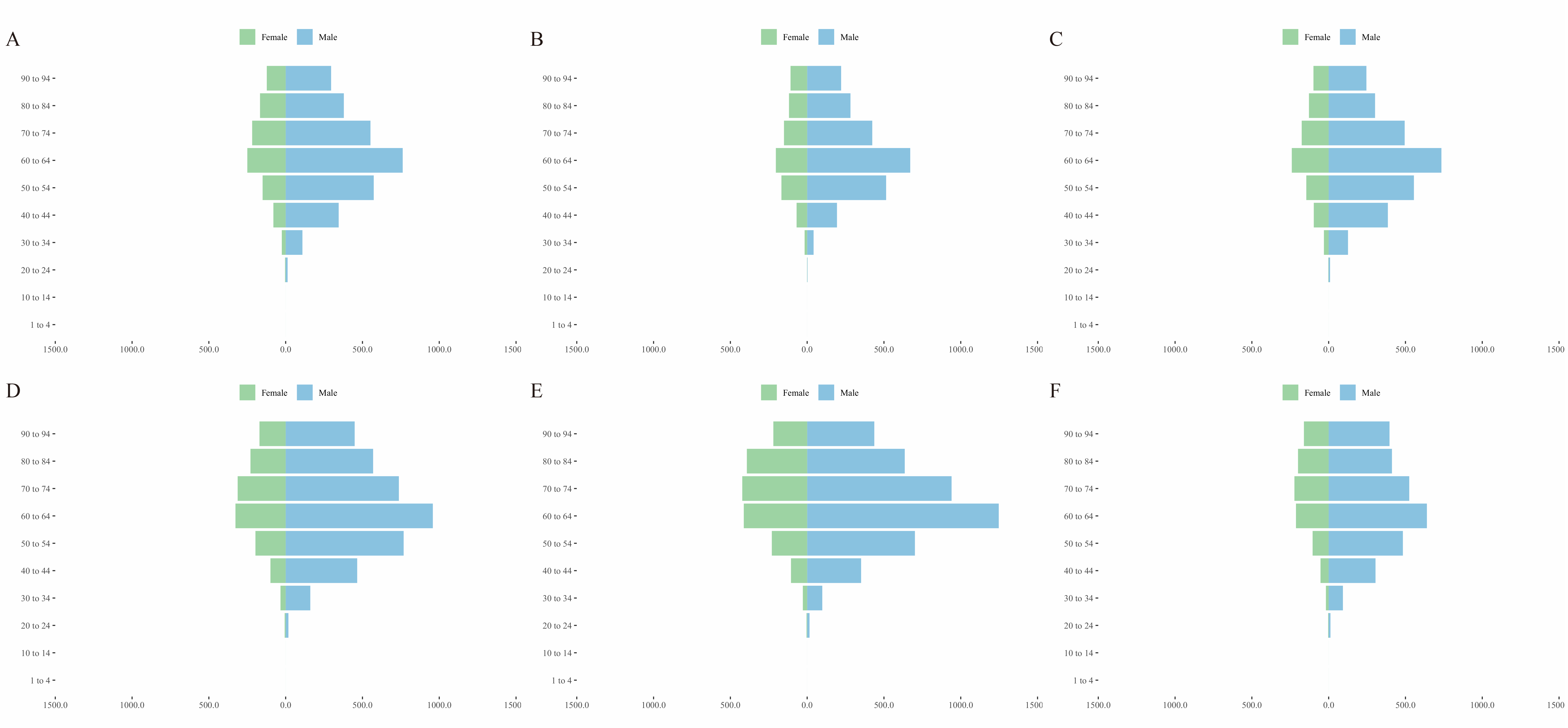


**Supplemental Fig 8. Distribution of different ages in cirrhosis and other chronic liver diseases due to alcohol use death in global** (A), high SDI (B), high-middle SDI (C), middle SDI (D), middle-low SDI (E), low SDI (F). Abbreviations: SDI, socio-demographic index.


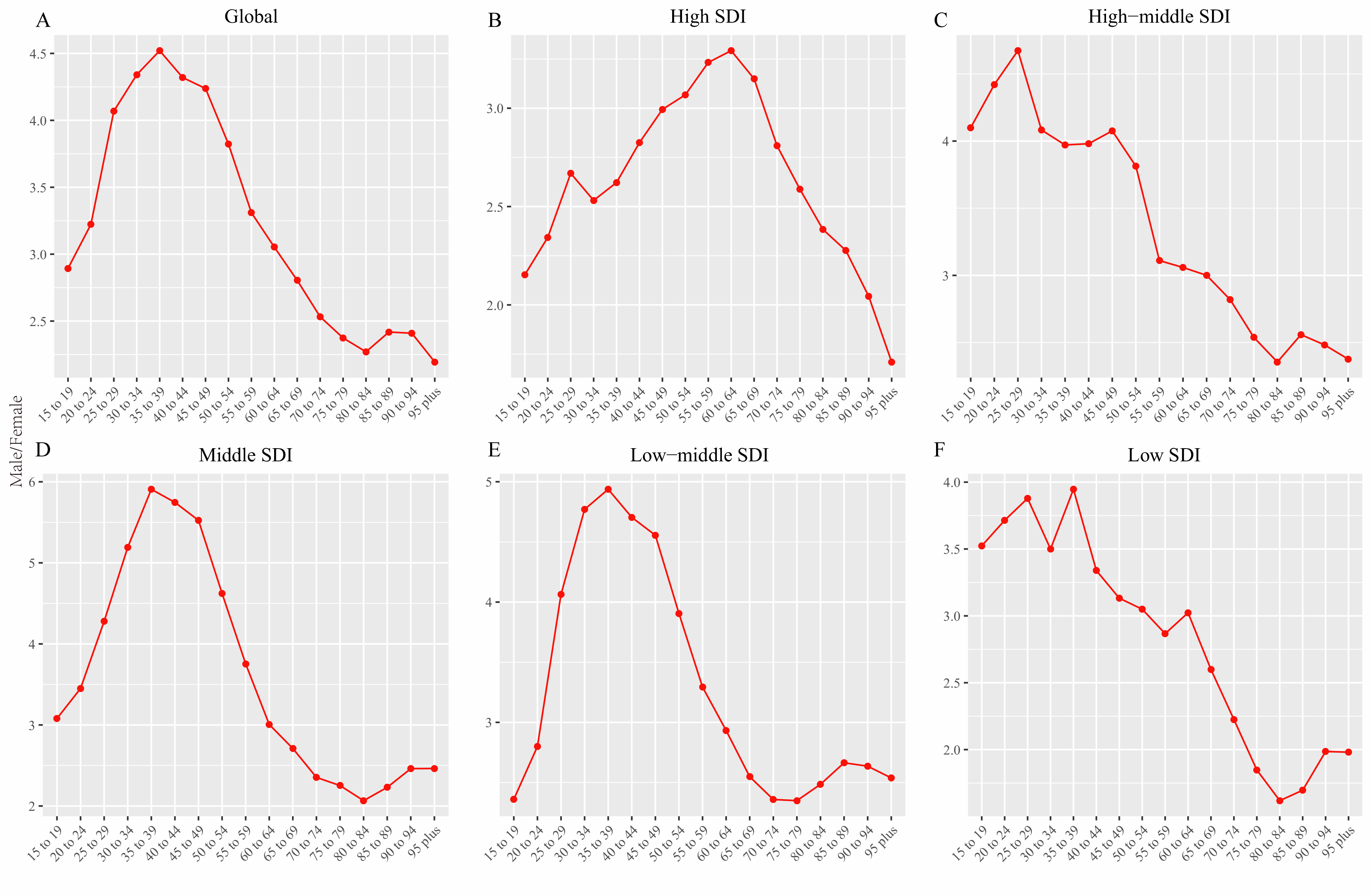


**Supplemental Fig 9: The ratio of male to female age standardized DALY rate among different age groups in 2019.** (A) Global. (B) High SDI. (C) High-middle SDI. (D) Middle SDI. (E) Middle-low SDI. (F) Low SDI. Abbreviations: SDI = socio-demographic index.

**
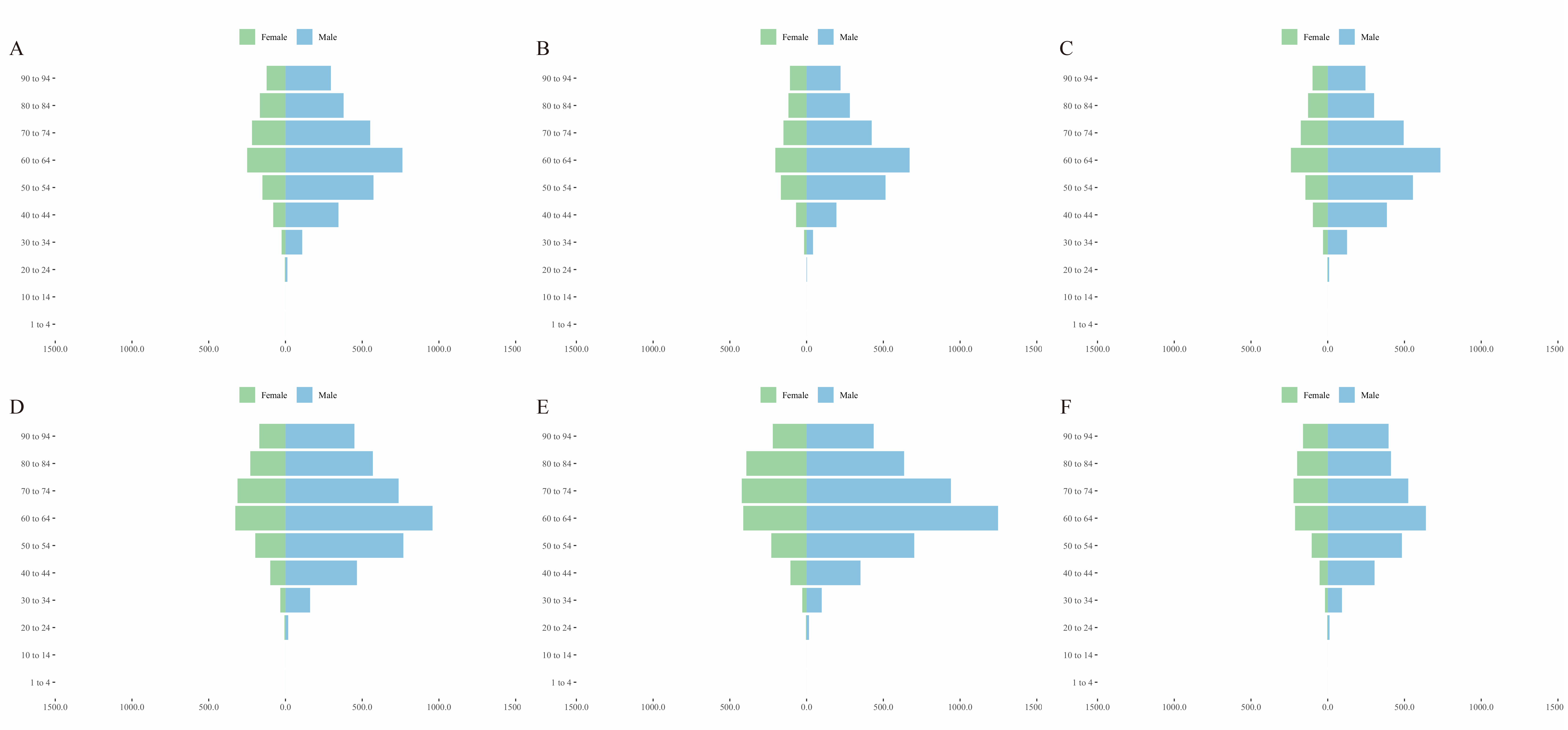
**

**Supplemental Fig 10. Distribution of different ages in cirrhosis and other chronic liver diseases due to alcohol use DALYs in global** (A), high SDI (B), high-middle SDI (C), middle SDI (D), middle-low SDI (E), low SDI (F). Abbreviations: Abbreviations: SDI, socio-demographic index.
