# Supplementary material for: Global, regional, and national burden of cirrhosis and other chronic liver diseases due to alcohol use, 1990–2019: a systematic analysis for the Global Burden of Disease study 2019
Source: BMC Gastroenterol. 2022 Nov 23;22:484. doi: 10.1186/s12876-022-02518-0 (PMC9685909; doi:10.1186/s12876-022-02518-0)
Supplement: Supplementary file 2 — Additional file 2. Supplementary tables. [file 12876_2022_2518_MOESM2_ESM.docx]

**Supplementary table 1. The top three and the bottom three countries of cirrhosis and other chronic liver diseases due to alcohol use incidence, death, or DALY.**

| **Measure** | **sex** | **Top three countries** | | | **Bottom three countries** | | |
| --- | --- | --- | --- | --- | --- | --- | --- |
| **2019 ASR (per 100,000 people)** | | | | | | | |
| **ASIR** |  |  |  |  |  |  |  |
|  | both | Kazakhstan(24.24) | Mongolia(30.70) | Republic of Moldova(35.64) | Papua New Guinea(0.70) | Sudan(0.83) | Kuwait(0.85) |
|  | female | Uzbekistan(17.96) | Republic of Moldova(25.56) | Mongolia(27.42) | Papua New Guinea(0.15) | Cook Islands(0.42) | Kuwait(0.58) |
|  | male | Kazakhstan(31.79) | Mongolia(33.66) | Republic of Moldova(45.22) | Pakistan(0.83) | Sudan(0.89) | Kuwait(1.06) |
| **ASDR** |  |  |  |  |  |  |  |
|  | both | Uzbekistan(21.05) | Republic of Moldova(23.42) | Mongolia(29.25) | Singapore(0.52) | Kuwait(0.61) | New Zealand(0.67) |
|  | female | Uzbekistan(15.21) | Republic of Moldova(16.47) | Mongolia(22.38) | Kuwait(0.27) | Singapore(0.32) | New Zealand(0.35) |
|  | male | Turkmenistan(30.80) | Republic of Moldova(32.25) | Mongolia(37.90) | Singapore(0.74) | Kuwait(0.85) | Iran (Islamic Republic of)(0.99) |
| **Age-standardized DALY rate** | | |  |  |  |  |  |
|  | both | Turkmenistan(698.61) | Republic of Moldova(716.53) | Mongolia(746.81) | Singapore(13.42) | Kuwait(14.43) | Iran (Islamic Republic of)(15.93) |
|  | female | Uzbekistan(428.38) | Republic of Moldova(483.86) | Mongolia(519.95) | Kuwait(6.00) | Singapore(7.64) | Iran (Islamic Republic of)(8.00) |
|  | male | Republic of Moldova(993.58) | Mongolia(1010.74) | Turkmenistan(1043.21) | Singapore(18.94) | Kuwait(20.36) | Iran (Islamic Republic of)(23.90) |
| **1990-2019 increase times** | | | | | | | |
| **Incidence (cases)** | | | | | | | |
|  | both | Bahrain(6.95) | Qatar(11.81) | United Arab Emirates(12.26) | Portugal(-0.37) | Hungary(-0.31) | Japan(-0.28) |
|  | female | Bahrain(5.39) | Qatar(12.99) | United Arab Emirates(11.79) | Portugal(-0.38) | Hungary(-0.38) | Japan(-0.32) |
|  | male | Bahrain(7.48) | Qatar(11.61) | United Arab Emirates(12.33) | Portugal(-0.37) | Northern Mariana Islands(-0.33) | Republic of Korea(-0.28) |
| **Deaths (cases)** | | | | | | | |
|  | both | Kuwait(3.03) | Qatar(4.85) | United Arab Emirates(7.18) | Portugal(-0.42) | Italy(-0.39) | Hungary(-0.36) |
|  | female | Nicaragua(3.28) | United Arab Emirates(3.95) | Qatar(4.12) | Portugal(-0.49) | Croatia(-0.44) | Bermuda(-0.41) |
|  | male | Belize(3.61) | Qatar(4.98) | United Arab Emirates(7.91) | Italy(-0.42) | Portugal(-0.40) | Hungary(-0.35) |
| **DALYs (cases)** | |  |  |  |  |  |  |
|  | both | Belize(3.50) | Qatar(4.85) | United Arab Emirates(7.67) | Italy(-0.47) | Portugal(-0.47) | Hungary(-0.45) |
|  | female | Uzbekistan(2.80) | Qatar(4.20) | United Arab Emirates(4.60) | Portugal(-0.56) | Croatia(-0.55) | Hungary(-0.50) |
|  | male | Belize(4.09) | Qatar(4.95) | United Arab Emirates(8.28) | Italy(-0.47) | Portugal(-0.44) | Hungary(-0.44) |
| **EAPC** | | | | | | | |
| **Incidence** |  |  |  |  |  |  |  |
|  | both | Lithuania(4.30) | Armenia(4.48) | Kazakhstan(5.29) | Republic of Korea(-3.55) | Portugal(-2.57) | Singapore(-1.91) |
|  | female | Belarus(3.95) | Armenia(4.17) | Kazakhstan(4.86) | Republic of Korea(-2.86) | Portugal(-2.79) | Hungary(-2.10) |
|  | male | Lithuania(4.49) | Armenia(4.66) | Kazakhstan(5.54) | Republic of Korea(-3.82) | Singapore(-2.50) | Portugal(-2.48) |
| **Deaths (cases)** | |  |  |  |  |  |  |
|  | both | Kazakhstan(3.99) | Belarus(4.40) | Lithuania(4.61) | Republic of Korea(-5.07) | Portugal(-3.56) | Hungary(-3.49) |
|  | female | Armenia(3.67) | Lithuania(4.19) | Belarus(4.43) | Bermuda(-5.07) | Republic of Korea(-4.76) | Portugal(-4.42) |
|  | male | Kazakhstan(4.15) | Belarus(4.20) | Lithuania(4.64) | Republic of Korea(-5.39) | Singapore(-3.78) | Italy(-3.49) |
| **DALY** |  |  |  |  |  |  |  |
|  | both | Ukraine(4.40) | Belarus(4.80) | Lithuania(4.82) | Republic of Korea(-5.33) | Hungary(-4.09) | Portugal(-3.61) |
|  | female | Russian Federation(4.22) | Lithuania(4.38) | Belarus(4.82) | Bermuda(-5.06) | Republic of Korea(-4.97) | Portugal(-4.64) |
|  | male | Kazakhstan(4.36) | Belarus(4.65) | Lithuania(4.88) | Republic of Korea(-5.54) | Singapore(-4.06) | Hungary(-4.01) |

**Supplementary table 2. The top three and the bottom three regions of cirrhosis and other chronic liver diseases due to alcohol use incidence, death, or DALY.**

| **Measure** | **sex** | **Top three regions** | | | | | **Bottom three regions** | | | |
| --- | --- | --- | --- | --- | --- | --- | --- | --- | --- | --- |
| **2019 ASR (per 100,000 people)** |  |  |  |  |  | | | |  |  |
| **ASIR** |  |  |  |  |  | | | |  |  |
|  | both | Andean Latin America(13.04) | Central Latin America(13.17) | Central Asia(21.15) | Oceania(1.04) | | | | North Africa and Middle East(1.54) | Australasia(1.68) |
|  | female | Andean Latin America(10.31) | Central Latin America(10.99) | Central Asia(16.09) | Oceania(0.38) | | | | Australasia(1.03) | North Africa and Middle East(1.16) |
|  | male | Andean Latin America(15.89) | Central Europe(17.17) | Central Asia(26.21) | Oceania(1.7) | | | | North Africa and Middle East(1.88) | Australasia(2.37) |
| **ASDR** |  |  |  |  |  | | | |  |  |
|  | both | Andean Latin America(10.19) | Central Latin America(10.31) | Central Asia(17.22) | Australasia(1.01) | | | | East Asia(1.59) | North Africa and Middle East(1.73) |
|  | female | Eastern Europe(5.62) | Andean Latin America(5.74) | Central Asia(11.23) | Australasia(0.47) | | | | East Asia(0.56) | Oceania(0.84) |
|  | male | Eastern Europe(15.69) | Central Latin America(16.65) | Central Asia(24.77) | Australasia(1.59) | | | | North Africa and Middle East(2.38) | East Asia(2.74) |
| **Age-standardized DALY rate** |  |  |  |  |  | | | |  |  |
|  | both | Central Latin America(297.28) | Eastern Europe(364.77) | Central Asia(505.54) | | | | Australasia(28.99) | North Africa and Middle East(39.03) | East Asia(44.8) |
|  | female | Andean Latin America(136.54) | Eastern Europe(196.69) | Central Asia(308.35) | East Asia(13.03) | | | | Australasia(13.49) | North Africa and Middle East(22.77) |
|  | male | Central Latin America(494.96) | Eastern Europe(563.24) | Central Asia(733.95) | Australasia(45.51) | | | | North Africa and Middle East(54.52) | East Asia(77.29) |
| **1990-2019 increase times** |  |  |  |  |  | | | |  |  |
| **Incidence (cases)** |  |  |  |  |  | | | |  |  |
|  | both | Andean Latin America(2.78) | North Africa and Middle East(2.97) | Central Asia(3.2) | High-income Asia Pacific(-0.24) | | | | Western Europe(0.03) | Central Europe(0.11) |
|  | female | North Africa and Middle East(2.63) | Central Asia(3.1) | Andean Latin America(3.4) | High-income Asia Pacific(-0.17) | | | | Central Europe(-0.02) | Western Europe(0.05) |
|  | male | Central Sub-Saharan Africa(2.93) | North Africa and Middle East(3.18) | Central Asia(3.27) | High-income Asia Pacific(-0.27) | | | | Western Europe(0.02) | High-income North America(0.04) |
| **Deaths (cases)** |  |  |  |  | |  | | |  |  |
|  | both | Andean Latin America(1.25) | Eastern Europe(1.72) | Central Asia(1.96) | | High-income Asia Pacific(-0.11) | | | Western Europe(-0.07) | East Asia(0.14) |
|  | female | Eastern Europe(1.36) | Andean Latin America(1.41) | Central Asia(1.65) | | Western Europe(-0.09) | | | East Asia(-0.02) | High-income Asia Pacific(0.09) |
|  | male | Andean Latin America(1.2) | Eastern Europe(1.93) | Central Asia(2.17) | High-income Asia Pacific(-0.17) | | | | Western Europe(-0.06) | East Asia(0.19) |
| **DALY (cases)** |  |  |  |  |  | | | |  |  |
|  | both | Oceania(1.09) | Eastern Europe(2.07) | Central Asia(2.2) | High-income Asia Pacific(-0.3) | | | | Western Europe(-0.15) | East Asia(0.01) |
|  | female | Andean Latin America(1.03) | Eastern Europe(1.69) | Central Asia(1.81) | High-income Asia Pacific(-0.22) | | | | Western Europe(-0.18) | East Asia(-0.16) |
|  | male | Oceania(1.11) | Eastern Europe(2.27) | Central Asia(2.43) | High-income Asia Pacific(-0.32) | | | | Western Europe(-0.14) | East Asia(0.05) |
| **EAPC** |  |  |  |  |  | | | |  |  |
| **Incidence** |  |  |  |  |  | | | |  |  |
|  | both | Andean Latin America(1.74) | Eastern Europe(2.01) | Central Asia(3.2) | High-income Asia Pacific(-1.73) | | | | Western Europe(-0.59) | High-income North America(-0.39) |
|  | female | Eastern Europe(1.4) | Andean Latin America(2.18) | Central Asia(3.04) | High-income Asia Pacific(-1.02) | | | | Tropical Latin America(-0.61) | Western Europe(-0.55) |
|  | male | South Asia(1.5) | Eastern Europe(2.22) | Central Asia(3.29) | High-income Asia Pacific(-2.06) | | | | High-income North America(-0.78) | Western Europe(-0.6) |
| **Deaths** |  |  |  |  |  | | | |  |  |
|  | both | High-income North America(0.09) | Central Asia(2) | Eastern Europe(3.27) | High-income Asia Pacific(-3.1) | | | | East Asia(-2.47) | Western Europe(-1.83) |
|  | female | High-income North America(0.53) | Central Asia(1.7) | Eastern Europe(3.11) | East Asia(-3.35) | | | | High-income Asia Pacific(-2.93) | Western Europe(-1.96) |
|  | male | South Asia(0.17) | Central Asia(2.07) | Eastern Europe(3.11) | High-income Asia Pacific(-3.26) | | | | East Asia(-2.16) | Western Europe(-1.91) |
| **DALY** |  |  |  |  |  | | | |  |  |
|  | both | High-income North America(-0.04) | Central Asia(2.05) | Eastern Europe(3.82) | High-income Asia Pacific(-3.34) | | | | East Asia(-2.71) | Western Europe(-1.98) |
|  | female | High-income North America(0.56) | Central Asia(1.61) | Eastern Europe(3.71) | East Asia(-3.74) | | | | High-income Asia Pacific(-3.01) | Western Europe(-2.14) |
|  | male | South Asia(-0.05) | Central Asia(2.2) | Eastern Europe(3.71) | High-income Asia Pacific(-3.48) | | | | East Asia(-2.41) | Western Europe(-1.99) |

**Supplementary table 3. The incidence cases and age-standardized incidence rate of cirrhosis and other chronic liver diseases due to alcohol use in 1990 and 2019, and its temporal trends from 1990 to 2019.**

| **Nation** | **Sex** | **Incident Cases No. (95% UI)** | | **1990-2019 EAPC of numbers** | **ASIR per 100,000 No.(95% UI)** | | **1990-2019 EAPC of ASIR** |
| --- | --- | --- | --- | --- | --- | --- | --- |
|  |  | **1990** | **2019** |  | **1990** | **2019** |  |
| Afghanistan | both | 67.68(116.24,34.84) | 227.32(388.34,122.94) | 4.12(3.60,4.65) | 0.85(1.44,0.45) | 1.12(1.78,0.63) | 0.74(0.54,0.93) |
| Albania | both | 200.95(263.89,141.57) | 245.56(315.16,180.03) | 1.01(0.80,1.22) | 7.60(9.92,5.33) | 7.91(10.09,5.84) | 0.36(0.18,0.55) |
| Algeria | both | 118.85(190.62,67.01) | 445.72(708.31,263.99) | 4.39(4.10,4.68) | 0.84(1.34,0.47) | 1.10(1.73,0.65) | 0.62(0.34,0.89) |
| American Samoa | both | 0.66(1.03,0.37) | 0.82(1.30,0.49) | 0.36(0.05,0.67) | 1.76(2.78,1.01) | 1.56(2.44,0.93) | -0.63(-0.73,-0.54) |
| Andorra | both | 6.27(7.98,4.58) | 10.60(13.76,7.77) | 1.57(1.12,2.03) | 9.79(12.41,7.09) | 8.71(11.06,6.36) | -0.48(-0.59,-0.37) |
| Angola | both | 119.32(196.03,63.91) | 551.90(872.34,323.65) | 5.43(5.06,5.81) | 1.98(3.24,1.00) | 3.05(4.91,1.77) | 1.45(1.15,1.76) |
| Antigua and Barbuda | both | 2.89(3.89,1.97) | 7.23(9.73,5.03) | 3.29(3.05,3.52) | 5.87(7.91,4.01) | 6.40(8.52,4.50) | 0.25(0.14,0.36) |
| Argentina | both | 1855.91(2617.75,1213.37) | 3930.44(5511.01,2592.31) | 2.65(2.50,2.80) | 5.90(8.33,3.84) | 8.15(11.43,5.34) | 1.17(1.05,1.28) |
| Armenia | both | 192.05(253.41,137.56) | 663.50(844.61,487.57) | 5.19(4.92,5.47) | 6.05(7.96,4.34) | 17.74(22.51,12.99) | 4.48(4.24,4.72) |
| Australia | both | 333.45(490.46,207.32) | 497.65(749.54,303.35) | 1.54(1.36,1.72) | 1.84(2.72,1.14) | 1.74(2.61,1.07) | 0.01(-0.21,0.24) |
| Austria | both | 1532.90(1899.65,1140.30) | 1410.96(1733.59,1072.88) | -0.11(-0.40,0.18) | 18.26(22.65,13.52) | 13.39(16.45,10.16) | -1.06(-1.20,-0.93) |
| Azerbaijan | both | 611.71(819.97,438.94) | 2464.50(3301.15,1736.94) | 5.41(5.21,5.61) | 10.18(13.60,7.25) | 19.08(25.33,13.46) | 2.27(2.14,2.39) |
| Bahrain | both | 4.48(7.25,2.64) | 35.60(57.24,20.81) | 8.36(7.95,8.77) | 1.52(2.41,0.89) | 1.91(2.94,1.16) | 1.06(0.80,1.33) |
| Bangladesh | both | 1772.95(2843.64,1054.10) | 6045.98(8968.98,3805.67) | 4.45(4.33,4.58) | 2.48(3.93,1.45) | 3.91(5.79,2.45) | 1.68(1.55,1.82) |
| Barbados | both | 11.82(15.72,8.11) | 18.07(24.13,13.00) | 1.35(1.19,1.51) | 5.06(6.77,3.48) | 4.98(6.57,3.58) | -0.23(-0.31,-0.15) |
| Belarus | both | 411.83(559.01,275.95) | 1008.62(1325.13,696.46) | 3.89(3.41,4.38) | 3.83(5.22,2.55) | 9.50(12.42,6.64) | 4.24(3.72,4.76) |
| Belgium | both | 1190.32(1446.62,943.16) | 1406.48(1742.81,1065.58) | 0.59(0.36,0.83) | 10.95(13.29,8.57) | 10.98(13.59,8.34) | 0.00(-0.13,0.13) |
| Belize | both | 7.04(9.57,4.88) | 33.10(44.88,23.01) | 5.58(5.39,5.77) | 6.49(8.84,4.45) | 8.57(11.56,5.97) | 0.86(0.75,0.96) |
| Benin | both | 73.96(118.12,38.93) | 293.42(442.96,179.42) | 4.59(4.12,5.07) | 3.02(4.88,1.52) | 3.99(6.08,2.42) | 0.68(0.29,1.07) |
| Bermuda | both | 4.25(5.57,3.00) | 4.06(5.36,2.87) | -0.45(-0.62,-0.27) | 6.15(8.07,4.33) | 4.69(6.12,3.34) | -1.26(-1.41,-1.11) |
| Bhutan | both | 15.79(23.85,9.69) | 44.06(65.46,27.63) | 4.19(3.90,4.48) | 3.77(5.89,2.24) | 5.73(8.59,3.55) | 1.77(1.56,1.99) |
| Bolivia (Plurinational State of) | both | 354.75(498.87,228.63) | 1298.70(1757.95,926.42) | 4.58(4.46,4.70) | 8.29(11.89,5.29) | 12.46(16.85,8.83) | 1.39(1.32,1.47) |
| Bosnia and Herzegovina | both | 411.89(529.36,293.16) | 322.38(413.38,235.03) | -0.63(-0.75,-0.51) | 8.54(11.15,6.01) | 7.88(10.02,5.70) | -0.28(-0.45,-0.12) |
| Botswana | both | 25.64(41.15,14.70) | 93.39(143.66,55.47) | 4.71(4.51,4.91) | 3.11(5.09,1.70) | 4.05(6.31,2.35) | 1.30(1.10,1.49) |
| Brazil | both | 11022.07(15306.32,7346.56) | 20885.77(28756.00,14232.78) | 2.07(1.94,2.20) | 8.57(12.14,5.58) | 8.30(11.39,5.67) | -0.23(-0.30,-0.17) |
| Brunei Darussalam | both | 8.23(12.41,5.09) | 16.87(24.78,10.55) | 1.74(1.38,2.09) | 3.69(5.50,2.28) | 3.07(4.49,1.93) | -1.22(-1.41,-1.04) |
| Bulgaria | both | 921.08(1183.83,668.94) | 1077.44(1364.57,784.67) | 0.82(0.69,0.95) | 9.48(12.26,6.92) | 13.21(16.69,9.60) | 1.44(1.30,1.58) |
| Burkina Faso | both | 158.11(246.06,94.24) | 461.08(720.40,258.41) | 3.53(3.24,3.82) | 3.00(4.69,1.76) | 3.50(5.46,1.93) | 0.20(-0.02,0.42) |
| Burundi | both | 103.09(161.95,59.86) | 294.74(450.63,173.64) | 3.95(3.55,4.36) | 3.36(5.40,1.88) | 4.28(6.72,2.37) | 0.99(0.79,1.18) |
| Cambodia | both | 396.98(629.98,225.54) | 1461.85(2258.40,901.36) | 4.61(4.39,4.83) | 6.17(10.00,3.47) | 9.50(14.74,5.75) | 1.49(1.24,1.74) |
| Cameroon | both | 212.22(337.20,123.42) | 733.07(1109.79,435.78) | 4.38(4.20,4.56) | 3.49(5.57,1.96) | 3.96(6.04,2.29) | 0.38(0.27,0.49) |
| Canada | both | 1728.89(2442.79,1156.08) | 2218.32(3147.30,1419.47) | 0.83(0.53,1.14) | 5.74(8.11,3.80) | 5.26(7.50,3.43) | -0.33(-0.38,-0.28) |
| Cabo Verde | both | 4.69(7.44,2.68) | 18.86(28.13,11.74) | 5.02(4.83,5.20) | 2.37(3.78,1.29) | 3.46(5.13,2.14) | 1.17(0.95,1.38) |
| Central African Republic | both | 56.79(95.78,28.27) | 133.20(221.54,68.72) | 2.77(2.65,2.89) | 3.21(5.57,1.50) | 3.60(6.12,1.78) | 0.16(0.03,0.30) |
| Chad | both | 118.34(188.57,64.15) | 296.35(465.59,173.71) | 3.02(2.94,3.10) | 3.62(5.85,1.91) | 3.56(5.65,2.07) | -0.29(-0.37,-0.21) |
| Chile | both | 1269.00(1819.11,820.50) | 2617.32(3665.62,1741.08) | 2.45(2.21,2.69) | 10.67(15.28,6.80) | 12.02(16.81,8.12) | 0.15(0.01,0.29) |
| China | both | 38110.69(55785.45,23453.35) | 69310.00(97801.04,46617.48) | 2.39(2.31,2.47) | 3.47(5.12,2.08) | 3.59(4.97,2.47) | 0.31(0.10,0.52) |
| Colombia | both | 1473.11(1948.54,1046.32) | 2697.62(3566.59,1922.78) | 2.06(1.90,2.23) | 5.97(7.92,4.19) | 5.15(6.83,3.68) | -0.60(-0.66,-0.54) |
| Comoros | both | 8.24(13.34,4.57) | 23.86(36.74,13.78) | 3.79(3.61,3.97) | 2.99(4.92,1.62) | 3.90(6.04,2.24) | 0.95(0.79,1.11) |
| Congo | both | 31.81(51.76,17.53) | 119.88(188.60,69.48) | 4.64(4.35,4.94) | 2.18(3.64,1.14) | 2.87(4.45,1.67) | 0.85(0.64,1.05) |
| Costa Rica | both | 228.08(313.65,158.16) | 555.79(743.76,397.77) | 3.09(2.83,3.34) | 9.61(12.97,6.69) | 10.59(14.16,7.51) | 0.34(0.30,0.38) |
| Côte d'Ivoire | both | 220.07(343.40,124.85) | 735.93(1132.62,444.84) | 4.04(3.75,4.33) | 3.34(5.30,1.85) | 4.14(6.48,2.47) | 0.59(0.31,0.88) |
| Croatia | both | 850.27(1100.40,612.24) | 679.03(849.10,509.60) | -0.71(-0.87,-0.56) | 15.44(20.03,11.10) | 13.65(17.04,10.17) | -0.28(-0.43,-0.13) |
| Cuba | both | 463.61(611.06,333.09) | 859.48(1152.05,591.79) | 2.45(2.19,2.70) | 4.40(5.79,3.15) | 5.99(7.85,4.23) | 1.10(1.06,1.15) |
| Cyprus | both | 66.13(87.57,47.18) | 130.74(166.63,96.42) | 2.25(2.06,2.44) | 8.38(11.11,6.00) | 7.99(10.13,5.90) | -0.30(-0.45,-0.15) |
| Czechia | both | 919.41(1193.40,654.54) | 1178.92(1488.86,869.41) | 1.05(0.80,1.31) | 7.79(9.98,5.68) | 9.04(11.30,6.72) | 0.67(0.54,0.80) |
| Democratic Republic of the Congo | both | 604.73(988.13,331.16) | 2137.04(3362.46,1264.75) | 4.29(3.99,4.58) | 2.69(4.48,1.40) | 3.77(6.09,2.13) | 0.96(0.72,1.19) |
| Denmark | both | 403.99(519.86,288.65) | 519.63(667.65,365.56) | 1.09(0.56,1.63) | 6.97(8.91,4.94) | 8.44(10.93,6.02) | 0.76(0.28,1.25) |
| Djibouti | both | 9.38(14.57,5.48) | 38.36(59.40,23.38) | 4.92(4.74,5.11) | 3.52(5.55,2.05) | 3.75(5.92,2.26) | 0.06(-0.12,0.23) |
| Dominica | both | 2.97(3.98,2.05) | 3.94(5.41,2.73) | 0.67(0.49,0.85) | 5.40(7.31,3.71) | 5.17(6.94,3.55) | -0.56(-0.71,-0.41) |
| Dominican Republic | both | 374.31(504.73,262.62) | 1156.29(1568.80,805.24) | 3.76(3.67,3.86) | 7.66(10.41,5.30) | 10.73(14.65,7.44) | 0.99(0.89,1.09) |
| Ecuador | both | 479.77(641.68,343.56) | 2561.65(3224.54,1911.54) | 5.79(5.50,6.08) | 6.81(9.12,4.80) | 15.53(19.60,11.61) | 2.73(2.46,2.99) |
| Egypt | both | 773.74(1231.02,449.62) | 2956.81(4734.19,1716.93) | 4.88(4.73,5.03) | 2.18(3.52,1.25) | 3.68(5.86,2.12) | 1.95(1.81,2.09) |
| El Salvador | both | 355.88(490.51,243.91) | 681.41(922.59,473.56) | 2.24(2.14,2.35) | 9.49(13.14,6.51) | 11.43(15.46,7.94) | 0.65(0.53,0.78) |
| Equatorial Guinea | both | 7.73(13.39,3.76) | 24.85(38.36,15.13) | 4.21(3.93,4.50) | 2.90(5.06,1.36) | 3.07(4.83,1.81) | 0.25(0.14,0.37) |
| Eritrea | both | 55.16(89.49,31.46) | 231.48(360.96,137.04) | 5.12(4.89,5.34) | 3.20(5.16,1.81) | 5.05(7.83,2.98) | 1.61(1.33,1.90) |
| Estonia | both | 58.25(80.74,37.98) | 113.74(148.18,77.94) | 2.53(2.20,2.86) | 3.54(4.91,2.31) | 8.24(10.71,5.68) | 3.19(2.78,3.61) |
| Ethiopia | both | 847.75(1506.31,333.87) | 1925.76(3054.11,969.96) | 2.71(2.53,2.88) | 3.16(5.78,1.11) | 3.35(5.66,1.57) | 0.22(0.11,0.34) |
| Micronesia (Federated States of) | both | 1.78(2.98,1.00) | 2.54(4.10,1.44) | 1.01(0.84,1.18) | 2.49(4.10,1.38) | 2.65(4.25,1.50) | 0.10(0.02,0.18) |
| Fiji | both | 8.28(13.07,4.80) | 13.24(20.69,7.91) | 1.58(1.38,1.78) | 1.29(2.03,0.75) | 1.40(2.18,0.84) | 0.22(0.14,0.30) |
| Finland | both | 322.51(421.06,225.30) | 455.59(589.53,325.26) | 1.48(1.07,1.88) | 5.36(6.94,3.80) | 8.42(10.86,6.04) | 2.00(1.56,2.44) |
| France | both | 8329.33(10532.85,6229.36) | 6436.74(8105.58,4752.49) | -1.19(-1.35,-1.03) | 13.48(16.94,10.10) | 9.07(11.50,6.71) | -1.60(-1.68,-1.52) |
| Gabon | both | 13.22(20.95,7.22) | 35.31(55.03,21.14) | 3.41(3.14,3.68) | 2.04(3.23,1.09) | 2.41(3.74,1.44) | 0.48(0.27,0.68) |
| Georgia | both | 578.27(762.41,416.30) | 492.70(641.84,351.84) | -0.56(-0.65,-0.48) | 9.94(13.08,7.06) | 12.03(15.70,8.42) | -0.19(-2.59,2.28) |
| Germany | both | 12951.16(16175.25,9647.21) | 12149.84(15164.64,9049.48) | -0.18(-0.55,0.19) | 14.49(18.17,10.74) | 13.09(16.34,9.81) | -0.35(-0.52,-0.18) |
| Ghana | both | 278.26(443.74,151.77) | 783.63(1170.17,465.12) | 3.34(3.12,3.55) | 3.16(5.13,1.71) | 3.34(5.04,1.98) | -0.08(-0.27,0.12) |
| Greece | both | 1101.40(1398.02,799.97) | 809.66(1034.56,589.13) | -1.16(-1.28,-1.04) | 9.28(11.80,6.77) | 6.46(8.27,4.70) | -1.44(-1.57,-1.31) |
| Greenland | both | 2.13(3.13,1.34) | 2.06(3.03,1.29) | 0.15(-0.23,0.54) | 3.37(4.96,2.14) | 3.53(5.16,2.17) | 0.26(0.22,0.31) |
| Grenada | both | 3.95(5.31,2.76) | 9.70(13.01,6.93) | 3.00(2.60,3.41) | 6.84(9.28,4.70) | 8.23(10.92,5.85) | 0.45(0.38,0.53) |
| Guam | both | 3.68(5.82,2.20) | 3.62(5.79,2.15) | -0.56(-0.87,-0.24) | 2.88(4.48,1.72) | 2.14(3.35,1.27) | -1.39(-1.51,-1.28) |
| Guatemala | both | 672.55(942.69,450.05) | 2288.76(3130.77,1564.21) | 4.47(4.34,4.59) | 12.26(17.36,8.28) | 15.42(20.85,10.65) | 0.93(0.84,1.01) |
| Guinea | both | 109.67(179.13,56.43) | 213.24(330.12,125.43) | 1.67(1.35,2.00) | 2.90(4.87,1.44) | 2.91(4.50,1.73) | -0.49(-0.76,-0.22) |
| Guinea-Bissau | both | 20.51(33.19,11.22) | 41.56(65.55,24.73) | 1.88(1.68,2.07) | 3.75(6.26,1.92) | 3.50(5.55,2.02) | -0.76(-0.90,-0.63) |
| Guyana | both | 67.15(91.21,44.59) | 97.97(131.09,66.37) | 1.31(1.24,1.39) | 11.64(15.94,7.69) | 12.71(16.92,8.60) | 0.35(0.21,0.49) |
| Haiti | both | 308.38(442.63,195.21) | 900.20(1269.33,590.38) | 3.68(3.61,3.75) | 6.87(9.99,4.31) | 8.63(12.20,5.69) | 0.76(0.70,0.81) |
| Honduras | both | 322.29(453.25,213.87) | 901.73(1279.18,601.21) | 3.60(3.49,3.71) | 11.07(15.56,7.38) | 11.17(15.69,7.42) | 0.00(-0.10,0.11) |
| Hungary | both | 2188.57(2849.81,1490.93) | 1520.39(1883.63,1187.08) | -1.53(-1.73,-1.34) | 19.08(25.04,13.08) | 12.45(15.30,9.72) | -1.83(-2.20,-1.47) |
| Iceland | both | 7.77(10.36,5.45) | 12.17(15.80,8.60) | 1.55(1.32,1.78) | 3.04(4.01,2.14) | 3.28(4.32,2.28) | 0.35(0.27,0.43) |
| India | both | 20002.67(32085.43,9205.96) | 68961.06(104885.51,37913.26) | 4.65(4.35,4.94) | 2.96(4.83,1.31) | 4.91(7.56,2.68) | 1.99(1.71,2.27) |
| Indonesia | both | 5016.54(8622.72,2240.44) | 9819.39(15222.85,5193.38) | 2.29(2.22,2.36) | 3.72(6.43,1.52) | 3.51(5.45,1.88) | -0.30(-0.38,-0.23) |
| Iran (Islamic Republic of) | both | 204.51(326.98,108.18) | 817.95(1221.23,503.15) | 5.24(5.01,5.48) | 0.63(1.01,0.33) | 0.92(1.39,0.56) | 1.51(1.29,1.74) |
| Iraq | both | 72.35(121.74,41.64) | 314.24(507.52,182.57) | 5.27(5.17,5.38) | 0.76(1.24,0.43) | 0.98(1.60,0.57) | 0.97(0.86,1.08) |
| Ireland | both | 140.01(180.49,101.57) | 345.75(446.91,245.05) | 3.22(2.84,3.61) | 4.05(5.20,2.93) | 6.03(7.83,4.32) | 1.23(0.96,1.50) |
| Israel | both | 288.55(377.06,206.95) | 594.73(788.69,418.41) | 2.14(1.80,2.49) | 6.72(8.77,4.77) | 6.40(8.46,4.53) | -0.42(-0.58,-0.25) |
| Italy | both | 6740.84(9027.02,4859.76) | 6137.53(8296.59,4450.03) | -0.11(-0.23,0.01) | 10.41(13.81,7.68) | 7.65(10.01,5.73) | -1.07(-1.16,-0.98) |
| Jamaica | both | 49.84(66.52,34.75) | 77.92(103.97,55.48) | 1.29(1.16,1.41) | 3.01(4.03,2.07) | 2.59(3.43,1.83) | -0.83(-1.02,-0.65) |
| Japan | both | 7860.91(10883.83,5350.72) | 5630.71(7804.44,3788.05) | -0.47(-0.82,-0.11) | 4.87(6.63,3.39) | 3.93(5.40,2.75) | 0.02(-0.34,0.38) |
| Jordan | both | 15.09(24.57,8.55) | 95.06(152.88,55.54) | 6.62(6.13,7.12) | 0.79(1.27,0.45) | 1.01(1.62,0.60) | 0.98(0.90,1.06) |
| Kazakhstan | both | 1021.75(1343.70,726.24) | 4941.80(6272.72,3590.96) | 6.62(6.10,7.15) | 6.93(9.15,4.87) | 24.24(30.78,17.55) | 5.29(4.85,5.73) |
| Kenya | both | 710.77(1171.61,314.91) | 2687.22(4314.50,1313.03) | 4.38(4.07,4.69) | 6.58(11.35,2.67) | 8.14(13.68,3.66) | 0.50(0.25,0.75) |
| Kiribati | both | 1.19(2.02,0.67) | 2.24(3.68,1.23) | 2.17(2.05,2.29) | 1.99(3.37,1.08) | 2.11(3.46,1.12) | 0.13(-0.03,0.29) |
| Kuwait | both | 6.46(10.55,3.68) | 44.59(73.29,25.40) | 7.54(7.31,7.76) | 0.48(0.77,0.27) | 0.85(1.34,0.50) | 2.28(1.88,2.67) |
| Kyrgyzstan | both | 334.28(449.08,235.96) | 1064.17(1397.54,762.72) | 4.58(4.28,4.88) | 10.26(13.90,7.11) | 16.81(22.14,11.93) | 2.04(1.86,2.23) |
| Lao People's Democratic Republic | both | 86.49(139.74,50.17) | 277.52(430.33,168.30) | 4.03(3.77,4.29) | 3.09(5.00,1.78) | 4.36(6.69,2.60) | 1.12(0.89,1.36) |
| Latvia | both | 105.76(143.29,70.24) | 138.50(183.50,95.01) | 1.45(1.06,1.83) | 3.79(5.17,2.52) | 7.16(9.51,4.93) | 2.87(2.49,3.24) |
| Lebanon | both | 19.07(30.71,11.01) | 59.12(91.94,35.67) | 4.12(3.87,4.36) | 0.73(1.16,0.43) | 1.14(1.76,0.68) | 1.73(1.51,1.94) |
| Lesotho | both | 36.95(61.32,20.71) | 57.96(92.69,33.74) | 0.96(0.47,1.45) | 3.07(5.20,1.68) | 3.09(5.02,1.76) | -0.40(-0.83,0.02) |
| Liberia | both | 28.82(47.69,14.49) | 97.01(150.51,57.24) | 3.99(3.60,4.39) | 2.33(3.94,1.15) | 2.94(4.57,1.72) | 0.39(0.16,0.63) |
| Libya | both | 19.88(32.29,11.44) | 66.44(105.01,39.03) | 4.53(4.07,4.99) | 0.86(1.36,0.48) | 0.96(1.51,0.57) | 0.51(0.27,0.76) |
| Lithuania | both | 140.64(194.18,92.69) | 254.02(333.67,172.30) | 2.61(1.82,3.39) | 3.74(5.18,2.46) | 9.84(13.07,6.64) | 4.30(3.64,4.97) |
| Luxembourg | both | 69.38(88.01,50.70) | 93.79(118.03,67.86) | 0.98(0.72,1.23) | 15.61(19.84,11.44) | 11.90(14.98,8.72) | -1.02(-1.26,-0.77) |
| North Macedonia | both | 106.20(139.24,74.87) | 197.85(254.24,143.01) | 2.28(2.20,2.36) | 5.07(6.66,3.56) | 7.29(9.35,5.23) | 1.29(1.23,1.36) |
| Madagascar | both | 180.05(294.71,95.00) | 746.72(1166.91,449.72) | 5.38(4.90,5.87) | 2.68(4.48,1.33) | 4.11(6.41,2.43) | 1.72(1.34,2.10) |
| Malawi | both | 235.68(367.03,139.77) | 528.92(799.21,327.81) | 2.82(2.51,3.13) | 4.58(7.17,2.65) | 5.07(7.76,3.01) | 0.40(0.30,0.51) |
| Malaysia | both | 353.30(531.20,217.11) | 1117.49(1607.17,737.75) | 3.79(3.33,4.25) | 2.65(4.02,1.62) | 3.48(5.01,2.30) | 0.69(0.43,0.94) |
| Maldives | both | 2.29(3.61,1.36) | 13.11(19.68,8.35) | 6.49(5.90,7.09) | 1.79(2.81,1.07) | 2.49(3.69,1.59) | 1.40(1.12,1.69) |
| Mali | both | 129.32(217.71,63.85) | 462.74(731.76,269.51) | 5.01(4.67,5.36) | 2.49(4.17,1.21) | 3.77(5.97,2.19) | 1.82(1.60,2.04) |
| Malta | both | 26.38(34.43,18.77) | 26.20(33.31,18.91) | -0.18(-0.37,0.01) | 6.14(7.96,4.42) | 5.37(6.85,3.84) | -0.36(-0.46,-0.27) |
| Marshall Islands | both | 0.66(1.07,0.36) | 1.37(2.15,0.77) | 2.29(2.18,2.40) | 2.30(3.75,1.24) | 2.43(3.88,1.40) | 0.11(-0.08,0.31) |
| Mauritania | both | 38.18(59.73,22.03) | 85.55(134.22,50.34) | 2.61(2.39,2.83) | 3.11(4.95,1.76) | 3.14(4.96,1.81) | -0.30(-0.51,-0.08) |
| Mauritius | both | 42.41(65.92,25.22) | 46.23(69.81,28.48) | -0.16(-0.56,0.25) | 4.01(6.24,2.38) | 2.99(4.51,1.80) | -1.29(-1.49,-1.10) |
| Mexico | both | 11173.02(15445.86,7403.49) | 24106.77(32911.64,16809.70) | 2.88(2.77,3.00) | 17.87(25.05,11.67) | 17.99(24.53,12.48) | 0.25(0.19,0.30) |
| Republic of Moldova | both | 1745.87(2211.93,1286.40) | 1607.61(2020.43,1179.77) | -0.22(-0.68,0.25) | 39.82(50.70,28.93) | 35.64(44.76,26.10) | -0.19(-0.44,0.06) |
| Mongolia | both | 193.49(272.28,128.93) | 1142.75(1519.18,794.96) | 7.30(6.89,7.71) | 14.86(21.12,9.74) | 30.70(40.67,21.58) | 3.20(2.88,3.53) |
| Montenegro | both | 19.15(25.51,13.25) | 24.66(32.02,17.60) | 0.57(0.37,0.77) | 2.99(3.97,2.06) | 3.56(4.59,2.54) | 0.42(0.19,0.65) |
| Morocco | both | 129.07(210.31,73.07) | 421.20(680.96,244.39) | 4.24(3.94,4.53) | 0.80(1.28,0.45) | 1.11(1.78,0.65) | 1.16(0.89,1.42) |
| Mozambique | both | 169.75(269.09,96.63) | 428.87(651.80,253.90) | 3.26(2.94,3.58) | 2.15(3.38,1.18) | 2.70(4.22,1.48) | 0.76(0.49,1.02) |
| Myanmar | both | 1332.75(2177.42,738.06) | 2851.95(4418.85,1614.19) | 2.50(2.38,2.62) | 4.14(6.93,2.25) | 4.93(7.63,2.81) | 0.51(0.32,0.70) |
| Namibia | both | 25.78(41.96,14.67) | 78.58(120.60,45.89) | 3.31(3.09,3.53) | 2.92(4.82,1.64) | 3.96(6.19,2.32) | 0.61(0.38,0.85) |
| Nepal | both | 757.26(1071.88,491.50) | 2438.67(3276.98,1681.84) | 4.42(4.22,4.62) | 5.24(7.48,3.42) | 8.88(11.97,6.08) | 2.07(1.87,2.27) |
| Netherlands | both | 1102.04(1436.81,799.40) | 1158.72(1466.34,843.89) | 0.06(-0.29,0.42) | 6.45(8.43,4.68) | 5.91(7.65,4.33) | -0.42(-0.61,-0.23) |
| New Zealand | both | 48.27(70.05,29.90) | 73.29(104.41,48.89) | 1.36(1.13,1.59) | 1.38(2.00,0.85) | 1.36(1.90,0.95) | -0.23(-0.32,-0.13) |
| Nicaragua | both | 207.82(283.12,141.10) | 737.99(998.09,512.78) | 4.81(4.64,4.98) | 8.64(11.79,5.88) | 11.93(16.07,8.29) | 1.51(1.27,1.76) |
| Niger | both | 154.42(253.70,83.19) | 471.28(747.15,269.24) | 3.61(3.51,3.72) | 3.75(6.14,1.94) | 4.08(6.43,2.23) | -0.02(-0.12,0.07) |
| Nigeria | both | 1685.41(2827.76,705.99) | 4725.48(7510.90,2333.08) | 3.61(3.41,3.81) | 3.14(5.37,1.23) | 3.75(6.13,1.78) | 0.55(0.41,0.70) |
| Democratic People's Republic of Korea | both | 686.26(1058.29,407.17) | 1123.46(1748.23,673.51) | 1.77(1.59,1.94) | 3.35(5.19,1.99) | 3.42(5.25,2.07) | -0.03(-0.32,0.26) |
| Northern Mariana Islands | both | 2.30(3.71,1.28) | 2.01(3.24,1.14) | -0.73(-1.62,0.17) | 4.57(7.43,2.54) | 3.76(5.82,2.28) | -1.09(-1.25,-0.93) |
| Norway | both | 246.98(338.06,167.51) | 326.77(447.79,219.25) | 1.93(1.61,2.26) | 5.38(7.32,3.64) | 5.35(7.23,3.67) | 1.01(0.68,1.34) |
| Oman | both | 13.74(22.57,7.72) | 65.13(103.95,38.63) | 5.65(5.21,6.09) | 1.30(2.18,0.72) | 2.02(3.22,1.20) | 1.53(1.25,1.81) |
| Pakistan | both | 1044.93(1732.36,491.25) | 2203.15(3476.06,1145.06) | 2.65(2.50,2.80) | 1.50(2.52,0.68) | 1.39(2.26,0.68) | -0.23(-0.30,-0.16) |
| Palestine | both | 8.83(14.21,5.10) | 41.63(67.80,24.79) | 5.94(5.60,6.29) | 0.90(1.44,0.51) | 1.28(2.09,0.75) | 1.50(1.22,1.79) |
| Panama | both | 126.12(171.00,88.25) | 326.34(429.64,231.83) | 3.48(3.39,3.58) | 6.79(9.12,4.79) | 7.61(10.05,5.41) | 0.50(0.35,0.66) |
| Papua New Guinea | both | 20.74(33.90,11.05) | 64.59(102.34,36.09) | 4.06(3.99,4.13) | 0.63(1.05,0.33) | 0.70(1.12,0.39) | 0.38(0.28,0.48) |
| Paraguay | both | 146.34(188.65,106.27) | 425.84(551.65,312.81) | 3.66(3.46,3.85) | 5.05(6.55,3.69) | 6.39(8.30,4.73) | 0.78(0.53,1.02) |
| Peru | both | 1285.53(1686.27,941.28) | 4151.62(5385.11,3029.92) | 4.20(4.02,4.39) | 8.36(11.07,6.05) | 12.00(15.57,8.71) | 1.32(1.12,1.52) |
| Philippines | both | 933.59(1440.37,515.58) | 3498.00(5249.50,2065.82) | 4.98(4.88,5.08) | 2.12(3.41,1.10) | 3.39(5.20,1.95) | 1.92(1.78,2.05) |
| Poland | both | 3126.05(4295.65,2125.40) | 3523.77(4952.88,2315.28) | 0.27(-0.01,0.55) | 7.57(10.48,5.10) | 7.73(10.85,5.16) | 0.25(-0.04,0.54) |
| Portugal | both | 1693.19(2130.70,1250.17) | 1063.40(1356.86,760.08) | -1.81(-2.02,-1.60) | 16.18(20.47,12.01) | 8.31(10.64,5.96) | -2.57(-2.70,-2.44) |
| Puerto Rico | both | 422.62(562.89,291.10) | 373.97(494.59,259.67) | -0.94(-1.26,-0.62) | 11.77(15.62,8.09) | 9.06(11.87,6.28) | -1.40(-1.60,-1.20) |
| Qatar | both | 5.22(8.81,2.95) | 66.91(104.17,39.07) | 10.40(9.94,10.85) | 1.84(2.95,1.03) | 2.89(4.55,1.72) | 1.95(1.56,2.34) |
| Romania | both | 4100.55(5238.49,3047.17) | 5500.43(6854.24,4128.93) | 1.18(0.89,1.47) | 16.72(21.64,12.36) | 22.24(27.75,16.94) | 1.21(0.91,1.51) |
| Russian Federation | both | 8420.53(12161.79,5299.08) | 15224.71(22686.92,8997.42) | 1.90(1.77,2.03) | 5.21(7.50,3.23) | 9.09(13.35,5.48) | 2.11(2.06,2.17) |
| Rwanda | both | 180.18(283.36,103.25) | 508.80(772.54,313.80) | 4.49(4.04,4.95) | 4.60(7.51,2.54) | 5.87(8.92,3.58) | 1.00(0.90,1.09) |
| Saint Lucia | both | 6.77(9.03,4.54) | 14.99(19.98,10.37) | 2.64(2.55,2.73) | 7.26(9.62,4.82) | 6.80(9.02,4.78) | -0.49(-0.69,-0.29) |
| Saint Vincent and the Grenadines | both | 3.73(5.08,2.53) | 7.02(9.45,4.86) | 2.05(1.95,2.15) | 5.16(7.05,3.50) | 5.47(7.40,3.79) | -0.03(-0.18,0.12) |
| Samoa | both | 2.38(3.79,1.39) | 4.02(6.40,2.33) | 1.67(1.36,1.98) | 2.19(3.56,1.25) | 2.24(3.56,1.31) | -0.13(-0.30,0.04) |
| Sao Tome and Principe | both | 3.32(5.23,1.95) | 8.34(12.95,4.96) | 3.28(2.86,3.71) | 4.77(7.55,2.74) | 5.30(8.14,3.15) | 0.18(0.02,0.33) |
| Saudi Arabia | both | 139.34(221.28,81.59) | 657.74(1053.89,386.79) | 5.31(5.21,5.40) | 1.70(2.65,0.98) | 2.15(3.44,1.29) | 0.88(0.79,0.98) |
| Senegal | both | 135.49(211.70,77.84) | 386.00(586.92,223.53) | 3.70(3.58,3.83) | 3.27(5.23,1.82) | 3.76(5.78,2.13) | 0.45(0.28,0.61) |
| Serbia | both | 663.28(853.84,474.53) | 684.44(858.73,510.35) | -0.04(-0.19,0.11) | 6.42(8.27,4.58) | 6.97(8.80,5.15) | 0.36(0.27,0.46) |
| Seychelles | both | 2.32(3.58,1.37) | 6.85(10.36,4.25) | 4.09(3.72,4.47) | 4.10(6.42,2.41) | 5.33(7.93,3.29) | 1.08(0.88,1.27) |
| Sierra Leone | both | 50.12(82.13,25.27) | 185.74(289.41,111.09) | 5.06(4.59,5.53) | 2.24(3.72,1.11) | 3.53(5.53,2.03) | 1.78(1.50,2.07) |
| Singapore | both | 62.32(95.10,38.60) | 94.69(143.59,58.94) | 1.17(0.97,1.38) | 1.86(2.86,1.14) | 1.16(1.75,0.74) | -1.91(-2.06,-1.77) |
| Slovakia | both | 692.27(904.28,478.18) | 897.40(1144.98,641.99) | 1.04(0.86,1.21) | 12.14(15.67,8.51) | 13.34(16.97,9.61) | 0.67(0.48,0.86) |
| Slovenia | both | 345.25(448.50,242.40) | 323.77(412.26,236.64) | -0.08(-0.29,0.14) | 15.71(20.43,11.02) | 12.76(16.30,9.38) | -0.52(-0.70,-0.35) |
| Solomon Islands | both | 4.91(8.18,2.55) | 14.30(23.39,7.95) | 3.82(3.56,4.07) | 2.10(3.58,1.06) | 2.41(3.92,1.34) | 0.43(0.28,0.58) |
| Somalia | both | 154.87(256.77,81.41) | 407.64(652.72,225.00) | 3.37(3.26,3.47) | 3.81(6.45,1.90) | 3.90(6.37,2.08) | 0.11(0.03,0.19) |
| South Africa | both | 617.49(988.18,303.45) | 1136.45(1789.42,602.55) | 1.80(1.67,1.94) | 2.26(3.83,1.02) | 2.04(3.28,1.03) | -0.52(-0.71,-0.33) |
| Republic of Korea | both | 9593.64(12442.67,7089.43) | 7552.52(9394.80,5650.75) | -1.87(-2.30,-1.44) | 21.75(28.32,15.93) | 10.44(13.03,7.97) | -3.55(-3.87,-3.22) |
| South Sudan | both | 101.43(161.34,57.04) | 189.43(301.14,106.47) | 2.40(2.28,2.51) | 3.18(5.16,1.70) | 3.25(5.19,1.78) | 0.16(0.08,0.25) |
| Spain | both | 6114.28(7442.24,4782.27) | 6066.67(7596.82,4656.10) | 0.18(-0.05,0.41) | 15.48(18.88,12.07) | 9.80(12.19,7.65) | -1.72(-1.82,-1.63) |
| Sri Lanka | both | 545.03(849.85,315.97) | 1143.44(1706.94,726.55) | 2.18(1.99,2.38) | 3.43(5.36,2.01) | 4.55(6.77,2.93) | 0.68(0.52,0.84) |
| Sudan | both | 57.21(93.36,30.10) | 197.72(318.09,114.29) | 4.07(3.45,4.70) | 0.54(0.90,0.28) | 0.83(1.29,0.47) | 1.26(0.75,1.76) |
| Suriname | both | 22.42(30.80,15.46) | 57.68(78.91,40.66) | 3.48(3.30,3.66) | 7.20(9.94,4.92) | 8.77(11.78,6.20) | 0.46(0.39,0.54) |
| Eswatini | both | 10.69(17.50,5.88) | 24.84(38.36,14.54) | 2.69(2.34,3.04) | 2.29(3.85,1.21) | 2.68(4.23,1.53) | 0.57(0.10,1.05) |
| Sweden | both | 552.09(744.61,376.23) | 713.77(957.06,495.84) | 1.07(0.88,1.25) | 5.70(7.62,3.96) | 6.36(8.44,4.48) | 0.56(0.35,0.77) |
| Switzerland | both | 640.65(820.62,469.84) | 762.76(977.15,554.96) | 0.70(0.44,0.97) | 7.87(10.08,5.75) | 7.16(9.03,5.24) | -0.36(-0.56,-0.15) |
| Syrian Arab Republic | both | 60.46(98.05,34.54) | 212.82(343.17,126.02) | 4.98(4.65,5.31) | 0.92(1.49,0.53) | 1.38(2.20,0.82) | 1.69(1.52,1.85) |
| Taiwan (Province of China) | both | 1333.24(1857.05,892.93) | 1523.96(2213.78,992.59) | 0.15(-0.44,0.74) | 6.71(9.33,4.49) | 4.81(7.04,3.08) | -1.36(-1.77,-0.95) |
| Tajikistan | both | 244.15(329.27,170.07) | 1537.40(2029.96,1100.68) | 7.31(6.86,7.77) | 7.44(10.19,5.10) | 18.55(24.52,13.13) | 3.60(3.37,3.82) |
| United Republic of Tanzania | both | 488.29(748.58,299.22) | 1413.67(2134.56,863.73) | 3.66(3.53,3.79) | 3.48(5.35,2.09) | 4.04(6.05,2.44) | 0.49(0.42,0.56) |
| Thailand | both | 1789.39(2630.66,1108.76) | 4397.61(6578.59,2786.22) | 3.57(3.19,3.94) | 3.43(5.16,2.11) | 4.78(7.10,3.01) | 1.51(1.32,1.70) |
| Bahamas | both | 16.39(22.21,11.27) | 29.91(40.36,21.14) | 1.78(1.59,1.97) | 7.58(10.38,5.22) | 6.51(8.74,4.60) | -0.93(-1.12,-0.74) |
| Gambia | both | 18.68(29.45,10.59) | 56.89(85.16,35.29) | 3.79(3.61,3.97) | 3.69(5.93,2.06) | 4.16(6.35,2.54) | 0.35(0.19,0.52) |
| Timor-Leste | both | 16.49(27.06,9.42) | 41.03(64.11,24.86) | 2.94(2.72,3.16) | 2.98(4.89,1.70) | 4.37(6.89,2.57) | 1.25(1.11,1.38) |
| Togo | both | 56.76(88.48,32.62) | 227.90(361.41,130.98) | 4.62(4.44,4.79) | 3.09(4.89,1.71) | 3.92(6.20,2.24) | 0.53(0.35,0.71) |
| Tonga | both | 1.76(2.86,1.01) | 2.67(4.25,1.58) | 1.51(1.21,1.81) | 2.70(4.35,1.55) | 3.04(4.80,1.80) | 0.23(-0.01,0.48) |
| Trinidad and Tobago | both | 48.58(66.31,32.65) | 72.01(95.94,50.37) | 1.16(1.05,1.27) | 4.65(6.30,3.13) | 4.36(5.76,3.02) | -0.35(-0.48,-0.22) |
| Tunisia | both | 68.86(110.03,38.64) | 218.45(345.23,131.47) | 4.19(4.12,4.26) | 1.20(1.90,0.68) | 1.58(2.49,0.97) | 0.98(0.92,1.04) |
| Turkey | both | 260.72(409.74,156.68) | 1080.08(1684.75,643.50) | 5.77(5.42,6.12) | 0.60(0.93,0.36) | 1.12(1.75,0.67) | 2.86(2.53,3.19) |
| Turkmenistan | both | 267.12(359.90,184.01) | 852.67(1137.93,598.36) | 4.48(4.34,4.61) | 10.98(15.06,7.59) | 15.98(21.44,11.20) | 1.43(1.26,1.61) |
| Uganda | both | 339.92(526.23,205.35) | 964.14(1441.14,586.67) | 3.64(3.43,3.84) | 3.98(6.27,2.32) | 4.58(7.00,2.71) | 0.46(0.35,0.56) |
| Ukraine | both | 2985.37(4417.62,1827.50) | 4248.60(6855.41,2101.33) | 1.54(1.44,1.65) | 5.48(7.94,3.41) | 8.64(13.77,4.46) | 2.06(1.90,2.22) |
| United Arab Emirates | both | 11.46(19.22,6.23) | 151.94(253.99,84.56) | 9.05(8.80,9.31) | 1.04(1.72,0.56) | 1.27(2.03,0.74) | 0.50(0.28,0.73) |
| United Kingdom | both | 4104.76(5452.63,2890.33) | 8422.12(10583.25,6497.67) | 2.16(1.72,2.61) | 6.71(8.90,4.72) | 12.02(15.13,9.32) | 1.67(1.31,2.03) |
| United States of America | both | 16777.92(22720.78,11687.97) | 19543.95(26402.45,14147.25) | 0.36(0.13,0.59) | 6.06(8.19,4.19) | 5.47(7.24,4.02) | -0.39(-0.49,-0.30) |
| Uruguay | both | 153.34(217.60,99.41) | 184.93(258.20,122.14) | 0.55(0.45,0.65) | 4.88(6.91,3.13) | 4.77(6.65,3.17) | -0.25(-0.34,-0.16) |
| Uzbekistan | both | 1517.74(2062.17,1060.59) | 7687.58(9851.24,5557.81) | 6.20(5.92,6.49) | 11.44(15.48,7.95) | 22.73(29.25,16.37) | 2.53(2.33,2.73) |
| Vanuatu | both | 3.37(5.42,1.88) | 7.89(12.69,4.32) | 3.04(2.88,3.21) | 3.09(5.09,1.70) | 3.23(5.24,1.77) | 0.13(0.04,0.21) |
| Venezuela (Bolivarian Republic of) | both | 1182.30(1600.22,820.55) | 2117.93(2853.46,1466.60) | 2.10(1.95,2.25) | 8.26(11.05,5.66) | 6.68(8.98,4.62) | -0.77(-0.84,-0.70) |
| Viet nam | both | 1677.75(2612.43,1048.75) | 6443.38(9643.36,4109.01) | 4.88(4.59,5.18) | 3.74(5.89,2.27) | 5.56(8.27,3.57) | 1.43(1.02,1.85) |
| Virginia | both | 445.33(607.43,308.48) | 521.34(704.19,378.56) | 0.31(0.12,0.51) | 6.15(8.38,4.25) | 5.31(7.05,3.91) | -0.62(-0.70,-0.53) |
| Yemen | both | 53.09(85.49,29.12) | 205.65(335.85,118.97) | 4.88(4.49,5.27) | 0.85(1.36,0.46) | 1.13(1.85,0.64) | 1.09(0.79,1.40) |
| Zambia | both | 204.58(322.88,120.47) | 628.56(950.86,393.57) | 3.86(3.40,4.33) | 5.04(8.06,2.91) | 5.94(9.07,3.64) | 0.51(0.30,0.72) |
| Zimbabwe | both | 160.09(251.34,96.71) | 325.81(520.25,190.81) | 2.01(1.37,2.66) | 2.88(4.60,1.69) | 3.11(4.98,1.75) | 0.04(-0.51,0.60) |
| Monaco | both | 3.08(4.09,2.17) | 3.83(5.07,2.67) | 1.04(0.79,1.29) | 8.60(11.36,6.08) | 9.78(12.60,6.83) | 0.56(0.44,0.68) |
| San Marino | both | 3.77(4.86,2.68) | 5.40(7.01,3.84) | 1.48(1.28,1.69) | 15.18(19.58,10.83) | 14.05(17.93,9.94) | -0.27(-0.33,-0.21) |
| Saint Kitts and Nevis | both | 2.71(3.64,1.84) | 7.41(10.01,5.23) | 3.51(3.31,3.70) | 10.00(13.60,6.72) | 9.68(13.02,6.85) | -0.35(-0.52,-0.18) |
| Cook Islands | both | 0.11(0.17,0.07) | 0.16(0.26,0.10) | 1.58(1.24,1.93) | 0.70(1.09,0.41) | 0.85(1.31,0.51) | 0.70(0.56,0.84) |
| Nauru | both | 0.16(0.26,0.09) | 0.23(0.36,0.13) | 0.92(0.73,1.10) | 1.95(3.24,1.09) | 2.41(3.78,1.38) | 0.66(0.38,0.94) |
| Niue | both | 0.03(0.05,0.02) | 0.04(0.06,0.02) | 0.04(-0.25,0.33) | 1.77(2.82,1.02) | 2.20(3.34,1.30) | 0.63(0.33,0.93) |
| Palau | both | 0.27(0.43,0.16) | 0.57(0.91,0.32) | 2.00(1.14,2.86) | 1.86(2.95,1.05) | 2.38(3.71,1.37) | 0.86(0.57,1.16) |
| Tokelau | both | 0.02(0.03,0.01) | 0.02(0.03,0.01) | 0.05(-0.24,0.33) | 1.38(2.23,0.80) | 1.45(2.28,0.86) | 0.00(-0.06,0.07) |
| Tuvalu | both | 0.12(0.19,0.07) | 0.21(0.32,0.12) | 1.89(1.84,1.94) | 1.42(2.33,0.82) | 1.89(2.94,1.11) | 0.92(0.87,0.97) |
| Afghanistan | male | 38.68(67.28,19.16) | 149.28(262.10,79.44) | 4.80(4.24,5.36) | 1.03(1.76,0.53) | 1.35(2.21,0.74) | 0.72(0.57,0.88) |
| Albania | male | 137.58(179.76,95.50) | 156.58(197.85,116.52) | 0.83(0.61,1.04) | 9.72(12.64,6.76) | 10.65(13.50,7.83) | 0.64(0.40,0.88) |
| Algeria | male | 68.43(112.11,38.19) | 276.40(447.28,161.45) | 4.68(4.41,4.95) | 0.97(1.59,0.53) | 1.33(2.14,0.77) | 0.78(0.50,1.06) |
| American Samoa | male | 0.53(0.83,0.29) | 0.57(0.92,0.32) | -0.18(-0.52,0.17) | 2.61(4.14,1.39) | 2.22(3.55,1.24) | -0.83(-0.94,-0.73) |
| Andorra | male | 3.92(4.95,2.87) | 6.60(8.47,4.89) | 1.51(1.04,1.98) | 11.31(14.33,8.27) | 10.38(13.00,7.72) | -0.33(-0.45,-0.22) |
| Angola | male | 66.74(106.43,36.85) | 327.91(513.41,197.32) | 5.68(5.17,6.18) | 2.14(3.45,1.14) | 3.86(6.06,2.25) | 2.04(1.62,2.45) |
| Antigua and Barbuda | male | 2.06(2.79,1.41) | 4.66(6.18,3.28) | 2.90(2.66,3.14) | 8.66(11.73,5.83) | 8.76(11.57,6.22) | -0.04(-0.15,0.06) |
| Argentina | male | 1382.31(1952.74,905.24) | 2939.48(4120.81,1898.21) | 2.71(2.56,2.86) | 8.97(12.70,5.87) | 12.53(17.57,8.10) | 1.25(1.15,1.36) |
| Armenia | male | 119.27(160.10,84.30) | 405.79(526.05,293.98) | 5.09(4.82,5.36) | 8.01(10.58,5.61) | 24.65(31.92,17.81) | 4.66(4.39,4.93) |
| Australia | male | 242.13(358.27,147.56) | 343.94(515.37,208.91) | 1.38(1.19,1.57) | 2.64(3.89,1.62) | 2.43(3.65,1.48) | -0.06(-0.27,0.16) |
| Austria | male | 1136.03(1406.57,855.04) | 985.40(1204.87,752.08) | -0.36(-0.61,-0.11) | 26.73(33.02,19.97) | 18.51(22.66,14.21) | -1.28(-1.39,-1.18) |
| Azerbaijan | male | 343.12(465.55,237.13) | 1354.38(1809.79,958.13) | 5.24(5.08,5.40) | 12.06(16.39,8.27) | 22.13(29.38,15.77) | 2.14(2.06,2.23) |
| Bahrain | male | 3.34(5.38,1.94) | 28.36(46.09,16.55) | 8.71(8.26,9.16) | 1.83(2.93,1.06) | 2.23(3.44,1.36) | 0.98(0.69,1.27) |
| Bangladesh | male | 1236.87(2020.02,708.12) | 3490.28(5269.11,2127.95) | 3.70(3.49,3.91) | 3.09(5.03,1.70) | 4.56(6.89,2.80) | 1.45(1.29,1.60) |
| Barbados | male | 9.17(12.23,6.26) | 13.17(17.43,9.31) | 1.08(0.89,1.28) | 8.18(10.93,5.60) | 7.82(10.26,5.49) | -0.33(-0.41,-0.25) |
| Belarus | male | 288.93(388.06,193.50) | 718.99(923.83,495.69) | 3.96(3.50,4.41) | 5.48(7.42,3.65) | 13.77(17.70,9.53) | 4.33(3.80,4.85) |
| Belgium | male | 723.05(873.57,578.36) | 871.71(1071.59,674.03) | 0.69(0.44,0.95) | 13.08(15.80,10.48) | 13.38(16.40,10.40) | 0.10(-0.06,0.26) |
| Belize | male | 4.77(6.49,3.21) | 22.94(31.12,15.68) | 5.58(5.36,5.80) | 8.32(11.36,5.62) | 11.94(16.10,8.14) | 1.10(0.98,1.21) |
| Benin | male | 42.01(68.10,21.81) | 207.65(313.18,122.34) | 5.47(4.91,6.03) | 3.56(5.93,1.73) | 5.61(8.64,3.29) | 1.39(0.88,1.89) |
| Bermuda | male | 3.07(4.06,2.15) | 2.99(3.97,2.10) | -0.30(-0.48,-0.13) | 9.03(11.92,6.26) | 7.15(9.31,5.09) | -1.05(-1.17,-0.93) |
| Bhutan | male | 11.08(17.32,6.44) | 31.07(46.98,18.31) | 4.33(4.02,4.64) | 4.60(7.36,2.56) | 7.21(11.01,4.24) | 1.89(1.67,2.12) |
| Bolivia (Plurinational State of) | male | 231.47(327.47,148.80) | 807.16(1093.74,580.95) | 4.36(4.21,4.50) | 10.86(15.43,6.95) | 15.41(20.97,11.03) | 1.17(1.08,1.26) |
| Bosnia and Herzegovina | male | 285.61(371.72,200.35) | 227.45(289.39,164.38) | -0.60(-0.74,-0.46) | 11.89(15.60,8.32) | 11.37(14.54,8.17) | -0.18(-0.34,-0.02) |
| Botswana | male | 19.74(31.31,10.99) | 75.68(117.02,44.10) | 4.77(4.60,4.95) | 5.07(8.38,2.75) | 6.60(10.35,3.78) | 1.23(1.05,1.41) |
| Brazil | male | 9141.42(12795.41,6067.78) | 17342.82(24071.83,11686.62) | 2.07(1.92,2.22) | 14.25(20.23,9.10) | 14.24(19.69,9.57) | -0.14(-0.21,-0.06) |
| Brunei Darussalam | male | 4.81(7.34,2.93) | 8.83(12.94,5.37) | 1.24(0.88,1.60) | 3.66(5.44,2.21) | 2.97(4.32,1.82) | -1.32(-1.53,-1.12) |
| Bulgaria | male | 673.35(879.67,475.64) | 860.92(1104.82,619.06) | 1.19(1.04,1.34) | 14.21(18.47,10.08) | 20.69(26.20,14.95) | 1.62(1.45,1.78) |
| Burkina Faso | male | 92.47(145.28,52.88) | 306.19(484.58,172.91) | 4.05(3.75,4.36) | 3.73(5.86,2.14) | 4.84(7.64,2.62) | 0.63(0.41,0.84) |
| Burundi | male | 59.06(95.60,31.60) | 191.04(299.31,105.72) | 4.52(4.06,4.98) | 3.88(6.42,1.97) | 5.05(7.90,2.72) | 1.12(0.87,1.38) |
| Cambodia | male | 212.14(350.64,114.70) | 838.58(1314.06,491.34) | 4.84(4.62,5.07) | 7.30(12.31,3.80) | 11.13(17.68,6.30) | 1.44(1.16,1.73) |
| Cameroon | male | 142.78(231.20,79.96) | 534.51(821.14,322.32) | 4.72(4.56,4.87) | 4.69(7.66,2.52) | 5.60(8.67,3.13) | 0.59(0.49,0.69) |
| Canada | male | 1121.61(1562.96,752.84) | 1387.05(1961.70,909.54) | 0.72(0.44,1.00) | 7.50(10.47,5.00) | 6.56(9.23,4.30) | -0.52(-0.57,-0.46) |
| Cabo Verde | male | 2.78(4.57,1.52) | 13.34(20.33,7.84) | 5.61(5.53,5.68) | 3.52(5.65,1.82) | 4.52(6.92,2.58) | 0.64(0.49,0.80) |
| Central African Republic | male | 41.04(70.59,20.24) | 98.52(166.36,49.52) | 2.84(2.72,2.96) | 4.80(8.61,2.16) | 5.40(9.29,2.59) | 0.15(-0.02,0.32) |
| Chad | male | 73.01(116.48,40.06) | 203.63(330.84,117.41) | 3.45(3.36,3.55) | 4.71(7.56,2.45) | 4.73(7.64,2.62) | -0.23(-0.33,-0.13) |
| Chile | male | 902.46(1302.87,584.61) | 1794.99(2513.73,1188.69) | 2.22(2.00,2.43) | 15.62(22.39,9.98) | 17.08(23.80,11.30) | -0.04(-0.17,0.09) |
| China | male | 29901.78(44191.48,17881.48) | 52363.79(74028.47,34385.98) | 2.26(2.18,2.35) | 5.14(7.67,2.96) | 5.50(7.62,3.79) | 0.45(0.25,0.65) |
| Colombia | male | 855.27(1147.35,593.28) | 1438.35(1896.11,1019.67) | 1.77(1.60,1.94) | 6.77(9.09,4.71) | 5.82(7.69,4.12) | -0.58(-0.63,-0.52) |
| Comoros | male | 4.76(7.81,2.43) | 14.02(22.17,7.64) | 3.91(3.71,4.11) | 3.45(5.76,1.74) | 4.44(7.11,2.41) | 0.92(0.74,1.10) |
| Congo | male | 21.16(34.21,11.60) | 78.10(120.37,46.68) | 4.49(4.16,4.81) | 3.02(5.02,1.52) | 3.72(5.75,2.18) | 0.51(0.29,0.73) |
| Costa Rica | male | 139.50(192.35,95.13) | 295.61(399.23,206.48) | 2.53(2.25,2.80) | 11.11(15.45,7.51) | 12.00(16.27,8.31) | 0.29(0.26,0.33) |
| Côte d'Ivoire | male | 156.03(244.53,86.06) | 556.28(855.88,323.69) | 4.27(3.94,4.60) | 4.25(6.93,2.26) | 5.65(8.75,3.25) | 0.78(0.47,1.09) |
| Croatia | male | 637.13(824.70,454.37) | 525.08(652.19,391.15) | -0.58(-0.74,-0.43) | 22.86(29.76,16.30) | 21.09(26.29,15.60) | -0.10(-0.26,0.06) |
| Cuba | male | 287.57(379.74,205.25) | 601.07(810.41,413.46) | 2.97(2.63,3.32) | 5.43(7.16,3.88) | 8.79(11.66,6.07) | 1.76(1.68,1.83) |
| Cyprus | male | 39.88(52.00,28.91) | 82.90(104.67,61.60) | 2.46(2.25,2.68) | 10.16(13.23,7.35) | 10.69(13.49,7.94) | 0.10(-0.03,0.23) |
| Czechia | male | 653.45(850.39,462.59) | 846.35(1062.58,625.36) | 1.14(0.88,1.41) | 11.05(14.27,7.92) | 12.70(15.80,9.49) | 0.63(0.50,0.77) |
| Democratic Republic of the Congo | male | 394.92(636.18,220.86) | 1571.52(2455.24,939.83) | 4.76(4.43,5.09) | 3.74(6.24,1.94) | 5.52(8.89,3.15) | 1.15(0.90,1.40) |
| Denmark | male | 279.12(357.40,198.98) | 333.26(422.05,242.45) | 0.81(0.32,1.30) | 9.42(12.04,6.75) | 10.65(13.60,7.82) | 0.51(0.07,0.95) |
| Djibouti | male | 5.81(9.33,3.22) | 23.77(37.33,13.82) | 5.00(4.82,5.17) | 3.74(6.06,2.01) | 4.16(6.58,2.43) | 0.20(0.01,0.40) |
| Dominica | male | 1.96(2.68,1.32) | 2.60(3.53,1.78) | 0.62(0.36,0.88) | 7.12(9.61,4.75) | 6.86(9.15,4.72) | -0.52(-0.67,-0.37) |
| Dominican Republic | male | 241.92(333.28,167.82) | 792.42(1070.00,549.06) | 3.97(3.85,4.08) | 9.95(13.72,6.85) | 14.45(19.64,10.01) | 1.06(0.94,1.18) |
| Ecuador | male | 321.36(429.77,228.12) | 1483.60(1852.61,1135.34) | 5.18(4.92,5.44) | 8.94(12.03,6.31) | 18.28(22.97,14.01) | 2.25(2.01,2.48) |
| Egypt | male | 461.65(738.79,263.46) | 1871.62(3024.57,1056.70) | 5.10(4.94,5.27) | 2.50(4.03,1.44) | 4.33(6.96,2.45) | 2.07(1.90,2.23) |
| El Salvador | male | 226.70(316.80,148.79) | 349.02(491.69,232.55) | 1.33(1.19,1.48) | 12.22(17.23,7.93) | 13.16(18.59,8.71) | 0.15(0.00,0.31) |
| Equatorial Guinea | male | 5.51(9.65,2.48) | 17.24(26.67,10.64) | 4.06(3.69,4.44) | 4.56(8.11,1.97) | 4.55(7.25,2.59) | -0.02(-0.11,0.07) |
| Eritrea | male | 30.11(49.87,15.83) | 141.53(223.65,78.72) | 5.56(5.29,5.83) | 3.40(5.70,1.70) | 5.78(9.21,3.07) | 2.00(1.62,2.37) |
| Estonia | male | 42.29(57.92,27.77) | 93.97(120.51,65.33) | 3.08(2.79,3.37) | 5.27(7.19,3.45) | 13.31(17.15,9.33) | 3.52(3.14,3.91) |
| Ethiopia | male | 487.56(903.22,174.70) | 1062.71(1739.94,486.15) | 2.55(2.36,2.74) | 3.55(6.80,1.18) | 3.44(6.03,1.43) | -0.10(-0.24,0.05) |
| Micronesia (Federated States of) | male | 1.46(2.50,0.77) | 1.95(3.21,1.05) | 0.73(0.56,0.90) | 3.75(6.31,1.95) | 3.97(6.59,2.10) | 0.08(-0.02,0.19) |
| Fiji | male | 6.12(9.72,3.37) | 9.16(14.60,5.21) | 1.32(1.10,1.54) | 1.80(2.89,0.98) | 1.88(2.98,1.08) | 0.08(-0.02,0.17) |
| Finland | male | 217.09(282.58,152.84) | 320.39(411.65,233.14) | 1.68(1.30,2.06) | 7.08(9.05,5.00) | 11.54(14.84,8.40) | 2.15(1.73,2.58) |
| France | male | 5822.55(7373.93,4396.96) | 4462.39(5597.12,3311.40) | -1.22(-1.39,-1.06) | 18.81(23.58,14.17) | 12.64(15.84,9.48) | -1.61(-1.69,-1.53) |
| Gabon | male | 8.16(12.47,4.65) | 21.00(32.84,12.93) | 3.26(2.98,3.53) | 2.53(3.97,1.37) | 2.90(4.50,1.76) | 0.31(0.05,0.56) |
| Georgia | male | 378.65(499.36,264.60) | 348.75(467.95,235.82) | -0.28(-0.38,-0.17) | 14.15(18.71,9.87) | 18.25(24.57,12.31) | -0.40(-3.26,2.56) |
| Germany | male | 8977.45(11099.03,6709.73) | 7957.52(9821.01,6091.47) | -0.36(-0.70,-0.02) | 19.53(24.42,14.66) | 16.76(20.66,12.85) | -0.51(-0.66,-0.36) |
| Ghana | male | 171.84(278.32,89.81) | 505.31(762.80,297.32) | 3.51(3.31,3.71) | 3.79(6.26,1.84) | 4.41(6.73,2.58) | 0.32(0.12,0.53) |
| Greece | male | 696.67(881.47,512.34) | 543.90(692.02,392.91) | -0.88(-1.01,-0.76) | 11.88(15.02,8.79) | 8.76(11.06,6.40) | -1.18(-1.30,-1.05) |
| Greenland | male | 1.26(1.84,0.79) | 1.12(1.65,0.69) | 0.02(-0.26,0.30) | 3.45(5.01,2.18) | 3.49(5.16,2.18) | 0.14(0.04,0.24) |
| Grenada | male | 2.90(3.97,2.00) | 7.83(10.44,5.58) | 3.31(2.86,3.77) | 10.26(14.04,6.94) | 13.07(17.26,9.34) | 0.65(0.57,0.72) |
| Guam | male | 2.96(4.71,1.71) | 2.65(4.25,1.45) | -0.87(-1.18,-0.55) | 4.10(6.56,2.34) | 3.14(5.01,1.76) | -1.24(-1.35,-1.14) |
| Guatemala | male | 418.77(588.11,268.24) | 1333.62(1859.97,893.13) | 4.26(4.11,4.40) | 15.07(21.52,9.54) | 18.21(25.98,11.97) | 0.84(0.76,0.93) |
| Guinea | male | 62.97(105.28,31.55) | 132.67(210.57,75.45) | 1.99(1.63,2.34) | 3.39(5.67,1.66) | 3.69(5.93,2.10) | -0.23(-0.57,0.10) |
| Guinea-Bissau | male | 13.83(22.66,7.47) | 28.91(44.65,16.84) | 1.95(1.78,2.13) | 5.37(9.08,2.73) | 4.98(7.91,2.76) | -0.83(-0.97,-0.69) |
| Guyana | male | 53.82(73.93,35.42) | 77.35(103.31,51.69) | 1.27(1.17,1.38) | 18.39(25.33,11.76) | 20.51(27.27,13.66) | 0.43(0.30,0.57) |
| Haiti | male | 231.85(335.83,145.50) | 650.12(902.03,429.28) | 3.52(3.45,3.59) | 10.47(15.13,6.52) | 13.02(18.38,8.53) | 0.73(0.68,0.78) |
| Honduras | male | 178.11(254.62,118.04) | 475.15(664.56,316.18) | 3.47(3.37,3.57) | 11.91(17.03,7.70) | 12.34(17.66,8.17) | 0.17(0.08,0.26) |
| Hungary | male | 1676.57(2203.83,1150.55) | 1201.41(1478.73,940.24) | -1.43(-1.65,-1.22) | 29.39(38.31,20.34) | 19.55(23.90,15.41) | -1.78(-2.18,-1.39) |
| Iceland | male | 5.35(7.01,3.83) | 8.57(11.05,6.08) | 1.55(1.33,1.77) | 4.13(5.40,2.98) | 4.50(5.85,3.19) | 0.33(0.29,0.37) |
| India | male | 13859.15(22847.76,6064.91) | 47254.61(73108.85,24545.67) | 4.60(4.27,4.94) | 3.77(6.28,1.60) | 6.46(10.17,3.28) | 2.14(1.82,2.46) |
| Indonesia | male | 2905.03(4863.97,1267.65) | 6273.17(9823.69,3379.60) | 2.87(2.78,2.95) | 4.25(7.40,1.69) | 4.37(6.78,2.35) | 0.22(0.10,0.35) |
| Iran (Islamic Republic of) | male | 145.82(234.65,73.56) | 542.84(829.49,326.44) | 5.19(4.89,5.48) | 0.85(1.39,0.44) | 1.19(1.84,0.70) | 1.54(1.25,1.84) |
| Iraq | male | 46.89(81.84,26.40) | 202.60(332.04,112.91) | 5.24(5.17,5.31) | 0.93(1.60,0.52) | 1.19(1.96,0.67) | 0.93(0.84,1.01) |
| Ireland | male | 81.54(102.99,59.77) | 215.78(279.03,155.31) | 3.56(3.15,3.98) | 4.68(5.92,3.44) | 7.65(9.84,5.56) | 1.62(1.35,1.90) |
| Israel | male | 169.30(220.62,121.63) | 348.36(456.65,244.30) | 2.09(1.76,2.42) | 8.09(10.51,5.82) | 7.54(9.84,5.32) | -0.51(-0.68,-0.34) |
| Italy | male | 4232.39(5634.34,3070.95) | 3959.75(5384.01,2895.36) | 0.03(-0.10,0.16) | 13.37(17.58,9.81) | 10.35(13.65,7.76) | -0.84(-0.91,-0.77) |
| Jamaica | male | 36.93(49.07,25.82) | 54.08(70.64,38.58) | 0.98(0.82,1.14) | 4.52(6.04,3.11) | 3.64(4.75,2.60) | -1.14(-1.35,-0.94) |
| Japan | male | 4879.84(6822.83,3268.50) | 3594.05(4982.07,2373.86) | -0.39(-0.75,-0.03) | 6.14(8.45,4.19) | 5.07(6.98,3.53) | 0.02(-0.32,0.36) |
| Jordan | male | 9.40(15.60,5.28) | 61.89(100.97,35.98) | 6.86(6.27,7.44) | 0.91(1.49,0.51) | 1.17(1.89,0.68) | 1.02(0.94,1.11) |
| Kazakhstan | male | 611.25(824.36,422.56) | 3115.43(3991.98,2249.93) | 6.82(6.36,7.29) | 8.58(11.54,5.93) | 31.79(40.78,22.88) | 5.54(5.14,5.93) |
| Kenya | male | 395.26(664.73,169.43) | 1704.86(2778.27,785.59) | 4.89(4.58,5.19) | 7.23(12.88,2.73) | 10.17(17.54,4.25) | 0.96(0.71,1.21) |
| Kiribati | male | 0.89(1.55,0.47) | 1.57(2.63,0.84) | 1.86(1.75,1.97) | 2.97(5.21,1.51) | 3.04(5.15,1.54) | 0.01(-0.14,0.16) |
| Kuwait | male | 5.17(8.45,2.87) | 32.65(53.87,18.47) | 7.21(6.95,7.47) | 0.57(0.92,0.32) | 1.06(1.70,0.62) | 2.43(2.07,2.78) |
| Kyrgyzstan | male | 200.77(277.15,137.73) | 718.25(959.52,495.14) | 4.95(4.62,5.29) | 12.41(17.14,8.24) | 22.89(30.91,15.61) | 2.58(2.37,2.79) |
| Lao People's Democratic Republic | male | 55.81(93.48,30.47) | 179.42(281.68,104.86) | 4.01(3.77,4.26) | 4.01(6.70,2.13) | 5.45(8.57,3.13) | 1.01(0.74,1.27) |
| Latvia | male | 74.43(100.55,50.02) | 107.98(140.49,75.04) | 1.87(1.46,2.28) | 5.52(7.47,3.67) | 11.18(14.66,7.85) | 3.16(2.75,3.57) |
| Lebanon | male | 11.26(18.45,6.37) | 34.16(53.85,20.32) | 4.08(3.80,4.36) | 0.87(1.40,0.51) | 1.43(2.27,0.85) | 1.96(1.73,2.20) |
| Lesotho | male | 28.69(48.63,15.84) | 46.98(76.09,26.79) | 1.12(0.61,1.63) | 4.81(8.18,2.61) | 4.93(8.04,2.71) | -0.30(-0.67,0.08) |
| Liberia | male | 15.13(25.70,7.58) | 59.63(93.63,33.78) | 4.61(4.12,5.11) | 2.32(4.00,1.14) | 3.28(5.12,1.83) | 0.72(0.38,1.07) |
| Libya | male | 13.21(21.38,7.45) | 42.18(68.88,24.44) | 4.37(3.90,4.83) | 0.99(1.60,0.55) | 1.14(1.81,0.67) | 0.59(0.37,0.80) |
| Lithuania | male | 103.41(140.08,68.68) | 196.61(255.10,134.23) | 2.78(2.03,3.54) | 5.61(7.66,3.71) | 15.31(19.99,10.38) | 4.49(3.80,5.17) |
| Luxembourg | male | 47.47(59.82,35.36) | 65.00(81.32,47.84) | 1.01(0.80,1.21) | 20.77(26.19,15.45) | 15.98(20.06,11.86) | -1.00(-1.22,-0.79) |
| North Macedonia | male | 74.09(97.66,51.99) | 145.74(187.22,104.96) | 2.49(2.41,2.57) | 7.07(9.33,4.94) | 10.52(13.51,7.56) | 1.45(1.37,1.53) |
| Madagascar | male | 83.50(141.24,41.91) | 440.94(683.21,249.19) | 6.46(5.86,7.07) | 2.36(4.14,1.06) | 4.66(7.43,2.57) | 2.83(2.30,3.38) |
| Malawi | male | 136.72(219.86,77.27) | 350.01(557.15,205.77) | 3.30(3.01,3.60) | 5.21(8.61,2.82) | 6.50(10.36,3.68) | 0.86(0.74,0.98) |
| Malaysia | male | 233.89(360.59,139.62) | 679.97(1012.20,436.46) | 3.43(2.97,3.90) | 3.31(5.03,1.96) | 3.99(5.97,2.56) | 0.42(0.20,0.64) |
| Maldives | male | 1.42(2.26,0.82) | 9.36(14.31,5.87) | 7.04(6.31,7.78) | 2.07(3.30,1.22) | 2.71(4.07,1.69) | 1.27(0.99,1.54) |
| Mali | male | 70.74(118.40,33.95) | 325.58(520.94,182.42) | 6.27(5.82,6.73) | 2.73(4.62,1.27) | 5.16(8.43,2.85) | 2.90(2.56,3.23) |
| Malta | male | 17.56(22.65,12.79) | 18.52(23.55,13.60) | 0.05(-0.16,0.25) | 8.32(10.76,6.08) | 7.38(9.38,5.40) | -0.27(-0.37,-0.17) |
| Marshall Islands | male | 0.54(0.90,0.29) | 1.07(1.73,0.59) | 2.09(1.98,2.21) | 3.45(5.70,1.78) | 3.65(5.86,2.01) | 0.11(-0.02,0.25) |
| Mauritania | male | 24.72(39.92,14.03) | 56.69(89.58,31.61) | 2.67(2.44,2.90) | 4.01(6.61,2.24) | 4.20(6.78,2.37) | -0.17(-0.41,0.08) |
| Mauritius | male | 34.83(55.77,20.01) | 33.75(53.56,18.87) | -0.55(-1.01,-0.08) | 6.44(10.20,3.65) | 4.44(6.95,2.47) | -1.49(-1.72,-1.27) |
| Mexico | male | 7449.53(10502.55,4789.68) | 13322.08(18449.77,8663.39) | 2.20(2.04,2.36) | 23.58(33.92,14.32) | 20.78(29.06,13.58) | -0.19(-0.26,-0.12) |
| Republic of Moldova | male | 949.49(1173.74,717.79) | 1015.51(1283.88,742.84) | 0.23(-0.16,0.62) | 45.50(56.84,34.25) | 45.22(57.02,33.22) | 0.23(-0.02,0.48) |
| Mongolia | male | 111.83(158.62,75.83) | 621.99(829.96,431.42) | 7.08(6.66,7.51) | 16.71(23.99,11.14) | 33.66(45.18,23.42) | 3.09(2.77,3.42) |
| Montenegro | male | 14.49(19.21,9.92) | 18.39(23.70,13.07) | 0.47(0.27,0.67) | 4.50(5.99,3.08) | 5.38(6.92,3.85) | 0.45(0.23,0.66) |
| Morocco | male | 82.66(139.41,46.12) | 278.00(448.31,157.69) | 4.37(4.09,4.66) | 1.02(1.71,0.56) | 1.46(2.35,0.84) | 1.24(0.98,1.50) |
| Mozambique | male | 74.28(124.49,36.90) | 225.75(361.92,123.15) | 3.99(3.74,4.24) | 1.94(3.27,0.96) | 2.80(4.49,1.45) | 1.29(1.03,1.55) |
| Myanmar | male | 1023.25(1727.02,535.49) | 1994.73(3194.67,1101.63) | 2.10(1.98,2.23) | 6.36(11.12,3.18) | 7.28(11.68,4.04) | 0.35(0.16,0.53) |
| Namibia | male | 20.53(33.18,11.47) | 63.10(97.94,36.60) | 3.32(3.10,3.55) | 4.77(7.97,2.62) | 6.60(10.59,3.76) | 0.64(0.43,0.86) |
| Nepal | male | 490.95(720.98,302.36) | 1315.94(1842.70,839.72) | 3.60(3.32,3.88) | 6.47(9.61,3.85) | 10.38(14.65,6.48) | 1.76(1.54,1.98) |
| Netherlands | male | 670.94(861.47,498.59) | 705.02(892.27,519.12) | 0.09(-0.25,0.44) | 7.72(9.88,5.74) | 7.17(9.13,5.30) | -0.34(-0.52,-0.16) |
| New Zealand | male | 32.19(47.34,19.94) | 50.19(72.10,33.65) | 1.41(1.17,1.66) | 1.85(2.71,1.14) | 1.95(2.71,1.35) | -0.05(-0.16,0.06) |
| Nicaragua | male | 140.91(195.46,93.87) | 475.70(661.12,315.68) | 4.58(4.41,4.75) | 11.48(15.86,7.65) | 15.25(20.92,10.13) | 1.37(1.14,1.60) |
| Niger | male | 101.99(164.97,55.00) | 322.50(516.19,176.68) | 3.75(3.65,3.84) | 4.67(7.61,2.44) | 5.61(9.15,2.93) | 0.33(0.23,0.43) |
| Nigeria | male | 1066.65(1850.54,410.96) | 2605.93(4162.20,1290.12) | 3.01(2.80,3.22) | 3.43(5.93,1.29) | 4.22(7.01,1.87) | 0.62(0.41,0.83) |
| Democratic People's Republic of Korea | male | 503.81(776.16,286.01) | 846.60(1325.27,474.93) | 1.91(1.78,2.04) | 5.08(7.92,2.90) | 5.03(7.76,2.91) | -0.16(-0.41,0.09) |
| Northern Mariana Islands | male | 1.98(3.23,1.08) | 1.32(2.19,0.69) | -1.62(-2.62,-0.61) | 6.32(10.23,3.43) | 5.15(8.03,2.92) | -1.24(-1.47,-1.02) |
| Norway | male | 161.14(217.52,110.54) | 213.87(294.17,145.75) | 1.72(1.48,1.96) | 6.87(9.22,4.71) | 6.80(9.10,4.71) | 0.85(0.58,1.13) |
| Oman | male | 10.43(17.50,5.84) | 51.67(83.38,30.28) | 5.87(5.34,6.40) | 1.54(2.61,0.85) | 2.46(3.95,1.45) | 1.61(1.30,1.92) |
| Pakistan | male | 337.83(585.64,138.43) | 751.32(1253.76,338.20) | 2.84(2.67,3.01) | 0.84(1.51,0.32) | 0.83(1.42,0.37) | 0.01(-0.15,0.17) |
| Palestine | male | 5.16(8.29,3.00) | 26.91(43.63,15.87) | 6.37(6.06,6.68) | 1.17(1.88,0.67) | 1.61(2.60,0.93) | 1.39(1.10,1.69) |
| Panama | male | 77.21(104.61,52.95) | 185.39(243.98,129.27) | 3.24(3.15,3.33) | 7.94(10.80,5.50) | 8.64(11.37,6.00) | 0.43(0.27,0.59) |
| Papua New Guinea | male | 18.91(31.19,9.97) | 58.66(94.18,31.89) | 4.05(3.99,4.11) | 1.10(1.86,0.56) | 1.25(2.02,0.68) | 0.48(0.40,0.55) |
| Paraguay | male | 119.91(153.93,87.95) | 364.37(470.64,266.69) | 3.80(3.61,3.99) | 8.08(10.49,5.84) | 10.76(14.00,7.86) | 0.96(0.72,1.20) |
| Peru | male | 839.60(1089.81,615.91) | 2517.88(3211.63,1885.03) | 3.96(3.77,4.16) | 10.82(14.23,7.94) | 14.86(18.91,11.05) | 1.18(0.99,1.37) |
| Philippines | male | 640.76(1016.20,333.94) | 2365.32(3590.94,1369.48) | 4.89(4.80,4.98) | 2.76(4.51,1.35) | 4.41(6.77,2.50) | 1.91(1.81,2.01) |
| Poland | male | 2105.84(2968.92,1403.51) | 2657.36(3719.68,1752.15) | 0.71(0.45,0.97) | 10.27(14.35,6.70) | 11.57(16.19,7.73) | 0.64(0.33,0.96) |
| Portugal | male | 1236.72(1552.71,923.96) | 779.76(991.52,563.99) | -1.76(-1.99,-1.53) | 24.39(30.61,18.22) | 12.64(15.94,9.20) | -2.48(-2.60,-2.36) |
| Puerto Rico | male | 320.67(436.82,215.89) | 258.94(350.24,177.49) | -1.31(-1.63,-0.98) | 18.90(25.65,12.72) | 13.80(18.57,9.51) | -1.64(-1.86,-1.43) |
| Qatar | male | 4.49(7.73,2.52) | 56.65(90.02,32.64) | 10.42(9.93,10.92) | 2.06(3.32,1.16) | 3.06(4.84,1.80) | 1.66(1.29,2.02) |
| Romania | male | 2672.48(3465.99,1982.92) | 3812.33(4770.34,2839.07) | 1.43(1.16,1.70) | 21.99(28.70,16.23) | 30.51(38.04,22.98) | 1.35(1.05,1.65) |
| Russian Federation | male | 5997.19(8690.70,3768.79) | 11091.37(16321.60,6569.65) | 2.01(1.89,2.12) | 7.55(10.79,4.68) | 13.51(19.69,8.19) | 2.22(2.15,2.30) |
| Rwanda | male | 100.40(162.46,51.76) | 297.44(460.72,173.32) | 4.64(4.15,5.14) | 5.20(8.73,2.41) | 6.99(10.81,4.02) | 1.19(1.06,1.32) |
| Saint Lucia | male | 4.40(5.87,2.87) | 9.63(13.03,6.57) | 2.58(2.47,2.69) | 9.50(12.88,6.17) | 8.95(11.92,6.15) | -0.50(-0.69,-0.31) |
| Saint Vincent and the Grenadines | male | 2.92(3.98,1.94) | 5.52(7.43,3.77) | 2.06(1.92,2.21) | 8.01(11.07,5.27) | 8.58(11.51,5.82) | 0.02(-0.14,0.17) |
| Samoa | male | 1.80(2.91,1.03) | 3.01(4.85,1.68) | 1.61(1.27,1.95) | 3.23(5.28,1.78) | 3.23(5.12,1.78) | -0.20(-0.35,-0.05) |
| Sao Tome and Principe | male | 2.27(3.62,1.27) | 6.06(9.41,3.54) | 3.55(3.12,3.97) | 6.78(10.81,3.76) | 7.32(11.49,4.22) | 0.05(-0.07,0.16) |
| Saudi Arabia | male | 102.06(160.25,59.19) | 480.38(770.63,283.88) | 5.32(5.22,5.41) | 1.95(3.05,1.11) | 2.54(4.03,1.52) | 1.03(0.92,1.13) |
| Senegal | male | 92.04(145.85,51.28) | 283.25(434.68,162.41) | 4.03(3.93,4.14) | 4.47(7.21,2.41) | 5.61(8.81,3.10) | 0.80(0.66,0.95) |
| Serbia | male | 481.19(620.92,339.97) | 516.18(653.58,377.79) | 0.00(-0.17,0.17) | 9.39(12.13,6.62) | 10.50(13.34,7.70) | 0.44(0.35,0.54) |
| Seychelles | male | 1.69(2.66,0.94) | 4.75(7.41,2.67) | 3.89(3.45,4.32) | 5.61(8.95,3.02) | 6.75(10.36,3.85) | 0.83(0.59,1.08) |
| Sierra Leone | male | 26.00(42.44,13.29) | 132.18(205.39,76.29) | 6.59(6.02,7.16) | 2.27(3.77,1.10) | 4.74(7.46,2.64) | 3.07(2.70,3.44) |
| Singapore | male | 40.48(61.97,24.83) | 54.89(82.97,34.46) | 0.79(0.54,1.04) | 2.41(3.71,1.47) | 1.29(1.93,0.84) | -2.50(-2.66,-2.34) |
| Slovakia | male | 519.12(682.41,354.02) | 681.78(876.81,482.60) | 1.13(0.94,1.32) | 18.24(23.85,12.81) | 19.85(25.26,14.20) | 0.68(0.47,0.88) |
| Slovenia | male | 254.59(330.80,178.70) | 257.27(325.41,188.86) | 0.20(-0.02,0.43) | 22.57(29.36,15.85) | 19.53(24.94,14.33) | -0.29(-0.48,-0.10) |
| Solomon Islands | male | 4.13(6.98,2.11) | 11.88(19.73,6.49) | 3.79(3.50,4.08) | 3.44(5.92,1.73) | 4.00(6.66,2.16) | 0.47(0.30,0.63) |
| Somalia | male | 66.98(110.63,35.81) | 190.23(310.86,107.34) | 3.82(3.63,4.00) | 3.02(5.05,1.57) | 3.22(5.32,1.75) | 0.32(0.22,0.43) |
| South Africa | male | 449.28(722.55,226.45) | 812.74(1258.35,434.87) | 1.70(1.55,1.85) | 3.42(5.80,1.53) | 3.04(4.88,1.55) | -0.57(-0.69,-0.44) |
| Republic of Korea | male | 7065.60(9244.32,5214.29) | 5055.79(6348.68,3763.24) | -2.31(-2.76,-1.85) | 30.93(40.56,22.60) | 13.73(17.25,10.33) | -3.82(-4.17,-3.48) |
| South Sudan | male | 60.48(98.04,32.89) | 99.78(167.31,53.77) | 2.01(1.85,2.17) | 3.25(5.47,1.69) | 3.37(5.59,1.77) | 0.21(0.13,0.29) |
| Spain | male | 4114.47(5016.52,3223.11) | 4209.26(5244.43,3230.54) | 0.33(0.10,0.56) | 21.09(25.83,16.63) | 13.43(16.65,10.56) | -1.68(-1.76,-1.61) |
| Sri Lanka | male | 423.80(681.17,231.14) | 783.30(1203.56,473.81) | 1.69(1.46,1.91) | 5.16(8.32,2.79) | 6.62(10.18,4.02) | 0.55(0.39,0.71) |
| Sudan | male | 27.49(45.08,13.91) | 113.35(181.84,65.38) | 4.85(4.14,5.58) | 0.50(0.84,0.25) | 0.89(1.42,0.51) | 1.92(1.28,2.57) |
| Suriname | male | 15.30(20.72,10.42) | 40.39(55.28,27.97) | 3.62(3.36,3.89) | 10.03(13.66,6.74) | 12.58(16.91,8.78) | 0.57(0.49,0.64) |
| Eswatini | male | 7.55(12.33,4.12) | 18.60(29.13,10.72) | 2.98(2.65,3.32) | 3.44(5.74,1.86) | 4.05(6.45,2.26) | 0.61(0.19,1.03) |
| Sweden | male | 371.77(509.29,250.43) | 486.47(660.25,340.58) | 1.13(0.97,1.30) | 7.54(10.11,5.24) | 8.48(11.23,6.10) | 0.62(0.42,0.81) |
| Switzerland | male | 450.41(573.44,335.21) | 500.76(633.63,371.37) | 0.49(0.25,0.73) | 10.83(13.74,8.11) | 9.16(11.45,6.79) | -0.58(-0.77,-0.39) |
| Syrian Arab Republic | male | 37.45(62.09,20.75) | 132.67(214.49,74.99) | 5.06(4.67,5.46) | 1.09(1.83,0.60) | 1.70(2.70,1.00) | 1.83(1.66,1.99) |
| Taiwan (Province of China) | male | 1016.79(1456.73,682.43) | 1023.68(1557.05,609.57) | -0.38(-0.96,0.20) | 9.73(13.76,6.43) | 6.99(10.49,4.16) | -1.29(-1.63,-0.96) |
| Tajikistan | male | 143.81(196.95,97.62) | 896.29(1173.49,641.62) | 7.21(6.73,7.70) | 8.59(11.81,5.77) | 21.35(28.45,15.16) | 3.59(3.26,3.93) |
| United Republic of Tanzania | male | 261.71(413.43,159.79) | 818.21(1271.80,480.53) | 4.02(3.91,4.14) | 3.71(5.90,2.16) | 4.57(7.13,2.59) | 0.73(0.63,0.83) |
| Thailand | male | 1101.27(1668.23,653.90) | 2564.31(4006.87,1493.97) | 3.39(2.92,3.86) | 4.19(6.45,2.43) | 6.06(9.34,3.55) | 1.66(1.43,1.89) |
| Bahamas | male | 11.91(16.14,8.03) | 20.37(27.35,14.12) | 1.50(1.29,1.71) | 11.22(15.28,7.51) | 9.30(12.47,6.53) | -1.11(-1.33,-0.90) |
| Gambia | male | 13.66(21.58,7.75) | 40.47(61.12,24.47) | 3.67(3.47,3.86) | 4.84(7.88,2.63) | 5.74(8.81,3.43) | 0.58(0.34,0.82) |
| Timor-Leste | male | 11.50(19.48,6.05) | 26.94(43.41,15.32) | 2.70(2.41,2.99) | 3.81(6.50,1.97) | 5.50(8.90,3.19) | 1.11(0.94,1.29) |
| Togo | male | 34.36(54.41,19.28) | 167.07(265.19,96.32) | 5.39(5.20,5.59) | 3.85(6.20,2.08) | 5.86(9.53,3.31) | 1.19(0.98,1.40) |
| Tonga | male | 1.27(2.07,0.69) | 1.94(3.06,1.09) | 1.57(1.21,1.93) | 4.19(6.83,2.28) | 4.59(7.27,2.57) | 0.07(-0.14,0.29) |
| Trinidad and Tobago | male | 37.23(51.10,25.12) | 54.32(72.10,37.60) | 1.10(0.99,1.21) | 7.07(9.68,4.78) | 6.52(8.58,4.50) | -0.40(-0.54,-0.25) |
| Tunisia | male | 43.72(69.34,24.75) | 138.16(217.58,82.42) | 4.19(4.11,4.27) | 1.51(2.40,0.86) | 2.03(3.18,1.22) | 1.03(0.96,1.10) |
| Turkey | male | 151.40(244.90,87.82) | 688.81(1087.64,398.70) | 6.25(5.84,6.65) | 0.68(1.11,0.39) | 1.43(2.25,0.83) | 3.42(3.03,3.81) |
| Turkmenistan | male | 154.70(210.46,105.54) | 562.87(770.40,371.78) | 4.99(4.83,5.14) | 12.64(17.47,8.47) | 20.03(27.35,13.23) | 1.87(1.63,2.11) |
| Uganda | male | 209.32(327.19,121.89) | 598.56(908.43,342.32) | 3.72(3.52,3.92) | 4.77(7.57,2.60) | 5.66(8.77,3.14) | 0.58(0.48,0.69) |
| Ukraine | male | 2226.12(3274.30,1385.35) | 3431.99(5595.41,1698.22) | 1.88(1.75,2.00) | 8.48(12.42,5.27) | 14.14(22.73,7.36) | 2.30(2.11,2.49) |
| United Arab Emirates | male | 9.90(16.83,5.31) | 132.05(223.88,73.29) | 9.02(8.74,9.29) | 1.14(1.91,0.61) | 1.36(2.21,0.78) | 0.38(0.15,0.62) |
| United Kingdom | male | 2514.78(3309.38,1790.89) | 5475.75(6808.15,4244.76) | 2.34(1.88,2.80) | 8.24(10.84,5.88) | 15.57(19.32,12.20) | 1.81(1.42,2.21) |
| United States of America | male | 11365.76(15526.60,7884.19) | 11616.64(15892.42,8278.52) | 0.03(-0.22,0.27) | 8.33(11.36,5.68) | 6.44(8.60,4.68) | -0.81(-0.93,-0.69) |
| Uruguay | male | 114.61(161.77,72.59) | 139.55(192.83,91.83) | 0.62(0.47,0.78) | 7.55(10.65,4.80) | 7.49(10.50,4.95) | -0.15(-0.29,-0.02) |
| Uzbekistan | male | 890.04(1186.38,623.36) | 4596.98(5881.79,3325.96) | 6.20(5.95,6.44) | 13.35(18.06,9.22) | 27.55(35.29,19.96) | 2.65(2.50,2.80) |
| Vanuatu | male | 2.79(4.54,1.51) | 6.34(10.35,3.33) | 2.94(2.77,3.10) | 4.85(7.91,2.56) | 5.06(8.34,2.63) | 0.11(0.01,0.21) |
| Venezuela (Bolivarian Republic of) | male | 837.19(1154.01,578.14) | 1469.95(2026.20,983.55) | 2.09(1.88,2.31) | 11.45(15.56,7.70) | 9.46(12.98,6.33) | -0.67(-0.71,-0.62) |
| Viet nam | male | 1162.99(1902.65,666.88) | 4626.77(7089.70,2802.80) | 4.99(4.75,5.22) | 5.56(9.33,3.09) | 7.96(12.14,4.82) | 1.30(0.88,1.72) |
| Virginia | male | 296.19(406.56,202.04) | 303.26(421.28,211.34) | 0.01(-0.18,0.20) | 8.30(11.48,5.62) | 6.13(8.31,4.39) | -1.00(-1.10,-0.90) |
| Yemen | male | 30.39(49.78,16.89) | 133.59(224.02,75.84) | 5.39(4.96,5.82) | 0.91(1.47,0.49) | 1.42(2.37,0.79) | 1.72(1.40,2.05) |
| Zambia | male | 122.84(197.21,69.63) | 394.96(615.07,233.56) | 4.00(3.56,4.44) | 5.91(9.64,3.19) | 6.80(10.65,3.85) | 0.34(0.12,0.55) |
| Zimbabwe | male | 114.92(182.23,68.87) | 233.16(381.57,135.43) | 1.90(1.17,2.63) | 4.11(6.55,2.42) | 4.60(7.52,2.57) | 0.04(-0.58,0.66) |
| Monaco | male | 1.90(2.48,1.35) | 2.25(2.96,1.61) | 0.88(0.64,1.12) | 10.91(14.20,7.77) | 11.84(15.20,8.41) | 0.40(0.31,0.49) |
| San Marino | male | 2.30(2.93,1.66) | 2.94(3.74,2.14) | 1.01(0.85,1.17) | 17.82(22.68,12.83) | 16.34(20.50,11.92) | -0.29(-0.33,-0.24) |
| Saint Kitts and Nevis | male | 2.05(2.84,1.34) | 5.96(8.07,4.15) | 3.66(3.42,3.90) | 15.26(20.95,9.84) | 15.33(20.55,10.83) | -0.20(-0.38,-0.02) |
| Cook Islands | male | 0.08(0.13,0.05) | 0.12(0.19,0.07) | 1.42(1.01,1.83) | 1.03(1.61,0.60) | 1.34(2.06,0.81) | 0.99(0.84,1.14) |
| Nauru | male | 0.14(0.22,0.07) | 0.18(0.29,0.10) | 0.74(0.58,0.89) | 2.97(4.96,1.55) | 3.65(5.81,1.99) | 0.57(0.36,0.77) |
| Niue | male | 0.03(0.04,0.01) | 0.03(0.04,0.02) | -0.06(-0.36,0.23) | 2.63(4.25,1.45) | 3.22(5.05,1.85) | 0.55(0.27,0.82) |
| Palau | male | 0.21(0.34,0.12) | 0.44(0.71,0.23) | 1.84(0.90,2.79) | 2.73(4.42,1.48) | 3.24(5.22,1.80) | 0.67(0.45,0.90) |
| Tokelau | male | 0.01(0.02,0.01) | 0.01(0.02,0.01) | 0.17(-0.12,0.46) | 2.02(3.29,1.09) | 2.04(3.29,1.15) | -0.08(-0.15,-0.02) |
| Tuvalu | male | 0.08(0.14,0.04) | 0.15(0.24,0.09) | 2.03(1.96,2.10) | 2.27(3.77,1.22) | 2.63(4.15,1.44) | 0.42(0.36,0.49) |
| Afghanistan | female | 29.00(49.28,14.83) | 78.03(127.12,42.54) | 3.07(2.59,3.55) | 0.72(1.22,0.37) | 0.86(1.38,0.48) | 0.36(0.12,0.60) |
| Albania | female | 63.37(84.87,44.64) | 88.97(119.58,61.54) | 1.37(1.13,1.60) | 5.16(6.97,3.60) | 5.33(7.12,3.80) | 0.18(0.08,0.28) |
| Algeria | female | 50.42(80.81,28.58) | 169.32(268.25,101.18) | 3.95(3.62,4.28) | 0.71(1.12,0.40) | 0.88(1.36,0.52) | 0.39(0.12,0.66) |
| American Samoa | female | 0.13(0.21,0.08) | 0.25(0.41,0.15) | 2.03(1.79,2.26) | 0.83(1.32,0.49) | 0.90(1.45,0.55) | 0.10(0.02,0.19) |
| Andorra | female | 2.35(3.12,1.64) | 4.00(5.35,2.77) | 1.68(1.24,2.12) | 8.00(10.71,5.55) | 6.93(9.16,4.79) | -0.61(-0.70,-0.52) |
| Angola | female | 52.58(92.33,22.71) | 223.98(375.10,119.13) | 5.12(4.91,5.33) | 1.80(3.23,0.73) | 2.35(3.89,1.23) | 0.83(0.65,1.01) |
| Antigua and Barbuda | female | 0.83(1.17,0.54) | 2.57(3.58,1.73) | 4.12(3.87,4.37) | 3.32(4.77,2.16) | 4.27(5.88,2.91) | 0.86(0.80,0.92) |
| Argentina | female | 473.60(687.96,300.69) | 990.95(1426.38,633.34) | 2.48(2.30,2.66) | 2.94(4.26,1.87) | 3.99(5.74,2.55) | 0.96(0.81,1.12) |
| Armenia | female | 72.78(96.78,50.49) | 257.72(349.55,173.24) | 5.37(5.07,5.67) | 4.20(5.69,2.87) | 11.37(15.01,8.02) | 4.17(3.94,4.40) |
| Australia | female | 91.32(139.93,55.54) | 153.71(238.15,92.93) | 1.93(1.74,2.11) | 1.01(1.55,0.61) | 1.06(1.64,0.64) | 0.34(0.11,0.56) |
| Austria | female | 396.86(512.92,279.42) | 425.56(546.77,304.43) | 0.48(0.08,0.88) | 9.62(12.49,6.73) | 8.23(10.56,5.92) | -0.49(-0.71,-0.26) |
| Azerbaijan | female | 268.59(374.52,184.35) | 1110.12(1519.27,741.39) | 5.62(5.35,5.90) | 8.40(11.71,5.77) | 15.96(21.55,10.92) | 2.38(2.17,2.60) |
| Bahrain | female | 1.13(1.82,0.65) | 7.24(11.61,4.23) | 7.16(6.88,7.43) | 1.03(1.70,0.57) | 1.27(2.04,0.76) | 0.77(0.55,0.99) |
| Bangladesh | female | 536.08(868.60,310.66) | 2555.70(3891.35,1521.82) | 5.86(5.74,5.98) | 1.79(2.87,1.02) | 3.33(5.08,1.99) | 2.35(2.26,2.44) |
| Barbados | female | 2.65(3.70,1.77) | 4.90(6.87,3.31) | 2.20(2.12,2.27) | 2.20(3.06,1.44) | 2.34(3.25,1.59) | 0.11(0.03,0.20) |
| Belarus | female | 122.90(180.40,77.18) | 289.63(413.77,182.05) | 3.75(3.19,4.31) | 2.21(3.25,1.39) | 5.19(7.35,3.29) | 3.95(3.44,4.46) |
| Belgium | female | 467.27(581.06,351.58) | 534.77(683.98,377.22) | 0.44(0.23,0.64) | 8.75(10.93,6.60) | 8.54(10.96,6.03) | -0.15(-0.23,-0.06) |
| Belize | female | 2.27(3.13,1.52) | 10.16(14.18,6.75) | 5.58(5.42,5.74) | 4.53(6.40,2.97) | 5.39(7.59,3.57) | 0.64(0.54,0.74) |
| Benin | female | 31.95(50.51,16.66) | 85.76(131.54,51.65) | 3.07(2.76,3.38) | 2.58(4.22,1.30) | 2.47(3.80,1.44) | -0.55(-0.78,-0.32) |
| Bermuda | female | 1.18(1.60,0.80) | 1.06(1.47,0.71) | -0.83(-1.04,-0.63) | 3.37(4.59,2.29) | 2.20(3.03,1.51) | -2.04(-2.25,-1.83) |
| Bhutan | female | 4.71(7.47,2.73) | 12.99(19.42,7.95) | 3.86(3.60,4.13) | 2.81(4.57,1.61) | 4.01(5.99,2.43) | 1.43(1.24,1.62) |
| Bolivia (Plurinational State of) | female | 123.28(181.38,78.06) | 491.53(695.69,332.33) | 4.97(4.88,5.07) | 5.85(8.63,3.67) | 9.52(13.43,6.30) | 1.72(1.66,1.78) |
| Bosnia and Herzegovina | female | 126.28(166.65,87.49) | 94.93(124.93,67.88) | -0.71(-0.82,-0.60) | 5.12(6.76,3.55) | 4.37(5.72,3.12) | -0.56(-0.75,-0.37) |
| Botswana | female | 5.90(9.64,3.17) | 17.71(28.19,10.25) | 4.46(4.14,4.78) | 1.39(2.29,0.71) | 1.58(2.56,0.89) | 1.13(0.81,1.44) |
| Brazil | female | 1880.65(2639.53,1218.77) | 3542.95(5050.60,2286.19) | 2.09(2.02,2.17) | 3.12(4.53,1.96) | 2.70(3.82,1.75) | -0.61(-0.73,-0.50) |
| Brunei Darussalam | female | 3.42(5.17,2.09) | 8.05(11.95,4.94) | 2.35(1.99,2.71) | 3.72(5.67,2.28) | 3.15(4.64,1.92) | -1.15(-1.32,-0.97) |
| Bulgaria | female | 247.73(321.63,180.85) | 216.52(280.51,155.97) | -0.40(-0.50,-0.29) | 4.80(6.27,3.52) | 5.24(6.78,3.79) | 0.43(0.32,0.55) |
| Burkina Faso | female | 65.64(100.15,38.01) | 154.89(247.59,84.38) | 2.66(2.38,2.94) | 2.38(3.65,1.37) | 2.35(3.83,1.24) | -0.48(-0.74,-0.22) |
| Burundi | female | 44.03(68.01,24.86) | 103.69(159.45,59.14) | 3.07(2.74,3.40) | 2.88(4.59,1.57) | 3.47(5.53,1.93) | 0.68(0.56,0.81) |
| Cambodia | female | 184.84(299.55,104.34) | 623.27(979.66,375.38) | 4.32(4.11,4.54) | 5.33(8.79,2.97) | 7.77(12.20,4.68) | 1.27(1.04,1.51) |
| Cameroon | female | 69.44(108.17,39.42) | 198.56(308.72,110.95) | 3.57(3.34,3.80) | 2.35(3.64,1.32) | 2.36(3.70,1.30) | -0.15(-0.31,0.02) |
| Canada | female | 607.28(887.50,380.42) | 831.28(1233.97,514.35) | 1.04(0.69,1.39) | 3.98(5.82,2.50) | 4.01(5.87,2.49) | 0.03(-0.04,0.10) |
| Cabo Verde | female | 1.91(3.09,0.98) | 5.53(8.50,3.41) | 3.81(3.22,4.40) | 1.61(2.58,0.83) | 2.17(3.31,1.33) | 0.86(0.41,1.30) |
| Central African Republic | female | 15.75(27.66,8.16) | 34.68(58.59,18.54) | 2.58(2.46,2.70) | 1.76(3.07,0.87) | 1.90(3.20,0.99) | 0.02(-0.09,0.14) |
| Chad | female | 45.33(72.38,24.16) | 92.72(144.72,54.78) | 2.17(2.00,2.35) | 2.69(4.37,1.42) | 2.44(3.83,1.39) | -0.60(-0.68,-0.52) |
| Chile | female | 366.54(525.71,227.63) | 822.34(1179.79,522.47) | 2.97(2.66,3.29) | 6.02(8.69,3.72) | 7.19(10.21,4.54) | 0.57(0.36,0.78) |
| China | female | 8208.92(12121.14,5077.42) | 16946.21(24588.07,11316.20) | 2.83(2.67,2.99) | 1.66(2.50,1.01) | 1.64(2.32,1.11) | 0.11(-0.12,0.33) |
| Colombia | female | 617.84(821.37,429.02) | 1259.27(1695.08,860.68) | 2.44(2.29,2.60) | 5.20(6.88,3.60) | 4.50(6.07,3.07) | -0.64(-0.70,-0.57) |
| Comoros | female | 3.47(5.61,1.87) | 9.84(15.21,5.61) | 3.63(3.47,3.79) | 2.60(4.35,1.36) | 3.31(5.15,1.86) | 0.84(0.69,0.99) |
| Congo | female | 10.65(17.92,5.50) | 41.78(67.83,22.17) | 4.94(4.69,5.19) | 1.43(2.39,0.71) | 2.01(3.23,1.08) | 1.26(1.08,1.43) |
| Costa Rica | female | 88.58(119.53,61.35) | 260.18(349.08,181.75) | 3.84(3.60,4.09) | 8.14(11.06,5.64) | 9.27(12.42,6.49) | 0.42(0.37,0.48) |
| Côte d'Ivoire | female | 64.03(101.20,35.99) | 179.65(276.74,104.54) | 3.41(3.22,3.60) | 2.31(3.57,1.27) | 2.41(3.76,1.41) | 0.08(-0.14,0.29) |
| Croatia | female | 213.14(282.53,149.81) | 153.95(198.58,112.89) | -1.12(-1.28,-0.96) | 7.82(10.42,5.48) | 6.04(7.78,4.47) | -0.83(-0.95,-0.70) |
| Cuba | female | 176.03(244.80,120.46) | 258.41(369.82,169.33) | 1.37(1.29,1.46) | 3.39(4.70,2.32) | 3.14(4.34,2.12) | -0.35(-0.44,-0.27) |
| Cyprus | female | 26.25(35.34,18.09) | 47.85(63.79,33.42) | 1.91(1.75,2.07) | 6.62(8.87,4.55) | 5.57(7.36,3.91) | -0.83(-0.98,-0.67) |
| Czechia | female | 265.95(344.02,191.66) | 332.56(438.92,236.73) | 0.83(0.59,1.08) | 4.48(5.74,3.28) | 5.17(6.77,3.70) | 0.63(0.50,0.76) |
| Democratic Republic of the Congo | female | 209.81(361.68,105.13) | 565.52(916.97,321.05) | 3.22(3.00,3.45) | 1.79(3.10,0.87) | 2.03(3.35,1.12) | 0.15(-0.05,0.34) |
| Denmark | female | 124.88(170.77,82.65) | 186.36(249.02,125.35) | 1.65(1.02,2.30) | 4.42(6.01,2.96) | 6.20(8.18,4.30) | 1.30(0.74,1.87) |
| Djibouti | female | 3.57(5.59,2.06) | 14.60(22.28,8.68) | 4.80(4.58,5.03) | 3.25(5.12,1.88) | 3.41(5.19,2.03) | 0.01(-0.16,0.18) |
| Dominica | female | 1.01(1.44,0.65) | 1.34(1.89,0.88) | 0.78(0.67,0.89) | 3.49(4.98,2.23) | 3.42(4.82,2.26) | -0.38(-0.50,-0.25) |
| Dominican Republic | female | 132.39(191.88,87.98) | 363.86(510.54,238.27) | 3.36(3.28,3.44) | 5.51(8.00,3.60) | 6.91(9.69,4.50) | 0.65(0.58,0.73) |
| Ecuador | female | 158.41(217.88,106.24) | 1078.05(1430.21,758.63) | 6.79(6.41,7.17) | 4.72(6.57,3.21) | 12.88(17.07,9.04) | 3.51(3.18,3.84) |
| Egypt | female | 312.09(504.01,177.70) | 1085.19(1771.03,610.79) | 4.53(4.40,4.65) | 1.86(3.04,1.03) | 3.00(4.90,1.70) | 1.81(1.67,1.95) |
| El Salvador | female | 129.18(179.28,86.91) | 332.39(459.18,229.95) | 3.47(3.40,3.55) | 7.03(9.78,4.71) | 9.85(13.61,6.81) | 1.31(1.23,1.38) |
| Equatorial Guinea | female | 2.21(3.71,1.12) | 7.60(11.82,4.49) | 4.55(4.41,4.69) | 1.54(2.58,0.77) | 1.83(2.85,1.06) | 0.76(0.66,0.87) |
| Eritrea | female | 25.05(41.04,14.30) | 89.95(139.88,54.90) | 4.51(4.36,4.67) | 3.00(4.88,1.68) | 4.22(6.59,2.54) | 1.12(0.91,1.34) |
| Estonia | female | 15.96(23.98,9.60) | 19.77(28.68,12.02) | 0.73(0.19,1.28) | 1.87(2.78,1.13) | 2.88(4.14,1.79) | 1.59(1.02,2.15) |
| Ethiopia | female | 360.19(604.50,155.32) | 863.05(1327.42,479.27) | 2.91(2.76,3.06) | 2.80(4.83,1.12) | 3.28(5.31,1.69) | 0.58(0.48,0.68) |
| Micronesia (Federated States of) | female | 0.32(0.52,0.18) | 0.60(0.95,0.33) | 2.08(1.86,2.31) | 1.07(1.76,0.59) | 1.25(1.98,0.71) | 0.41(0.31,0.52) |
| Fiji | female | 2.16(3.51,1.23) | 4.08(6.43,2.36) | 2.24(2.08,2.40) | 0.76(1.25,0.42) | 0.88(1.37,0.51) | 0.44(0.38,0.49) |
| Finland | female | 105.43(144.15,72.29) | 135.20(182.82,91.54) | 1.04(0.57,1.52) | 3.56(4.85,2.45) | 5.12(6.89,3.51) | 1.65(1.16,2.14) |
| France | female | 2506.78(3280.24,1670.13) | 1974.35(2643.12,1356.20) | -1.10(-1.25,-0.96) | 8.07(10.54,5.38) | 5.57(7.42,3.82) | -1.51(-1.58,-1.43) |
| Gabon | female | 5.06(8.39,2.41) | 14.31(22.94,7.82) | 3.64(3.37,3.90) | 1.56(2.61,0.74) | 1.94(3.08,1.06) | 0.65(0.49,0.81) |
| Georgia | female | 199.62(271.11,135.02) | 143.95(189.55,101.79) | -1.16(-1.21,-1.11) | 6.05(8.13,4.13) | 5.76(7.49,4.14) | 0.12(-1.51,1.78) |
| Germany | female | 3973.71(5109.90,2831.26) | 4192.32(5535.08,2890.99) | 0.19(-0.24,0.62) | 9.23(11.92,6.52) | 9.31(12.17,6.56) | 0.00(-0.20,0.21) |
| Ghana | female | 106.43(167.71,60.98) | 278.31(426.86,159.89) | 3.02(2.77,3.28) | 2.55(4.01,1.42) | 2.39(3.70,1.32) | -0.57(-0.77,-0.37) |
| Greece | female | 404.73(535.28,284.03) | 265.76(360.09,185.76) | -1.69(-1.81,-1.56) | 6.73(9.03,4.67) | 4.21(5.61,2.94) | -1.94(-2.09,-1.79) |
| Greenland | female | 0.87(1.32,0.52) | 0.94(1.41,0.56) | 0.33(-0.21,0.86) | 3.23(4.97,1.90) | 3.54(5.38,2.12) | 0.49(0.35,0.63) |
| Grenada | female | 1.05(1.48,0.68) | 1.86(2.60,1.23) | 1.90(1.66,2.13) | 3.54(5.08,2.29) | 3.11(4.31,2.11) | -0.62(-0.78,-0.47) |
| Guam | female | 0.72(1.13,0.43) | 0.97(1.51,0.56) | 0.49(0.15,0.84) | 1.40(2.19,0.83) | 1.08(1.68,0.64) | -1.43(-1.59,-1.26) |
| Guatemala | female | 253.78(360.54,167.59) | 955.14(1303.81,650.33) | 4.78(4.68,4.88) | 9.69(13.83,6.27) | 12.90(17.75,8.74) | 1.06(1.00,1.13) |
| Guinea | female | 46.70(74.67,24.10) | 80.56(126.06,45.98) | 1.22(0.94,1.50) | 2.48(3.96,1.27) | 2.27(3.60,1.26) | -0.75(-0.96,-0.55) |
| Guinea-Bissau | female | 6.68(10.98,3.55) | 12.65(20.01,7.25) | 1.70(1.44,1.96) | 2.39(3.93,1.24) | 2.19(3.51,1.24) | -0.76(-0.94,-0.58) |
| Guyana | female | 13.34(18.92,8.61) | 20.62(29.71,13.38) | 1.48(1.35,1.61) | 4.98(7.15,3.23) | 5.23(7.53,3.40) | 0.14(-0.11,0.39) |
| Haiti | female | 76.53(117.16,45.44) | 250.08(371.27,156.70) | 4.13(4.05,4.21) | 3.47(5.24,2.06) | 4.78(7.08,2.98) | 1.11(1.03,1.18) |
| Honduras | female | 144.18(204.51,96.18) | 426.58(605.58,274.74) | 3.76(3.63,3.89) | 10.31(14.58,6.88) | 10.17(14.42,6.60) | -0.15(-0.29,-0.02) |
| Hungary | female | 512.00(695.06,332.06) | 318.97(410.84,235.98) | -1.89(-2.00,-1.77) | 8.85(11.96,5.78) | 5.28(6.73,3.91) | -2.10(-2.39,-1.82) |
| Iceland | female | 2.42(3.36,1.60) | 3.60(5.03,2.36) | 1.54(1.26,1.82) | 1.90(2.66,1.26) | 2.01(2.80,1.31) | 0.42(0.26,0.57) |
| India | female | 6143.52(10057.60,2861.22) | 21706.44(34030.73,11279.43) | 4.73(4.50,4.96) | 2.09(3.46,0.94) | 3.28(5.19,1.67) | 1.76(1.56,1.96) |
| Indonesia | female | 2111.51(3591.99,922.24) | 3546.21(5557.33,1845.19) | 1.39(1.13,1.66) | 3.19(5.58,1.29) | 2.62(4.16,1.34) | -1.09(-1.36,-0.83) |
| Iran (Islamic Republic of) | female | 58.69(91.88,32.11) | 275.11(403.00,169.96) | 5.37(5.25,5.50) | 0.38(0.60,0.21) | 0.65(0.97,0.40) | 1.54(1.41,1.68) |
| Iraq | female | 25.46(41.45,14.37) | 111.64(178.45,65.52) | 5.33(5.15,5.50) | 0.56(0.91,0.32) | 0.75(1.21,0.44) | 1.07(0.91,1.22) |
| Ireland | female | 58.47(78.89,40.66) | 129.98(174.83,88.25) | 2.70(2.34,3.06) | 3.41(4.56,2.37) | 4.47(5.96,3.05) | 0.67(0.40,0.94) |
| Israel | female | 119.24(160.44,82.33) | 246.37(334.34,169.04) | 2.21(1.85,2.58) | 5.42(7.34,3.71) | 5.27(7.20,3.63) | -0.32(-0.48,-0.15) |
| Italy | female | 2508.46(3375.18,1770.16) | 2177.78(2992.79,1512.95) | -0.37(-0.58,-0.16) | 7.51(10.08,5.39) | 4.94(6.64,3.49) | -1.53(-1.73,-1.33) |
| Jamaica | female | 12.90(18.07,8.40) | 23.84(33.54,15.69) | 2.09(2.02,2.16) | 1.53(2.17,0.98) | 1.57(2.20,1.03) | -0.01(-0.13,0.12) |
| Japan | female | 2981.06(4135.07,2019.66) | 2036.66(2889.71,1371.74) | -0.59(-0.93,-0.25) | 3.59(4.94,2.45) | 2.74(3.81,1.89) | 0.00(-0.40,0.40) |
| Jordan | female | 5.70(9.30,3.27) | 33.16(54.16,18.99) | 6.24(5.90,6.58) | 0.66(1.07,0.37) | 0.82(1.31,0.47) | 0.81(0.71,0.92) |
| Kazakhstan | female | 410.51(555.12,285.83) | 1826.38(2398.90,1314.52) | 6.29(5.66,6.93) | 5.34(7.24,3.71) | 17.00(22.30,12.22) | 4.86(4.33,5.39) |
| Kenya | female | 315.52(512.43,152.95) | 982.36(1527.49,522.00) | 3.63(3.32,3.93) | 5.94(10.01,2.71) | 6.22(10.04,3.16) | -0.11(-0.35,0.14) |
| Kiribati | female | 0.30(0.50,0.15) | 0.67(1.09,0.36) | 2.99(2.82,3.17) | 1.08(1.80,0.54) | 1.26(2.08,0.68) | 0.52(0.32,0.72) |
| Kuwait | female | 1.29(2.11,0.74) | 11.95(19.41,6.87) | 8.68(8.36,8.99) | 0.31(0.50,0.17) | 0.58(0.95,0.34) | 2.53(2.14,2.91) |
| Kyrgyzstan | female | 133.51(182.34,92.35) | 345.92(458.27,241.64) | 3.92(3.66,4.19) | 8.09(11.16,5.49) | 10.79(14.39,7.51) | 1.14(0.95,1.33) |
| Lao People's Democratic Republic | female | 30.69(48.14,17.61) | 98.10(149.28,59.70) | 4.06(3.79,4.33) | 2.22(3.51,1.27) | 3.25(4.99,1.99) | 1.27(1.07,1.48) |
| Latvia | female | 31.32(45.16,19.17) | 30.53(45.21,18.31) | 0.23(-0.10,0.56) | 2.14(3.09,1.30) | 3.07(4.52,1.89) | 1.72(1.42,2.03) |
| Lebanon | female | 7.82(12.51,4.46) | 24.96(38.55,14.99) | 4.17(3.96,4.37) | 0.60(0.96,0.34) | 0.89(1.37,0.53) | 1.48(1.29,1.67) |
| Lesotho | female | 8.26(14.39,3.80) | 10.98(18.29,5.48) | 0.38(-0.05,0.80) | 1.35(2.39,0.59) | 1.21(2.03,0.58) | -0.91(-1.32,-0.49) |
| Liberia | female | 13.68(22.14,6.81) | 37.38(59.11,21.47) | 3.20(2.92,3.47) | 2.39(3.95,1.16) | 2.60(4.17,1.48) | -0.05(-0.20,0.10) |
| Libya | female | 6.67(10.77,3.81) | 24.26(39.04,14.30) | 4.83(4.37,5.28) | 0.68(1.07,0.38) | 0.77(1.22,0.45) | 0.57(0.31,0.83) |
| Lithuania | female | 37.24(55.18,22.80) | 57.41(82.63,33.80) | 2.09(1.21,2.96) | 1.92(2.85,1.19) | 4.26(6.13,2.56) | 3.54(2.90,4.19) |
| Luxembourg | female | 21.90(28.79,15.25) | 28.78(37.96,19.54) | 0.92(0.55,1.28) | 10.17(13.38,7.04) | 7.62(10.03,5.17) | -1.04(-1.36,-0.71) |
| North Macedonia | female | 32.11(43.54,22.29) | 52.11(68.73,36.38) | 1.74(1.63,1.86) | 3.05(4.14,2.09) | 3.84(5.03,2.69) | 0.74(0.67,0.82) |
| Madagascar | female | 96.55(158.10,50.27) | 305.79(475.47,181.74) | 4.21(3.87,4.55) | 3.00(4.92,1.48) | 3.59(5.55,2.07) | 0.69(0.46,0.92) |
| Malawi | female | 98.96(151.67,58.78) | 178.92(264.76,108.11) | 2.02(1.70,2.34) | 3.97(6.13,2.29) | 3.71(5.58,2.18) | -0.24(-0.34,-0.14) |
| Malaysia | female | 119.41(179.45,72.94) | 437.51(637.81,282.74) | 4.42(3.97,4.87) | 1.96(3.00,1.19) | 2.86(4.15,1.85) | 1.06(0.72,1.39) |
| Maldives | female | 0.87(1.37,0.52) | 3.75(5.67,2.39) | 5.49(5.14,5.85) | 1.54(2.46,0.90) | 2.01(3.01,1.28) | 1.13(0.89,1.38) |
| Mali | female | 58.58(97.63,28.89) | 137.16(214.64,78.47) | 2.92(2.71,3.14) | 2.29(3.83,1.09) | 2.43(3.87,1.34) | 0.10(-0.01,0.20) |
| Malta | female | 8.81(11.82,5.95) | 7.68(10.33,5.25) | -0.68(-0.85,-0.51) | 4.05(5.42,2.74) | 3.27(4.41,2.24) | -0.73(-0.81,-0.65) |
| Marshall Islands | female | 0.12(0.20,0.07) | 0.29(0.47,0.16) | 3.11(2.95,3.26) | 1.00(1.67,0.55) | 1.13(1.81,0.63) | 0.46(0.15,0.77) |
| Mauritania | female | 13.45(21.35,7.66) | 28.86(44.83,17.05) | 2.49(2.29,2.69) | 2.26(3.67,1.26) | 2.18(3.43,1.26) | -0.42(-0.57,-0.26) |
| Mauritius | female | 7.59(11.46,4.64) | 12.48(18.76,7.79) | 1.29(1.05,1.53) | 1.55(2.40,0.94) | 1.48(2.19,0.93) | -0.56(-0.72,-0.41) |
| Mexico | female | 3723.49(5071.28,2521.30) | 10784.69(14713.62,7653.89) | 4.01(3.92,4.10) | 12.51(17.33,8.32) | 15.36(20.92,10.87) | 0.91(0.85,0.96) |
| Republic of Moldova | female | 796.38(1057.14,543.20) | 592.10(790.88,409.93) | -0.84(-1.42,-0.25) | 34.49(45.99,23.17) | 25.56(34.37,17.62) | -0.94(-1.22,-0.67) |
| Mongolia | female | 81.66(115.67,52.71) | 520.76(711.54,347.92) | 7.58(7.18,7.98) | 12.96(18.51,8.25) | 27.42(37.39,18.24) | 3.27(2.96,3.59) |
| Montenegro | female | 4.66(6.42,3.09) | 6.27(8.37,4.30) | 0.87(0.67,1.08) | 1.45(1.99,0.97) | 1.74(2.31,1.21) | 0.51(0.24,0.77) |
| Morocco | female | 46.42(75.28,25.53) | 143.20(231.22,82.42) | 3.98(3.66,4.30) | 0.58(0.96,0.32) | 0.77(1.23,0.44) | 0.94(0.66,1.23) |
| Mozambique | female | 95.47(148.39,56.60) | 203.12(313.26,117.71) | 2.58(2.20,2.95) | 2.39(3.71,1.36) | 2.62(4.08,1.48) | 0.28(-0.02,0.58) |
| Myanmar | female | 309.50(502.79,174.37) | 857.21(1366.27,488.55) | 3.63(3.51,3.75) | 1.99(3.25,1.11) | 2.79(4.45,1.59) | 1.16(0.97,1.35) |
| Namibia | female | 5.25(8.78,2.82) | 15.47(24.69,8.50) | 3.27(3.07,3.47) | 1.16(1.95,0.60) | 1.54(2.47,0.83) | 0.63(0.45,0.82) |
| Nepal | female | 266.31(380.91,171.16) | 1122.73(1514.36,774.56) | 5.67(5.46,5.89) | 4.17(6.00,2.62) | 7.92(10.72,5.46) | 2.67(2.49,2.85) |
| Netherlands | female | 431.10(582.04,295.58) | 453.71(602.01,309.26) | 0.02(-0.36,0.39) | 5.12(6.90,3.51) | 4.65(6.23,3.17) | -0.51(-0.71,-0.30) |
| New Zealand | female | 16.08(23.28,9.89) | 23.10(33.09,14.85) | 1.25(1.04,1.46) | 0.90(1.32,0.56) | 0.84(1.17,0.56) | -0.36(-0.43,-0.29) |
| Nicaragua | female | 66.91(92.23,45.69) | 262.29(358.59,179.86) | 5.25(5.07,5.44) | 6.07(8.47,4.12) | 8.62(11.90,5.93) | 1.62(1.38,1.85) |
| Niger | female | 52.43(87.48,26.39) | 148.78(241.02,81.25) | 3.33(3.19,3.48) | 2.81(4.71,1.37) | 2.68(4.36,1.42) | -0.50(-0.63,-0.37) |
| Nigeria | female | 618.76(1019.41,285.67) | 2119.56(3337.78,1085.74) | 4.50(4.30,4.70) | 2.73(4.60,1.19) | 3.32(5.35,1.67) | 0.70(0.63,0.76) |
| Democratic People's Republic of Korea | female | 182.44(290.87,104.25) | 276.86(446.25,156.94) | 1.35(1.01,1.70) | 1.63(2.57,0.95) | 1.59(2.53,0.93) | -0.24(-0.54,0.06) |
| Northern Mariana Islands | female | 0.32(0.52,0.19) | 0.69(1.15,0.38) | 2.45(1.78,3.12) | 2.02(3.23,1.16) | 2.19(3.44,1.34) | -0.05(-0.18,0.09) |
| Norway | female | 85.84(120.04,56.50) | 112.90(158.80,72.90) | 2.30(1.83,2.78) | 3.83(5.33,2.53) | 3.80(5.25,2.51) | 1.26(0.83,1.69) |
| Oman | female | 3.30(5.43,1.88) | 13.46(21.32,7.93) | 4.82(4.66,4.98) | 0.89(1.47,0.49) | 1.26(2.01,0.73) | 1.10(0.84,1.36) |
| Pakistan | female | 707.10(1194.86,313.67) | 1451.83(2353.82,713.80) | 2.56(2.41,2.71) | 2.26(3.97,0.96) | 2.01(3.39,0.95) | -0.37(-0.44,-0.30) |
| Palestine | female | 3.66(5.96,2.10) | 14.71(24.42,8.49) | 5.25(4.84,5.66) | 0.68(1.11,0.38) | 0.94(1.55,0.55) | 1.39(1.08,1.70) |
| Panama | female | 48.91(67.21,33.27) | 140.95(188.29,97.96) | 3.84(3.73,3.94) | 5.62(7.75,3.87) | 6.57(8.76,4.59) | 0.61(0.46,0.75) |
| Papua New Guinea | female | 1.83(3.09,0.92) | 5.93(10.10,3.03) | 4.17(3.92,4.42) | 0.13(0.23,0.07) | 0.15(0.26,0.07) | 0.35(0.11,0.58) |
| Paraguay | female | 26.43(37.65,17.33) | 61.47(87.09,39.97) | 2.90(2.66,3.14) | 1.94(2.78,1.26) | 1.91(2.73,1.24) | -0.08(-0.35,0.20) |
| Peru | female | 445.93(628.14,301.54) | 1633.74(2222.57,1112.83) | 4.62(4.45,4.79) | 5.95(8.40,4.02) | 9.29(12.63,6.42) | 1.58(1.37,1.79) |
| Philippines | female | 292.83(449.60,164.20) | 1132.68(1705.02,681.98) | 5.19(4.97,5.41) | 1.46(2.31,0.78) | 2.29(3.44,1.36) | 1.87(1.65,2.10) |
| Poland | female | 1020.21(1386.35,685.36) | 866.41(1247.12,525.23) | -0.82(-1.19,-0.44) | 4.87(6.59,3.30) | 3.80(5.42,2.35) | -0.82(-1.08,-0.55) |
| Portugal | female | 456.48(602.45,312.68) | 283.64(379.65,193.68) | -1.96(-2.13,-1.79) | 8.57(11.30,5.86) | 4.28(5.67,2.95) | -2.79(-2.93,-2.65) |
| Puerto Rico | female | 101.94(140.69,68.19) | 115.02(162.26,77.28) | 0.03(-0.30,0.36) | 5.46(7.50,3.65) | 4.71(6.57,3.18) | -0.89(-1.05,-0.73) |
| Qatar | female | 0.73(1.21,0.41) | 10.27(16.70,5.91) | 10.22(9.98,10.46) | 1.24(2.05,0.66) | 2.36(3.87,1.37) | 2.74(2.30,3.19) |
| Romania | female | 1428.07(1837.15,1057.44) | 1688.10(2136.66,1253.70) | 0.68(0.33,1.02) | 11.46(14.86,8.41) | 13.56(17.09,10.19) | 0.79(0.48,1.10) |
| Russian Federation | female | 2423.34(3552.59,1433.13) | 4133.34(6379.39,2253.92) | 1.63(1.46,1.81) | 2.92(4.30,1.71) | 4.74(7.31,2.67) | 1.82(1.78,1.87) |
| Rwanda | female | 79.78(127.54,46.13) | 211.36(319.47,126.29) | 4.30(3.86,4.74) | 4.04(6.44,2.30) | 4.83(7.35,2.85) | 0.77(0.67,0.88) |
| Saint Lucia | female | 2.37(3.26,1.62) | 5.36(7.57,3.54) | 2.76(2.60,2.91) | 5.10(7.16,3.40) | 4.68(6.55,3.12) | -0.52(-0.76,-0.28) |
| Saint Vincent and the Grenadines | female | 0.81(1.15,0.54) | 1.49(2.13,0.99) | 2.02(1.86,2.18) | 2.31(3.30,1.50) | 2.29(3.21,1.52) | -0.30(-0.53,-0.07) |
| Samoa | female | 0.57(0.92,0.33) | 1.01(1.61,0.60) | 1.87(1.65,2.08) | 1.13(1.83,0.64) | 1.18(1.88,0.70) | -0.07(-0.23,0.09) |
| Sao Tome and Principe | female | 1.05(1.70,0.59) | 2.29(3.50,1.34) | 2.65(2.22,3.09) | 2.95(4.76,1.65) | 3.21(4.92,1.85) | 0.09(-0.12,0.29) |
| Saudi Arabia | female | 37.28(59.12,21.65) | 177.36(297.22,104.37) | 5.28(5.18,5.38) | 1.28(2.04,0.73) | 1.55(2.51,0.91) | 0.64(0.57,0.71) |
| Senegal | female | 43.46(68.02,24.10) | 102.76(163.52,58.05) | 2.88(2.70,3.06) | 2.17(3.46,1.18) | 2.06(3.33,1.14) | -0.35(-0.58,-0.13) |
| Serbia | female | 182.09(242.23,130.00) | 168.26(218.26,122.09) | -0.16(-0.32,0.00) | 3.39(4.49,2.40) | 3.35(4.37,2.43) | 0.14(0.06,0.21) |
| Seychelles | female | 0.63(0.98,0.39) | 2.10(3.17,1.28) | 4.64(4.40,4.87) | 2.31(3.59,1.41) | 3.39(5.05,2.12) | 1.51(1.34,1.68) |
| Sierra Leone | female | 24.12(39.63,11.55) | 53.56(84.67,30.35) | 2.65(2.29,3.01) | 2.24(3.74,1.05) | 2.29(3.62,1.25) | -0.19(-0.43,0.05) |
| Singapore | female | 21.84(34.25,13.30) | 39.80(61.21,24.16) | 1.81(1.68,1.94) | 1.31(2.05,0.79) | 1.03(1.59,0.63) | -1.09(-1.22,-0.96) |
| Slovakia | female | 173.15(231.04,117.36) | 215.62(280.95,152.79) | 0.76(0.61,0.90) | 6.08(8.07,4.16) | 6.50(8.42,4.63) | 0.47(0.31,0.62) |
| Slovenia | female | 90.66(119.91,61.56) | 66.51(87.01,46.52) | -0.98(-1.19,-0.78) | 8.50(11.22,5.81) | 5.43(7.06,3.78) | -1.38(-1.54,-1.23) |
| Solomon Islands | female | 0.78(1.35,0.37) | 2.41(4.04,1.24) | 3.94(3.83,4.05) | 0.68(1.22,0.31) | 0.84(1.44,0.43) | 0.71(0.56,0.85) |
| Somalia | female | 87.89(155.71,38.53) | 217.41(357.37,109.91) | 3.02(2.86,3.17) | 4.52(8.38,1.83) | 4.35(7.45,2.04) | -0.15(-0.24,-0.07) |
| South Africa | female | 168.21(278.19,78.32) | 323.71(523.18,160.40) | 2.09(1.83,2.34) | 1.19(2.04,0.51) | 1.12(1.84,0.55) | -0.31(-0.68,0.06) |
| Republic of Korea | female | 2528.04(3391.56,1777.45) | 2496.72(3325.15,1808.34) | -0.80(-1.21,-0.39) | 11.98(16.19,8.32) | 6.85(8.96,4.99) | -2.86(-3.17,-2.54) |
| South Sudan | female | 40.95(65.77,22.59) | 89.65(142.20,51.09) | 2.92(2.80,3.04) | 3.19(5.23,1.67) | 3.26(5.24,1.75) | 0.16(0.07,0.25) |
| Spain | female | 1999.81(2557.69,1487.57) | 1857.41(2437.42,1334.77) | -0.14(-0.38,0.10) | 9.94(12.82,7.31) | 6.11(8.01,4.44) | -1.91(-2.02,-1.79) |
| Sri Lanka | female | 121.23(181.92,74.63) | 360.14(536.12,233.02) | 3.64(3.42,3.86) | 1.71(2.60,1.03) | 2.58(3.79,1.68) | 1.20(0.91,1.48) |
| Sudan | female | 29.73(49.19,15.81) | 84.37(133.77,48.75) | 3.24(2.72,3.76) | 0.60(1.02,0.30) | 0.77(1.23,0.44) | 0.59(0.23,0.96) |
| Suriname | female | 7.12(10.40,4.61) | 17.29(24.62,11.45) | 3.14(3.09,3.19) | 4.53(6.59,2.91) | 5.05(7.16,3.37) | 0.10(0.00,0.21) |
| Eswatini | female | 3.14(5.24,1.59) | 6.23(10.03,3.51) | 1.92(1.54,2.31) | 1.29(2.18,0.62) | 1.38(2.25,0.76) | 0.09(-0.43,0.62) |
| Sweden | female | 180.32(249.65,120.51) | 227.29(314.36,150.67) | 0.92(0.67,1.18) | 3.79(5.17,2.57) | 4.16(5.64,2.79) | 0.45(0.20,0.71) |
| Switzerland | female | 190.24(254.68,132.21) | 262.01(345.08,184.61) | 1.15(0.81,1.49) | 4.81(6.40,3.35) | 5.09(6.68,3.58) | 0.11(-0.14,0.36) |
| Syrian Arab Republic | female | 23.01(37.02,13.26) | 80.14(130.38,46.53) | 4.83(4.61,5.06) | 0.76(1.22,0.44) | 1.09(1.76,0.63) | 1.52(1.38,1.66) |
| Taiwan (Province of China) | female | 316.45(471.15,200.12) | 500.28(741.18,306.21) | 1.54(0.89,2.20) | 3.68(5.52,2.32) | 2.62(3.85,1.62) | -1.45(-2.02,-0.89) |
| Tajikistan | female | 100.35(140.09,66.85) | 641.11(865.67,428.66) | 7.45(7.03,7.87) | 6.22(8.75,4.08) | 15.69(21.24,10.52) | 3.59(3.49,3.69) |
| United Republic of Tanzania | female | 226.57(342.21,138.21) | 595.47(901.36,353.93) | 3.21(3.05,3.36) | 3.29(5.05,1.95) | 3.60(5.55,2.08) | 0.25(0.19,0.32) |
| Thailand | female | 688.12(1040.64,436.90) | 1833.29(2725.90,1149.86) | 3.84(3.61,4.08) | 2.70(4.13,1.69) | 3.48(5.15,2.23) | 1.18(1.04,1.32) |
| Bahamas | female | 4.48(6.22,3.01) | 9.55(13.38,6.29) | 2.47(2.31,2.63) | 4.17(5.82,2.77) | 3.90(5.43,2.60) | -0.47(-0.59,-0.35) |
| Gambia | female | 5.02(7.75,2.94) | 16.41(25.27,9.72) | 4.13(4.00,4.26) | 2.35(3.79,1.30) | 2.59(4.03,1.53) | 0.31(0.23,0.38) |
| Timor-Leste | female | 4.99(8.02,2.98) | 14.10(21.97,8.39) | 3.43(3.32,3.55) | 2.11(3.34,1.24) | 3.22(5.05,1.89) | 1.35(1.26,1.44) |
| Togo | female | 22.41(36.05,12.66) | 60.83(96.45,34.23) | 3.06(2.91,3.21) | 2.46(3.96,1.35) | 2.16(3.39,1.18) | -0.84(-0.98,-0.69) |
| Tonga | female | 0.49(0.79,0.28) | 0.73(1.17,0.42) | 1.36(1.21,1.52) | 1.42(2.28,0.81) | 1.64(2.62,0.95) | 0.34(0.16,0.53) |
| Trinidad and Tobago | female | 11.35(16.27,7.23) | 17.70(25.62,11.55) | 1.34(1.22,1.47) | 2.22(3.15,1.41) | 2.12(3.02,1.38) | -0.28(-0.37,-0.19) |
| Tunisia | female | 25.14(40.76,13.80) | 80.29(130.50,47.53) | 4.19(4.08,4.30) | 0.89(1.45,0.50) | 1.15(1.85,0.68) | 0.92(0.84,1.00) |
| Turkey | female | 109.32(170.94,63.95) | 391.28(614.34,235.01) | 5.03(4.74,5.32) | 0.51(0.81,0.30) | 0.81(1.28,0.49) | 2.07(1.81,2.34) |
| Turkmenistan | female | 112.43(152.95,76.51) | 289.80(390.14,199.24) | 3.67(3.52,3.82) | 9.32(12.90,6.22) | 11.05(14.84,7.50) | 0.49(0.36,0.61) |
| Uganda | female | 130.60(202.21,79.31) | 365.58(551.56,219.04) | 3.50(3.29,3.71) | 3.22(5.06,1.88) | 3.61(5.60,2.10) | 0.31(0.20,0.42) |
| Ukraine | female | 759.25(1175.04,410.50) | 816.61(1410.35,343.44) | 0.35(0.24,0.45) | 2.64(4.02,1.48) | 3.19(5.46,1.43) | 0.95(0.86,1.05) |
| United Arab Emirates | female | 1.55(2.50,0.89) | 19.89(33.79,10.93) | 9.31(9.13,9.49) | 0.68(1.11,0.37) | 0.92(1.51,0.52) | 1.16(0.91,1.42) |
| United Kingdom | female | 1589.98(2149.20,1085.42) | 2946.37(3796.58,2203.71) | 1.86(1.45,2.26) | 5.18(6.99,3.56) | 8.47(10.81,6.38) | 1.41(1.10,1.72) |
| United States of America | female | 5412.16(7376.98,3719.94) | 7927.31(10665.04,5707.59) | 0.93(0.69,1.17) | 3.84(5.26,2.65) | 4.53(5.98,3.28) | 0.31(0.20,0.43) |
| Uruguay | female | 38.73(56.42,24.97) | 45.38(66.48,28.93) | 0.32(0.13,0.50) | 2.37(3.44,1.51) | 2.24(3.21,1.45) | -0.49(-0.68,-0.29) |
| Uzbekistan | female | 627.70(872.83,426.28) | 3090.60(4126.98,2188.98) | 6.21(5.86,6.56) | 9.50(13.30,6.29) | 17.96(23.93,12.65) | 2.36(2.07,2.64) |
| Vanuatu | female | 0.58(0.96,0.31) | 1.55(2.52,0.87) | 3.51(3.35,3.68) | 1.24(2.07,0.64) | 1.39(2.29,0.77) | 0.36(0.30,0.42) |
| Venezuela (Bolivarian Republic of) | female | 345.11(462.16,238.98) | 647.97(874.15,446.87) | 2.11(2.05,2.17) | 5.15(6.96,3.58) | 3.99(5.37,2.74) | -1.08(-1.29,-0.87) |
| Viet nam | female | 514.76(770.89,320.86) | 1816.62(2667.57,1154.26) | 4.63(4.12,5.14) | 2.16(3.28,1.31) | 3.08(4.50,1.96) | 1.27(0.83,1.71) |
| Virginia | female | 149.14(204.97,101.92) | 218.08(296.88,155.73) | 0.81(0.57,1.06) | 4.05(5.61,2.76) | 4.52(6.10,3.27) | 0.02(-0.10,0.14) |
| Yemen | female | 22.71(36.73,12.05) | 72.06(116.50,41.65) | 4.09(3.77,4.41) | 0.78(1.29,0.41) | 0.84(1.35,0.48) | 0.23(-0.02,0.48) |
| Zambia | female | 81.74(123.88,48.71) | 233.60(353.10,144.65) | 3.63(3.13,4.14) | 4.25(6.65,2.47) | 5.04(7.67,3.04) | 0.59(0.36,0.82) |
| Zimbabwe | female | 45.17(70.66,26.42) | 92.65(145.29,51.28) | 2.26(1.81,2.71) | 1.71(2.79,0.95) | 1.77(2.89,0.93) | 0.07(-0.32,0.46) |
| Monaco | female | 1.18(1.62,0.79) | 1.58(2.14,1.05) | 1.29(1.03,1.54) | 6.40(8.72,4.33) | 7.86(10.52,5.37) | 0.83(0.66,1.00) |
| San Marino | female | 1.47(1.96,1.02) | 2.46(3.28,1.65) | 2.13(1.87,2.39) | 12.40(16.62,8.54) | 12.05(15.91,8.19) | -0.10(-0.19,-0.01) |
| Saint Kitts and Nevis | female | 0.66(0.92,0.44) | 1.45(2.04,0.97) | 2.92(2.83,3.02) | 4.79(6.80,3.14) | 3.71(5.28,2.48) | -1.17(-1.32,-1.03) |
| Cook Islands | female | 0.03(0.04,0.01) | 0.04(0.07,0.03) | 2.08(1.94,2.22) | 0.35(0.55,0.20) | 0.42(0.66,0.24) | 0.62(0.59,0.65) |
| Nauru | female | 0.03(0.05,0.02) | 0.05(0.08,0.03) | 1.69(1.39,2.00) | 0.83(1.36,0.47) | 1.14(1.83,0.64) | 1.01(0.51,1.50) |
| Niue | female | 0.01(0.01,0.01) | 0.01(0.02,0.01) | 0.31(0.02,0.60) | 0.89(1.42,0.53) | 1.17(1.86,0.69) | 0.83(0.58,1.07) |
| Palau | female | 0.06(0.09,0.03) | 0.13(0.21,0.07) | 2.59(2.06,3.13) | 0.93(1.50,0.54) | 1.11(1.74,0.63) | 0.63(0.39,0.87) |
| Tokelau | female | 0.01(0.01,0.00) | 0.01(0.01,0.00) | -0.23(-0.51,0.05) | 0.82(1.34,0.46) | 0.83(1.29,0.49) | -0.14(-0.21,-0.07) |
| Tuvalu | female | 0.03(0.06,0.02) | 0.05(0.09,0.03) | 1.52(1.39,1.66) | 0.77(1.25,0.42) | 1.01(1.62,0.58) | 0.86(0.77,0.95) |

**Supplementary table 4. The deaths and age-standardized death rate of cirrhosis and other chronic liver diseases due to alcohol use in 1990 and 2019, and its temporal trends from 1990 to 2019.**

| **Nation** | **Sex** | **Death Cases No. (95% UI)** | | **1990-2019 EAPC of numbers** | **ASDR per 100,000 No.(95% UI)** | | **1990-2019 EAPC of ASDR** |
| --- | --- | --- | --- | --- | --- | --- | --- |
|  |  | **1990** | **2019** |  | **1990** | **2019** |  |
| Afghanistan | both | 159.20(264.98,83.04) | 224.08(380.32,114.51) | 0.67(0.44,0.89) | 2.26(3.62,1.25) | 1.83(2.98,1.02) | -1.02(-1.16,-0.87) |
| Albania | both | 111.66(135.01,87.83) | 148.33(204.86,100.95) | 1.31(0.98,1.64) | 5.50(6.70,4.34) | 3.54(4.86,2.45) | -1.23(-1.56,-0.89) |
| Algeria | both | 141.06(223.23,83.55) | 292.89(462.23,167.68) | 2.35(2.12,2.58) | 1.31(2.02,0.80) | 0.96(1.49,0.55) | -1.24(-1.42,-1.06) |
| American Samoa | both | 0.81(1.14,0.55) | 1.15(1.66,0.76) | 1.04(0.90,1.18) | 3.14(4.39,2.17) | 2.30(3.27,1.56) | -1.16(-1.32,-1.00) |
| Andorra | both | 2.95(4.52,1.79) | 5.85(7.84,4.15) | 2.07(1.84,2.29) | 5.22(7.93,3.20) | 4.18(5.63,2.96) | -0.83(-0.88,-0.79) |
| Angola | both | 475.18(734.80,286.92) | 1100.45(1594.27,712.50) | 2.93(2.74,3.11) | 11.40(17.05,7.04) | 9.32(13.48,6.17) | -0.73(-0.81,-0.65) |
| Antigua and Barbuda | both | 3.37(4.12,2.61) | 5.39(6.84,4.09) | 1.64(1.28,2.01) | 6.64(8.04,5.10) | 5.23(6.65,3.97) | -0.95(-1.28,-0.63) |
| Argentina | both | 1816.12(2317.47,1332.41) | 2619.55(3435.25,1875.74) | 1.49(1.19,1.80) | 5.61(7.12,4.13) | 4.99(6.50,3.57) | -0.19(-0.46,0.08) |
| Armenia | both | 150.00(181.43,117.97) | 469.52(598.88,359.09) | 4.55(4.02,5.09) | 5.48(6.66,4.34) | 11.64(14.77,8.89) | 3.26(2.67,3.84) |
| Australia | both | 266.33(359.36,190.28) | 404.08(552.15,282.43) | 2.01(1.73,2.30) | 1.41(1.90,1.01) | 1.07(1.47,0.74) | -0.44(-0.72,-0.16) |
| Austria | both | 1086.84(1255.44,901.38) | 885.24(1041.38,725.53) | -0.75(-0.83,-0.66) | 10.38(11.95,8.63) | 5.66(6.64,4.63) | -2.21(-2.28,-2.13) |
| Azerbaijan | both | 525.84(648.22,416.13) | 1244.21(1631.31,905.21) | 2.29(1.97,2.62) | 10.45(12.76,8.23) | 14.65(19.80,10.39) | 0.55(0.12,0.99) |
| Bahamas | both | 15.53(18.83,12.13) | 25.94(34.23,18.99) | 1.64(1.37,1.90) | 9.42(11.42,7.37) | 6.22(8.15,4.59) | -1.59(-1.87,-1.31) |
| Bahrain | both | 3.50(5.36,2.15) | 11.41(18.74,6.62) | 3.99(3.70,4.29) | 2.19(3.40,1.33) | 1.29(2.04,0.75) | -2.14(-2.44,-1.84) |
| Bangladesh | both | 4738.73(6701.00,3178.52) | 6415.29(9208.49,4222.95) | 0.86(0.63,1.09) | 9.43(13.44,6.25) | 4.97(7.05,3.29) | -2.47(-2.71,-2.23) |
| Barbados | both | 13.87(16.71,11.04) | 20.72(26.87,15.31) | 1.08(0.78,1.38) | 5.24(6.33,4.17) | 4.34(5.59,3.21) | -1.05(-1.28,-0.82) |
| Belarus | both | 339.34(415.48,264.72) | 1053.14(1426.82,754.98) | 4.77(3.68,5.87) | 2.61(3.18,2.04) | 7.16(9.70,5.17) | 4.40(3.21,5.61) |
| Belgium | both | 842.50(966.31,713.33) | 1023.25(1207.23,837.09) | 0.63(0.56,0.70) | 5.92(6.72,5.05) | 5.05(5.91,4.15) | -0.65(-0.76,-0.55) |
| Belize | both | 6.74(8.24,5.26) | 26.79(33.53,21.03) | 4.71(4.39,5.03) | 7.22(8.88,5.60) | 8.94(11.21,7.03) | 0.49(0.09,0.89) |
| Benin | both | 231.25(337.83,156.23) | 452.06(657.75,297.91) | 2.16(2.04,2.27) | 11.50(16.72,7.83) | 8.80(12.80,5.81) | -1.09(-1.19,-0.99) |
| Bermuda | both | 4.14(4.98,3.30) | 3.39(4.41,2.53) | -0.78(-1.20,-0.36) | 6.58(7.87,5.26) | 2.74(3.56,2.05) | -3.16(-3.56,-2.77) |
| Bhutan | both | 21.10(36.10,12.35) | 45.44(93.39,26.45) | 2.91(2.76,3.07) | 7.36(12.41,4.34) | 7.76(16.23,4.50) | 0.34(0.20,0.48) |
| Bolivia (Plurinational State of) | both | 530.23(773.43,334.12) | 1356.46(1870.73,927.88) | 3.34(3.30,3.38) | 15.94(23.28,10.07) | 15.31(21.09,10.57) | -0.18(-0.24,-0.12) |
| Bosnia and Herzegovina | both | 295.26(356.40,230.77) | 279.77(379.08,200.96) | -0.70(-0.94,-0.46) | 6.75(8.07,5.32) | 4.79(6.45,3.50) | -1.65(-1.95,-1.35) |
| Botswana | both | 49.96(87.22,27.12) | 106.30(157.55,65.34) | 1.97(1.52,2.42) | 8.20(14.19,4.51) | 6.91(10.31,4.38) | -1.07(-1.55,-0.59) |
| Brazil | both | 9534.03(10280.02,8827.05) | 17641.82(19291.02,16156.57) | 2.31(2.20,2.42) | 9.58(10.37,8.86) | 7.23(7.92,6.64) | -0.82(-0.93,-0.72) |
| Brunei Darussalam | both | 3.82(5.44,2.53) | 8.35(11.50,5.62) | 2.20(1.94,2.46) | 3.23(4.56,2.13) | 2.33(3.19,1.63) | -1.66(-1.90,-1.43) |
| Bulgaria | both | 943.03(1111.64,769.04) | 1344.47(1742.17,1003.52) | 1.11(0.93,1.28) | 7.63(8.96,6.23) | 10.95(14.24,8.26) | 1.10(0.91,1.29) |
| Burkina Faso | both | 475.28(664.98,319.44) | 693.58(1140.02,351.37) | 0.62(0.22,1.01) | 11.30(15.69,7.61) | 7.63(12.47,3.83) | -2.03(-2.41,-1.66) |
| Burundi | both | 330.37(518.26,186.80) | 452.70(835.07,248.79) | 0.61(0.30,0.92) | 13.90(21.66,7.98) | 9.41(17.17,5.23) | -1.83(-2.02,-1.64) |
| Cabo Verde | both | 11.82(18.53,7.44) | 31.58(42.95,22.08) | 2.48(2.02,2.93) | 5.46(8.45,3.45) | 7.08(9.69,4.93) | -0.08(-0.49,0.33) |
| Cambodia | both | 689.97(973.02,457.82) | 1756.80(2526.98,1165.57) | 3.25(3.04,3.47) | 13.75(19.35,9.23) | 13.97(20.03,9.33) | -0.04(-0.22,0.13) |
| Cameroon | both | 500.15(719.56,331.25) | 991.91(1508.00,581.99) | 2.25(2.12,2.37) | 10.89(15.62,7.23) | 7.75(11.78,4.64) | -1.31(-1.46,-1.17) |
| Canada | both | 949.26(1243.16,691.18) | 1669.54(2185.75,1197.74) | 2.03(1.92,2.13) | 2.99(3.90,2.17) | 2.58(3.35,1.86) | -0.50(-0.60,-0.41) |
| Central African Republic | both | 139.12(222.82,77.10) | 215.59(370.10,120.27) | 1.23(1.13,1.34) | 10.87(17.13,6.22) | 8.95(14.71,5.17) | -0.95(-1.03,-0.87) |
| Chad | both | 284.84(442.35,169.14) | 577.50(831.20,371.07) | 2.42(2.38,2.46) | 10.05(15.64,5.93) | 9.76(14.17,6.28) | -0.14(-0.26,-0.02) |
| Chile | both | 1163.16(1498.81,842.87) | 1613.82(2105.79,1164.90) | 1.38(1.17,1.60) | 11.11(14.36,8.11) | 6.73(8.75,4.90) | -1.54(-1.73,-1.34) |
| China | both | 27440.57(34391.59,21620.45) | 30548.39(38769.91,23231.35) | 0.26(0.15,0.37) | 3.04(3.80,2.43) | 1.50(1.90,1.15) | -2.55(-2.66,-2.45) |
| Colombia | both | 746.26(890.49,602.25) | 1473.45(2038.86,1034.79) | 1.92(1.70,2.15) | 4.21(5.06,3.40) | 2.77(3.83,1.94) | -1.92(-2.13,-1.72) |
| Comoros | both | 17.39(33.05,7.95) | 33.49(53.02,19.66) | 2.10(1.88,2.31) | 7.82(14.71,3.71) | 6.86(10.88,3.98) | -0.60(-0.78,-0.42) |
| Congo | both | 120.12(181.03,74.92) | 217.33(322.21,133.09) | 1.89(1.67,2.10) | 10.68(15.81,6.74) | 7.87(11.77,4.93) | -1.21(-1.32,-1.10) |
| Cook Islands | both | 0.37(0.53,0.24) | 0.48(0.70,0.30) | 1.04(0.92,1.16) | 2.75(3.92,1.81) | 1.97(2.83,1.28) | -1.04(-1.16,-0.92) |
| Costa Rica | both | 116.57(139.26,94.53) | 371.97(505.98,260.83) | 3.47(3.18,3.76) | 6.41(7.69,5.17) | 7.13(9.71,5.01) | -0.23(-0.50,0.04) |
| Côte d'Ivoire | both | 490.73(742.92,314.77) | 959.04(1449.51,594.99) | 2.11(1.90,2.31) | 11.40(16.97,7.35) | 8.28(12.50,5.20) | -1.16(-1.32,-1.00) |
| Croatia | both | 792.05(934.21,638.73) | 569.52(758.54,420.78) | -1.20(-1.38,-1.03) | 12.19(14.30,9.94) | 7.19(9.62,5.31) | -1.89(-2.08,-1.70) |
| Cuba | both | 415.90(503.18,330.00) | 901.86(1197.25,656.83) | 2.69(2.43,2.95) | 4.05(4.89,3.21) | 4.89(6.51,3.58) | 0.58(0.31,0.85) |
| Cyprus | both | 43.12(59.07,29.27) | 62.96(78.37,48.79) | 0.97(0.85,1.08) | 5.68(7.84,3.82) | 3.40(4.25,2.68) | -2.21(-2.36,-2.05) |
| Czechia | both | 985.63(1139.21,824.40) | 1161.74(1508.74,891.38) | 1.05(0.87,1.22) | 7.45(8.61,6.24) | 6.39(8.32,4.92) | -0.17(-0.32,-0.02) |
| Democratic People's Republic of Korea | both | 657.15(993.64,376.92) | 1118.31(1689.00,637.23) | 1.77(1.59,1.94) | 3.71(5.50,2.24) | 3.39(5.11,1.95) | -0.32(-0.49,-0.15) |
| Democratic Republic of the Congo | both | 1514.81(2226.33,954.26) | 2754.02(4246.74,1637.35) | 1.81(1.60,2.01) | 9.17(13.30,5.94) | 7.09(10.77,4.21) | -1.07(-1.19,-0.95) |
| Denmark | both | 369.82(428.84,304.23) | 440.53(535.99,345.36) | 0.08(-0.40,0.57) | 5.51(6.40,4.49) | 4.49(5.44,3.54) | -1.30(-1.79,-0.81) |
| Djibouti | both | 13.08(24.24,7.87) | 50.07(88.43,27.99) | 4.56(4.46,4.65) | 8.77(15.61,5.36) | 8.03(14.02,4.46) | -0.50(-0.58,-0.41) |
| Dominica | both | 4.31(5.37,3.27) | 4.53(5.95,3.34) | -0.05(-0.27,0.16) | 6.46(8.06,4.95) | 5.12(6.66,3.77) | -1.01(-1.17,-0.84) |
| Dominican Republic | both | 517.30(635.70,400.58) | 1151.95(1610.49,772.06) | 2.44(2.07,2.81) | 13.74(17.07,10.56) | 12.36(17.11,8.41) | -0.66(-1.09,-0.22) |
| Ecuador | both | 517.25(646.42,406.08) | 1535.37(2084.83,1107.70) | 4.08(3.84,4.33) | 9.34(11.72,7.35) | 10.26(14.01,7.43) | 0.61(0.39,0.84) |
| Egypt | both | 1742.65(2644.20,1085.26) | 3595.28(5968.87,1917.34) | 2.86(2.66,3.06) | 7.07(10.65,4.39) | 6.75(10.97,3.68) | 0.23(0.01,0.45) |
| El Salvador | both | 323.08(392.91,255.24) | 539.76(745.51,375.43) | 1.52(1.30,1.74) | 10.34(12.64,8.12) | 9.15(12.67,6.40) | -0.66(-0.87,-0.44) |
| Equatorial Guinea | both | 16.60(27.54,9.10) | 21.97(36.01,12.48) | 0.71(0.35,1.08) | 7.86(13.04,4.31) | 4.40(7.23,2.54) | -2.30(-2.53,-2.08) |
| Eritrea | both | 119.60(191.43,67.39) | 337.97(519.46,210.16) | 3.63(3.46,3.79) | 11.13(17.58,6.50) | 11.77(17.86,7.38) | 0.09(-0.05,0.24) |
| Estonia | both | 57.23(70.30,44.75) | 135.45(181.50,97.60) | 2.83(2.03,3.63) | 2.87(3.52,2.26) | 6.71(9.03,4.81) | 2.73(1.87,3.61) |
| Eswatini | both | 24.29(37.56,15.43) | 47.31(70.14,29.74) | 2.40(1.97,2.82) | 7.72(11.89,4.88) | 7.52(10.93,4.87) | 0.16(-0.17,0.49) |
| Ethiopia | both | 2585.44(3883.80,1450.42) | 3347.82(4262.28,2641.17) | 0.54(0.34,0.74) | 12.48(18.54,7.19) | 8.21(10.39,6.49) | -1.66(-1.77,-1.55) |
| Fiji | both | 10.32(14.80,6.88) | 17.29(26.17,10.85) | 1.94(1.84,2.04) | 2.49(3.57,1.69) | 2.18(3.20,1.42) | -0.45(-0.53,-0.37) |
| Finland | both | 286.97(335.52,233.89) | 551.65(665.06,428.95) | 3.10(2.52,3.70) | 4.41(5.16,3.59) | 5.95(7.17,4.61) | 1.77(1.24,2.30) |
| France | both | 6360.52(7397.56,5275.99) | 4907.16(5904.65,3936.16) | -1.01(-1.09,-0.92) | 8.50(9.85,6.99) | 4.26(5.09,3.40) | -2.53(-2.64,-2.42) |
| Gabon | both | 61.91(94.65,37.06) | 87.40(131.11,55.76) | 0.99(0.85,1.13) | 10.71(16.21,6.49) | 8.03(12.29,5.13) | -1.12(-1.19,-1.04) |
| Gambia | both | 36.08(57.12,21.54) | 86.92(128.45,53.26) | 2.80(2.44,3.17) | 9.90(15.68,5.93) | 8.81(12.89,5.46) | -0.61(-0.88,-0.35) |
| Georgia | both | 660.35(801.43,517.13) | 687.60(864.95,526.53) | 0.39(0.16,0.61) | 3.92(4.55,3.36) | 12.77(16.04,9.75) | -0.37(-4.80,4.27) |
| Germany | both | 10632.04(12278.88,8858.65) | 9846.14(11800.03,8059.49) | -0.81(-0.99,-0.62) | 9.23(10.63,7.68) | 6.11(7.19,5.03) | -2.00(-2.20,-1.81) |
| Ghana | both | 874.12(1350.75,531.93) | 1580.98(2259.26,1030.66) | 1.86(1.71,2.02) | 13.45(21.00,8.19) | 9.33(13.09,6.07) | -1.39(-1.52,-1.26) |
| Greece | both | 753.34(897.21,597.41) | 603.75(728.55,482.90) | -0.92(-1.16,-0.67) | 5.05(6.02,4.05) | 2.90(3.46,2.36) | -1.98(-2.27,-1.69) |
| Greenland | both | 1.60(2.34,1.03) | 2.55(3.74,1.65) | 1.89(1.74,2.04) | 3.53(5.12,2.34) | 3.28(4.70,2.18) | -0.13(-0.20,-0.06) |
| Grenada | both | 5.88(7.20,4.66) | 8.29(10.09,6.53) | 1.34(1.12,1.56) | 8.88(10.82,6.97) | 7.11(8.63,5.62) | -0.85(-1.06,-0.64) |
| Guam | both | 3.54(4.98,2.43) | 5.69(8.34,3.78) | 1.54(1.41,1.66) | 4.41(6.18,3.09) | 2.97(4.25,1.99) | -1.46(-1.74,-1.18) |
| Guatemala | both | 746.16(933.35,567.89) | 1914.53(2568.70,1379.00) | 3.12(2.88,3.35) | 17.75(21.97,13.55) | 16.15(21.59,11.60) | -0.52(-0.74,-0.31) |
| Guinea | both | 335.85(492.43,218.55) | 508.11(759.92,325.33) | 1.25(1.07,1.42) | 10.02(14.54,6.58) | 8.88(13.34,5.64) | -0.40(-0.50,-0.29) |
| Guinea-Bissau | both | 62.80(96.80,35.99) | 104.45(151.85,66.42) | 1.59(1.43,1.75) | 14.76(22.37,8.78) | 13.23(19.16,8.47) | -0.57(-0.66,-0.48) |
| Guyana | both | 85.40(106.13,66.48) | 109.66(147.93,79.05) | 1.23(0.94,1.52) | 20.44(25.54,15.94) | 15.95(21.15,11.52) | -0.56(-0.82,-0.30) |
| Haiti | both | 527.75(737.03,287.63) | 813.92(1222.44,410.96) | 1.57(1.39,1.74) | 15.10(21.03,8.54) | 10.83(16.15,5.44) | -1.06(-1.19,-0.92) |
| Honduras | both | 315.91(419.17,228.63) | 1006.90(1497.51,609.41) | 4.28(4.12,4.44) | 14.47(18.99,10.44) | 16.60(24.70,10.04) | 0.64(0.48,0.80) |
| Hungary | both | 2516.20(2926.55,2090.41) | 1602.94(2021.09,1236.78) | -2.68(-3.13,-2.24) | 18.31(21.31,15.24) | 9.55(12.21,7.29) | -3.49(-3.98,-3.00) |
| Iceland | both | 5.55(6.79,4.46) | 7.69(9.58,6.02) | 0.99(0.92,1.07) | 2.09(2.53,1.67) | 1.52(1.87,1.19) | -1.29(-1.38,-1.21) |
| India | both | 31840.63(39729.76,26614.43) | 61527.26(77858.97,48695.18) | 2.24(1.99,2.50) | 6.19(7.83,5.15) | 5.14(6.51,4.09) | -0.75(-1.01,-0.48) |
| Indonesia | both | 8258.22(10256.76,6550.60) | 14250.74(18054.05,11181.03) | 1.94(1.84,2.04) | 7.90(9.74,6.27) | 6.71(8.31,5.37) | -0.44(-0.54,-0.33) |
| Iran (Islamic Republic of) | both | 233.44(301.79,176.53) | 484.53(600.78,387.30) | 3.00(2.74,3.25) | 0.95(1.23,0.71) | 0.68(0.84,0.55) | -0.78(-1.01,-0.54) |
| Iraq | both | 87.16(139.38,51.62) | 199.97(310.24,120.61) | 2.78(2.66,2.90) | 1.12(1.79,0.67) | 0.87(1.32,0.52) | -0.96(-1.10,-0.82) |
| Ireland | both | 83.78(100.22,67.75) | 178.93(213.07,144.10) | 2.85(2.41,3.29) | 2.16(2.58,1.75) | 2.56(3.05,2.06) | 0.67(0.16,1.17) |
| Israel | both | 147.24(186.32,113.19) | 276.79(349.28,209.80) | 1.60(1.29,1.91) | 3.11(3.87,2.39) | 2.43(3.05,1.86) | -1.39(-1.68,-1.11) |
| Italy | both | 4013.53(4522.61,3528.02) | 2467.18(2870.68,2108.67) | -1.93(-2.10,-1.75) | 4.69(5.29,4.16) | 1.89(2.18,1.63) | -3.38(-3.56,-3.20) |
| Jamaica | both | 54.27(66.15,42.12) | 72.82(99.49,51.73) | 0.72(0.32,1.12) | 3.16(3.87,2.45) | 2.43(3.33,1.72) | -1.21(-1.61,-0.81) |
| Japan | both | 4636.60(5241.05,4096.38) | 4392.00(5279.30,3578.99) | -0.05(-0.20,0.10) | 2.75(3.09,2.43) | 1.39(1.65,1.17) | -2.17(-2.34,-2.01) |
| Jordan | both | 15.40(24.70,9.36) | 48.88(74.40,30.08) | 3.72(3.45,3.99) | 1.23(1.96,0.75) | 0.81(1.24,0.50) | -1.61(-1.77,-1.45) |
| Kazakhstan | both | 799.49(962.35,638.59) | 2855.57(3671.54,2200.12) | 5.13(4.59,5.66) | 6.24(7.50,5.02) | 15.81(20.20,12.33) | 3.99(3.39,4.59) |
| Kenya | both | 1260.02(2240.74,768.18) | 3376.26(5010.44,2343.25) | 3.70(3.59,3.82) | 15.46(27.37,9.54) | 15.25(22.62,10.63) | 0.22(0.09,0.35) |
| Kiribati | both | 3.82(6.06,1.96) | 4.72(8.55,2.11) | 0.79(0.56,1.02) | 9.16(13.77,5.40) | 5.97(10.11,2.94) | -1.55(-1.73,-1.37) |
| Kuwait | both | 4.02(6.03,2.55) | 16.19(24.68,10.03) | 5.43(4.75,6.12) | 0.60(0.91,0.38) | 0.61(0.95,0.37) | 0.57(-0.27,1.42) |
| Kyrgyzstan | both | 349.26(420.62,275.07) | 880.56(1085.30,693.09) | 3.18(2.72,3.65) | 11.23(13.48,8.88) | 16.97(20.65,13.41) | 1.45(0.84,2.06) |
| Lao People's Democratic Republic | both | 158.45(237.85,93.90) | 267.65(414.24,159.99) | 1.52(1.22,1.83) | 6.89(10.32,4.12) | 5.56(8.56,3.29) | -1.02(-1.25,-0.80) |
| Latvia | both | 97.96(119.72,75.85) | 182.25(238.64,136.20) | 1.91(1.23,2.59) | 2.81(3.41,2.18) | 5.98(7.81,4.50) | 2.44(1.74,3.15) |
| Lebanon | both | 23.72(37.49,13.25) | 41.21(73.43,19.66) | 2.15(2.01,2.30) | 1.10(1.70,0.62) | 0.80(1.43,0.38) | -0.89(-1.08,-0.69) |
| Lesotho | both | 67.87(120.37,37.89) | 100.93(157.38,60.60) | 1.29(1.11,1.47) | 6.60(11.50,3.69) | 7.41(11.61,4.67) | 0.47(0.32,0.61) |
| Liberia | both | 151.25(221.75,99.34) | 217.81(322.74,133.52) | 0.79(0.60,0.99) | 13.63(19.96,9.22) | 10.08(14.67,6.24) | -1.47(-1.61,-1.32) |
| Libya | both | 24.85(43.14,12.43) | 52.39(83.45,28.29) | 2.62(2.34,2.90) | 1.37(2.43,0.68) | 1.05(1.70,0.58) | -0.87(-1.03,-0.71) |
| Lithuania | both | 139.80(168.84,109.51) | 399.85(523.14,301.99) | 4.47(3.33,5.63) | 3.18(3.85,2.52) | 9.26(12.22,7.01) | 4.61(3.47,5.76) |
| Luxembourg | both | 47.44(55.51,39.08) | 43.40(53.14,34.12) | -0.54(-0.64,-0.44) | 9.21(10.75,7.58) | 4.58(5.61,3.59) | -2.60(-2.70,-2.49) |
| Madagascar | both | 526.14(758.86,341.20) | 960.41(1449.18,600.61) | 2.03(1.88,2.17) | 10.10(14.69,6.56) | 8.41(12.49,5.43) | -0.73(-0.84,-0.61) |
| Malawi | both | 557.26(824.16,365.11) | 803.92(1154.66,538.40) | 0.90(0.74,1.06) | 14.25(20.66,9.50) | 10.75(15.60,7.32) | -1.22(-1.43,-1.00) |
| Malaysia | both | 246.87(343.01,169.49) | 835.26(1237.42,527.57) | 3.85(3.56,4.14) | 2.52(3.51,1.72) | 3.07(4.51,1.93) | 0.09(-0.25,0.42) |
| Maldives | both | 3.23(5.03,1.73) | 6.37(8.77,4.48) | 1.98(1.56,2.41) | 3.30(5.07,1.87) | 1.90(2.69,1.30) | -2.21(-2.44,-1.98) |
| Mali | both | 506.77(746.93,335.64) | 684.01(1091.74,432.23) | 0.76(0.57,0.96) | 11.90(17.32,7.86) | 7.59(11.88,4.90) | -1.83(-2.01,-1.64) |
| Malta | both | 16.04(19.39,12.74) | 18.48(22.80,14.45) | 0.51(0.41,0.61) | 3.75(4.54,2.97) | 2.33(2.87,1.84) | -1.68(-1.74,-1.62) |
| Marshall Islands | both | 1.20(1.77,0.71) | 1.95(3.44,1.00) | 1.69(1.59,1.78) | 6.62(9.75,4.07) | 4.86(8.37,2.57) | -1.11(-1.22,-0.99) |
| Mauritania | both | 120.48(176.34,75.08) | 131.51(207.30,76.35) | 0.14(-0.03,0.31) | 11.94(17.60,7.40) | 6.22(9.59,3.68) | -2.41(-2.52,-2.30) |
| Mauritius | both | 50.65(68.13,35.53) | 61.99(90.42,41.17) | 0.11(-0.23,0.46) | 6.20(8.31,4.36) | 3.47(5.01,2.33) | -2.62(-2.93,-2.32) |
| Mexico | both | 8481.13(9296.37,7674.81) | 16907.15(20723.44,13729.15) | 2.08(1.93,2.23) | 18.15(19.90,16.43) | 13.99(17.14,11.34) | -1.24(-1.39,-1.09) |
| Micronesia (Federated States of) | both | 3.44(5.31,1.93) | 3.88(7.03,1.75) | 0.41(0.20,0.62) | 6.74(10.26,3.99) | 4.84(8.38,2.34) | -1.17(-1.35,-0.99) |
| Monaco | both | 2.62(3.49,1.89) | 3.23(4.19,2.35) | 0.89(0.79,0.98) | 4.52(5.98,3.30) | 4.15(5.37,3.02) | -0.12(-0.21,-0.03) |
| Mongolia | both | 259.19(336.55,188.30) | 678.06(905.47,490.61) | 3.58(3.40,3.75) | 24.58(31.91,17.88) | 29.25(38.89,21.58) | 0.85(0.48,1.21) |
| Montenegro | both | 14.68(19.03,11.24) | 24.02(31.93,17.79) | 1.48(1.29,1.67) | 2.27(2.94,1.75) | 2.54(3.35,1.89) | 0.20(0.05,0.36) |
| Morocco | both | 166.97(258.52,95.22) | 348.69(534.34,207.55) | 2.53(2.25,2.80) | 1.29(2.01,0.72) | 1.18(1.78,0.72) | -0.34(-0.63,-0.05) |
| Mozambique | both | 311.79(456.94,199.02) | 501.55(830.21,268.75) | 1.85(1.69,2.01) | 5.37(7.66,3.50) | 4.56(7.44,2.48) | -0.39(-0.59,-0.20) |
| Myanmar | both | 2374.54(3512.38,1473.79) | 4110.38(5990.23,2723.96) | 1.89(1.76,2.01) | 8.70(12.97,5.48) | 7.68(10.94,5.11) | -0.47(-0.58,-0.37) |
| Namibia | both | 50.06(80.91,28.87) | 90.67(134.75,57.77) | 1.49(1.23,1.75) | 6.79(11.00,3.93) | 6.17(9.07,3.96) | -0.74(-0.97,-0.52) |
| Nauru | both | 0.31(0.52,0.13) | 0.29(0.50,0.12) | -0.46(-0.56,-0.36) | 6.32(10.37,2.96) | 5.21(8.53,2.54) | -0.75(-0.84,-0.65) |
| Nepal | both | 1541.69(2286.50,1040.32) | 3179.37(4691.77,2172.82) | 2.89(2.61,3.18) | 14.73(22.12,9.82) | 14.11(20.80,9.64) | 0.11(-0.16,0.38) |
| Netherlands | both | 596.36(708.03,485.41) | 743.79(927.27,584.88) | 0.30(-0.01,0.61) | 3.13(3.69,2.55) | 2.30(2.81,1.83) | -1.53(-1.87,-1.20) |
| New Zealand | both | 34.62(40.68,29.21) | 50.19(60.46,41.32) | 1.29(1.23,1.35) | 0.92(1.08,0.78) | 0.67(0.81,0.56) | -1.16(-1.22,-1.09) |
| Nicaragua | both | 170.19(205.56,135.13) | 620.58(817.06,459.21) | 4.60(4.44,4.76) | 10.47(12.72,8.33) | 13.63(17.63,10.15) | 1.01(0.87,1.15) |
| Niger | both | 316.41(492.98,196.37) | 685.39(1123.21,400.21) | 2.36(2.19,2.53) | 10.68(16.67,6.69) | 8.31(13.43,4.90) | -1.28(-1.45,-1.11) |
| Nigeria | both | 5616.12(8316.19,3630.90) | 8774.15(13591.47,5593.25) | 1.77(1.63,1.90) | 12.50(18.34,8.13) | 9.91(15.00,6.47) | -0.52(-0.70,-0.35) |
| Niue | both | 0.10(0.14,0.06) | 0.08(0.11,0.05) | -1.04(-1.24,-0.84) | 4.57(6.87,2.81) | 3.66(5.31,2.33) | -0.89(-1.02,-0.76) |
| North Macedonia | both | 77.62(96.17,60.94) | 146.78(196.64,106.59) | 2.06(1.96,2.17) | 3.99(4.92,3.15) | 4.56(6.11,3.35) | 0.31(0.21,0.40) |
| Northern Mariana Islands | both | 1.71(2.48,1.10) | 2.39(3.47,1.54) | 1.14(0.85,1.43) | 7.34(10.45,5.03) | 4.25(5.93,2.88) | -2.15(-2.33,-1.97) |
| Norway | both | 147.34(165.93,129.97) | 150.89(176.64,130.80) | 0.16(0.10,0.22) | 2.51(2.82,2.24) | 1.69(1.96,1.47) | -1.34(-1.44,-1.23) |
| Oman | both | 10.73(17.76,5.85) | 21.47(35.97,11.61) | 3.09(2.83,3.35) | 1.63(2.69,0.90) | 1.32(2.05,0.78) | -0.05(-0.40,0.30) |
| Pakistan | both | 2795.91(4756.16,1514.66) | 4413.20(6780.55,2830.11) | 1.45(1.40,1.49) | 4.84(8.34,2.58) | 3.91(6.15,2.44) | -0.89(-1.03,-0.75) |
| Palau | both | 0.43(0.72,0.21) | 0.81(1.35,0.41) | 2.08(1.77,2.39) | 4.09(6.78,2.14) | 3.47(5.72,1.87) | -0.46(-0.56,-0.35) |
| Palestine | both | 12.38(22.56,6.48) | 25.61(39.06,15.86) | 2.89(2.66,3.13) | 1.50(2.69,0.82) | 1.19(1.82,0.75) | -0.53(-0.71,-0.34) |
| Panama | both | 73.64(88.76,58.26) | 187.90(257.43,133.26) | 3.56(3.36,3.76) | 4.91(5.95,3.86) | 4.51(6.19,3.21) | -0.04(-0.22,0.13) |
| Papua New Guinea | both | 48.00(70.46,30.68) | 124.58(192.80,74.38) | 3.61(3.41,3.81) | 2.08(3.07,1.34) | 2.05(3.08,1.25) | 0.13(0.03,0.22) |
| Paraguay | both | 111.77(136.59,87.67) | 346.74(470.06,245.56) | 3.93(3.72,4.15) | 4.88(5.95,3.77) | 6.03(8.20,4.30) | 0.69(0.48,0.90) |
| Peru | both | 1494.53(1930.12,1134.21) | 2838.94(4032.54,1879.05) | 2.13(1.93,2.33) | 12.02(15.49,9.05) | 8.80(12.52,5.82) | -1.17(-1.35,-0.99) |
| Philippines | both | 1336.54(1815.95,987.93) | 3010.65(3879.94,2257.89) | 2.74(2.58,2.91) | 4.04(5.55,2.91) | 3.59(4.61,2.73) | -0.49(-0.67,-0.30) |
| Poland | both | 2582.71(2809.46,2364.11) | 4121.02(5024.47,3353.36) | 1.87(1.60,2.14) | 5.97(6.48,5.47) | 6.73(8.29,5.44) | 0.58(0.32,0.84) |
| Portugal | both | 1722.49(2004.93,1412.12) | 998.41(1189.98,811.17) | -2.16(-2.30,-2.02) | 13.04(15.14,10.79) | 5.04(5.96,4.09) | -3.56(-3.70,-3.42) |
| Puerto Rico | both | 433.07(513.46,351.02) | 423.84(570.97,301.42) | -0.88(-1.23,-0.53) | 12.11(14.34,9.75) | 6.66(9.07,4.77) | -2.87(-3.23,-2.50) |
| Qatar | both | 2.52(4.03,1.45) | 14.75(24.46,8.12) | 6.54(6.34,6.74) | 2.43(3.87,1.47) | 2.33(3.78,1.29) | 0.05(-0.26,0.36) |
| Republic of Korea | both | 5778.36(7050.48,4516.54) | 4825.56(5938.66,3812.59) | -1.77(-2.17,-1.37) | 17.10(20.90,13.55) | 5.46(6.71,4.34) | -5.07(-5.46,-4.67) |
| Republic of Moldova | both | 1399.37(1631.28,1132.38) | 1326.69(1627.42,1039.42) | -0.19(-0.56,0.19) | 30.22(35.23,24.80) | 23.42(28.65,18.47) | -0.83(-1.23,-0.42) |
| Romania | both | 3546.09(4170.21,2917.43) | 5074.65(6474.87,3907.40) | 0.74(0.34,1.15) | 12.47(14.55,10.32) | 15.10(19.42,11.52) | 0.16(-0.24,0.56) |
| Russian Federation | both | 6309.72(7126.74,5546.05) | 19609.16(23478.68,16272.40) | 3.93(3.09,4.79) | 3.46(3.91,3.06) | 9.17(10.94,7.58) | 3.46(2.57,4.35) |
| Rwanda | both | 524.47(749.06,338.03) | 571.89(844.39,366.34) | -0.07(-0.57,0.44) | 17.68(25.43,11.55) | 9.15(13.38,5.95) | -3.18(-3.57,-2.80) |
| Saint Kitts and Nevis | both | 4.37(5.35,3.36) | 5.64(7.43,3.97) | 0.43(-0.23,1.09) | 12.76(15.50,9.91) | 7.85(10.27,5.72) | -2.17(-2.60,-1.74) |
| Saint Lucia | both | 8.73(10.46,7.00) | 14.12(17.84,10.83) | 1.44(0.97,1.92) | 10.14(12.13,8.11) | 6.47(8.17,4.98) | -1.91(-2.36,-1.46) |
| Saint Vincent and the Grenadines | both | 4.54(5.60,3.51) | 8.56(10.89,6.57) | 2.11(1.59,2.63) | 6.47(7.93,4.97) | 6.25(7.90,4.83) | -0.37(-0.81,0.07) |
| Samoa | both | 4.27(6.76,2.71) | 5.35(7.89,3.41) | 0.85(0.72,0.97) | 4.68(7.39,2.97) | 3.46(4.97,2.24) | -1.00(-1.13,-0.88) |
| San Marino | both | 1.97(2.56,1.48) | 3.39(5.06,2.13) | 2.05(1.96,2.15) | 6.21(7.98,4.72) | 5.51(8.25,3.41) | -0.28(-0.39,-0.16) |
| Sao Tome and Principe | both | 9.01(14.00,5.33) | 13.60(20.59,8.97) | 1.03(0.83,1.23) | 13.93(21.52,8.37) | 12.52(18.98,8.12) | -0.63(-0.85,-0.41) |
| Saudi Arabia | both | 153.80(261.27,81.80) | 247.58(379.99,148.30) | 1.08(0.81,1.35) | 2.93(5.17,1.54) | 1.73(2.71,1.04) | -1.99(-2.12,-1.86) |
| Senegal | both | 300.27(466.87,187.03) | 552.38(872.04,344.79) | 2.17(1.94,2.40) | 9.11(14.05,5.68) | 7.09(10.99,4.43) | -0.86(-1.08,-0.65) |
| Serbia | both | 634.12(802.02,481.63) | 680.36(914.47,504.53) | -0.16(-0.36,0.04) | 5.33(6.72,4.16) | 4.60(6.18,3.42) | -0.83(-1.00,-0.66) |
| Seychelles | both | 3.23(4.54,2.18) | 7.73(10.68,5.19) | 2.85(2.64,3.07) | 5.85(8.23,3.90) | 6.49(8.94,4.43) | 0.06(-0.22,0.35) |
| Sierra Leone | both | 223.89(331.09,143.93) | 271.62(404.27,171.06) | 0.20(-0.08,0.49) | 11.70(17.20,7.57) | 7.19(10.72,4.51) | -2.16(-2.42,-1.89) |
| Singapore | both | 30.65(42.35,21.40) | 41.83(58.87,28.58) | 1.05(0.69,1.42) | 1.32(1.86,0.92) | 0.52(0.73,0.36) | -3.19(-3.43,-2.96) |
| Slovakia | both | 682.04(822.70,554.10) | 835.59(1131.34,610.25) | 1.29(1.02,1.56) | 11.67(14.04,9.47) | 9.68(13.11,7.08) | -0.13(-0.38,0.13) |
| Slovenia | both | 322.02(443.24,230.58) | 274.10(379.52,198.41) | -0.71(-0.93,-0.49) | 13.22(18.22,9.44) | 7.14(9.86,5.16) | -2.31(-2.55,-2.07) |
| Solomon Islands | both | 11.52(17.99,6.89) | 23.98(34.98,15.41) | 2.63(2.53,2.73) | 6.70(10.52,4.12) | 5.83(8.32,3.86) | -0.38(-0.46,-0.29) |
| Somalia | both | 320.60(506.70,183.07) | 708.70(1150.96,408.80) | 2.77(2.68,2.86) | 12.10(18.77,7.09) | 10.11(16.28,5.91) | -0.59(-0.68,-0.51) |
| South Africa | both | 898.56(1209.33,666.96) | 1350.71(1626.66,1099.56) | 1.02(0.51,1.54) | 4.03(5.49,2.95) | 2.91(3.49,2.37) | -1.41(-1.88,-0.95) |
| South Sudan | both | 241.82(409.09,144.31) | 331.31(554.48,191.91) | 1.18(1.05,1.31) | 10.04(16.77,6.01) | 8.63(14.40,5.15) | -0.42(-0.49,-0.36) |
| Spain | both | 4865.37(5749.48,3964.84) | 3456.68(4216.38,2806.97) | -1.29(-1.40,-1.17) | 9.39(10.99,7.69) | 3.93(4.69,3.18) | -3.11(-3.24,-2.99) |
| Sri Lanka | both | 682.42(930.82,471.87) | 1025.91(1504.10,653.57) | 0.60(-0.02,1.22) | 5.74(7.81,4.04) | 4.01(5.83,2.58) | -1.94(-2.49,-1.38) |
| Sudan | both | 169.25(282.10,91.51) | 276.07(505.17,141.40) | 1.43(1.33,1.53) | 1.90(3.19,1.01) | 1.56(2.86,0.79) | -0.76(-0.81,-0.71) |
| Suriname | both | 27.42(33.30,21.40) | 53.26(69.74,39.25) | 2.14(1.92,2.35) | 10.16(12.36,7.97) | 8.63(11.22,6.36) | -0.90(-1.10,-0.70) |
| Sweden | both | 405.02(442.44,367.83) | 497.27(559.79,438.57) | 0.95(0.80,1.10) | 3.14(3.42,2.88) | 2.68(3.01,2.37) | -0.33(-0.49,-0.18) |
| Switzerland | both | 416.06(490.46,341.73) | 465.41(577.39,365.84) | 0.21(0.08,0.35) | 4.41(5.18,3.60) | 2.93(3.57,2.31) | -1.62(-1.79,-1.44) |
| Syrian Arab Republic | both | 76.52(120.13,44.07) | 147.89(236.32,82.65) | 2.50(2.26,2.74) | 1.52(2.36,0.88) | 1.32(2.13,0.77) | -0.41(-0.60,-0.23) |
| Taiwan (Province of China) | both | 1129.80(1467.31,863.06) | 1727.26(2460.87,1152.96) | 1.07(0.70,1.45) | 7.01(9.12,5.36) | 4.54(6.46,3.03) | -1.95(-2.28,-1.61) |
| Tajikistan | both | 267.11(332.02,207.31) | 791.85(1043.84,582.39) | 3.65(3.46,3.84) | 9.11(11.35,7.06) | 14.50(18.88,10.81) | 1.61(1.44,1.78) |
| Thailand | both | 2470.76(3306.14,1775.62) | 5731.32(8531.58,3746.82) | 3.18(3.04,3.32) | 6.15(8.29,4.42) | 5.59(8.22,3.69) | -0.16(-0.30,-0.03) |
| Timor-Leste | both | 16.94(29.70,9.03) | 42.34(71.74,21.64) | 3.14(2.90,3.39) | 4.82(8.26,2.56) | 5.06(8.54,2.68) | -0.07(-0.30,0.16) |
| Togo | both | 139.58(202.02,92.63) | 300.37(448.38,190.30) | 2.29(2.08,2.50) | 10.81(15.72,7.27) | 7.55(11.18,4.85) | -1.66(-1.89,-1.44) |
| Tokelau | both | 0.05(0.08,0.03) | 0.04(0.05,0.02) | -1.41(-1.72,-1.10) | 3.93(5.98,2.43) | 2.70(3.99,1.69) | -1.34(-1.44,-1.24) |
| Tonga | both | 3.08(4.45,1.97) | 3.76(5.39,2.43) | 0.69(0.48,0.90) | 5.30(7.60,3.45) | 4.66(6.65,3.00) | -0.45(-0.65,-0.25) |
| Trinidad and Tobago | both | 57.00(67.65,45.52) | 80.52(112.43,55.47) | 1.18(0.97,1.40) | 6.59(7.85,5.24) | 4.33(6.04,2.99) | -1.52(-1.75,-1.29) |
| Tunisia | both | 56.97(94.38,32.37) | 110.52(190.07,61.21) | 2.33(2.31,2.36) | 1.19(1.96,0.69) | 0.90(1.53,0.50) | -0.86(-0.90,-0.82) |
| Turkey | both | 340.92(538.91,199.91) | 626.03(954.58,383.78) | 2.21(1.99,2.44) | 0.94(1.50,0.55) | 0.72(1.09,0.44) | -0.78(-1.00,-0.55) |
| Turkmenistan | both | 262.12(318.46,208.71) | 940.22(1232.76,691.33) | 4.60(4.30,4.90) | 12.94(15.58,10.38) | 20.80(26.92,15.44) | 1.65(1.38,1.92) |
| Tuvalu | both | 0.39(0.62,0.21) | 0.42(0.67,0.24) | 0.31(0.05,0.56) | 5.40(8.40,3.07) | 3.99(6.36,2.31) | -0.96(-1.16,-0.76) |
| Uganda | both | 678.74(1038.48,432.23) | 1209.91(1718.62,799.63) | 1.59(1.37,1.81) | 10.41(15.94,6.59) | 8.39(11.87,5.53) | -1.09(-1.29,-0.89) |
| Ukraine | both | 3035.74(3461.89,2666.36) | 8231.90(10219.29,6607.10) | 3.41(2.70,4.12) | 4.30(4.88,3.77) | 12.81(15.93,10.16) | 3.78(3.00,4.57) |
| United Arab Emirates | both | 5.47(9.48,3.09) | 44.71(75.54,22.66) | 7.01(6.66,7.36) | 1.34(2.29,0.72) | 1.07(1.88,0.53) | -0.94(-1.40,-0.48) |
| United Kingdom | both | 2520.36(2766.40,2253.28) | 5297.83(5801.52,4817.57) | 2.65(2.21,3.09) | 3.22(3.52,2.90) | 5.16(5.63,4.71) | 1.63(1.13,2.12) |
| United Republic of Tanzania | both | 1128.23(1612.47,755.67) | 1910.97(2778.90,1209.14) | 1.81(1.61,2.01) | 10.09(14.24,6.83) | 7.57(11.05,4.72) | -0.94(-1.18,-0.70) |
| United States of America | both | 10565.81(11966.95,9213.35) | 17341.02(20292.07,14692.19) | 2.16(2.00,2.31) | 3.57(4.05,3.10) | 3.37(3.95,2.86) | 0.14(0.02,0.26) |
| United States Virgin Islands | both | 7.84(9.90,5.86) | 14.44(18.68,10.66) | 2.55(2.26,2.83) | 8.65(10.97,6.46) | 8.16(10.55,6.07) | 0.10(-0.19,0.39) |
| Uruguay | both | 150.97(200.15,108.06) | 138.20(183.79,98.81) | -0.68(-0.84,-0.52) | 3.98(5.22,2.87) | 2.73(3.66,1.96) | -1.63(-1.76,-1.49) |
| Uzbekistan | both | 1327.03(1620.79,1034.69) | 5088.29(6451.21,3890.51) | 3.90(3.42,4.38) | 11.18(13.61,8.76) | 21.05(26.18,16.16) | 1.60(1.02,2.18) |
| Vanuatu | both | 4.44(7.25,2.58) | 9.23(14.84,4.91) | 2.49(2.31,2.66) | 6.08(9.96,3.71) | 4.93(7.85,2.70) | -0.91(-1.05,-0.76) |
| Venezuela (Bolivarian Republic of) | both | 799.11(951.10,644.96) | 1907.56(2656.75,1328.19) | 2.93(2.68,3.18) | 7.81(9.29,6.30) | 6.33(8.74,4.44) | -0.95(-1.19,-0.71) |
| Viet Nam | both | 2819.90(4328.92,1728.98) | 5351.69(7632.31,3481.13) | 1.99(1.43,2.56) | 7.02(10.65,4.33) | 5.48(7.76,3.65) | -1.20(-1.70,-0.69) |
| Yemen | both | 88.89(153.50,46.59) | 190.13(311.63,106.29) | 2.56(2.50,2.61) | 1.86(3.32,0.94) | 1.49(2.50,0.82) | -0.90(-0.97,-0.83) |
| Zambia | both | 477.34(717.66,307.37) | 1006.42(1438.36,673.38) | 2.20(1.98,2.42) | 16.65(24.98,10.96) | 14.35(20.42,9.54) | -0.83(-1.05,-0.61) |
| Zimbabwe | both | 304.71(423.32,203.12) | 478.06(757.57,276.81) | 1.38(1.21,1.55) | 7.34(10.25,4.94) | 6.55(10.33,3.97) | -0.27(-0.48,-0.06) |
| Afghanistan | male | 107.65(179.07,53.69) | 142.45(236.09,74.57) | 0.41(0.13,0.69) | 2.94(4.80,1.50) | 1.83(2.98,1.02) | -0.99(-1.12,-0.85) |
| Albania | male | 75.86(93.23,59.24) | 103.41(143.01,70.15) | 1.50(1.14,1.87) | 7.86(9.69,6.05) | 3.54(4.86,2.45) | -1.02(-1.41,-0.62) |
| Algeria | male | 97.96(158.87,56.10) | 210.68(351.91,113.52) | 2.48(2.23,2.73) | 1.78(2.80,1.06) | 0.96(1.49,0.55) | -1.28(-1.47,-1.09) |
| American Samoa | male | 0.66(0.94,0.44) | 0.85(1.22,0.55) | 0.67(0.53,0.81) | 4.85(6.86,3.32) | 2.30(3.27,1.56) | -1.28(-1.40,-1.16) |
| Andorra | male | 2.26(3.40,1.38) | 4.27(5.66,3.05) | 1.87(1.60,2.14) | 7.71(11.50,4.76) | 4.18(5.63,2.96) | -0.86(-0.88,-0.83) |
| Angola | male | 377.77(571.39,230.66) | 841.67(1219.26,550.99) | 2.79(2.59,2.99) | 18.33(27.25,11.19) | 9.32(13.48,6.17) | -0.51(-0.59,-0.43) |
| Antigua and Barbuda | male | 2.56(3.09,1.99) | 3.86(4.88,2.95) | 1.58(1.16,2.00) | 11.48(13.87,8.90) | 5.23(6.65,3.97) | -1.30(-1.68,-0.91) |
| Argentina | male | 1426.48(1828.76,1051.43) | 2093.70(2745.17,1493.45) | 1.56(1.25,1.86) | 9.70(12.28,7.22) | 4.99(6.50,3.57) | -0.11(-0.35,0.14) |
| Armenia | male | 104.44(126.66,82.26) | 308.43(388.90,234.57) | 4.16(3.66,4.67) | 8.65(10.43,6.90) | 11.64(14.77,8.89) | 3.03(2.49,3.56) |
| Australia | male | 202.37(273.18,144.25) | 303.42(419.77,211.83) | 1.97(1.71,2.24) | 2.27(3.08,1.64) | 1.07(1.47,0.74) | -0.53(-0.79,-0.28) |
| Austria | male | 804.37(924.80,672.99) | 657.95(770.72,538.83) | -0.70(-0.80,-0.61) | 17.73(20.37,14.86) | 5.66(6.64,4.63) | -2.41(-2.49,-2.33) |
| Azerbaijan | male | 312.77(388.89,245.12) | 786.53(1076.82,565.74) | 2.57(2.30,2.84) | 15.16(18.44,12.12) | 14.65(19.80,10.39) | 0.38(0.06,0.71) |
| Bahamas | male | 11.59(14.16,9.10) | 19.77(26.15,14.53) | 1.70(1.43,1.98) | 15.42(18.75,12.14) | 6.22(8.15,4.59) | -1.64(-1.94,-1.34) |
| Bahrain | male | 2.63(4.01,1.62) | 9.31(15.45,5.27) | 4.35(4.03,4.67) | 3.09(4.81,1.88) | 1.29(2.04,0.75) | -2.30(-2.65,-1.95) |
| Bangladesh | male | 3785.82(5298.03,2517.76) | 4830.34(7047.84,3237.02) | 0.57(0.33,0.81) | 13.78(19.24,9.07) | 4.97(7.05,3.29) | -2.47(-2.70,-2.23) |
| Barbados | male | 10.72(12.88,8.57) | 16.43(21.38,11.93) | 1.06(0.71,1.41) | 9.45(11.38,7.52) | 4.34(5.59,3.21) | -1.28(-1.57,-0.99) |
| Belarus | male | 224.19(275.05,173.98) | 701.22(953.32,505.67) | 4.78(3.68,5.89) | 4.46(5.44,3.51) | 7.16(9.70,5.17) | 4.20(3.06,5.35) |
| Belgium | male | 529.46(597.45,453.01) | 680.59(800.01,560.44) | 0.85(0.77,0.94) | 8.51(9.59,7.34) | 5.05(5.91,4.15) | -0.62(-0.73,-0.50) |
| Belize | male | 4.54(5.57,3.55) | 20.92(26.35,16.44) | 5.11(4.70,5.52) | 9.58(11.81,7.48) | 8.94(11.21,7.03) | 0.84(0.36,1.32) |
| Benin | male | 170.56(249.22,110.98) | 329.68(488.22,209.53) | 2.10(1.99,2.22) | 17.63(25.75,11.36) | 8.80(12.80,5.81) | -1.13(-1.25,-1.01) |
| Bermuda | male | 3.12(3.75,2.49) | 2.79(3.60,2.08) | -0.28(-0.70,0.13) | 11.16(13.37,9.08) | 2.74(3.56,2.05) | -2.72(-3.10,-2.34) |
| Bhutan | male | 16.25(30.74,9.22) | 36.52(83.44,20.63) | 3.15(2.98,3.32) | 11.09(20.76,6.29) | 7.76(16.23,4.50) | 0.50(0.35,0.65) |
| Bolivia (Plurinational State of) | male | 369.16(536.33,233.32) | 950.00(1339.57,638.12) | 3.39(3.32,3.45) | 23.35(33.94,14.69) | 15.31(21.09,10.57) | -0.17(-0.26,-0.08) |
| Bosnia and Herzegovina | male | 209.88(256.10,163.91) | 204.78(277.25,147.43) | -0.55(-0.78,-0.32) | 10.55(12.72,8.33) | 4.79(6.45,3.50) | -1.51(-1.80,-1.23) |
| Botswana | male | 38.14(66.85,20.86) | 81.80(124.59,50.76) | 1.84(1.31,2.37) | 13.91(24.53,7.71) | 6.91(10.31,4.38) | -1.06(-1.63,-0.49) |
| Brazil | male | 8136.24(8750.56,7569.87) | 14927.93(16316.87,13719.17) | 2.29(2.18,2.40) | 16.95(18.21,15.71) | 7.23(7.92,6.64) | -0.71(-0.81,-0.60) |
| Brunei Darussalam | male | 2.67(3.83,1.76) | 5.86(8.33,3.82) | 2.24(2.02,2.46) | 4.25(6.05,2.76) | 2.33(3.19,1.63) | -1.48(-1.69,-1.28) |
| Bulgaria | male | 729.99(865.25,587.29) | 1075.49(1402.04,805.82) | 1.23(1.02,1.43) | 12.55(14.79,10.24) | 10.95(14.24,8.26) | 1.28(1.05,1.50) |
| Burkina Faso | male | 337.26(478.82,220.92) | 482.02(912.55,204.52) | 0.48(0.07,0.90) | 16.61(23.25,11.05) | 7.63(12.47,3.83) | -1.97(-2.37,-1.57) |
| Burundi | male | 235.02(382.44,128.59) | 346.03(712.82,173.34) | 0.94(0.65,1.23) | 21.63(35.00,12.01) | 9.41(17.17,5.23) | -2.08(-2.25,-1.90) |
| Cabo Verde | male | 8.08(13.08,4.93) | 24.91(33.77,17.45) | 2.78(2.27,3.30) | 9.09(14.58,5.65) | 7.08(9.69,4.93) | -0.21(-0.71,0.30) |
| Cambodia | male | 403.12(577.66,264.06) | 1088.23(1597.31,715.24) | 3.53(3.27,3.80) | 17.93(25.70,11.98) | 13.97(20.03,9.33) | 0.23(0.01,0.45) |
| Cameroon | male | 367.34(549.11,234.05) | 748.88(1142.89,437.25) | 2.32(2.19,2.46) | 16.17(24.06,10.26) | 7.75(11.78,4.64) | -1.26(-1.43,-1.09) |
| Canada | male | 703.22(909.76,511.97) | 1206.39(1568.56,865.47) | 1.93(1.81,2.06) | 4.88(6.27,3.55) | 2.58(3.35,1.86) | -0.76(-0.87,-0.65) |
| Central African Republic | male | 113.58(193.94,57.99) | 162.98(291.95,81.17) | 0.93(0.78,1.08) | 19.21(32.63,10.34) | 8.95(14.71,5.17) | -1.19(-1.27,-1.11) |
| Chad | male | 200.99(322.70,111.58) | 438.13(650.26,278.12) | 2.69(2.63,2.76) | 14.69(23.47,8.18) | 9.76(14.17,6.28) | -0.34(-0.50,-0.19) |
| Chile | male | 898.51(1151.84,658.63) | 1251.02(1610.31,900.89) | 1.40(1.18,1.63) | 18.46(23.67,13.58) | 6.73(8.75,4.90) | -1.51(-1.72,-1.30) |
| China | male | 21665.68(27654.97,16720.09) | 25146.14(32578.57,18543.58) | 0.44(0.34,0.53) | 4.81(6.05,3.75) | 1.50(1.90,1.15) | -2.21(-2.31,-2.12) |
| Colombia | male | 520.34(622.86,421.92) | 977.06(1368.98,670.27) | 1.71(1.44,1.98) | 5.98(7.13,4.85) | 2.77(3.83,1.94) | -1.83(-2.07,-1.59) |
| Comoros | male | 12.09(27.08,4.85) | 22.36(39.35,12.11) | 1.96(1.71,2.21) | 11.12(24.89,4.72) | 6.86(10.88,3.98) | -0.54(-0.73,-0.35) |
| Congo | male | 94.85(142.70,58.43) | 159.55(236.15,94.93) | 1.55(1.21,1.89) | 19.04(28.36,12.11) | 7.87(11.77,4.93) | -1.85(-2.03,-1.68) |
| Cook Islands | male | 0.33(0.48,0.22) | 0.43(0.62,0.27) | 0.95(0.84,1.07) | 4.68(6.67,3.11) | 1.97(2.83,1.28) | -0.81(-0.92,-0.70) |
| Costa Rica | male | 83.77(99.46,68.25) | 262.27(355.75,183.50) | 3.39(3.10,3.68) | 9.31(11.10,7.54) | 7.13(9.71,5.01) | -0.09(-0.38,0.19) |
| Côte d'Ivoire | male | 399.92(613.60,247.95) | 760.93(1162.33,482.55) | 1.95(1.73,2.17) | 16.99(25.39,10.79) | 8.28(12.50,5.20) | -1.25(-1.46,-1.04) |
| Croatia | male | 565.70(672.71,453.13) | 443.43(592.74,325.64) | -0.83(-1.01,-0.66) | 20.02(23.66,16.21) | 7.19(9.62,5.31) | -1.61(-1.82,-1.41) |
| Cuba | male | 278.72(334.99,224.83) | 715.34(952.51,527.32) | 3.34(3.11,3.57) | 5.51(6.61,4.45) | 4.89(6.51,3.58) | 1.31(1.08,1.54) |
| Cyprus | male | 28.73(38.95,19.69) | 45.74(57.33,35.49) | 1.31(1.20,1.42) | 7.81(10.66,5.28) | 3.40(4.25,2.68) | -1.69(-1.86,-1.53) |
| Czechia | male | 717.59(833.22,596.38) | 828.02(1080.01,627.88) | 1.02(0.83,1.21) | 12.28(14.25,10.27) | 6.39(8.32,4.92) | -0.44(-0.61,-0.27) |
| Democratic People's Republic of Korea | male | 492.16(757.85,283.35) | 862.03(1311.87,438.70) | 1.86(1.67,2.05) | 6.69(10.11,4.06) | 3.39(5.11,1.95) | -0.35(-0.52,-0.18) |
| Democratic Republic of the Congo | male | 1240.40(1836.09,776.23) | 2254.37(3533.30,1295.76) | 1.80(1.57,2.03) | 16.07(23.67,10.38) | 7.09(10.77,4.21) | -1.00(-1.10,-0.90) |
| Denmark | male | 268.20(309.75,221.61) | 312.65(376.89,245.70) | 0.07(-0.39,0.53) | 8.36(9.67,6.91) | 4.49(5.44,3.54) | -1.35(-1.81,-0.88) |
| Djibouti | male | 9.93(20.51,5.77) | 40.00(74.22,22.43) | 4.75(4.65,4.85) | 12.70(24.74,7.51) | 8.03(14.02,4.46) | -0.53(-0.62,-0.44) |
| Dominica | male | 2.75(3.46,2.11) | 3.33(4.40,2.43) | 0.41(0.20,0.62) | 9.88(12.42,7.54) | 5.12(6.66,3.77) | -1.26(-1.46,-1.06) |
| Dominican Republic | male | 362.57(447.62,283.28) | 834.53(1170.20,543.03) | 2.57(2.16,2.97) | 19.38(23.86,15.03) | 12.36(17.11,8.41) | -0.47(-0.92,-0.02) |
| Ecuador | male | 387.06(476.81,306.49) | 1076.62(1481.45,772.90) | 3.74(3.51,3.97) | 13.93(17.28,10.99) | 10.26(14.01,7.43) | 0.43(0.23,0.63) |
| Egypt | male | 1103.03(1672.60,692.09) | 2491.26(4100.56,1269.62) | 3.16(2.95,3.37) | 8.69(13.20,5.42) | 6.75(10.97,3.68) | 0.04(-0.19,0.27) |
| El Salvador | male | 243.47(297.59,193.47) | 380.47(525.08,267.03) | 1.10(0.83,1.37) | 16.44(20.07,13.08) | 9.15(12.67,6.40) | -0.67(-0.94,-0.41) |
| Equatorial Guinea | male | 13.23(23.16,6.67) | 15.53(26.99,8.29) | 0.24(-0.28,0.77) | 14.20(24.96,7.25) | 4.40(7.23,2.54) | -2.66(-2.96,-2.36) |
| Eritrea | male | 84.12(137.67,46.48) | 231.34(357.19,139.52) | 3.43(3.25,3.62) | 18.08(29.26,10.37) | 11.77(17.86,7.38) | -0.21(-0.41,-0.02) |
| Estonia | male | 38.06(46.79,30.09) | 99.91(134.94,71.38) | 3.32(2.55,4.10) | 4.71(5.75,3.75) | 6.71(9.03,4.81) | 2.93(2.12,3.76) |
| Eswatini | male | 18.75(30.02,11.27) | 36.86(54.45,23.12) | 2.39(1.97,2.82) | 13.49(21.19,8.35) | 7.52(10.93,4.87) | 0.41(0.10,0.72) |
| Ethiopia | male | 1849.78(2878.09,978.35) | 2440.83(3219.97,1829.26) | 0.69(0.52,0.86) | 16.87(25.73,9.20) | 8.21(10.39,6.49) | -1.42(-1.53,-1.31) |
| Fiji | male | 7.86(11.45,5.12) | 12.76(19.72,8.04) | 1.79(1.68,1.91) | 3.70(5.43,2.47) | 2.18(3.20,1.42) | -0.43(-0.54,-0.33) |
| Finland | male | 209.46(242.86,169.97) | 418.61(503.15,327.74) | 3.30(2.69,3.92) | 7.06(8.19,5.74) | 5.95(7.17,4.61) | 1.76(1.23,2.29) |
| France | male | 4621.74(5348.83,3849.97) | 3602.60(4314.47,2862.32) | -0.91(-1.00,-0.83) | 13.74(15.88,11.44) | 4.26(5.09,3.40) | -2.50(-2.61,-2.40) |
| Gabon | male | 50.90(80.42,29.95) | 70.15(106.19,43.08) | 0.92(0.71,1.12) | 19.76(30.80,11.80) | 8.03(12.29,5.13) | -1.39(-1.49,-1.29) |
| Gambia | male | 27.83(45.27,16.38) | 64.55(94.71,40.25) | 2.66(2.29,3.03) | 14.12(22.52,8.40) | 8.81(12.89,5.46) | -0.40(-0.67,-0.13) |
| Georgia | male | 468.14(573.96,364.13) | 546.67(687.58,416.01) | 0.93(0.67,1.19) | 5.99(6.94,5.14) | 12.77(16.04,9.75) | -0.36(-5.23,4.77) |
| Germany | male | 7309.81(8359.69,6111.01) | 6982.46(8246.86,5772.58) | -0.72(-0.91,-0.53) | 14.60(16.67,12.26) | 6.11(7.19,5.03) | -2.14(-2.34,-1.94) |
| Ghana | male | 636.68(996.19,380.39) | 1204.43(1749.20,763.28) | 2.11(1.98,2.24) | 20.09(31.81,11.78) | 9.33(13.09,6.07) | -0.85(-0.97,-0.73) |
| Greece | male | 541.52(640.08,437.68) | 446.51(533.37,364.62) | -0.69(-0.94,-0.43) | 7.96(9.38,6.46) | 2.90(3.46,2.36) | -1.73(-2.05,-1.41) |
| Greenland | male | 0.93(1.41,0.57) | 1.67(2.45,1.05) | 2.36(2.22,2.50) | 3.92(5.84,2.53) | 3.28(4.70,2.18) | 0.15(0.07,0.24) |
| Grenada | male | 4.33(5.23,3.42) | 6.85(8.36,5.40) | 1.76(1.55,1.96) | 15.08(18.25,11.87) | 7.11(8.63,5.62) | -0.79(-0.96,-0.62) |
| Guam | male | 2.88(4.05,1.97) | 4.51(6.62,2.97) | 1.47(1.36,1.58) | 6.84(9.54,4.83) | 2.97(4.25,1.99) | -1.30(-1.53,-1.07) |
| Guatemala | male | 549.29(691.65,419.02) | 1386.06(1862.94,993.13) | 2.99(2.77,3.21) | 26.02(32.50,19.97) | 16.15(21.59,11.60) | -0.27(-0.48,-0.07) |
| Guinea | male | 235.64(361.03,139.36) | 375.62(547.34,241.18) | 1.50(1.33,1.67) | 13.98(21.14,8.28) | 8.88(13.34,5.64) | -0.26(-0.40,-0.11) |
| Guinea-Bissau | male | 47.28(72.83,25.52) | 76.08(112.80,47.64) | 1.46(1.27,1.64) | 22.84(34.50,13.00) | 13.23(19.16,8.47) | -0.61(-0.71,-0.52) |
| Guyana | male | 70.40(88.69,54.40) | 91.32(122.73,65.81) | 1.26(0.98,1.54) | 34.21(42.87,26.67) | 15.95(21.15,11.52) | -0.46(-0.72,-0.19) |
| Haiti | male | 379.55(546.35,204.65) | 586.99(887.67,307.62) | 1.57(1.37,1.78) | 22.06(31.68,12.41) | 10.83(16.15,5.44) | -0.93(-1.11,-0.75) |
| Honduras | male | 204.03(275.16,142.70) | 584.07(865.52,355.89) | 3.97(3.73,4.21) | 19.12(25.80,13.56) | 16.60(24.70,10.04) | 0.42(0.17,0.68) |
| Hungary | male | 1814.70(2121.51,1504.41) | 1183.77(1510.67,907.43) | -2.68(-3.16,-2.19) | 29.32(34.29,24.27) | 9.55(12.21,7.29) | -3.42(-3.95,-2.88) |
| Iceland | male | 3.88(4.74,3.08) | 5.99(7.42,4.70) | 1.44(1.34,1.53) | 3.03(3.69,2.41) | 1.52(1.87,1.19) | -0.97(-1.05,-0.88) |
| India | male | 25061.49(30660.13,20808.99) | 47575.28(61551.03,37076.05) | 2.20(1.93,2.48) | 9.07(11.16,7.45) | 5.14(6.51,4.09) | -0.49(-0.77,-0.21) |
| Indonesia | male | 5121.42(6569.45,3924.41) | 8997.78(11807.99,6630.93) | 1.99(1.88,2.09) | 9.93(12.60,7.67) | 6.71(8.31,5.37) | -0.42(-0.53,-0.32) |
| Iran (Islamic Republic of) | male | 169.72(219.33,126.56) | 356.72(440.18,281.92) | 2.99(2.74,3.23) | 1.29(1.62,0.98) | 0.68(0.84,0.55) | -0.56(-0.80,-0.33) |
| Iraq | male | 62.55(108.08,35.89) | 149.28(233.24,88.79) | 2.87(2.76,2.99) | 1.62(2.77,0.93) | 0.87(1.32,0.52) | -0.85(-0.99,-0.71) |
| Ireland | male | 53.83(64.14,43.72) | 122.61(147.26,98.50) | 3.26(2.78,3.73) | 2.99(3.56,2.44) | 2.56(3.05,2.06) | 0.91(0.39,1.42) |
| Israel | male | 99.49(123.46,77.32) | 185.17(228.82,143.18) | 1.61(1.32,1.90) | 4.59(5.64,3.58) | 2.43(3.05,1.86) | -1.38(-1.67,-1.10) |
| Italy | male | 2826.49(3160.51,2520.50) | 1651.07(1896.05,1432.84) | -2.05(-2.24,-1.85) | 7.49(8.31,6.72) | 1.89(2.18,1.63) | -3.49(-3.67,-3.31) |
| Jamaica | male | 41.22(50.42,31.94) | 54.00(74.22,37.58) | 0.62(0.15,1.10) | 5.14(6.29,4.00) | 2.43(3.33,1.72) | -1.37(-1.84,-0.90) |
| Japan | male | 3497.82(3947.37,3100.50) | 3082.84(3656.29,2573.34) | -0.34(-0.47,-0.21) | 4.65(5.19,4.14) | 1.39(1.65,1.17) | -2.33(-2.49,-2.17) |
| Jordan | male | 10.02(17.17,5.96) | 35.09(54.14,21.46) | 4.09(3.75,4.43) | 1.53(2.60,0.93) | 0.81(1.24,0.50) | -1.36(-1.45,-1.26) |
| Kazakhstan | male | 458.24(561.82,359.93) | 1839.31(2439.69,1389.40) | 5.53(4.89,6.17) | 9.24(11.15,7.37) | 15.81(20.20,12.33) | 4.15(3.53,4.79) |
| Kenya | male | 877.82(1701.35,520.36) | 2401.35(3652.28,1652.93) | 3.76(3.63,3.88) | 21.86(42.16,12.96) | 15.25(22.62,10.63) | 0.32(0.13,0.51) |
| Kiribati | male | 2.88(4.77,1.30) | 3.48(6.51,1.40) | 0.71(0.45,0.96) | 14.44(22.93,7.38) | 5.97(10.11,2.94) | -1.59(-1.81,-1.37) |
| Kuwait | male | 3.26(4.91,2.04) | 13.58(20.96,8.09) | 5.48(4.78,6.19) | 0.77(1.15,0.49) | 0.61(0.95,0.37) | 0.74(-0.08,1.57) |
| Kyrgyzstan | male | 208.80(251.82,164.75) | 625.44(773.25,491.87) | 3.92(3.39,4.46) | 15.94(19.00,12.71) | 16.97(20.65,13.41) | 1.85(1.23,2.46) |
| Lao People's Democratic Republic | male | 111.47(171.07,63.64) | 199.02(318.42,113.94) | 1.73(1.42,2.04) | 9.90(15.09,5.60) | 5.56(8.56,3.29) | -0.92(-1.16,-0.69) |
| Latvia | male | 59.87(72.73,47.48) | 123.63(167.28,87.36) | 2.20(1.49,2.92) | 4.40(5.32,3.48) | 5.98(7.81,4.50) | 2.45(1.75,3.16) |
| Lebanon | male | 16.39(26.33,8.61) | 28.47(51.53,10.04) | 2.27(2.09,2.44) | 1.53(2.40,0.84) | 0.80(1.43,0.38) | -0.45(-0.71,-0.20) |
| Lesotho | male | 52.08(95.97,28.47) | 75.57(119.75,44.76) | 1.01(0.77,1.26) | 11.42(20.74,6.25) | 7.41(11.61,4.67) | 0.40(0.19,0.61) |
| Liberia | male | 112.39(164.37,72.79) | 160.60(243.57,94.96) | 0.74(0.52,0.97) | 18.80(27.36,12.26) | 10.08(14.67,6.24) | -1.44(-1.60,-1.28) |
| Libya | male | 17.74(31.52,8.66) | 37.68(63.09,18.72) | 2.62(2.36,2.88) | 1.85(3.31,0.90) | 1.05(1.70,0.58) | -0.73(-0.86,-0.60) |
| Lithuania | male | 92.64(110.52,73.04) | 279.08(367.58,209.76) | 4.65(3.44,5.87) | 5.06(6.03,4.01) | 9.26(12.22,7.01) | 4.64(3.47,5.83) |
| Luxembourg | male | 33.69(39.29,28.05) | 31.27(38.15,24.54) | -0.54(-0.67,-0.41) | 14.70(17.06,12.24) | 4.58(5.61,3.59) | -2.85(-2.97,-2.74) |
| Madagascar | male | 383.58(579.78,242.43) | 669.75(1037.42,412.02) | 1.87(1.72,2.03) | 14.42(21.50,9.17) | 8.41(12.49,5.43) | -0.79(-0.92,-0.65) |
| Malawi | male | 405.81(614.05,261.61) | 613.30(881.78,394.71) | 1.05(0.89,1.21) | 21.82(32.77,14.19) | 10.75(15.60,7.32) | -0.86(-1.12,-0.61) |
| Malaysia | male | 185.13(260.30,127.29) | 618.04(906.91,389.46) | 3.93(3.69,4.17) | 3.75(5.25,2.56) | 3.07(4.51,1.93) | 0.05(-0.22,0.33) |
| Maldives | male | 2.20(3.37,1.21) | 4.84(6.71,3.32) | 2.38(1.90,2.87) | 3.84(5.80,2.13) | 1.90(2.69,1.30) | -1.76(-1.96,-1.55) |
| Mali | male | 352.87(542.10,213.07) | 489.88(813.92,283.68) | 0.91(0.71,1.12) | 16.47(24.81,10.10) | 7.59(11.88,4.90) | -1.83(-2.01,-1.65) |
| Malta | male | 11.36(13.82,8.99) | 13.60(16.65,10.69) | 0.74(0.62,0.86) | 5.89(7.18,4.69) | 2.33(2.87,1.84) | -1.70(-1.79,-1.62) |
| Marshall Islands | male | 0.96(1.45,0.55) | 1.53(2.67,0.78) | 1.67(1.55,1.78) | 10.51(16.10,6.29) | 4.86(8.37,2.57) | -1.23(-1.43,-1.03) |
| Mauritania | male | 82.69(125.32,50.20) | 91.88(157.41,49.74) | 0.22(0.04,0.40) | 16.94(25.81,10.30) | 6.22(9.59,3.68) | -2.51(-2.64,-2.38) |
| Mauritius | male | 43.31(58.12,29.90) | 50.88(74.81,32.98) | -0.03(-0.40,0.34) | 11.06(14.80,7.70) | 3.47(5.01,2.33) | -2.76(-3.10,-2.42) |
| Mexico | male | 6643.10(7258.03,6051.48) | 13046.39(16405.01,10072.10) | 2.05(1.90,2.20) | 28.87(31.50,26.31) | 13.99(17.14,11.34) | -1.13(-1.28,-0.98) |
| Micronesia (Federated States of) | male | 2.70(4.25,1.45) | 3.05(5.56,1.35) | 0.45(0.25,0.64) | 10.29(15.92,5.86) | 4.84(8.38,2.34) | -1.04(-1.18,-0.90) |
| Monaco | male | 1.84(2.42,1.34) | 2.20(2.85,1.62) | 0.75(0.68,0.82) | 7.17(9.36,5.27) | 4.15(5.37,3.02) | -0.41(-0.47,-0.36) |
| Mongolia | male | 156.36(205.66,112.70) | 408.68(546.55,300.24) | 3.59(3.41,3.77) | 33.14(43.48,24.25) | 29.25(38.89,21.58) | 0.57(0.24,0.91) |
| Montenegro | male | 11.39(15.01,8.56) | 19.08(25.95,13.89) | 1.57(1.40,1.75) | 3.85(5.07,2.92) | 2.54(3.35,1.89) | 0.21(0.06,0.37) |
| Morocco | male | 112.84(178.30,60.62) | 249.60(389.50,144.47) | 2.68(2.30,3.06) | 1.78(2.85,0.95) | 1.18(1.78,0.72) | -0.29(-0.68,0.10) |
| Mozambique | male | 209.96(315.75,128.23) | 344.21(620.97,168.26) | 1.95(1.69,2.21) | 7.61(11.31,4.77) | 4.56(7.44,2.48) | -0.13(-0.45,0.20) |
| Myanmar | male | 2024.42(2996.55,1247.97) | 3247.00(4707.42,2136.82) | 1.64(1.51,1.77) | 15.35(22.73,9.48) | 7.68(10.94,5.11) | -0.53(-0.64,-0.41) |
| Namibia | male | 38.24(63.89,20.93) | 70.06(104.78,43.73) | 1.51(1.25,1.77) | 11.27(18.70,6.18) | 6.17(9.07,3.96) | -0.48(-0.71,-0.26) |
| Nauru | male | 0.26(0.45,0.11) | 0.23(0.42,0.10) | -0.61(-0.73,-0.50) | 9.63(16.22,4.28) | 5.21(8.53,2.54) | -0.42(-0.48,-0.35) |
| Nepal | male | 1129.57(1750.03,748.68) | 2324.55(3685.08,1572.76) | 2.88(2.61,3.15) | 20.82(32.58,13.62) | 14.11(20.80,9.64) | 0.38(0.10,0.67) |
| Netherlands | male | 375.10(436.88,308.19) | 484.22(598.53,380.71) | 0.55(0.27,0.84) | 4.59(5.38,3.76) | 2.30(2.81,1.83) | -1.52(-1.84,-1.20) |
| New Zealand | male | 23.55(27.88,19.62) | 35.85(43.61,29.57) | 1.39(1.33,1.46) | 1.36(1.60,1.14) | 0.67(0.81,0.56) | -1.09(-1.19,-1.00) |
| Nicaragua | male | 138.48(167.73,109.95) | 484.87(639.59,358.86) | 4.44(4.26,4.62) | 18.25(22.26,14.60) | 13.63(17.63,10.15) | 0.92(0.74,1.10) |
| Niger | male | 238.55(392.97,139.61) | 490.59(848.29,261.48) | 2.20(2.02,2.38) | 15.13(24.30,8.82) | 8.31(13.43,4.90) | -1.21(-1.38,-1.03) |
| Nigeria | male | 4381.66(6838.63,2583.32) | 6502.31(10834.76,3922.20) | 1.57(1.42,1.73) | 18.24(27.66,10.99) | 9.91(15.00,6.47) | -0.29(-0.46,-0.13) |
| Niue | male | 0.07(0.11,0.04) | 0.06(0.09,0.04) | -0.97(-1.19,-0.74) | 7.68(11.62,4.65) | 3.66(5.31,2.33) | -0.94(-1.07,-0.80) |
| North Macedonia | male | 59.19(73.20,45.92) | 112.58(152.94,81.00) | 2.07(1.96,2.18) | 6.24(7.71,4.84) | 4.56(6.11,3.35) | 0.29(0.18,0.40) |
| Northern Mariana Islands | male | 1.45(2.11,0.92) | 1.87(2.76,1.21) | 0.88(0.56,1.20) | 10.04(14.31,6.88) | 4.25(5.93,2.88) | -1.89(-2.06,-1.72) |
| Norway | male | 101.17(113.63,89.62) | 103.73(121.08,90.43) | 0.21(0.14,0.28) | 3.79(4.24,3.39) | 1.69(1.96,1.47) | -1.45(-1.56,-1.33) |
| Oman | male | 7.71(13.08,4.14) | 16.58(29.04,8.84) | 3.39(3.10,3.68) | 2.22(3.69,1.21) | 1.32(2.05,0.78) | 0.11(-0.25,0.47) |
| Pakistan | male | 1362.83(2420.13,667.41) | 1982.09(3405.46,1138.43) | 1.32(1.28,1.37) | 4.17(7.46,2.01) | 3.91(6.15,2.44) | -1.03(-1.19,-0.87) |
| Palau | male | 0.36(0.60,0.18) | 0.68(1.16,0.34) | 2.08(1.73,2.44) | 6.85(11.37,3.61) | 3.47(5.72,1.87) | -0.55(-0.62,-0.48) |
| Palestine | male | 8.36(15.89,4.34) | 16.91(25.85,10.22) | 2.79(2.56,3.01) | 2.27(4.17,1.24) | 1.19(1.82,0.75) | -0.77(-0.96,-0.57) |
| Panama | male | 52.48(63.07,41.21) | 131.26(181.86,92.84) | 3.45(3.21,3.68) | 6.91(8.33,5.43) | 4.51(6.19,3.21) | -0.03(-0.24,0.17) |
| Papua New Guinea | male | 43.66(64.69,27.79) | 113.41(176.76,67.44) | 3.61(3.41,3.81) | 3.63(5.38,2.34) | 2.05(3.08,1.25) | 0.08(-0.02,0.18) |
| Paraguay | male | 91.55(111.84,71.72) | 303.63(413.45,215.38) | 4.16(3.95,4.37) | 8.31(10.16,6.46) | 6.03(8.20,4.30) | 0.88(0.67,1.09) |
| Peru | male | 1097.97(1418.52,829.00) | 2045.13(2906.26,1353.70) | 2.16(1.98,2.35) | 17.87(23.01,13.46) | 8.80(12.52,5.82) | -1.07(-1.25,-0.89) |
| Philippines | male | 1002.48(1359.34,733.12) | 2296.46(3160.43,1626.44) | 2.81(2.66,2.96) | 5.96(8.16,4.27) | 3.59(4.61,2.73) | -0.26(-0.43,-0.10) |
| Poland | male | 1688.45(1835.41,1540.94) | 3025.75(3867.02,2352.78) | 2.26(1.95,2.57) | 9.13(9.91,8.33) | 6.73(8.29,5.44) | 0.69(0.40,0.98) |
| Portugal | male | 1302.53(1516.60,1076.23) | 782.58(927.59,636.40) | -1.99(-2.11,-1.86) | 21.94(25.48,18.31) | 5.04(5.96,4.09) | -3.40(-3.54,-3.26) |
| Puerto Rico | male | 355.19(418.63,287.05) | 325.32(435.46,233.84) | -1.11(-1.47,-0.75) | 21.43(25.38,17.26) | 6.66(9.07,4.77) | -2.93(-3.31,-2.56) |
| Qatar | male | 2.12(3.50,1.15) | 12.69(21.03,6.93) | 6.57(6.37,6.78) | 3.31(5.29,1.97) | 2.33(3.78,1.29) | -0.99(-1.32,-0.65) |
| Republic of Korea | male | 4749.49(5751.48,3680.40) | 3769.88(4625.73,2975.93) | -1.94(-2.34,-1.55) | 31.18(37.79,24.71) | 5.46(6.71,4.34) | -5.39(-5.80,-4.98) |
| Republic of Moldova | male | 689.04(793.04,581.86) | 785.14(954.58,622.32) | 0.51(0.12,0.90) | 35.86(41.18,30.30) | 23.42(28.65,18.47) | -0.27(-0.66,0.13) |
| Romania | male | 2318.87(2744.16,1889.63) | 3402.61(4378.38,2595.49) | 0.76(0.34,1.19) | 17.76(20.84,14.62) | 15.10(19.42,11.52) | 0.28(-0.15,0.71) |
| Russian Federation | male | 3962.43(4474.22,3512.54) | 12783.85(15941.87,10173.40) | 3.96(3.10,4.84) | 5.80(6.50,5.15) | 9.17(10.94,7.58) | 3.14(2.32,3.96) |
| Rwanda | male | 360.51(514.20,231.26) | 406.14(624.23,251.38) | 0.03(-0.47,0.54) | 26.87(38.15,17.50) | 9.15(13.38,5.95) | -2.97(-3.35,-2.58) |
| Saint Kitts and Nevis | male | 3.28(4.03,2.55) | 4.82(6.37,3.41) | 0.72(0.04,1.42) | 21.84(26.76,17.22) | 7.85(10.27,5.72) | -2.22(-2.68,-1.76) |
| Saint Lucia | male | 5.82(6.96,4.71) | 10.50(13.46,8.12) | 1.95(1.52,2.37) | 15.16(18.08,12.28) | 6.47(8.17,4.98) | -1.66(-2.07,-1.25) |
| Saint Vincent and the Grenadines | male | 3.56(4.40,2.74) | 7.35(9.29,5.68) | 2.43(1.93,2.93) | 11.22(13.86,8.70) | 6.25(7.90,4.83) | -0.50(-0.93,-0.07) |
| Samoa | male | 3.39(5.63,2.09) | 4.09(6.02,2.57) | 0.73(0.59,0.87) | 7.47(12.29,4.69) | 3.46(4.97,2.24) | -1.21(-1.33,-1.08) |
| San Marino | male | 1.28(1.66,0.96) | 2.16(3.14,1.39) | 1.86(1.80,1.91) | 8.93(11.46,6.74) | 5.51(8.25,3.41) | -0.54(-0.65,-0.44) |
| Sao Tome and Principe | male | 6.90(10.90,3.92) | 11.01(16.95,7.43) | 1.27(1.06,1.48) | 22.53(34.98,13.19) | 12.52(18.98,8.12) | -0.50(-0.69,-0.31) |
| Saudi Arabia | male | 110.56(188.95,58.95) | 189.72(293.37,112.37) | 1.29(1.01,1.57) | 3.65(6.33,1.96) | 1.73(2.71,1.04) | -1.84(-2.02,-1.65) |
| Senegal | male | 230.51(377.63,137.13) | 415.49(678.55,251.68) | 2.10(1.86,2.34) | 13.71(22.28,8.20) | 7.09(10.99,4.43) | -0.80(-1.01,-0.59) |
| Serbia | male | 489.09(633.70,366.75) | 526.33(699.83,383.38) | -0.12(-0.31,0.08) | 8.63(11.06,6.63) | 4.60(6.18,3.42) | -0.70(-0.88,-0.52) |
| Seychelles | male | 2.50(3.57,1.64) | 6.16(8.64,4.03) | 2.92(2.72,3.12) | 9.64(13.84,6.32) | 6.49(8.94,4.43) | -0.26(-0.56,0.05) |
| Sierra Leone | male | 171.17(260.26,105.63) | 196.35(303.51,117.40) | -0.04(-0.37,0.29) | 17.38(26.24,10.80) | 7.19(10.72,4.51) | -2.40(-2.69,-2.10) |
| Singapore | male | 23.68(32.90,16.54) | 29.08(40.96,19.43) | 0.65(0.25,1.04) | 2.18(3.06,1.51) | 0.52(0.73,0.36) | -3.78(-4.03,-3.53) |
| Slovakia | male | 510.95(620.77,408.75) | 619.69(852.76,450.01) | 1.25(0.99,1.51) | 19.38(23.45,15.51) | 9.68(13.11,7.08) | -0.26(-0.50,-0.02) |
| Slovenia | male | 214.49(292.51,153.91) | 210.41(289.53,151.39) | -0.17(-0.42,0.08) | 20.33(27.76,14.68) | 7.14(9.86,5.16) | -2.03(-2.29,-1.77) |
| Solomon Islands | male | 9.12(14.52,5.39) | 18.98(27.69,12.13) | 2.65(2.56,2.74) | 9.69(15.50,5.75) | 5.83(8.32,3.86) | -0.20(-0.29,-0.12) |
| Somalia | male | 232.66(372.88,127.44) | 493.43(789.17,277.94) | 2.60(2.52,2.68) | 18.37(28.48,10.58) | 10.11(16.28,5.91) | -0.48(-0.59,-0.38) |
| South Africa | male | 651.50(900.50,465.06) | 988.50(1191.48,797.28) | 0.94(0.40,1.49) | 6.71(9.31,4.76) | 2.91(3.49,2.37) | -1.44(-1.96,-0.93) |
| South Sudan | male | 189.45(351.13,108.62) | 250.99(442.64,140.28) | 1.04(0.94,1.14) | 13.88(25.22,8.08) | 8.63(14.40,5.15) | -0.35(-0.41,-0.28) |
| Spain | male | 3458.67(4079.83,2834.43) | 2451.80(2938.60,1967.74) | -1.20(-1.32,-1.08) | 15.01(17.57,12.37) | 3.93(4.69,3.18) | -3.08(-3.21,-2.95) |
| Sri Lanka | male | 583.79(792.54,399.60) | 834.81(1241.96,532.09) | 0.31(-0.37,1.00) | 9.58(12.92,6.65) | 4.01(5.83,2.58) | -1.83(-2.46,-1.21) |
| Sudan | male | 123.02(213.83,56.92) | 205.42(394.03,94.85) | 1.50(1.40,1.60) | 2.65(4.71,1.24) | 1.56(2.86,0.79) | -0.87(-0.92,-0.81) |
| Suriname | male | 19.80(24.02,15.42) | 40.06(52.11,29.46) | 2.30(2.10,2.50) | 15.15(18.30,11.86) | 8.63(11.22,6.36) | -0.64(-0.83,-0.44) |
| Sweden | male | 295.33(321.39,270.39) | 368.22(414.49,323.98) | 1.02(0.86,1.18) | 4.98(5.40,4.57) | 2.68(3.01,2.37) | -0.44(-0.60,-0.28) |
| Switzerland | male | 302.23(353.04,248.91) | 322.75(393.77,253.61) | 0.11(0.01,0.21) | 7.10(8.28,5.84) | 2.93(3.57,2.31) | -1.86(-2.01,-1.71) |
| Syrian Arab Republic | male | 53.07(82.40,30.26) | 104.15(168.69,55.86) | 2.54(2.34,2.75) | 1.97(3.07,1.11) | 1.32(2.13,0.77) | -0.37(-0.55,-0.19) |
| Taiwan (Province of China) | male | 937.35(1190.67,725.06) | 1399.48(1981.59,927.64) | 1.05(0.68,1.42) | 11.03(14.12,8.57) | 4.54(6.46,3.03) | -1.59(-1.89,-1.28) |
| Tajikistan | male | 157.25(199.23,119.70) | 502.74(664.46,370.41) | 4.02(3.81,4.22) | 11.88(15.11,9.11) | 14.50(18.88,10.81) | 1.63(1.51,1.76) |
| Thailand | male | 1669.66(2267.34,1167.63) | 4267.75(6394.81,2730.90) | 3.63(3.45,3.81) | 8.53(11.55,6.04) | 5.59(8.22,3.69) | 0.37(0.22,0.52) |
| Timor-Leste | male | 12.21(24.38,5.93) | 31.03(54.91,14.78) | 3.20(2.93,3.47) | 6.42(12.23,3.00) | 5.06(8.54,2.68) | 0.24(-0.01,0.49) |
| Togo | male | 100.27(148.79,64.15) | 216.05(333.03,131.68) | 2.22(1.95,2.50) | 16.20(24.15,10.34) | 7.55(11.18,4.85) | -1.50(-1.81,-1.18) |
| Tokelau | male | 0.03(0.05,0.02) | 0.02(0.04,0.02) | -1.21(-1.51,-0.90) | 5.64(8.71,3.58) | 2.70(3.99,1.69) | -1.53(-1.61,-1.46) |
| Tonga | male | 2.34(3.39,1.49) | 2.90(4.22,1.85) | 0.75(0.49,1.00) | 8.33(12.10,5.40) | 4.66(6.65,3.00) | -0.31(-0.56,-0.07) |
| Trinidad and Tobago | male | 45.14(53.59,36.26) | 62.85(89.25,43.19) | 1.14(0.90,1.39) | 10.86(12.93,8.79) | 4.33(6.04,2.99) | -1.61(-1.87,-1.34) |
| Tunisia | male | 41.84(70.59,23.73) | 80.12(139.91,42.95) | 2.26(2.23,2.30) | 1.72(2.80,0.98) | 0.90(1.53,0.50) | -0.72(-0.77,-0.66) |
| Turkey | male | 237.79(389.94,135.97) | 437.04(668.58,267.54) | 2.23(2.00,2.46) | 1.34(2.18,0.78) | 0.72(1.09,0.44) | -0.56(-0.77,-0.36) |
| Turkmenistan | male | 152.35(185.71,119.75) | 664.99(871.12,488.45) | 5.42(5.09,5.74) | 17.20(20.76,13.76) | 20.80(26.92,15.44) | 2.12(1.83,2.42) |
| Tuvalu | male | 0.29(0.46,0.15) | 0.32(0.50,0.18) | 0.47(0.20,0.75) | 9.00(14.27,5.11) | 3.99(6.36,2.31) | -1.23(-1.43,-1.02) |
| Uganda | male | 521.78(806.55,325.07) | 874.14(1285.95,552.81) | 1.37(1.16,1.58) | 16.20(25.14,10.14) | 8.39(11.87,5.53) | -1.01(-1.21,-0.81) |
| Ukraine | male | 2052.41(2351.10,1805.93) | 6108.61(7736.48,4694.88) | 3.62(2.88,4.36) | 7.39(8.35,6.52) | 12.81(15.93,10.16) | 3.70(2.94,4.46) |
| United Arab Emirates | male | 4.45(7.97,2.47) | 39.70(67.93,19.52) | 7.28(6.88,7.68) | 1.67(2.99,0.86) | 1.07(1.88,0.53) | -1.23(-1.64,-0.82) |
| United Kingdom | male | 1626.72(1764.23,1463.03) | 3644.73(3966.73,3327.77) | 2.87(2.39,3.36) | 4.57(4.94,4.14) | 5.16(5.63,4.71) | 1.64(1.11,2.16) |
| United Republic of Tanzania | male | 821.15(1193.86,546.64) | 1378.94(2067.84,850.65) | 1.76(1.54,1.97) | 14.92(21.29,10.07) | 7.57(11.05,4.72) | -0.98(-1.26,-0.69) |
| United States of America | male | 7517.59(8475.51,6588.31) | 12103.29(14218.61,10254.87) | 2.09(1.92,2.25) | 5.65(6.36,4.94) | 3.37(3.95,2.86) | -0.13(-0.25,-0.01) |
| United States Virgin Islands | male | 5.85(7.46,4.32) | 11.56(14.88,8.40) | 2.96(2.58,3.33) | 13.66(17.32,10.20) | 8.16(10.55,6.07) | 0.65(0.29,1.01) |
| Uruguay | male | 117.47(156.54,84.42) | 109.81(145.10,78.49) | -0.66(-0.81,-0.51) | 6.88(9.05,5.00) | 2.73(3.66,1.96) | -1.47(-1.61,-1.34) |
| Uzbekistan | male | 782.75(955.65,612.62) | 3184.23(4032.75,2436.23) | 4.25(3.81,4.70) | 15.12(18.25,11.91) | 21.05(26.18,16.16) | 1.66(1.12,2.20) |
| Vanuatu | male | 3.67(6.22,2.06) | 7.48(12.14,3.84) | 2.43(2.27,2.60) | 9.03(15.46,5.27) | 4.93(7.85,2.70) | -0.74(-0.86,-0.61) |
| Venezuela (Bolivarian Republic of) | male | 629.41(745.94,512.98) | 1583.20(2206.62,1102.04) | 3.16(2.85,3.47) | 12.75(15.23,10.33) | 6.33(8.74,4.44) | -0.70(-0.99,-0.41) |
| Viet Nam | male | 2111.09(3262.50,1246.64) | 4363.89(6277.86,2788.49) | 2.31(1.71,2.92) | 12.18(18.60,7.24) | 5.48(7.76,3.65) | -1.06(-1.60,-0.53) |
| Yemen | male | 60.38(108.28,28.43) | 133.21(230.00,68.16) | 2.68(2.63,2.73) | 2.68(4.96,1.21) | 1.49(2.50,0.82) | -0.93(-0.99,-0.88) |
| Zambia | male | 360.88(567.87,227.02) | 774.14(1097.17,513.68) | 2.24(2.03,2.46) | 23.92(37.18,15.23) | 14.35(20.42,9.54) | -0.46(-0.70,-0.21) |
| Zimbabwe | male | 239.63(338.58,153.60) | 352.67(557.82,210.20) | 0.86(0.62,1.11) | 12.00(16.91,7.84) | 6.55(10.33,3.97) | -0.32(-0.58,-0.05) |
| Afghanistan | female | 51.55(89.32,23.71) | 81.63(148.59,36.24) | 1.14(0.94,1.33) | 1.53(2.53,0.72) | 1.32(2.25,0.66) | -0.78(-0.97,-0.59) |
| Albania | female | 35.80(43.61,27.28) | 44.92(63.29,30.17) | 0.89(0.62,1.16) | 3.39(4.17,2.54) | 1.97(2.78,1.33) | -1.76(-2.00,-1.51) |
| Algeria | female | 43.09(68.59,25.25) | 82.21(126.51,50.51) | 2.03(1.84,2.22) | 0.82(1.27,0.48) | 0.57(0.88,0.35) | -1.25(-1.39,-1.11) |
| American Samoa | female | 0.15(0.22,0.10) | 0.30(0.45,0.19) | 2.36(2.13,2.59) | 1.33(1.94,0.86) | 1.21(1.80,0.77) | -0.28(-0.58,0.02) |
| Andorra | female | 0.69(1.14,0.39) | 1.58(2.31,1.04) | 2.65(2.53,2.78) | 2.60(4.27,1.48) | 2.26(3.31,1.49) | -0.56(-0.73,-0.39) |
| Angola | female | 97.40(166.54,51.82) | 258.78(401.55,154.12) | 3.41(3.27,3.56) | 4.70(8.02,2.52) | 4.09(6.44,2.48) | -0.53(-0.60,-0.46) |
| Antigua and Barbuda | female | 0.81(1.07,0.61) | 1.53(2.03,1.09) | 1.82(1.48,2.16) | 2.74(3.56,2.06) | 2.85(3.78,2.03) | -0.30(-0.58,-0.02) |
| Argentina | female | 389.64(524.26,280.65) | 525.85(720.27,365.32) | 1.26(0.96,1.56) | 2.21(2.94,1.60) | 1.76(2.38,1.21) | -0.58(-0.88,-0.28) |
| Armenia | female | 45.56(56.84,34.56) | 161.09(214.12,116.41) | 5.37(4.74,6.00) | 3.04(3.83,2.30) | 6.63(8.77,4.84) | 3.67(3.01,4.35) |
| Australia | female | 63.96(87.88,44.96) | 100.66(140.68,67.70) | 2.14(1.79,2.50) | 0.63(0.87,0.44) | 0.50(0.69,0.34) | -0.30(-0.66,0.07) |
| Austria | female | 282.48(339.21,228.85) | 227.29(284.49,178.13) | -0.87(-0.93,-0.81) | 4.67(5.55,3.74) | 2.63(3.20,2.06) | -2.19(-2.26,-2.11) |
| Azerbaijan | female | 213.07(266.81,162.31) | 457.68(662.63,296.32) | 1.86(1.44,2.28) | 7.27(9.12,5.53) | 10.46(15.46,6.44) | 0.58(0.02,1.15) |
| Bahamas | female | 3.94(4.92,3.02) | 6.17(8.23,4.38) | 1.44(1.19,1.70) | 4.48(5.63,3.40) | 2.84(3.80,2.04) | -1.64(-1.87,-1.40) |
| Bahrain | female | 0.87(1.39,0.50) | 2.09(3.36,1.17) | 2.71(2.45,2.96) | 1.26(1.96,0.75) | 0.71(1.14,0.41) | -2.22(-2.47,-1.97) |
| Bangladesh | female | 952.91(1464.55,565.40) | 1584.95(2469.73,956.00) | 1.88(1.65,2.11) | 4.17(6.45,2.46) | 2.44(3.80,1.48) | -1.93(-2.19,-1.67) |
| Barbados | female | 3.16(4.15,2.31) | 4.29(5.84,3.01) | 1.17(0.95,1.39) | 1.99(2.57,1.49) | 1.61(2.19,1.13) | -0.67(-0.81,-0.52) |
| Belarus | female | 115.16(147.37,85.33) | 351.92(491.90,237.58) | 4.75(3.69,5.83) | 1.46(1.85,1.10) | 4.10(5.68,2.76) | 4.43(3.20,5.67) |
| Belgium | female | 313.04(373.83,254.87) | 342.67(429.55,269.11) | 0.21(0.15,0.28) | 3.88(4.55,3.21) | 3.06(3.71,2.43) | -0.98(-1.07,-0.89) |
| Belize | female | 2.20(2.81,1.66) | 5.87(7.61,4.24) | 3.59(3.45,3.74) | 4.76(6.07,3.57) | 4.13(5.37,2.99) | -0.39(-0.62,-0.16) |
| Benin | female | 60.69(92.12,39.58) | 122.37(183.25,80.05) | 2.30(2.18,2.42) | 5.93(9.01,3.84) | 4.69(7.09,3.07) | -0.94(-1.03,-0.85) |
| Bermuda | female | 1.02(1.32,0.76) | 0.60(0.85,0.41) | -2.59(-3.10,-2.07) | 2.91(3.74,2.17) | 0.83(1.17,0.57) | -5.07(-5.56,-4.59) |
| Bhutan | female | 4.85(7.60,2.76) | 8.91(13.96,5.52) | 2.04(1.87,2.20) | 3.67(5.68,2.09) | 3.27(5.15,1.99) | -0.51(-0.65,-0.37) |
| Bolivia (Plurinational State of) | female | 161.08(249.70,89.71) | 406.45(587.55,262.41) | 3.23(3.16,3.30) | 9.37(14.41,5.36) | 8.97(12.89,5.93) | -0.26(-0.31,-0.21) |
| Bosnia and Herzegovina | female | 85.38(105.81,65.68) | 75.00(103.24,52.65) | -1.10(-1.38,-0.81) | 3.65(4.52,2.82) | 2.28(3.14,1.61) | -2.21(-2.56,-1.86) |
| Botswana | female | 11.82(21.60,6.04) | 24.50(39.64,13.48) | 2.44(2.25,2.64) | 3.64(6.51,1.88) | 3.00(4.79,1.64) | -0.67(-0.91,-0.43) |
| Brazil | female | 1397.79(1596.20,1211.39) | 2713.89(3181.99,2285.55) | 2.44(2.30,2.58) | 2.92(3.35,2.53) | 2.08(2.44,1.75) | -1.06(-1.20,-0.92) |
| Brunei Darussalam | female | 1.15(1.74,0.71) | 2.49(3.61,1.64) | 2.10(1.73,2.47) | 2.15(3.25,1.34) | 1.53(2.17,1.02) | -1.77(-2.07,-1.48) |
| Bulgaria | female | 213.04(254.37,170.12) | 268.98(350.71,200.88) | 0.64(0.52,0.76) | 3.21(3.81,2.60) | 3.77(5.01,2.80) | 0.43(0.31,0.54) |
| Burkina Faso | female | 138.03(197.69,90.37) | 211.56(354.54,71.16) | 0.93(0.57,1.29) | 6.45(9.22,4.29) | 4.37(7.51,1.42) | -1.87(-2.18,-1.55) |
| Burundi | female | 95.35(150.19,55.03) | 106.67(177.64,48.36) | -0.32(-0.70,0.07) | 7.52(11.72,4.39) | 4.93(8.21,2.16) | -2.08(-2.35,-1.81) |
| Cabo Verde | female | 3.73(5.99,2.30) | 6.68(9.81,4.34) | 1.65(1.35,1.96) | 2.89(4.61,1.79) | 2.72(3.98,1.76) | -0.57(-0.86,-0.28) |
| Cambodia | female | 286.85(413.86,188.81) | 668.58(979.40,432.57) | 2.85(2.69,3.00) | 10.55(15.21,6.96) | 9.69(14.16,6.34) | -0.46(-0.59,-0.33) |
| Cameroon | female | 132.82(188.85,88.12) | 243.02(405.93,120.75) | 2.03(1.91,2.15) | 5.87(8.31,3.90) | 3.92(6.52,1.92) | -1.47(-1.59,-1.36) |
| Canada | female | 246.04(339.22,172.06) | 463.15(627.48,325.36) | 2.29(2.23,2.35) | 1.41(1.95,0.99) | 1.34(1.80,0.95) | -0.11(-0.19,-0.03) |
| Central African Republic | female | 25.54(41.37,14.07) | 52.61(91.55,28.47) | 2.34(2.14,2.54) | 3.82(6.14,2.12) | 4.19(7.26,2.32) | 0.16(-0.10,0.41) |
| Chad | female | 83.84(130.23,48.69) | 139.37(204.99,88.68) | 1.65(1.56,1.74) | 5.80(8.96,3.33) | 5.27(7.83,3.34) | -0.38(-0.41,-0.34) |
| Chile | female | 264.65(356.04,184.25) | 362.80(499.53,254.28) | 1.31(1.10,1.53) | 4.77(6.39,3.31) | 2.77(3.82,1.95) | -1.68(-1.85,-1.50) |
| China | female | 5774.88(7444.51,4316.56) | 5402.25(7073.65,3973.70) | -0.51(-0.71,-0.32) | 1.34(1.71,1.00) | 0.52(0.68,0.39) | -3.48(-3.65,-3.31) |
| Colombia | female | 225.91(282.23,175.54) | 496.39(700.66,337.68) | 2.38(2.22,2.54) | 2.54(3.22,1.96) | 1.70(2.37,1.15) | -1.83(-1.99,-1.67) |
| Comoros | female | 5.30(9.08,2.30) | 11.13(18.15,5.17) | 2.38(2.18,2.59) | 4.71(8.09,2.13) | 4.28(7.02,1.97) | -0.48(-0.66,-0.30) |
| Congo | female | 25.28(39.81,14.83) | 57.79(94.80,33.14) | 2.90(2.65,3.15) | 4.20(6.57,2.55) | 4.13(6.80,2.39) | 0.00(-0.32,0.33) |
| Cook Islands | female | 0.04(0.06,0.02) | 0.06(0.09,0.03) | 1.79(1.59,1.99) | 0.61(0.93,0.36) | 0.45(0.69,0.27) | -0.83(-1.02,-0.63) |
| Costa Rica | female | 32.80(41.40,25.09) | 109.70(151.68,75.29) | 3.67(3.36,3.98) | 3.63(4.61,2.78) | 3.93(5.46,2.69) | -0.29(-0.58,0.00) |
| Côte d'Ivoire | female | 90.82(137.07,57.80) | 198.11(325.04,114.67) | 2.78(2.61,2.95) | 4.97(7.53,3.19) | 3.90(6.38,2.25) | -0.66(-0.76,-0.56) |
| Croatia | female | 226.35(270.77,182.51) | 126.09(167.39,91.25) | -2.31(-2.51,-2.11) | 6.18(7.36,5.01) | 2.74(3.68,2.00) | -3.11(-3.32,-2.90) |
| Cuba | female | 137.18(175.91,103.01) | 186.53(257.01,130.29) | 0.85(0.53,1.18) | 2.63(3.38,1.98) | 1.88(2.60,1.31) | -1.37(-1.71,-1.04) |
| Cyprus | female | 14.39(21.41,9.13) | 17.22(23.05,12.55) | 0.17(0.01,0.33) | 3.80(5.70,2.38) | 1.79(2.37,1.32) | -3.09(-3.28,-2.90) |
| Czechia | female | 268.04(317.23,223.92) | 333.72(431.85,255.57) | 1.12(0.98,1.27) | 3.47(4.06,2.91) | 3.40(4.43,2.57) | 0.20(0.08,0.32) |
| Democratic People's Republic of Korea | female | 164.99(257.33,90.91) | 256.28(398.07,141.48) | 1.47(1.35,1.59) | 1.69(2.61,0.95) | 1.37(2.14,0.75) | -0.72(-0.85,-0.58) |
| Democratic Republic of the Congo | female | 274.41(420.08,169.51) | 499.65(771.18,304.36) | 1.84(1.72,1.97) | 3.13(4.79,1.96) | 2.49(3.86,1.50) | -0.93(-1.02,-0.84) |
| Denmark | female | 101.62(122.26,81.03) | 127.87(162.07,97.41) | 0.12(-0.42,0.66) | 2.87(3.42,2.30) | 2.46(3.10,1.89) | -1.26(-1.81,-0.71) |
| Djibouti | female | 3.16(5.15,1.84) | 10.07(18.31,4.89) | 3.86(3.74,3.98) | 4.72(7.79,2.80) | 3.84(6.94,1.92) | -0.88(-0.97,-0.79) |
| Dominica | female | 1.56(2.07,1.13) | 1.21(1.66,0.83) | -1.08(-1.28,-0.88) | 3.89(5.09,2.84) | 2.65(3.61,1.83) | -1.35(-1.52,-1.17) |
| Dominican Republic | female | 154.72(199.97,113.85) | 317.42(442.73,215.33) | 2.14(1.81,2.47) | 8.26(10.71,6.06) | 6.63(9.26,4.50) | -1.03(-1.47,-0.60) |
| Ecuador | female | 130.20(174.64,96.65) | 458.76(648.10,317.37) | 4.99(4.65,5.34) | 4.85(6.59,3.58) | 5.97(8.39,4.11) | 1.27(0.94,1.61) |
| Egypt | female | 639.61(995.34,396.37) | 1104.02(1957.27,581.43) | 2.28(2.07,2.48) | 5.45(8.33,3.31) | 5.34(8.95,2.87) | 0.45(0.24,0.66) |
| El Salvador | female | 79.61(103.10,58.70) | 159.29(225.18,102.82) | 2.70(2.50,2.89) | 4.96(6.45,3.66) | 4.66(6.61,3.01) | 0.07(-0.13,0.26) |
| Equatorial Guinea | female | 3.37(5.83,1.67) | 6.44(11.25,3.34) | 2.06(1.92,2.21) | 2.91(5.01,1.42) | 2.29(3.97,1.20) | -1.05(-1.26,-0.84) |
| Eritrea | female | 35.48(62.73,16.88) | 106.62(169.31,64.26) | 4.09(3.94,4.23) | 6.22(10.78,3.02) | 7.19(11.41,4.42) | 0.60(0.49,0.71) |
| Estonia | female | 19.16(24.94,14.02) | 35.54(48.81,24.76) | 1.65(0.75,2.56) | 1.60(2.08,1.18) | 3.09(4.25,2.13) | 1.71(0.72,2.70) |
| Eswatini | female | 5.54(8.85,3.51) | 10.45(17.07,5.36) | 2.41(1.95,2.87) | 3.25(5.18,2.02) | 3.01(4.88,1.58) | 0.10(-0.31,0.50) |
| Ethiopia | female | 735.65(1139.60,419.35) | 906.99(1200.24,658.29) | 0.14(-0.15,0.44) | 7.68(11.68,4.58) | 4.76(6.25,3.41) | -2.09(-2.28,-1.90) |
| Fiji | female | 2.46(3.61,1.52) | 4.53(6.76,2.79) | 2.37(2.26,2.48) | 1.29(1.91,0.82) | 1.16(1.71,0.73) | -0.25(-0.34,-0.16) |
| Finland | female | 77.51(94.75,61.90) | 133.03(166.74,99.90) | 2.52(2.01,3.03) | 2.06(2.51,1.64) | 2.66(3.31,1.98) | 1.44(0.93,1.96) |
| France | female | 1738.78(2087.50,1389.93) | 1304.56(1658.60,1001.48) | -1.26(-1.36,-1.15) | 4.26(5.09,3.40) | 2.01(2.50,1.56) | -2.89(-3.03,-2.76) |
| Gabon | female | 11.01(17.76,6.46) | 17.25(29.01,9.80) | 1.30(0.99,1.61) | 3.60(5.73,2.10) | 3.10(5.31,1.74) | -0.65(-0.99,-0.30) |
| Gambia | female | 8.25(13.42,4.56) | 22.37(36.00,12.84) | 3.25(2.89,3.60) | 5.05(8.18,2.80) | 4.56(7.38,2.57) | -0.65(-0.92,-0.38) |
| Georgia | female | 192.21(239.25,147.42) | 140.94(187.58,101.05) | -1.27(-1.51,-1.02) | 2.24(2.67,1.87) | 3.96(5.22,2.87) | -0.92(-4.69,2.99) |
| Germany | female | 3322.23(3987.88,2685.50) | 2863.68(3606.89,2201.71) | -1.00(-1.17,-0.83) | 5.06(5.95,4.12) | 3.24(3.98,2.56) | -2.10(-2.28,-1.91) |
| Ghana | female | 237.44(373.87,142.16) | 376.55(562.61,220.02) | 1.15(0.91,1.40) | 7.34(11.86,4.27) | 4.24(6.46,2.45) | -2.30(-2.53,-2.08) |
| Greece | female | 211.82(273.00,158.36) | 157.24(207.20,114.65) | -1.55(-1.82,-1.27) | 2.57(3.27,1.97) | 1.24(1.56,0.94) | -2.99(-3.26,-2.71) |
| Greenland | female | 0.67(0.98,0.42) | 0.88(1.33,0.56) | 1.14(0.94,1.34) | 3.21(4.71,2.04) | 2.56(3.79,1.68) | -0.75(-0.88,-0.63) |
| Grenada | female | 1.55(2.01,1.16) | 1.45(1.90,1.07) | -0.12(-0.54,0.30) | 3.90(4.99,2.92) | 2.48(3.25,1.83) | -1.62(-2.03,-1.20) |
| Guam | female | 0.65(0.96,0.43) | 1.18(1.74,0.77) | 1.80(1.52,2.09) | 1.93(2.83,1.27) | 1.19(1.77,0.78) | -1.93(-2.37,-1.49) |
| Guatemala | female | 196.87(254.12,144.27) | 528.47(712.57,365.97) | 3.50(3.15,3.85) | 9.69(12.49,7.23) | 8.57(11.68,5.93) | -0.49(-0.79,-0.20) |
| Guinea | female | 100.21(160.95,58.46) | 132.48(222.68,70.93) | 0.60(0.39,0.82) | 6.10(9.78,3.58) | 4.78(8.11,2.57) | -0.93(-1.01,-0.86) |
| Guinea-Bissau | female | 15.52(23.91,8.90) | 28.37(41.25,17.78) | 1.96(1.87,2.04) | 7.23(11.06,4.22) | 6.95(10.05,4.33) | -0.24(-0.31,-0.18) |
| Guyana | female | 15.00(19.65,10.90) | 18.34(25.64,12.56) | 1.09(0.73,1.45) | 7.45(9.81,5.46) | 5.28(7.33,3.66) | -0.84(-1.15,-0.53) |
| Haiti | female | 148.20(220.38,76.78) | 226.93(377.82,104.03) | 1.54(1.42,1.66) | 8.50(12.54,4.58) | 5.84(9.46,2.70) | -1.21(-1.29,-1.12) |
| Honduras | female | 111.88(149.56,78.95) | 422.83(656.71,237.21) | 4.77(4.56,4.99) | 10.02(13.40,7.05) | 13.26(20.48,7.48) | 1.04(0.75,1.33) |
| Hungary | female | 701.50(816.05,581.13) | 419.17(534.38,319.35) | -2.70(-3.02,-2.37) | 9.30(10.85,7.71) | 4.42(5.69,3.34) | -3.64(-4.01,-3.27) |
| Iceland | female | 1.68(2.11,1.28) | 1.70(2.21,1.25) | -0.22(-0.34,-0.10) | 1.23(1.55,0.94) | 0.66(0.86,0.48) | -2.46(-2.61,-2.31) |
| India | female | 6779.14(9726.33,4910.37) | 13951.99(19483.26,9923.73) | 2.38(2.16,2.61) | 3.04(4.28,2.23) | 2.38(3.32,1.71) | -1.08(-1.33,-0.83) |
| Indonesia | female | 3136.80(3982.79,2387.60) | 5252.95(6929.99,3874.77) | 1.86(1.76,1.97) | 6.00(7.69,4.56) | 4.97(6.48,3.70) | -0.47(-0.57,-0.36) |
| Iran (Islamic Republic of) | female | 63.72(90.11,44.34) | 127.81(164.15,99.97) | 3.04(2.70,3.37) | 0.59(0.87,0.39) | 0.38(0.48,0.29) | -1.09(-1.37,-0.81) |
| Iraq | female | 24.61(38.64,14.38) | 50.69(78.91,30.61) | 2.54(2.38,2.69) | 0.63(0.98,0.37) | 0.44(0.68,0.27) | -1.18(-1.34,-1.02) |
| Ireland | female | 29.95(37.37,23.20) | 56.33(70.43,43.58) | 2.06(1.65,2.47) | 1.45(1.80,1.13) | 1.55(1.92,1.21) | 0.02(-0.46,0.51) |
| Israel | female | 47.76(63.28,34.45) | 91.62(122.99,65.95) | 1.59(1.23,1.95) | 1.84(2.42,1.35) | 1.42(1.88,1.04) | -1.49(-1.81,-1.17) |
| Italy | female | 1187.04(1389.26,1000.31) | 816.11(1009.62,656.73) | -1.67(-1.85,-1.49) | 2.44(2.82,2.08) | 1.02(1.25,0.83) | -3.40(-3.62,-3.18) |
| Jamaica | female | 13.05(16.68,9.80) | 18.83(25.85,12.97) | 1.01(0.68,1.34) | 1.41(1.78,1.05) | 1.19(1.63,0.81) | -0.86(-1.20,-0.52) |
| Japan | female | 1138.78(1337.04,964.46) | 1309.16(1674.67,984.96) | 0.74(0.54,0.94) | 1.20(1.41,1.02) | 0.60(0.72,0.48) | -2.15(-2.37,-1.94) |
| Jordan | female | 5.38(8.47,3.15) | 13.79(22.20,8.06) | 2.92(2.69,3.16) | 0.91(1.44,0.54) | 0.51(0.82,0.30) | -2.27(-2.63,-1.91) |
| Kazakhstan | female | 341.25(417.39,266.37) | 1016.26(1281.88,780.89) | 4.47(4.06,4.88) | 4.41(5.38,3.44) | 9.91(12.48,7.70) | 3.52(3.03,4.01) |
| Kenya | female | 382.19(604.71,228.44) | 974.92(1587.81,548.35) | 3.58(3.44,3.72) | 9.38(14.55,5.68) | 8.79(14.20,4.96) | 0.12(-0.02,0.27) |
| Kiribati | female | 0.94(1.48,0.55) | 1.24(2.03,0.65) | 1.02(0.85,1.19) | 4.55(7.39,2.71) | 3.20(5.00,1.84) | -1.24(-1.34,-1.14) |
| Kuwait | female | 0.76(1.15,0.47) | 2.61(4.08,1.60) | 5.18(4.51,5.86) | 0.34(0.53,0.21) | 0.27(0.42,0.16) | 0.15(-0.74,1.05) |
| Kyrgyzstan | female | 140.46(170.72,108.83) | 255.12(320.01,197.35) | 1.75(1.35,2.14) | 7.76(9.43,6.04) | 9.41(11.75,7.30) | 0.48(-0.09,1.05) |
| Lao People's Democratic Republic | female | 46.98(72.82,25.10) | 68.63(102.69,41.62) | 0.98(0.69,1.27) | 4.11(6.29,2.25) | 3.00(4.45,1.80) | -1.37(-1.57,-1.17) |
| Latvia | female | 38.09(49.51,27.82) | 58.61(83.46,40.28) | 1.34(0.71,1.98) | 1.79(2.29,1.32) | 3.30(4.77,2.21) | 2.01(1.33,2.70) |
| Lebanon | female | 7.32(11.68,4.16) | 12.74(22.80,6.63) | 1.89(1.77,2.01) | 0.68(1.09,0.38) | 0.45(0.80,0.23) | -1.49(-1.62,-1.36) |
| Lesotho | female | 15.80(27.99,7.39) | 25.36(42.54,11.52) | 2.21(1.94,2.48) | 2.84(5.05,1.33) | 3.36(5.56,1.57) | 1.29(0.98,1.60) |
| Liberia | female | 38.86(60.66,24.65) | 57.21(85.10,35.95) | 0.93(0.80,1.05) | 7.77(12.12,4.99) | 5.73(8.56,3.65) | -1.39(-1.51,-1.26) |
| Libya | female | 7.11(12.63,3.59) | 14.71(22.61,8.73) | 2.62(2.27,2.98) | 0.85(1.54,0.42) | 0.62(0.97,0.36) | -0.99(-1.24,-0.74) |
| Lithuania | female | 47.16(59.49,35.35) | 120.77(162.33,87.95) | 4.07(3.05,5.10) | 1.83(2.30,1.37) | 4.78(6.42,3.49) | 4.19(3.17,5.23) |
| Luxembourg | female | 13.75(16.93,10.87) | 12.12(15.49,9.17) | -0.55(-0.65,-0.45) | 4.81(5.82,3.79) | 2.43(3.09,1.85) | -2.40(-2.52,-2.27) |
| Madagascar | female | 142.56(213.11,91.31) | 290.66(445.94,177.06) | 2.41(2.28,2.53) | 5.66(8.67,3.63) | 5.19(7.98,3.20) | -0.38(-0.49,-0.27) |
| Malawi | female | 151.45(225.15,96.57) | 190.63(293.79,113.91) | 0.44(0.22,0.66) | 7.62(11.45,4.83) | 4.93(7.64,2.95) | -1.74(-1.89,-1.59) |
| Malaysia | female | 61.74(89.98,39.71) | 217.22(337.05,128.40) | 3.64(3.22,4.07) | 1.32(1.94,0.85) | 1.70(2.57,1.02) | 0.01(-0.50,0.51) |
| Maldives | female | 1.03(1.77,0.48) | 1.53(2.21,1.03) | 1.00(0.73,1.28) | 2.64(4.34,1.33) | 1.18(1.74,0.77) | -3.24(-3.47,-3.01) |
| Mali | female | 153.90(238.86,94.41) | 194.12(315.66,105.65) | 0.41(0.21,0.61) | 7.41(11.56,4.51) | 4.61(7.51,2.51) | -2.00(-2.22,-1.78) |
| Malta | female | 4.68(5.86,3.58) | 4.88(6.32,3.65) | -0.06(-0.19,0.07) | 2.01(2.52,1.54) | 1.19(1.53,0.91) | -2.03(-2.13,-1.92) |
| Marshall Islands | female | 0.24(0.37,0.14) | 0.42(0.79,0.18) | 1.76(1.67,1.85) | 2.79(4.19,1.72) | 2.33(4.26,1.06) | -0.90(-1.02,-0.78) |
| Mauritania | female | 37.79(56.89,23.96) | 39.63(60.44,23.37) | -0.03(-0.18,0.12) | 7.30(10.91,4.65) | 3.88(5.95,2.29) | -2.37(-2.48,-2.26) |
| Mauritius | female | 7.34(10.12,5.21) | 11.11(15.92,7.38) | 0.90(0.63,1.17) | 1.81(2.49,1.27) | 1.18(1.68,0.79) | -2.03(-2.29,-1.76) |
| Mexico | female | 1838.03(2069.67,1614.62) | 3860.77(4925.59,3004.31) | 2.19(2.03,2.35) | 8.10(9.15,7.10) | 6.18(7.84,4.82) | -1.33(-1.49,-1.17) |
| Micronesia (Federated States of) | female | 0.75(1.21,0.44) | 0.83(1.54,0.37) | 0.29(0.02,0.56) | 3.15(5.00,1.89) | 2.22(3.92,1.04) | -1.28(-1.52,-1.03) |
| Monaco | female | 0.78(1.10,0.50) | 1.03(1.43,0.68) | 1.19(1.02,1.36) | 2.26(3.18,1.53) | 2.33(3.20,1.55) | 0.36(0.20,0.52) |
| Mongolia | female | 102.83(135.97,72.30) | 269.38(365.74,187.72) | 3.56(3.39,3.73) | 18.05(23.82,12.73) | 22.38(30.16,15.46) | 1.09(0.69,1.50) |
| Montenegro | female | 3.29(4.55,2.42) | 4.94(6.38,3.67) | 1.15(0.87,1.43) | 0.95(1.31,0.69) | 0.99(1.29,0.74) | -0.10(-0.32,0.13) |
| Morocco | female | 54.13(88.22,30.52) | 99.09(157.53,59.03) | 2.18(2.10,2.27) | 0.83(1.39,0.45) | 0.67(1.04,0.40) | -0.62(-0.71,-0.52) |
| Mozambique | female | 101.83(163.85,60.16) | 157.34(278.61,50.33) | 1.63(1.45,1.81) | 3.45(5.49,2.04) | 2.76(4.88,0.87) | -0.65(-0.78,-0.52) |
| Myanmar | female | 350.12(552.90,202.33) | 863.38(1279.40,566.14) | 3.11(2.85,3.37) | 2.55(4.04,1.51) | 3.03(4.43,2.01) | 0.56(0.32,0.80) |
| Namibia | female | 11.81(19.71,6.21) | 20.61(36.08,9.28) | 1.41(1.15,1.67) | 3.02(5.02,1.59) | 2.53(4.42,1.15) | -0.96(-1.18,-0.75) |
| Nauru | female | 0.05(0.09,0.02) | 0.05(0.10,0.02) | 0.29(0.21,0.36) | 2.61(4.30,1.39) | 2.20(3.79,1.09) | -0.52(-0.63,-0.42) |
| Nepal | female | 412.13(587.17,269.10) | 854.83(1259.18,557.85) | 2.93(2.60,3.27) | 8.38(12.16,5.44) | 7.39(10.93,4.91) | -0.18(-0.46,0.10) |
| Netherlands | female | 221.26(276.95,171.93) | 259.57(339.94,192.35) | -0.13(-0.49,0.23) | 1.99(2.44,1.57) | 1.41(1.80,1.08) | -1.85(-2.22,-1.47) |
| New Zealand | female | 11.07(13.28,9.18) | 14.34(17.98,11.30) | 1.04(0.91,1.16) | 0.54(0.65,0.45) | 0.35(0.44,0.28) | -1.40(-1.51,-1.30) |
| Nicaragua | female | 31.72(40.41,23.84) | 135.71(180.02,99.02) | 5.22(4.92,5.52) | 3.75(4.80,2.81) | 5.71(7.51,4.24) | 1.61(1.25,1.97) |
| Niger | female | 77.86(119.59,48.23) | 194.80(306.01,105.30) | 2.79(2.63,2.96) | 5.80(8.97,3.54) | 4.84(7.80,2.64) | -1.06(-1.25,-0.88) |
| Nigeria | female | 1234.45(1874.43,793.78) | 2271.84(3613.23,1374.29) | 2.42(2.17,2.68) | 6.03(9.19,3.89) | 5.14(7.89,3.16) | -0.22(-0.38,-0.06) |
| Niue | female | 0.02(0.04,0.01) | 0.02(0.03,0.01) | -1.26(-1.41,-1.12) | 1.90(3.01,1.12) | 1.52(2.40,0.86) | -0.94(-1.03,-0.85) |
| North Macedonia | female | 18.44(25.13,14.12) | 34.20(46.56,24.00) | 2.04(1.89,2.19) | 1.89(2.55,1.46) | 2.07(2.81,1.48) | 0.25(0.14,0.36) |
| Northern Mariana Islands | female | 0.26(0.39,0.17) | 0.52(0.76,0.32) | 2.31(2.09,2.53) | 3.41(5.02,2.17) | 2.04(2.91,1.30) | -1.98(-2.18,-1.78) |
| Norway | female | 46.17(54.14,39.00) | 47.16(57.93,38.73) | 0.04(-0.03,0.12) | 1.39(1.60,1.19) | 0.97(1.17,0.81) | -1.32(-1.43,-1.22) |
| Oman | female | 3.02(5.09,1.66) | 4.89(7.90,2.76) | 2.13(1.93,2.33) | 1.05(1.76,0.57) | 0.72(1.15,0.42) | -0.79(-1.03,-0.55) |
| Pakistan | female | 1433.09(2562.73,771.25) | 2431.11(3824.97,1503.72) | 1.56(1.47,1.64) | 5.75(10.61,3.01) | 4.76(7.72,2.92) | -0.92(-1.05,-0.79) |
| Palau | female | 0.07(0.12,0.03) | 0.13(0.24,0.06) | 2.06(2.01,2.12) | 1.42(2.47,0.67) | 1.22(2.15,0.60) | -0.39(-0.47,-0.32) |
| Palestine | female | 4.03(6.91,2.14) | 8.70(13.80,5.28) | 3.12(2.82,3.41) | 0.89(1.51,0.48) | 0.79(1.24,0.47) | -0.07(-0.33,0.19) |
| Panama | female | 21.16(26.65,15.99) | 56.64(78.16,39.28) | 3.84(3.67,4.00) | 2.86(3.63,2.15) | 2.64(3.65,1.83) | 0.09(-0.06,0.24) |
| Papua New Guinea | female | 4.34(7.03,2.44) | 11.18(18.42,6.23) | 3.56(3.33,3.78) | 0.41(0.67,0.23) | 0.40(0.68,0.22) | 0.14(0.03,0.24) |
| Paraguay | female | 20.22(26.96,14.42) | 43.11(62.18,27.98) | 2.66(2.40,2.92) | 1.72(2.31,1.21) | 1.47(2.13,0.96) | -0.51(-0.76,-0.26) |
| Peru | female | 396.57(518.21,289.11) | 793.81(1151.20,497.22) | 2.05(1.80,2.31) | 6.36(8.32,4.64) | 4.78(6.98,2.98) | -1.33(-1.57,-1.10) |
| Philippines | female | 334.06(497.27,235.38) | 714.20(965.10,509.69) | 2.55(2.32,2.77) | 2.20(3.33,1.54) | 1.75(2.34,1.25) | -0.87(-1.12,-0.63) |
| Poland | female | 894.26(993.81,798.06) | 1095.27(1394.05,843.47) | 0.96(0.74,1.17) | 3.50(3.88,3.14) | 3.15(4.04,2.42) | -0.15(-0.37,0.07) |
| Portugal | female | 419.96(506.49,334.12) | 215.83(273.68,166.16) | -2.77(-2.98,-2.56) | 5.87(7.03,4.74) | 1.88(2.32,1.45) | -4.42(-4.63,-4.22) |
| Puerto Rico | female | 77.88(97.41,58.66) | 98.52(138.84,65.36) | 0.00(-0.31,0.31) | 4.03(5.03,3.05) | 2.51(3.56,1.69) | -2.41(-2.72,-2.10) |
| Qatar | female | 0.40(0.64,0.24) | 2.05(3.53,1.06) | 6.32(6.04,6.61) | 1.35(2.17,0.78) | 1.65(2.77,0.89) | 1.58(1.11,2.05) |
| Republic of Korea | female | 1028.87(1309.93,760.47) | 1055.68(1427.76,776.56) | -1.03(-1.45,-0.61) | 6.09(7.88,4.49) | 2.16(2.86,1.62) | -4.76(-5.16,-4.35) |
| Republic of Moldova | female | 710.33(853.76,547.65) | 541.54(693.17,408.53) | -1.02(-1.40,-0.64) | 26.44(31.60,20.57) | 16.47(20.84,12.44) | -1.64(-2.07,-1.22) |
| Romania | female | 1227.22(1450.86,1003.21) | 1672.04(2139.98,1295.42) | 0.70(0.34,1.07) | 7.95(9.37,6.55) | 8.64(11.14,6.63) | -0.10(-0.46,0.26) |
| Russian Federation | female | 2347.29(2731.90,1972.02) | 6825.31(8721.48,5356.31) | 3.88(3.06,4.72) | 2.07(2.41,1.74) | 5.50(7.06,4.30) | 3.58(2.65,4.53) |
| Rwanda | female | 163.95(250.23,97.43) | 165.75(247.68,98.57) | -0.30(-0.80,0.20) | 10.33(15.68,6.29) | 4.92(7.30,2.97) | -3.37(-3.74,-3.00) |
| Saint Kitts and Nevis | female | 1.08(1.44,0.77) | 0.82(1.17,0.53) | -0.79(-1.34,-0.23) | 5.38(6.96,3.95) | 2.37(3.41,1.55) | -2.66(-2.98,-2.33) |
| Saint Lucia | female | 2.91(3.65,2.23) | 3.62(4.77,2.67) | 0.26(-0.35,0.86) | 6.09(7.59,4.67) | 3.18(4.21,2.35) | -2.88(-3.44,-2.32) |
| Saint Vincent and the Grenadines | female | 0.98(1.28,0.71) | 1.22(1.61,0.86) | 0.61(0.03,1.19) | 2.51(3.27,1.83) | 1.85(2.44,1.31) | -1.29(-1.79,-0.79) |
| Samoa | female | 0.88(1.38,0.54) | 1.26(2.02,0.70) | 1.25(1.16,1.34) | 1.99(3.08,1.23) | 1.70(2.71,0.94) | -0.53(-0.65,-0.41) |
| San Marino | female | 0.68(0.95,0.49) | 1.23(1.94,0.73) | 2.40(2.18,2.63) | 3.92(5.39,2.81) | 3.66(5.78,2.19) | 0.15(-0.06,0.36) |
| Sao Tome and Principe | female | 2.12(3.20,1.30) | 2.60(4.35,1.32) | 0.20(-0.09,0.48) | 6.40(9.69,3.98) | 4.89(8.24,2.46) | -1.33(-1.67,-0.98) |
| Saudi Arabia | female | 43.24(80.65,21.05) | 57.87(91.01,34.88) | 0.48(0.24,0.72) | 1.99(3.75,0.95) | 1.02(1.62,0.60) | -2.53(-2.68,-2.38) |
| Senegal | female | 69.76(107.06,42.73) | 136.89(226.23,73.01) | 2.39(2.19,2.58) | 4.41(6.74,2.73) | 3.55(5.89,1.88) | -0.76(-0.96,-0.56) |
| Serbia | female | 145.04(192.43,108.49) | 154.04(209.21,112.20) | -0.32(-0.56,-0.08) | 2.39(3.13,1.81) | 1.86(2.53,1.37) | -1.26(-1.45,-1.08) |
| Seychelles | female | 0.73(1.02,0.49) | 1.57(2.23,1.05) | 2.62(2.32,2.92) | 2.32(3.24,1.57) | 2.76(3.90,1.83) | 0.51(0.18,0.85) |
| Sierra Leone | female | 52.72(83.65,32.44) | 75.28(127.63,35.92) | 0.88(0.67,1.09) | 5.78(9.18,3.57) | 4.15(7.10,1.94) | -1.51(-1.75,-1.28) |
| Singapore | female | 6.97(10.01,4.76) | 12.75(18.04,8.36) | 2.18(1.91,2.44) | 0.59(0.85,0.40) | 0.32(0.45,0.21) | -2.09(-2.26,-1.93) |
| Slovakia | female | 171.09(211.21,137.33) | 215.90(290.38,155.00) | 1.42(1.08,1.77) | 5.32(6.53,4.28) | 4.59(6.19,3.29) | 0.04(-0.29,0.38) |
| Slovenia | female | 107.53(147.52,75.45) | 63.68(88.34,44.82) | -2.11(-2.32,-1.89) | 7.76(10.64,5.44) | 3.08(4.28,2.18) | -3.57(-3.82,-3.32) |
| Solomon Islands | female | 2.40(3.93,1.40) | 5.00(7.50,3.20) | 2.57(2.44,2.70) | 3.21(5.20,1.85) | 2.71(3.97,1.72) | -0.53(-0.63,-0.43) |
| Somalia | female | 87.93(150.54,43.11) | 215.28(380.05,113.10) | 3.20(3.09,3.31) | 6.58(11.31,3.30) | 5.83(10.11,3.08) | -0.34(-0.44,-0.24) |
| South Africa | female | 247.06(348.73,186.35) | 362.21(459.30,282.35) | 1.24(0.78,1.70) | 1.98(2.87,1.47) | 1.40(1.77,1.09) | -1.20(-1.58,-0.81) |
| South Sudan | female | 52.37(81.42,31.73) | 80.32(133.60,46.35) | 1.67(1.43,1.91) | 5.08(7.94,3.04) | 4.63(7.66,2.69) | -0.12(-0.20,-0.04) |
| Spain | female | 1406.70(1741.14,1114.61) | 1004.87(1310.34,766.66) | -1.49(-1.63,-1.35) | 4.72(5.71,3.79) | 1.85(2.31,1.45) | -3.53(-3.69,-3.38) |
| Sri Lanka | female | 98.62(141.09,66.62) | 191.10(297.60,117.81) | 2.19(1.93,2.46) | 1.87(2.64,1.26) | 1.39(2.12,0.88) | -1.05(-1.30,-0.80) |
| Sudan | female | 46.23(82.92,22.85) | 70.65(122.53,36.96) | 1.24(1.13,1.36) | 1.09(2.02,0.54) | 0.88(1.58,0.44) | -0.77(-0.81,-0.72) |
| Suriname | female | 7.61(9.73,5.67) | 13.20(17.70,9.16) | 1.66(1.38,1.94) | 5.53(7.08,4.11) | 4.08(5.48,2.85) | -1.45(-1.69,-1.20) |
| Sweden | female | 109.69(125.76,94.58) | 129.05(155.12,106.45) | 0.76(0.63,0.88) | 1.53(1.73,1.33) | 1.30(1.55,1.08) | -0.34(-0.49,-0.20) |
| Switzerland | female | 113.83(140.81,89.60) | 142.66(186.76,108.65) | 0.47(0.22,0.71) | 2.20(2.66,1.74) | 1.65(2.09,1.27) | -1.35(-1.61,-1.09) |
| Syrian Arab Republic | female | 23.45(37.06,13.87) | 43.75(72.21,25.20) | 2.38(2.04,2.72) | 1.03(1.64,0.60) | 0.89(1.41,0.53) | -0.48(-0.72,-0.24) |
| Taiwan (Province of China) | female | 192.45(273.97,132.81) | 327.78(497.16,210.02) | 1.19(0.73,1.64) | 2.78(3.90,1.92) | 1.53(2.31,0.97) | -2.75(-3.21,-2.30) |
| Tajikistan | female | 109.85(138.01,84.41) | 289.10(385.68,207.11) | 3.07(2.88,3.27) | 6.91(8.67,5.25) | 10.76(14.32,7.74) | 1.45(1.21,1.70) |
| Thailand | female | 801.09(1126.02,545.26) | 1463.57(2182.78,915.75) | 2.04(1.89,2.19) | 3.99(5.58,2.74) | 2.62(3.91,1.66) | -1.50(-1.62,-1.38) |
| Timor-Leste | female | 4.73(7.42,2.77) | 11.32(18.42,6.43) | 3.00(2.81,3.18) | 3.12(4.85,1.82) | 2.80(4.47,1.63) | -0.68(-0.87,-0.49) |
| Togo | female | 39.31(57.30,26.39) | 84.32(133.51,47.26) | 2.46(2.35,2.58) | 5.98(8.69,3.99) | 4.04(6.54,2.23) | -1.58(-1.69,-1.48) |
| Tokelau | female | 0.02(0.03,0.01) | 0.01(0.02,0.01) | -1.81(-2.13,-1.48) | 2.54(4.23,1.35) | 1.72(2.88,0.96) | -1.34(-1.46,-1.22) |
| Tonga | female | 0.73(1.08,0.47) | 0.86(1.31,0.53) | 0.49(0.37,0.61) | 2.52(3.69,1.63) | 2.01(3.06,1.23) | -0.83(-0.96,-0.70) |
| Trinidad and Tobago | female | 11.86(14.95,8.99) | 17.67(25.05,11.69) | 1.33(1.15,1.51) | 2.68(3.37,2.02) | 1.86(2.63,1.23) | -1.35(-1.53,-1.16) |
| Tunisia | female | 15.12(24.82,8.42) | 30.40(50.75,16.66) | 2.53(2.49,2.57) | 0.67(1.08,0.37) | 0.48(0.80,0.26) | -0.92(-0.98,-0.85) |
| Turkey | female | 103.13(162.62,58.98) | 188.99(292.88,114.11) | 2.17(1.90,2.44) | 0.57(0.88,0.33) | 0.41(0.63,0.25) | -1.06(-1.36,-0.75) |
| Turkmenistan | female | 109.77(135.04,86.01) | 275.22(368.11,200.10) | 3.11(2.85,3.37) | 9.68(11.84,7.59) | 11.87(15.69,8.68) | 0.55(0.31,0.79) |
| Tuvalu | female | 0.11(0.18,0.05) | 0.10(0.18,0.05) | -0.17(-0.37,0.03) | 2.69(4.51,1.44) | 1.88(3.25,0.98) | -1.18(-1.34,-1.03) |
| Uganda | female | 156.97(246.13,84.25) | 335.77(518.16,172.18) | 2.25(1.97,2.53) | 4.92(7.65,2.65) | 4.46(6.88,2.20) | -0.64(-0.88,-0.39) |
| Ukraine | female | 983.33(1162.66,825.26) | 2123.29(2836.58,1558.62) | 2.88(2.22,3.55) | 2.27(2.69,1.93) | 5.74(7.64,4.15) | 3.46(2.66,4.26) |
| United Arab Emirates | female | 1.01(1.78,0.55) | 5.01(8.80,2.45) | 5.44(5.25,5.64) | 0.83(1.51,0.43) | 0.56(1.04,0.26) | -1.15(-2.07,-0.22) |
| United Kingdom | female | 893.64(1018.52,765.27) | 1653.11(1892.42,1435.22) | 2.19(1.84,2.54) | 2.09(2.36,1.82) | 3.09(3.50,2.71) | 1.35(0.95,1.76) |
| United Republic of Tanzania | female | 307.07(453.46,190.68) | 532.03(866.61,264.22) | 1.94(1.79,2.10) | 5.56(8.27,3.50) | 4.25(7.05,2.05) | -0.81(-0.98,-0.63) |
| United States of America | female | 3048.23(3582.38,2542.85) | 5237.72(6250.97,4342.41) | 2.34(2.16,2.51) | 1.83(2.15,1.53) | 1.92(2.27,1.60) | 0.59(0.43,0.74) |
| United States Virgin Islands | female | 1.99(2.67,1.42) | 2.88(3.99,1.93) | 1.13(1.04,1.22) | 4.36(5.88,3.08) | 2.84(3.85,1.95) | -1.64(-1.75,-1.53) |
| Uruguay | female | 33.50(45.87,23.45) | 28.39(39.97,19.26) | -0.75(-1.05,-0.45) | 1.58(2.14,1.12) | 0.92(1.28,0.63) | -2.02(-2.29,-1.75) |
| Uzbekistan | female | 544.28(675.15,421.98) | 1904.06(2448.88,1443.59) | 3.37(2.83,3.90) | 8.21(10.16,6.37) | 15.21(19.27,11.53) | 1.40(0.76,2.04) |
| Vanuatu | female | 0.77(1.25,0.42) | 1.76(2.90,0.95) | 2.73(2.49,2.97) | 2.55(4.23,1.37) | 2.08(3.43,1.15) | -1.03(-1.23,-0.82) |
| Venezuela (Bolivarian Republic of) | female | 169.70(208.66,131.34) | 324.36(459.86,212.11) | 1.88(1.50,2.26) | 3.29(4.08,2.53) | 2.08(2.95,1.36) | -2.01(-2.38,-1.63) |
| Viet Nam | female | 708.81(1099.20,414.25) | 987.80(1478.22,640.02) | 0.86(0.45,1.28) | 3.12(4.85,1.82) | 1.95(2.91,1.26) | -1.94(-2.33,-1.55) |
| Yemen | female | 28.51(51.80,14.08) | 56.91(92.58,30.50) | 2.27(2.19,2.35) | 1.19(2.19,0.57) | 0.89(1.50,0.48) | -1.15(-1.23,-1.07) |
| Zambia | female | 116.46(173.11,73.59) | 232.28(358.28,147.67) | 2.06(1.81,2.32) | 8.64(12.68,5.46) | 6.79(10.39,4.31) | -1.16(-1.36,-0.97) |
| Zimbabwe | female | 65.07(98.40,40.85) | 125.40(233.92,43.04) | 3.24(2.73,3.74) | 3.20(4.83,1.98) | 3.15(5.76,1.11) | 1.08(0.52,1.64) |

**Supplementary table 5. The DALYs and age-standardized DALY rate of cirrhosis and other chronic liver diseases due to alcohol use in 1990 and 2019, and its temporal trends from 1990 to 2019.**

| **Nation** | **Sex** | **DALY Cases No. (95% UI)** | | **1990-2019 EAPC of numbers** | **Age-standardized DALY rate per 100,000 No.(95% UI)** | | **1990-2019 EAPC of age-standardized DALY rate** |
| --- | --- | --- | --- | --- | --- | --- | --- |
|  |  | **1990** | **2019** |  | **1990** | **2019** |  |
| Afghanistan | both | 4388.41(7649.48,2124.58) | 6923.07(12371.02,3137.48) | 1.06(0.81,1.31) | 57.39(97.35,29.04) | 46.00(78.72,23.86) | -1.05(-1.20,-0.90) |
| Albania | both | 2964.29(3604.34,2384.06) | 3506.33(4850.29,2428.26) | 0.94(0.63,1.25) | 131.99(159.83,105.91) | 88.18(121.53,61.49) | -1.02(-1.38,-0.66) |
| Algeria | both | 3823.23(5896.54,2281.89) | 7498.85(12239.20,4345.39) | 2.17(1.94,2.41) | 29.63(45.80,18.08) | 21.11(33.35,12.25) | -1.34(-1.52,-1.16) |
| American Samoa | both | 27.77(39.24,18.74) | 36.43(53.44,23.41) | 0.74(0.60,0.89) | 94.33(133.83,63.81) | 68.53(98.64,44.41) | -1.19(-1.37,-1.01) |
| Andorra | both | 88.80(134.39,54.20) | 164.86(222.13,116.58) | 1.82(1.57,2.08) | 149.06(225.66,91.10) | 121.29(163.83,85.89) | -0.78(-0.81,-0.75) |
| Angola | both | 15073.79(23440.74,8982.50) | 34718.15(50954.88,22996.90) | 2.94(2.76,3.12) | 312.65(481.66,189.30) | 248.06(358.29,161.05) | -0.83(-0.92,-0.74) |
| Antigua and Barbuda | both | 90.31(109.51,68.82) | 148.06(188.73,112.55) | 1.78(1.49,2.07) | 185.13(225.03,139.75) | 136.47(171.97,104.51) | -1.18(-1.49,-0.86) |
| Argentina | both | 52306.84(66366.08,37540.22) | 70011.96(92209.22,50292.00) | 1.23(0.88,1.59) | 161.02(204.67,116.11) | 136.92(179.85,98.39) | -0.35(-0.66,-0.05) |
| Armenia | both | 4329.69(5230.13,3399.62) | 10894.49(13689.46,8326.57) | 3.56(3.07,4.04) | 146.78(176.59,116.70) | 273.15(340.76,210.39) | 2.58(2.06,3.10) |
| Australia | both | 7758.50(10511.36,5472.05) | 10830.03(14971.32,7426.57) | 1.81(1.53,2.10) | 41.64(56.71,29.06) | 31.09(42.63,21.20) | -0.42(-0.72,-0.13) |
| Austria | both | 32210.41(37078.49,26582.42) | 23511.56(27602.29,19173.63) | -1.18(-1.26,-1.09) | 326.58(376.30,269.40) | 163.71(191.47,135.15) | -2.55(-2.63,-2.46) |
| Azerbaijan | both | 14992.60(18653.67,11756.32) | 34579.10(45569.30,25466.19) | 2.11(1.82,2.40) | 274.64(338.76,217.00) | 338.79(440.93,252.72) | -0.02(-0.40,0.36) |
| Bahamas | both | 503.45(616.83,389.48) | 820.11(1096.46,598.09) | 1.55(1.31,1.79) | 282.87(346.06,218.69) | 186.29(246.52,136.65) | -1.61(-1.91,-1.32) |
| Bahrain | both | 100.59(154.07,61.56) | 346.38(574.03,197.72) | 4.38(4.13,4.64) | 48.91(74.39,29.82) | 27.35(43.16,16.36) | -2.38(-2.66,-2.09) |
| Bangladesh | both | 156161.55(220954.21,101168.16) | 186741.52(268922.42,123792.28) | 0.43(0.24,0.62) | 277.63(394.49,181.50) | 133.00(190.56,88.67) | -2.79(-2.99,-2.59) |
| Barbados | both | 375.64(449.68,300.54) | 546.52(712.38,402.61) | 1.07(0.81,1.34) | 152.41(183.46,120.63) | 119.95(156.77,89.35) | -1.19(-1.43,-0.94) |
| Belarus | both | 10028.52(12342.08,7759.14) | 34148.44(46766.24,24519.30) | 5.07(3.84,6.31) | 78.14(96.04,60.72) | 245.62(335.89,178.43) | 4.80(3.48,6.14) |
| Belgium | both | 22696.24(25674.19,19385.51) | 25673.34(30035.83,21163.77) | 0.30(0.17,0.42) | 171.45(193.55,147.44) | 142.02(165.62,117.78) | -0.86(-1.02,-0.71) |
| Belize | both | 189.40(232.34,147.48) | 853.00(1065.44,662.29) | 5.13(4.82,5.43) | 195.17(240.23,150.78) | 261.19(326.05,204.20) | 0.70(0.33,1.08) |
| Benin | both | 6660.47(9697.51,4458.83) | 14138.68(20846.89,9169.56) | 2.46(2.36,2.57) | 306.11(443.79,201.76) | 240.07(351.45,156.53) | -0.99(-1.09,-0.89) |
| Bermuda | both | 115.06(139.24,91.24) | 84.50(110.33,63.95) | -1.13(-1.50,-0.76) | 177.34(215.22,140.58) | 74.00(96.70,56.25) | -3.15(-3.56,-2.74) |
| Bhutan | both | 750.41(1295.19,433.52) | 1405.87(2857.20,809.04) | 2.43(2.27,2.60) | 227.36(390.13,133.50) | 217.93(445.81,126.12) | -0.01(-0.15,0.13) |
| Bolivia (Plurinational State of) | both | 15863.48(22840.00,9756.00) | 36526.05(51449.63,24567.38) | 2.93(2.88,2.98) | 438.13(634.88,272.60) | 386.74(540.97,261.87) | -0.49(-0.56,-0.42) |
| Bosnia and Herzegovina | both | 8981.65(10876.62,7045.55) | 7354.17(9971.98,5330.37) | -1.22(-1.49,-0.96) | 192.47(231.49,153.97) | 132.10(179.77,96.57) | -1.81(-2.13,-1.48) |
| Botswana | both | 1573.81(2733.34,851.77) | 3515.30(5341.62,2132.37) | 2.14(1.69,2.61) | 235.25(407.05,127.74) | 200.12(297.53,122.95) | -1.06(-1.56,-0.56) |
| Brazil | both | 334956.05(360681.88,310811.90) | 549305.07(599603.64,504231.51) | 1.85(1.74,1.96) | 305.68(329.09,283.65) | 220.97(241.26,203.09) | -1.01(-1.11,-0.92) |
| Brunei Darussalam | both | 135.17(193.14,89.17) | 284.88(400.36,191.67) | 2.04(1.78,2.30) | 95.50(135.70,62.92) | 67.16(92.06,46.36) | -1.76(-2.02,-1.50) |
| Bulgaria | both | 27576.50(32656.66,22315.25) | 38728.57(50766.03,29111.76) | 1.08(0.88,1.28) | 229.53(271.32,188.20) | 347.29(460.78,258.67) | 1.28(1.07,1.50) |
| Burkina Faso | both | 12922.61(18223.75,8711.28) | 20308.02(33912.31,10450.93) | 0.93(0.52,1.33) | 273.77(381.04,186.04) | 192.64(316.91,99.30) | -1.88(-2.27,-1.49) |
| Burundi | both | 9632.75(15339.37,5308.06) | 13994.82(25989.87,7623.89) | 0.84(0.54,1.13) | 374.47(590.67,208.76) | 252.28(463.45,138.81) | -1.86(-2.06,-1.66) |
| Cabo Verde | both | 339.86(517.32,222.63) | 919.88(1263.60,638.51) | 2.80(2.46,3.14) | 163.62(251.29,107.33) | 194.85(264.15,134.88) | -0.16(-0.47,0.16) |
| Cambodia | both | 23599.35(33498.19,15802.59) | 55462.18(79596.37,36683.60) | 2.95(2.76,3.15) | 422.16(595.82,280.62) | 401.14(576.34,265.55) | -0.28(-0.46,-0.10) |
| Cameroon | both | 15042.42(21532.17,10083.59) | 31665.11(48558.69,18695.96) | 2.50(2.38,2.62) | 290.11(416.24,195.19) | 213.39(325.92,125.01) | -1.17(-1.32,-1.02) |
| Canada | both | 25447.20(32967.44,18491.64) | 41692.50(54400.70,29686.79) | 1.92(1.81,2.02) | 81.49(106.33,58.78) | 70.11(90.79,50.58) | -0.42(-0.54,-0.30) |
| Central African Republic | both | 4383.67(7180.80,2362.65) | 7036.47(12464.54,3650.60) | 1.39(1.30,1.49) | 309.05(496.16,172.16) | 254.23(431.90,143.46) | -0.95(-1.03,-0.87) |
| Chad | both | 8125.67(12615.28,4864.66) | 17927.79(26251.93,11689.52) | 2.75(2.70,2.80) | 270.05(416.10,159.48) | 268.24(388.64,174.61) | -0.03(-0.17,0.10) |
| Chile | both | 36342.71(46854.59,26530.35) | 44386.67(57536.58,31770.80) | 1.02(0.77,1.26) | 335.20(434.80,243.03) | 187.55(241.22,135.86) | -1.75(-1.99,-1.51) |
| China | both | 892765.43(1116862.82,699382.84) | 877551.34(1114226.90,671826.49) | -0.18(-0.28,-0.07) | 91.33(114.07,72.03) | 42.23(53.27,32.67) | -2.81(-2.92,-2.70) |
| Colombia | both | 21629.16(25853.97,17372.33) | 36136.21(50415.25,25264.91) | 1.34(1.13,1.56) | 109.31(130.84,88.42) | 68.47(95.12,47.97) | -2.08(-2.27,-1.89) |
| Comoros | both | 483.35(947.14,199.42) | 936.95(1493.01,554.23) | 2.09(1.82,2.36) | 204.41(396.44,87.88) | 177.94(279.49,105.68) | -0.67(-0.89,-0.44) |
| Congo | both | 3696.83(5645.51,2238.68) | 6824.50(10072.28,4127.04) | 1.98(1.77,2.19) | 298.46(449.37,184.61) | 210.14(311.46,128.58) | -1.38(-1.51,-1.26) |
| Cook Islands | both | 11.42(16.63,7.25) | 13.78(19.89,8.73) | 0.83(0.70,0.96) | 80.19(115.58,52.28) | 57.70(83.34,36.74) | -0.96(-1.12,-0.80) |
| Costa Rica | both | 3612.29(4292.15,2894.88) | 10328.99(14118.02,7295.49) | 3.08(2.79,3.36) | 186.40(221.46,148.35) | 195.82(267.25,138.86) | -0.43(-0.69,-0.16) |
| Côte d'Ivoire | both | 15781.72(23839.54,10033.07) | 31196.94(47638.63,19022.06) | 2.17(1.97,2.38) | 306.77(463.46,197.03) | 226.62(344.46,141.44) | -1.11(-1.27,-0.94) |
| Croatia | both | 24121.89(28553.26,19473.21) | 14739.49(19782.77,10862.82) | -1.80(-1.97,-1.63) | 373.98(441.89,303.68) | 203.92(273.45,149.96) | -2.21(-2.41,-2.02) |
| Cuba | both | 11106.50(13407.09,8812.35) | 25528.22(33759.51,18594.13) | 2.95(2.75,3.15) | 107.72(129.80,85.50) | 143.00(189.26,104.82) | 0.94(0.71,1.17) |
| Cyprus | both | 1071.52(1453.89,735.12) | 1484.90(1816.62,1174.60) | 0.86(0.75,0.97) | 133.01(181.07,90.39) | 80.45(98.57,64.40) | -2.07(-2.21,-1.94) |
| Czechia | both | 29889.61(34577.82,24924.47) | 32922.58(42722.59,25387.94) | 0.73(0.58,0.88) | 233.32(270.82,195.06) | 196.53(255.57,151.70) | -0.28(-0.42,-0.15) |
| Democratic People's Republic of Korea | both | 21870.53(33702.63,12138.32) | 34080.40(51875.55,18660.24) | 1.46(1.28,1.64) | 113.28(170.44,65.53) | 101.08(153.17,56.42) | -0.42(-0.60,-0.24) |
| Democratic Republic of the Congo | both | 46423.25(67970.46,29787.16) | 88683.94(135310.74,53436.05) | 2.04(1.84,2.24) | 246.95(356.28,158.11) | 198.90(306.05,118.60) | -0.91(-1.03,-0.80) |
| Denmark | both | 12179.92(14126.08,9999.98) | 12015.59(14489.21,9339.59) | -0.65(-1.15,-0.14) | 189.95(220.49,155.38) | 133.90(161.00,104.32) | -1.88(-2.36,-1.39) |
| Djibouti | both | 418.64(789.23,247.30) | 1547.03(2741.42,868.80) | 4.44(4.33,4.55) | 232.05(422.59,141.59) | 208.24(367.50,117.27) | -0.58(-0.68,-0.48) |
| Dominica | both | 112.77(141.26,86.64) | 123.56(162.68,90.79) | 0.09(-0.11,0.29) | 181.45(227.95,139.56) | 143.31(189.44,105.33) | -1.08(-1.24,-0.92) |
| Dominican Republic | both | 15211.07(18674.14,11928.25) | 31636.01(45282.87,19957.12) | 2.16(1.74,2.58) | 362.63(444.21,282.04) | 321.95(458.46,205.73) | -0.78(-1.24,-0.31) |
| Ecuador | both | 15901.65(19737.94,12342.48) | 40414.09(55227.72,28799.89) | 3.40(3.16,3.64) | 263.13(327.38,203.87) | 257.33(351.76,184.76) | 0.09(-0.12,0.31) |
| Egypt | both | 42379.43(64733.00,27428.85) | 87922.95(147687.85,46380.70) | 2.91(2.71,3.11) | 144.52(218.15,91.75) | 136.54(226.16,73.92) | 0.19(-0.02,0.39) |
| El Salvador | both | 10621.04(12901.08,8443.30) | 15806.48(21809.76,10914.70) | 1.09(0.84,1.35) | 320.56(391.44,253.79) | 270.44(374.64,186.28) | -0.85(-1.10,-0.59) |
| Equatorial Guinea | both | 511.48(846.88,276.53) | 681.74(1160.23,378.65) | 0.71(0.31,1.10) | 222.34(369.39,121.25) | 114.60(187.99,65.35) | -2.61(-2.86,-2.36) |
| Eritrea | both | 3875.04(6148.59,2118.66) | 10927.72(16926.11,6687.90) | 3.62(3.46,3.78) | 308.78(488.79,177.80) | 320.13(490.75,199.84) | 0.03(-0.11,0.16) |
| Estonia | both | 1842.16(2287.24,1441.34) | 4312.80(5794.54,3128.30) | 2.70(1.84,3.56) | 94.69(117.85,74.09) | 231.64(309.80,168.11) | 2.78(1.86,3.71) |
| Eswatini | both | 770.24(1186.61,472.81) | 1548.89(2330.92,951.84) | 2.50(2.06,2.95) | 218.81(341.68,137.48) | 219.98(326.60,138.00) | 0.29(-0.06,0.64) |
| Ethiopia | both | 76998.04(116610.63,42526.88) | 93618.55(121316.84,71458.74) | 0.27(0.07,0.48) | 333.47(501.33,186.44) | 202.23(259.42,157.15) | -1.96(-2.08,-1.85) |
| Fiji | both | 361.05(515.40,238.12) | 555.52(844.37,347.84) | 1.63(1.52,1.74) | 74.35(106.91,49.93) | 63.34(94.55,40.65) | -0.54(-0.62,-0.46) |
| Finland | both | 9536.11(11167.04,7737.24) | 15977.40(19191.90,12438.80) | 2.60(1.97,3.23) | 150.84(177.86,122.14) | 191.17(228.95,149.35) | 1.53(0.99,2.08) |
| France | both | 180504.92(209236.76,149042.80) | 126600.92(150512.64,101526.33) | -1.33(-1.45,-1.21) | 255.45(296.42,209.22) | 124.37(148.31,99.53) | -2.66(-2.79,-2.52) |
| Gabon | both | 1810.89(2747.01,1093.06) | 2610.28(3883.14,1655.88) | 1.10(0.98,1.23) | 297.34(450.29,180.22) | 213.68(319.52,135.42) | -1.26(-1.33,-1.19) |
| Gambia | both | 1098.66(1755.51,647.12) | 2629.04(3913.28,1634.69) | 2.75(2.40,3.11) | 264.80(418.73,157.22) | 238.36(353.47,147.11) | -0.56(-0.84,-0.28) |
| Georgia | both | 19317.69(23492.95,15181.94) | 20263.25(25611.10,15357.02) | 0.52(0.25,0.78) | 306.29(370.78,241.62) | 405.52(509.74,303.88) | -0.37(-4.74,4.20) |
| Germany | both | 316986.29(362808.16,264743.90) | 253832.13(297948.72,207509.63) | -1.41(-1.61,-1.20) | 291.76(335.94,243.09) | 178.19(207.98,145.19) | -2.37(-2.58,-2.16) |
| Ghana | both | 26882.29(41511.87,16463.70) | 48822.64(71285.79,31780.42) | 1.90(1.75,2.05) | 360.37(558.33,218.46) | 252.11(361.31,165.08) | -1.33(-1.46,-1.21) |
| Greece | both | 17421.00(20416.47,14139.79) | 14135.66(16711.15,11488.52) | -0.70(-0.91,-0.48) | 120.07(140.01,98.20) | 79.62(94.60,65.02) | -1.30(-1.59,-1.02) |
| Greenland | both | 58.65(86.52,37.55) | 83.31(123.61,53.69) | 1.51(1.31,1.70) | 118.89(174.24,76.93) | 106.98(153.23,70.43) | -0.23(-0.31,-0.14) |
| Grenada | both | 163.26(198.59,128.41) | 242.86(297.32,189.94) | 1.57(1.44,1.70) | 262.98(321.20,205.11) | 202.10(246.73,159.07) | -0.94(-1.17,-0.70) |
| Guam | both | 116.50(162.83,80.78) | 171.94(249.91,113.78) | 1.25(1.17,1.34) | 120.20(167.63,83.47) | 90.21(130.49,60.18) | -1.05(-1.27,-0.83) |
| Guatemala | both | 26620.35(33369.94,20119.85) | 63859.72(86002.03,45057.58) | 2.76(2.52,3.00) | 568.95(709.92,432.28) | 498.03(667.90,355.16) | -0.72(-0.94,-0.49) |
| Guinea | both | 9574.73(13912.88,6244.40) | 15339.07(22615.95,9733.81) | 1.48(1.35,1.62) | 267.07(386.83,173.47) | 243.21(362.83,154.87) | -0.27(-0.37,-0.16) |
| Guinea-Bissau | both | 1935.57(3002.67,1087.57) | 3413.39(5045.13,2163.45) | 1.82(1.67,1.97) | 412.28(638.60,232.85) | 373.23(544.75,239.08) | -0.49(-0.58,-0.39) |
| Guyana | both | 2857.42(3611.63,2189.81) | 3583.51(4846.60,2541.24) | 1.07(0.83,1.30) | 619.10(779.48,477.36) | 491.68(659.98,353.39) | -0.56(-0.80,-0.32) |
| Haiti | both | 16683.00(23649.96,8808.04) | 25655.27(39241.21,12902.85) | 1.57(1.38,1.76) | 437.99(615.00,236.24) | 306.29(465.29,155.97) | -1.13(-1.28,-0.98) |
| Honduras | both | 10055.30(13535.02,7123.34) | 28158.82(42596.18,17135.26) | 3.73(3.62,3.83) | 414.78(556.28,296.04) | 426.73(642.29,259.53) | 0.18(0.07,0.30) |
| Hungary | both | 84115.22(98676.33,69873.04) | 45998.99(58982.51,35274.50) | -3.39(-3.91,-2.87) | 636.58(747.99,524.77) | 295.00(379.55,228.07) | -4.09(-4.65,-3.53) |
| Iceland | both | 162.72(196.47,129.29) | 213.74(263.40,166.75) | 0.81(0.74,0.88) | 63.28(76.76,50.06) | 45.08(55.28,35.44) | -1.40(-1.50,-1.30) |
| India | both | 1122305.87(1382546.11,937579.01) | 2025620.81(2565144.45,1589227.01) | 2.01(1.72,2.29) | 190.33(234.09,159.43) | 156.95(199.57,123.94) | -0.75(-1.03,-0.47) |
| Indonesia | both | 264709.24(331980.25,212628.74) | 409544.68(526282.30,319221.96) | 1.55(1.43,1.67) | 221.27(275.21,177.22) | 167.31(213.34,131.13) | -0.88(-1.00,-0.77) |
| Iran (Islamic Republic of) | both | 6495.06(8400.83,4949.78) | 12493.87(15351.56,9970.03) | 2.75(2.49,3.01) | 22.33(28.56,17.06) | 15.93(19.58,12.66) | -0.81(-1.03,-0.58) |
| Iraq | both | 2507.51(4170.11,1466.27) | 5776.47(9179.95,3445.98) | 2.80(2.67,2.94) | 29.47(48.68,17.03) | 22.03(34.46,13.23) | -1.11(-1.26,-0.96) |
| Ireland | both | 2319.24(2750.23,1873.80) | 5076.26(6025.98,4084.55) | 3.07(2.51,3.63) | 62.22(73.82,50.62) | 76.10(90.34,61.10) | 0.87(0.31,1.44) |
| Israel | both | 3649.70(4500.87,2847.84) | 6314.49(7780.83,4915.92) | 1.37(1.07,1.66) | 78.31(96.06,61.17) | 59.47(73.37,46.54) | -1.45(-1.71,-1.19) |
| Italy | both | 104718.06(117584.46,93180.46) | 55496.58(63948.57,47914.51) | -2.39(-2.59,-2.19) | 129.41(144.82,116.01) | 49.47(56.87,42.84) | -3.52(-3.72,-3.32) |
| Jamaica | both | 1460.10(1781.46,1124.43) | 1949.49(2692.32,1375.24) | 0.62(0.18,1.07) | 87.30(107.08,66.55) | 65.30(90.19,45.76) | -1.39(-1.83,-0.94) |
| Japan | both | 124524.34(141039.79,109894.68) | 91000.39(106457.71,76454.73) | -0.97(-1.11,-0.83) | 72.74(82.28,64.36) | 36.71(43.07,30.87) | -2.20(-2.35,-2.05) |
| Jordan | both | 430.82(684.43,262.99) | 1333.53(2017.46,818.25) | 3.62(3.33,3.91) | 28.84(46.15,17.46) | 18.27(27.74,11.33) | -1.77(-1.93,-1.60) |
| Kazakhstan | both | 23234.55(28203.87,18468.02) | 89914.72(116420.76,68114.88) | 5.40(4.68,6.11) | 170.58(205.93,135.81) | 466.80(597.13,359.23) | 4.18(3.37,4.98) |
| Kenya | both | 35310.65(62833.12,21630.53) | 99219.42(147741.94,67943.16) | 3.92(3.79,4.04) | 386.40(687.18,235.98) | 385.99(571.25,267.64) | 0.30(0.14,0.45) |
| Kiribati | both | 133.35(224.02,62.31) | 167.31(309.10,68.82) | 0.86(0.66,1.06) | 286.87(463.04,145.55) | 184.94(330.73,83.11) | -1.58(-1.76,-1.39) |
| Kuwait | both | 131.00(195.68,81.79) | 476.45(732.52,291.56) | 5.06(4.42,5.71) | 15.72(23.75,9.91) | 14.43(22.27,8.85) | 0.16(-0.71,1.03) |
| Kyrgyzstan | both | 10902.83(13183.67,8539.90) | 30508.25(37667.56,24027.30) | 3.67(3.13,4.20) | 339.09(410.21,266.49) | 541.12(664.76,427.27) | 1.66(1.02,2.30) |
| Lao People's Democratic Republic | both | 5324.72(8001.90,3093.51) | 8708.18(13447.48,5245.43) | 1.44(1.16,1.72) | 215.29(319.98,127.63) | 162.10(249.73,97.89) | -1.26(-1.48,-1.05) |
| Latvia | both | 3010.93(3677.26,2346.14) | 5756.14(7533.92,4330.47) | 1.95(1.20,2.70) | 88.84(108.43,69.69) | 208.52(273.35,153.24) | 2.70(1.93,3.48) |
| Lebanon | both | 614.96(965.69,346.38) | 953.09(1662.57,473.42) | 1.72(1.52,1.91) | 25.56(39.37,14.43) | 18.37(32.13,9.09) | -0.92(-1.16,-0.68) |
| Lesotho | both | 2054.98(3680.11,1158.11) | 3218.45(5051.05,1875.00) | 1.54(1.37,1.71) | 188.90(335.63,105.49) | 216.80(339.83,127.89) | 0.59(0.45,0.73) |
| Liberia | both | 4227.15(6168.90,2792.50) | 6849.46(10373.62,4108.23) | 1.31(1.08,1.53) | 357.56(521.94,236.48) | 266.73(398.12,161.30) | -1.41(-1.55,-1.27) |
| Libya | both | 661.72(1140.10,345.42) | 1419.25(2249.80,735.97) | 2.75(2.44,3.07) | 32.77(57.12,16.76) | 25.04(40.06,13.14) | -0.88(-1.05,-0.72) |
| Lithuania | both | 4581.20(5523.83,3578.73) | 13171.00(17476.17,10020.33) | 4.47(3.24,5.70) | 106.78(129.96,83.58) | 334.04(440.60,250.97) | 4.82(3.62,6.02) |
| Luxembourg | both | 1381.47(1612.32,1145.49) | 1200.76(1455.55,945.85) | -0.73(-0.84,-0.61) | 277.44(323.68,229.00) | 132.90(159.84,104.53) | -2.74(-2.84,-2.65) |
| Madagascar | both | 15527.28(22383.18,10167.38) | 30203.83(45771.74,19108.95) | 2.32(2.19,2.45) | 267.16(384.93,177.14) | 222.62(333.10,140.60) | -0.70(-0.79,-0.61) |
| Malawi | both | 16304.91(24056.54,10823.56) | 24029.09(34197.69,15945.15) | 0.98(0.82,1.15) | 369.61(547.95,243.99) | 283.69(409.91,188.68) | -1.17(-1.41,-0.94) |
| Malaysia | both | 7755.95(10776.66,5373.37) | 23831.83(35591.44,15320.17) | 3.65(3.39,3.91) | 71.84(100.44,49.23) | 81.09(120.55,52.06) | 0.02(-0.20,0.25) |
| Maldives | both | 105.54(165.24,56.43) | 202.40(286.93,139.40) | 1.84(1.30,2.39) | 94.24(145.01,52.15) | 49.65(69.24,34.78) | -2.54(-2.83,-2.25) |
| Mali | both | 15146.88(22587.93,9946.58) | 21087.94(33750.40,12958.51) | 0.91(0.71,1.11) | 321.49(474.02,211.77) | 208.06(330.39,129.64) | -1.75(-1.92,-1.58) |
| Malta | both | 458.19(550.73,363.85) | 485.91(597.47,384.32) | 0.26(0.14,0.38) | 107.23(129.15,85.36) | 69.73(84.72,55.02) | -1.48(-1.53,-1.43) |
| Marshall Islands | both | 41.29(62.67,23.85) | 69.16(122.22,34.62) | 1.71(1.67,1.75) | 201.67(301.34,118.83) | 148.82(260.27,77.20) | -1.13(-1.22,-1.04) |
| Mauritania | both | 3475.57(5018.43,2183.14) | 3793.57(6194.81,2117.73) | 0.20(0.05,0.36) | 320.20(461.68,202.83) | 162.80(259.66,92.86) | -2.43(-2.54,-2.33) |
| Mauritius | both | 1725.50(2329.68,1202.18) | 1935.90(2778.86,1272.09) | -0.22(-0.64,0.20) | 198.63(268.85,138.91) | 109.20(156.15,72.87) | -2.70(-3.03,-2.37) |
| Mexico | both | 284339.79(312944.47,255559.27) | 508318.49(622506.49,408038.24) | 1.68(1.51,1.85) | 553.38(609.83,498.69) | 403.80(494.74,325.00) | -1.44(-1.61,-1.26) |
| Micronesia (Federated States of) | both | 119.66(195.02,64.01) | 132.45(246.29,57.44) | 0.36(0.21,0.52) | 211.84(335.13,116.99) | 148.48(268.86,67.29) | -1.24(-1.41,-1.07) |
| Monaco | both | 67.96(89.61,49.96) | 82.19(106.76,60.13) | 0.87(0.76,0.97) | 135.37(179.63,101.30) | 125.00(164.24,90.93) | -0.09(-0.18,0.00) |
| Mongolia | both | 7648.87(10034.17,5547.63) | 21455.66(28842.07,15501.24) | 3.85(3.65,4.04) | 661.58(867.91,485.30) | 746.81(993.86,546.47) | 0.55(0.22,0.89) |
| Montenegro | both | 472.43(609.32,363.26) | 700.28(937.62,516.04) | 1.11(0.92,1.31) | 71.79(92.76,55.53) | 77.30(103.48,57.31) | 0.02(-0.14,0.19) |
| Morocco | both | 4469.42(6795.84,2631.50) | 8939.85(14247.04,5218.61) | 2.40(2.14,2.67) | 30.56(46.73,17.80) | 26.88(41.35,15.95) | -0.44(-0.68,-0.19) |
| Mozambique | both | 8878.94(13104.87,5687.19) | 15164.08(25190.53,8103.25) | 2.13(1.96,2.29) | 132.09(192.68,85.48) | 117.04(194.28,62.97) | -0.20(-0.40,0.01) |
| Myanmar | both | 88908.78(133364.95,55671.47) | 149952.20(219924.27,97576.91) | 1.80(1.69,1.92) | 310.78(466.64,193.96) | 269.94(393.34,178.61) | -0.53(-0.65,-0.42) |
| Namibia | both | 1473.79(2430.12,827.65) | 2774.53(4189.84,1746.44) | 1.62(1.35,1.90) | 189.83(313.88,106.42) | 172.70(259.64,108.90) | -0.77(-1.01,-0.53) |
| Nauru | both | 11.73(20.66,4.91) | 10.94(19.57,4.59) | -0.41(-0.51,-0.30) | 198.27(334.59,87.23) | 159.26(273.55,72.66) | -0.84(-0.93,-0.75) |
| Nepal | both | 52538.58(76479.62,36325.15) | 95128.92(137786.23,63674.17) | 2.45(2.19,2.71) | 444.60(652.93,305.65) | 387.35(565.45,262.65) | -0.19(-0.45,0.07) |
| Netherlands | both | 15321.07(17886.64,12698.44) | 16688.39(20022.88,13203.36) | -0.16(-0.52,0.20) | 83.70(97.49,68.97) | 57.57(68.74,45.87) | -1.78(-2.14,-1.43) |
| New Zealand | both | 930.99(1096.32,783.31) | 1218.77(1472.56,1008.28) | 0.98(0.91,1.05) | 25.27(29.89,21.22) | 17.78(21.50,14.71) | -1.28(-1.33,-1.22) |
| Nicaragua | both | 5336.92(6437.96,4246.09) | 18005.52(23812.73,13246.20) | 4.37(4.18,4.56) | 296.80(359.77,236.52) | 361.93(478.06,269.98) | 0.80(0.62,0.97) |
| Niger | both | 9846.64(15200.44,6082.08) | 21355.25(35041.44,12494.51) | 2.38(2.20,2.56) | 289.90(446.26,181.87) | 226.25(372.61,133.62) | -1.24(-1.41,-1.07) |
| Nigeria | both | 165122.10(245236.39,106047.27) | 263211.42(413700.98,163186.59) | 1.86(1.72,2.01) | 335.31(500.04,215.88) | 253.78(394.08,160.91) | -0.69(-0.86,-0.52) |
| Niue | both | 2.86(4.39,1.72) | 2.26(3.40,1.37) | -1.28(-1.52,-1.04) | 140.95(216.95,84.30) | 106.55(157.60,64.58) | -1.13(-1.26,-1.00) |
| North Macedonia | both | 2334.51(2949.98,1862.79) | 4015.30(5419.26,2895.45) | 1.68(1.57,1.80) | 114.70(144.50,91.50) | 125.22(168.88,90.87) | 0.06(-0.05,0.18) |
| Northern Mariana Islands | both | 65.34(97.33,41.04) | 74.67(110.95,48.55) | 0.41(0.01,0.81) | 214.57(307.76,141.89) | 120.91(170.16,81.49) | -2.24(-2.42,-2.07) |
| Norway | both | 3952.92(4403.24,3539.34) | 3752.90(4313.59,3291.92) | -0.04(-0.12,0.05) | 74.34(82.95,66.44) | 46.48(53.29,40.78) | -1.56(-1.68,-1.44) |
| Oman | both | 316.92(529.06,175.57) | 649.08(1133.47,348.70) | 3.14(2.88,3.40) | 40.77(66.95,22.31) | 29.92(48.73,17.03) | -0.44(-0.86,-0.02) |
| Pakistan | both | 82208.34(135958.52,46607.37) | 138131.19(207691.66,91203.34) | 1.69(1.64,1.75) | 130.72(217.52,72.35) | 103.40(157.60,67.05) | -0.94(-1.08,-0.80) |
| Palau | both | 14.38(24.84,6.90) | 26.81(45.71,13.68) | 2.03(1.64,2.42) | 124.20(212.03,61.54) | 106.23(179.16,55.78) | -0.41(-0.51,-0.30) |
| Palestine | both | 303.53(562.86,159.94) | 664.33(1020.61,408.96) | 3.10(2.86,3.35) | 33.98(62.54,18.16) | 26.30(40.14,16.30) | -0.56(-0.76,-0.36) |
| Panama | both | 2022.50(2408.02,1614.40) | 4838.37(6584.21,3455.72) | 3.39(3.17,3.60) | 127.21(152.29,101.14) | 115.93(157.63,83.04) | -0.03(-0.22,0.16) |
| Papua New Guinea | both | 1792.70(2628.53,1159.10) | 4633.92(7296.16,2760.19) | 3.60(3.41,3.79) | 70.05(103.19,45.05) | 66.87(103.42,40.52) | 0.00(-0.10,0.09) |
| Paraguay | both | 3342.35(4106.89,2663.13) | 10384.11(14101.14,7282.91) | 3.96(3.73,4.19) | 135.93(166.98,107.56) | 172.24(234.41,121.77) | 0.80(0.57,1.02) |
| Peru | both | 45413.68(58477.21,33838.68) | 76574.94(108644.16,50931.12) | 1.65(1.42,1.87) | 339.67(436.92,253.15) | 233.08(332.68,155.35) | -1.46(-1.66,-1.25) |
| Philippines | both | 44979.59(59749.06,33770.93) | 95638.91(124489.72,71847.66) | 2.50(2.36,2.64) | 120.88(163.27,89.81) | 104.04(134.04,78.14) | -0.66(-0.83,-0.50) |
| Poland | both | 71569.43(77451.60,65854.63) | 125279.72(154007.35,101007.55) | 2.20(1.84,2.56) | 165.02(179.06,151.48) | 220.12(272.44,176.42) | 1.18(0.85,1.52) |
| Portugal | both | 50045.66(58029.10,41408.08) | 26680.36(31396.42,21694.18) | -2.43(-2.54,-2.32) | 396.70(459.51,328.34) | 151.94(178.52,123.63) | -3.61(-3.73,-3.48) |
| Puerto Rico | both | 12994.81(15350.95,10341.64) | 10659.46(14536.15,7663.67) | -1.53(-1.88,-1.17) | 364.37(430.67,290.55) | 188.33(255.07,134.44) | -3.13(-3.51,-2.75) |
| Qatar | both | 80.96(129.02,45.36) | 473.35(788.55,267.10) | 6.67(6.45,6.88) | 55.25(88.55,31.82) | 41.92(66.90,23.89) | -0.88(-1.24,-0.52) |
| Republic of Korea | both | 196776.68(240671.96,151961.60) | 132477.94(161197.54,104540.45) | -2.45(-2.81,-2.09) | 523.50(635.76,408.02) | 151.08(182.81,119.44) | -5.33(-5.68,-4.96) |
| Republic of Moldova | both | 42187.23(49131.86,34149.73) | 39317.82(48349.23,30835.31) | -0.19(-0.60,0.22) | 897.63(1043.15,732.51) | 716.53(880.81,563.68) | -0.70(-1.13,-0.27) |
| Romania | both | 104215.34(122811.50,85876.69) | 137630.49(177569.03,105086.37) | 0.37(-0.07,0.82) | 369.06(435.07,305.78) | 443.34(575.62,337.96) | 0.03(-0.42,0.49) |
| Russian Federation | both | 191858.29(217277.80,169577.98) | 666445.50(798352.19,550271.06) | 4.33(3.34,5.33) | 105.69(119.73,93.43) | 328.47(392.51,270.12) | 4.00(2.98,5.03) |
| Rwanda | both | 15458.51(21875.84,9728.95) | 17340.94(25895.98,11215.60) | 0.07(-0.41,0.55) | 474.28(672.30,301.12) | 242.12(359.00,156.55) | -3.27(-3.67,-2.87) |
| Saint Kitts and Nevis | both | 117.00(141.92,91.11) | 166.34(223.74,110.72) | 0.78(0.17,1.41) | 367.99(450.05,284.81) | 216.68(289.37,146.02) | -2.44(-2.90,-1.99) |
| Saint Lucia | both | 254.28(304.02,200.91) | 409.96(525.25,314.20) | 1.46(1.02,1.90) | 288.67(344.72,227.76) | 184.42(235.40,141.98) | -1.86(-2.30,-1.41) |
| Saint Vincent and the Grenadines | both | 133.67(164.68,103.10) | 255.67(327.46,194.61) | 2.17(1.71,2.63) | 190.70(234.61,146.48) | 184.92(234.64,141.53) | -0.36(-0.78,0.06) |
| Samoa | both | 136.86(216.92,85.14) | 171.52(257.78,104.20) | 0.83(0.74,0.93) | 139.84(223.02,87.64) | 103.04(153.03,63.71) | -1.04(-1.14,-0.94) |
| San Marino | both | 48.64(62.26,36.74) | 75.15(113.48,46.48) | 1.66(1.56,1.75) | 160.93(205.77,121.95) | 141.41(217.94,86.99) | -0.34(-0.46,-0.23) |
| Sao Tome and Principe | both | 254.92(396.72,150.35) | 409.20(619.70,267.45) | 1.33(1.13,1.52) | 374.72(580.50,222.54) | 328.58(495.67,216.56) | -0.72(-0.97,-0.48) |
| Saudi Arabia | both | 4038.05(6984.59,2171.05) | 7061.63(10833.35,4245.44) | 1.29(0.99,1.60) | 64.75(111.26,33.99) | 35.69(55.27,21.97) | -2.30(-2.48,-2.13) |
| Senegal | both | 8813.85(13914.65,5574.41) | 16619.04(26227.51,10325.76) | 2.30(2.06,2.54) | 243.40(381.77,151.69) | 192.36(303.95,119.70) | -0.76(-0.99,-0.53) |
| Serbia | both | 18950.23(23829.49,14635.93) | 18015.83(24152.80,13202.15) | -0.59(-0.79,-0.39) | 157.37(197.10,123.10) | 131.63(178.11,96.52) | -0.98(-1.16,-0.80) |
| Seychelles | both | 104.02(144.52,70.48) | 249.85(346.17,167.14) | 2.89(2.66,3.12) | 188.94(262.37,127.04) | 198.62(270.39,133.92) | -0.14(-0.40,0.12) |
| Sierra Leone | both | 6285.68(9544.80,3962.75) | 8484.79(12549.96,5322.72) | 0.70(0.42,0.98) | 307.07(465.29,191.81) | 198.29(294.68,124.40) | -1.92(-2.16,-1.68) |
| Singapore | both | 924.82(1262.62,644.98) | 1109.40(1542.80,761.14) | 0.61(0.30,0.93) | 36.08(50.06,25.27) | 13.42(18.59,9.24) | -3.45(-3.67,-3.23) |
| Slovakia | both | 21938.10(26705.96,17487.99) | 25221.93(34290.37,18331.34) | 1.05(0.77,1.33) | 381.18(465.18,302.13) | 306.93(418.79,223.32) | -0.25(-0.51,0.01) |
| Slovenia | both | 10018.51(13790.71,7167.43) | 7239.54(9959.48,5169.68) | -1.34(-1.59,-1.10) | 415.57(572.35,297.04) | 205.25(283.15,146.80) | -2.69(-2.95,-2.44) |
| Solomon Islands | both | 432.63(671.60,260.12) | 929.82(1359.17,599.10) | 2.79(2.69,2.89) | 230.91(360.91,138.86) | 201.06(291.21,131.29) | -0.37(-0.46,-0.28) |
| Somalia | both | 10132.34(16048.15,5696.94) | 22387.27(36920.90,12821.15) | 2.71(2.61,2.81) | 325.52(508.65,186.28) | 272.05(443.11,156.56) | -0.61(-0.69,-0.52) |
| South Africa | both | 29657.97(39118.67,22203.02) | 40839.34(49123.45,33628.13) | 0.73(0.16,1.31) | 121.43(162.61,90.34) | 81.11(97.50,66.63) | -1.67(-2.16,-1.17) |
| South Sudan | both | 6887.52(11957.59,4085.27) | 9653.24(16756.70,5430.00) | 1.31(1.17,1.45) | 262.44(453.17,157.67) | 217.06(363.90,125.47) | -0.57(-0.63,-0.51) |
| Spain | both | 129319.88(150582.65,106361.03) | 82842.03(98058.52,67676.80) | -1.56(-1.69,-1.42) | 263.78(305.70,217.47) | 105.97(124.52,86.76) | -3.23(-3.37,-3.10) |
| Sri Lanka | both | 23036.92(31482.82,15649.15) | 30502.91(45947.01,19649.52) | -0.02(-0.72,0.69) | 174.47(238.76,119.49) | 116.67(174.64,75.91) | -2.31(-2.95,-1.67) |
| Sudan | both | 4553.48(7442.51,2422.40) | 7400.89(13597.84,3794.67) | 1.46(1.35,1.57) | 45.90(75.61,24.63) | 36.31(67.28,18.52) | -0.88(-0.93,-0.84) |
| Suriname | both | 826.77(1004.02,637.42) | 1536.24(2005.22,1123.45) | 1.96(1.75,2.17) | 290.10(353.15,224.45) | 240.30(311.60,176.66) | -1.04(-1.25,-0.83) |
| Sweden | both | 10926.84(11826.90,10056.43) | 11717.21(13136.45,10407.66) | 0.51(0.34,0.68) | 93.60(101.30,85.95) | 71.46(80.73,63.52) | -0.72(-0.88,-0.55) |
| Switzerland | both | 11756.02(13616.25,9624.43) | 11366.28(13552.22,8934.56) | -0.33(-0.46,-0.20) | 131.42(152.41,107.73) | 79.32(94.37,62.58) | -1.99(-2.16,-1.83) |
| Syrian Arab Republic | both | 2052.53(3171.12,1202.92) | 3818.73(6238.69,2101.09) | 2.36(2.15,2.56) | 35.88(56.23,20.83) | 29.10(46.72,16.99) | -0.66(-0.85,-0.46) |
| Taiwan (Province of China) | both | 34879.00(43912.20,27067.00) | 50170.43(71041.75,33593.73) | 0.94(0.54,1.34) | 196.24(246.12,152.11) | 137.38(195.75,91.91) | -1.60(-1.93,-1.27) |
| Tajikistan | both | 8453.34(10556.39,6541.64) | 26750.40(35444.78,19375.58) | 3.78(3.51,4.06) | 272.61(336.80,209.96) | 403.10(525.00,298.85) | 1.16(0.99,1.33) |
| Thailand | both | 84056.03(114073.80,59397.27) | 181519.81(273489.41,117880.19) | 2.94(2.80,3.09) | 189.09(253.21,134.44) | 179.03(265.92,118.40) | -0.01(-0.14,0.12) |
| Timor-Leste | both | 605.27(1076.52,329.34) | 1260.89(2096.56,615.16) | 2.40(2.10,2.69) | 146.96(256.49,79.55) | 143.18(238.78,71.11) | -0.36(-0.63,-0.09) |
| Togo | both | 4308.16(6264.62,2883.74) | 9692.58(14553.32,6098.18) | 2.49(2.26,2.73) | 288.84(422.32,192.39) | 210.22(312.58,134.63) | -1.47(-1.70,-1.24) |
| Tokelau | both | 1.47(2.28,0.89) | 1.06(1.60,0.64) | -1.33(-1.66,-1.00) | 116.74(179.20,70.87) | 78.77(118.05,47.43) | -1.41(-1.51,-1.30) |
| Tonga | both | 96.31(139.25,62.13) | 112.62(163.19,71.37) | 0.59(0.39,0.79) | 155.37(221.55,99.92) | 135.50(195.97,86.16) | -0.46(-0.65,-0.27) |
| Trinidad and Tobago | both | 1779.38(2134.94,1402.80) | 2370.34(3370.68,1604.67) | 0.99(0.81,1.17) | 195.40(232.80,152.57) | 128.62(183.77,87.34) | -1.51(-1.74,-1.29) |
| Tunisia | both | 1489.62(2467.20,844.99) | 2722.17(4619.22,1492.45) | 2.14(2.11,2.17) | 27.94(45.97,16.08) | 20.87(35.03,11.50) | -0.92(-0.95,-0.89) |
| Turkey | both | 9502.88(15341.19,5446.31) | 15064.58(22655.39,9297.43) | 1.55(1.31,1.79) | 24.26(38.61,14.03) | 16.59(25.01,10.28) | -1.29(-1.52,-1.06) |
| Turkmenistan | both | 8348.67(10184.18,6652.96) | 34097.59(45013.54,24698.78) | 5.19(4.88,5.50) | 377.69(457.96,300.14) | 698.61(909.60,513.53) | 2.25(1.96,2.55) |
| Tuvalu | both | 12.78(20.48,6.86) | 13.47(21.80,7.41) | 0.31(0.06,0.56) | 166.01(262.35,90.54) | 122.43(197.15,68.24) | -0.94(-1.15,-0.74) |
| Uganda | both | 19306.98(29813.59,12157.68) | 36508.81(52394.59,23996.68) | 1.76(1.52,2.00) | 269.10(410.59,170.10) | 219.18(311.25,144.36) | -1.11(-1.34,-0.88) |
| Ukraine | both | 93078.81(106469.77,81522.42) | 299138.11(372466.44,237024.26) | 3.96(3.13,4.79) | 135.40(154.70,118.84) | 491.58(611.44,390.22) | 4.40(3.50,5.31) |
| United Arab Emirates | both | 179.56(310.76,102.17) | 1557.12(2671.81,796.21) | 7.38(7.04,7.71) | 32.45(56.83,17.80) | 26.01(44.46,13.02) | -0.92(-1.33,-0.51) |
| United Kingdom | both | 74206.42(80697.95,67359.41) | 156977.07(171418.54,143902.22) | 2.63(2.11,3.15) | 102.46(111.46,93.34) | 168.08(183.03,153.64) | 1.66(1.12,2.20) |
| United Republic of Tanzania | both | 32534.43(46398.58,21449.53) | 56904.64(83457.08,35755.02) | 1.93(1.74,2.11) | 266.08(379.04,175.78) | 199.58(293.06,127.04) | -0.94(-1.16,-0.72) |
| United States of America | both | 311781.35(356259.43,271300.95) | 475596.95(562531.31,399466.44) | 1.95(1.78,2.12) | 109.76(125.77,94.69) | 98.87(117.06,83.15) | -0.01(-0.14,0.12) |
| United States Virgin Islands | both | 243.87(308.92,180.76) | 383.02(497.05,281.08) | 1.93(1.67,2.19) | 245.30(309.02,182.61) | 229.54(298.40,165.98) | 0.03(-0.24,0.30) |
| Uruguay | both | 4029.09(5358.99,2870.06) | 3392.12(4526.94,2425.08) | -0.95(-1.13,-0.76) | 109.80(145.54,79.18) | 72.48(96.40,51.71) | -1.75(-1.90,-1.60) |
| Uzbekistan | both | 41800.35(51252.04,32744.46) | 176777.85(225388.54,135143.47) | 4.33(3.86,4.81) | 331.87(405.10,260.27) | 622.60(781.39,481.80) | 1.53(0.99,2.08) |
| Vanuatu | both | 154.24(256.64,83.85) | 313.94(514.95,166.07) | 2.37(2.22,2.53) | 185.43(305.64,105.93) | 151.97(246.13,82.13) | -0.92(-1.07,-0.76) |
| Venezuela (Bolivarian Republic of) | both | 24928.09(29525.65,20020.50) | 55355.66(77575.32,38027.11) | 2.73(2.49,2.98) | 223.13(265.76,179.54) | 178.55(247.77,123.34) | -0.95(-1.19,-0.71) |
| Viet Nam | both | 82167.92(125167.16,51168.82) | 162157.61(236525.16,105457.97) | 2.20(1.64,2.76) | 195.16(295.03,121.40) | 152.54(219.49,100.09) | -1.16(-1.68,-0.64) |
| Yemen | both | 2538.14(4190.18,1349.85) | 5356.34(8698.22,3002.41) | 2.50(2.43,2.57) | 46.35(78.04,24.60) | 35.98(58.16,19.92) | -1.00(-1.06,-0.94) |
| Zambia | both | 14018.38(21464.52,9100.48) | 31700.03(46336.84,20606.94) | 2.45(2.22,2.68) | 429.52(650.93,279.11) | 384.00(552.91,253.89) | -0.72(-0.95,-0.49) |
| Zimbabwe | both | 8831.83(12329.98,5962.95) | 14854.75(23545.29,8316.39) | 1.69(1.53,1.85) | 190.94(265.15,129.16) | 178.29(283.06,102.19) | -0.09(-0.31,0.13) |
| Afghanistan | male | 2928.47(5105.22,1394.86) | 4456.05(7643.15,2175.75) | 0.91(0.59,1.24) | 74.94(127.18,36.20) | 60.44(98.84,31.81) | -1.01(-1.15,-0.88) |
| Albania | male | 2093.49(2578.42,1639.68) | 2590.85(3622.11,1771.86) | 1.19(0.85,1.52) | 187.95(231.35,148.19) | 136.44(192.68,93.15) | -0.64(-1.04,-0.25) |
| Algeria | male | 2659.88(4220.64,1547.73) | 5441.43(9107.40,2990.78) | 2.33(2.09,2.58) | 41.52(64.24,24.69) | 29.88(49.81,16.32) | -1.34(-1.54,-1.14) |
| American Samoa | male | 23.27(33.24,15.50) | 27.97(41.02,17.78) | 0.42(0.27,0.56) | 148.92(211.58,99.13) | 106.26(154.86,68.70) | -1.26(-1.41,-1.11) |
| Andorra | male | 68.12(101.07,41.40) | 121.78(161.78,85.87) | 1.67(1.37,1.96) | 216.90(320.98,133.21) | 173.95(232.64,122.22) | -0.79(-0.81,-0.76) |
| Angola | male | 11968.55(18221.76,7275.14) | 26677.88(38644.32,17563.56) | 2.82(2.62,3.02) | 494.43(748.34,304.61) | 416.33(598.80,273.04) | -0.62(-0.72,-0.53) |
| Antigua and Barbuda | male | 70.77(85.60,53.97) | 109.40(139.64,83.11) | 1.67(1.34,2.00) | 317.56(385.53,239.33) | 210.90(266.86,160.94) | -1.46(-1.79,-1.13) |
| Argentina | male | 41663.77(52564.25,30156.00) | 57634.09(75265.60,41734.83) | 1.34(1.00,1.69) | 274.18(345.33,199.29) | 242.09(315.72,176.20) | -0.24(-0.51,0.04) |
| Armenia | male | 3182.25(3843.20,2513.58) | 8014.25(10125.71,6087.55) | 3.49(2.99,3.99) | 237.16(284.62,190.01) | 458.84(575.79,347.86) | 2.64(2.12,3.17) |
| Australia | male | 6020.15(8166.88,4241.61) | 8257.83(11377.65,5669.76) | 1.75(1.48,2.01) | 66.19(89.73,46.48) | 48.81(67.65,33.16) | -0.48(-0.76,-0.21) |
| Austria | male | 24404.88(27926.79,20351.63) | 17916.42(20819.19,14743.52) | -1.13(-1.23,-1.04) | 539.43(618.29,450.35) | 257.91(298.34,212.11) | -2.66(-2.76,-2.57) |
| Azerbaijan | male | 9533.42(11929.46,7487.07) | 23335.09(31898.89,16716.68) | 2.36(2.08,2.64) | 397.14(484.77,313.76) | 481.73(650.49,355.04) | -0.03(-0.34,0.29) |
| Bahamas | male | 386.94(475.30,299.22) | 644.50(863.15,470.48) | 1.63(1.39,1.87) | 464.66(567.94,360.35) | 309.66(411.71,227.83) | -1.59(-1.90,-1.28) |
| Bahrain | male | 77.32(118.27,47.56) | 291.74(491.61,163.49) | 4.78(4.51,5.06) | 66.77(103.24,40.72) | 36.08(56.85,21.30) | -2.49(-2.82,-2.15) |
| Bangladesh | male | 124874.05(175540.35,82126.93) | 139474.20(200782.52,91813.18) | 0.12(-0.08,0.31) | 407.47(573.46,270.52) | 196.77(282.33,130.01) | -2.80(-3.00,-2.60) |
| Barbados | male | 300.78(360.72,240.42) | 444.52(577.87,325.47) | 1.05(0.75,1.36) | 274.43(330.50,217.49) | 210.77(274.06,155.25) | -1.35(-1.64,-1.06) |
| Belarus | male | 6808.31(8414.88,5279.30) | 23331.85(31625.37,17057.39) | 5.06(3.82,6.31) | 127.82(157.78,99.93) | 376.79(510.91,276.22) | 4.65(3.37,5.95) |
| Belgium | male | 14893.37(16748.52,12761.95) | 17696.04(20719.43,14667.29) | 0.48(0.33,0.63) | 240.00(269.66,206.47) | 201.48(235.29,168.79) | -0.79(-0.96,-0.62) |
| Belize | male | 133.25(163.89,104.42) | 678.85(847.31,526.71) | 5.48(5.11,5.85) | 266.12(325.84,206.91) | 410.84(513.47,320.48) | 1.10(0.67,1.53) |
| Benin | male | 4976.46(7218.89,3172.80) | 10560.77(15797.96,6831.30) | 2.46(2.36,2.56) | 479.74(704.08,306.35) | 371.57(551.18,237.86) | -1.05(-1.16,-0.94) |
| Bermuda | male | 90.53(109.66,72.10) | 71.60(93.63,53.92) | -0.70(-1.08,-0.32) | 302.31(365.70,240.58) | 133.21(172.57,101.26) | -2.78(-3.18,-2.39) |
| Bhutan | male | 592.68(1107.14,338.58) | 1155.57(2582.94,634.68) | 2.67(2.49,2.84) | 343.40(646.11,194.63) | 341.23(777.48,191.93) | 0.19(0.04,0.34) |
| Bolivia (Plurinational State of) | male | 11286.02(16220.19,7057.17) | 26375.90(37592.18,17660.03) | 3.01(2.95,3.08) | 649.05(933.76,406.96) | 573.42(809.67,383.72) | -0.45(-0.54,-0.36) |
| Bosnia and Herzegovina | male | 6667.98(8206.46,5159.79) | 5608.67(7650.46,4062.48) | -1.07(-1.31,-0.82) | 306.09(370.90,242.04) | 214.95(291.36,157.12) | -1.68(-1.99,-1.38) |
| Botswana | male | 1212.46(2137.83,640.03) | 2742.20(4164.95,1670.20) | 2.02(1.48,2.57) | 396.69(693.98,212.54) | 343.04(521.74,214.37) | -1.11(-1.70,-0.51) |
| Brazil | male | 292788.47(315475.95,271979.49) | 478492.49(522587.37,440802.30) | 1.84(1.72,1.96) | 549.51(591.20,509.93) | 407.77(446.10,376.06) | -0.92(-1.02,-0.82) |
| Brunei Darussalam | male | 96.61(136.67,63.51) | 208.25(300.42,138.99) | 2.12(1.89,2.36) | 126.16(181.52,82.19) | 94.01(132.31,63.06) | -1.53(-1.75,-1.31) |
| Bulgaria | male | 21996.05(26218.69,17723.32) | 31874.23(41844.51,23904.78) | 1.18(0.95,1.41) | 383.35(454.87,310.36) | 597.02(786.97,444.59) | 1.35(1.10,1.60) |
| Burkina Faso | male | 9164.55(12951.48,6068.80) | 14293.19(26724.35,6172.53) | 0.83(0.40,1.26) | 407.81(577.40,270.17) | 297.03(561.84,128.02) | -1.81(-2.23,-1.40) |
| Burundi | male | 6942.36(11424.52,3733.94) | 10855.57(22571.43,5584.74) | 1.18(0.91,1.44) | 589.49(962.81,316.21) | 366.95(755.94,186.25) | -2.10(-2.28,-1.92) |
| Cabo Verde | male | 244.81(391.53,155.59) | 761.83(1041.19,522.59) | 3.22(2.87,3.58) | 288.94(460.85,182.65) | 344.77(467.13,240.08) | -0.37(-0.75,0.01) |
| Cambodia | male | 14375.50(20736.81,9419.18) | 36547.63(52829.06,24319.52) | 3.31(3.08,3.54) | 579.39(829.03,376.63) | 579.66(848.28,384.58) | -0.05(-0.25,0.16) |
| Cameroon | male | 11139.39(16480.99,7288.90) | 24378.12(37312.57,14128.52) | 2.62(2.50,2.75) | 435.89(646.21,278.27) | 330.21(505.18,192.96) | -1.10(-1.26,-0.93) |
| Canada | male | 19233.97(24775.23,13966.52) | 30680.33(39433.83,21858.41) | 1.80(1.69,1.90) | 130.17(167.84,94.42) | 106.46(135.90,77.01) | -0.64(-0.76,-0.52) |
| Central African Republic | male | 3558.94(6109.62,1769.81) | 5352.88(9860.86,2478.11) | 1.13(1.00,1.26) | 537.59(921.58,277.13) | 414.33(737.39,211.81) | -1.23(-1.32,-1.15) |
| Chad | male | 5788.95(9270.30,3329.19) | 13684.54(20223.06,8784.79) | 3.02(2.94,3.10) | 402.96(647.98,229.32) | 383.60(570.60,244.39) | -0.22(-0.38,-0.07) |
| Chile | male | 28516.45(36619.46,20890.52) | 35338.28(45328.03,25390.93) | 1.08(0.84,1.32) | 558.27(715.45,408.91) | 317.37(404.63,230.32) | -1.70(-1.95,-1.45) |
| China | male | 731317.72(934925.86,561993.97) | 747176.58(970671.38,557407.92) | -0.01(-0.11,0.09) | 145.39(184.40,112.09) | 72.82(93.57,54.46) | -2.51(-2.60,-2.41) |
| Colombia | male | 15398.00(18271.58,12441.97) | 24819.83(34313.07,16924.32) | 1.19(0.92,1.45) | 157.64(187.72,127.27) | 101.95(140.96,69.72) | -1.99(-2.22,-1.76) |
| Comoros | male | 340.70(776.25,127.28) | 645.31(1106.32,345.30) | 2.01(1.71,2.32) | 294.20(665.93,114.25) | 259.28(449.79,140.00) | -0.62(-0.86,-0.38) |
| Congo | male | 2931.76(4429.28,1752.21) | 5043.26(7432.28,2993.24) | 1.65(1.32,1.98) | 525.08(787.06,326.19) | 315.42(462.44,188.34) | -2.05(-2.24,-1.86) |
| Cook Islands | male | 10.39(15.10,6.53) | 12.37(17.99,7.84) | 0.77(0.65,0.90) | 136.99(197.09,89.07) | 106.12(152.51,67.96) | -0.69(-0.84,-0.54) |
| Costa Rica | male | 2701.80(3221.86,2153.93) | 7583.01(10406.62,5382.39) | 2.98(2.69,3.28) | 279.93(333.52,225.36) | 306.16(418.69,217.45) | -0.30(-0.58,-0.02) |
| Côte d'Ivoire | male | 12947.29(20086.82,8155.94) | 25144.97(38098.35,15578.16) | 2.05(1.83,2.27) | 462.61(709.60,287.90) | 339.99(517.62,217.51) | -1.20(-1.41,-0.99) |
| Croatia | male | 17647.89(21144.70,14070.84) | 11805.85(15926.27,8665.40) | -1.41(-1.59,-1.23) | 600.04(714.88,483.90) | 348.13(463.72,256.69) | -1.91(-2.12,-1.70) |
| Cuba | male | 7689.08(9129.87,6153.46) | 20921.07(27691.97,15322.97) | 3.59(3.40,3.77) | 150.58(178.52,120.82) | 242.37(319.60,178.11) | 1.64(1.44,1.84) |
| Cyprus | male | 733.16(991.82,500.46) | 1106.56(1366.94,877.88) | 1.24(1.15,1.34) | 189.74(259.41,129.06) | 125.32(154.70,100.27) | -1.68(-1.80,-1.56) |
| Czechia | male | 22810.14(26632.21,19107.24) | 24033.55(31037.05,18316.75) | 0.59(0.43,0.76) | 384.97(449.22,321.26) | 294.20(380.68,225.89) | -0.61(-0.76,-0.45) |
| Democratic People's Republic of Korea | male | 17229.68(26875.80,9535.17) | 27656.23(42537.73,14068.69) | 1.56(1.37,1.76) | 202.41(306.42,118.17) | 174.75(261.58,89.15) | -0.52(-0.69,-0.34) |
| Democratic Republic of the Congo | male | 37680.83(55585.56,23975.14) | 73246.35(113976.49,42520.20) | 2.10(1.88,2.32) | 434.40(638.99,276.72) | 349.48(546.04,201.65) | -0.96(-1.07,-0.86) |
| Denmark | male | 8944.51(10344.36,7401.15) | 8685.75(10446.40,6796.19) | -0.64(-1.13,-0.16) | 284.77(329.34,234.82) | 196.35(235.41,154.66) | -1.90(-2.35,-1.44) |
| Djibouti | male | 322.90(667.70,187.76) | 1251.87(2271.44,700.14) | 4.63(4.51,4.75) | 337.28(684.01,198.46) | 303.75(561.34,172.49) | -0.59(-0.69,-0.49) |
| Dominica | male | 76.15(96.42,58.41) | 94.86(125.93,68.95) | 0.51(0.34,0.68) | 282.55(362.12,214.30) | 214.65(284.29,156.98) | -1.30(-1.51,-1.09) |
| Dominican Republic | male | 10775.68(13275.60,8412.87) | 23473.09(34264.64,14597.12) | 2.34(1.91,2.78) | 518.37(642.98,406.72) | 485.45(706.85,305.19) | -0.62(-1.10,-0.13) |
| Ecuador | male | 12224.83(15122.02,9545.25) | 29561.86(40817.90,20925.37) | 3.13(2.89,3.36) | 404.38(496.16,317.27) | 386.95(534.70,274.28) | -0.08(-0.28,0.12) |
| Egypt | male | 28104.44(42914.20,17909.00) | 62475.84(106252.89,31158.53) | 3.14(2.94,3.35) | 186.10(281.23,118.27) | 173.24(289.85,87.98) | 0.09(-0.12,0.30) |
| El Salvador | male | 8307.55(10153.89,6560.34) | 11908.29(16728.68,8155.85) | 0.81(0.52,1.10) | 525.46(641.89,414.74) | 475.62(670.19,324.92) | -0.75(-1.03,-0.46) |
| Equatorial Guinea | male | 405.46(710.84,203.78) | 489.93(858.66,258.93) | 0.31(-0.24,0.86) | 394.99(684.41,203.40) | 189.84(327.96,101.83) | -2.96(-3.29,-2.62) |
| Eritrea | male | 2787.40(4621.57,1505.43) | 7824.01(12174.33,4660.73) | 3.52(3.34,3.70) | 499.54(819.56,282.57) | 492.23(754.91,297.78) | -0.23(-0.42,-0.05) |
| Estonia | male | 1269.21(1573.29,1002.53) | 3253.59(4370.43,2338.41) | 3.14(2.32,3.96) | 151.79(187.12,120.85) | 374.00(503.90,267.74) | 2.97(2.09,3.85) |
| Eswatini | male | 599.28(956.45,356.37) | 1233.92(1866.21,752.43) | 2.54(2.10,2.99) | 373.07(596.76,224.46) | 403.97(596.86,253.99) | 0.54(0.19,0.88) |
| Ethiopia | male | 54896.22(86397.02,28600.39) | 69682.40(91506.76,51697.64) | 0.53(0.35,0.70) | 454.13(705.93,240.59) | 290.90(384.49,217.34) | -1.64(-1.75,-1.53) |
| Fiji | male | 281.47(410.78,179.02) | 424.05(655.00,261.32) | 1.53(1.41,1.65) | 113.63(164.98,74.88) | 97.06(147.22,61.70) | -0.59(-0.68,-0.49) |
| Finland | male | 7309.56(8569.02,5913.10) | 12356.45(14815.02,9747.38) | 2.67(2.03,3.31) | 241.96(284.58,196.44) | 300.67(358.48,237.10) | 1.52(0.98,2.06) |
| France | male | 132198.52(152606.85,109439.58) | 95296.15(113293.06,76385.58) | -1.18(-1.30,-1.05) | 395.33(458.29,328.16) | 196.21(233.14,157.01) | -2.53(-2.66,-2.40) |
| Gabon | male | 1507.13(2334.87,889.45) | 2126.29(3222.26,1309.09) | 1.04(0.86,1.23) | 534.95(832.02,318.79) | 360.78(542.27,224.63) | -1.52(-1.63,-1.42) |
| Gambia | male | 865.92(1394.41,507.90) | 2005.22(2980.46,1255.11) | 2.64(2.29,2.99) | 381.54(615.27,224.15) | 362.99(535.05,225.73) | -0.31(-0.59,-0.03) |
| Georgia | male | 14401.51(17546.37,11234.40) | 17141.45(21622.71,12953.52) | 1.09(0.80,1.38) | 528.84(638.42,418.48) | 759.86(960.70,569.91) | -0.24(-5.16,4.94) |
| Germany | male | 224800.70(257698.87,187445.08) | 185031.50(215338.40,152269.86) | -1.34(-1.55,-1.13) | 444.75(508.17,371.24) | 266.46(309.08,218.84) | -2.42(-2.63,-2.20) |
| Ghana | male | 19779.07(30850.34,11962.06) | 37951.49(56007.85,23949.53) | 2.16(2.03,2.29) | 542.65(847.15,326.51) | 430.60(627.77,273.36) | -0.79(-0.90,-0.68) |
| Greece | male | 13041.63(15110.79,10687.96) | 11068.62(13195.32,8991.62) | -0.43(-0.66,-0.20) | 191.81(221.82,157.99) | 132.86(157.48,108.23) | -1.05(-1.35,-0.75) |
| Greenland | male | 33.21(51.26,20.25) | 52.97(77.95,33.15) | 1.95(1.78,2.12) | 125.57(187.87,78.36) | 121.58(177.57,78.23) | 0.07(-0.01,0.14) |
| Grenada | male | 125.60(152.92,98.63) | 205.13(251.94,160.15) | 1.90(1.76,2.04) | 448.56(547.12,349.08) | 338.97(414.76,268.83) | -0.98(-1.16,-0.81) |
| Guam | male | 97.88(136.26,67.36) | 142.12(208.57,94.32) | 1.21(1.13,1.30) | 188.77(261.82,131.16) | 146.75(211.11,97.67) | -0.92(-1.07,-0.76) |
| Guatemala | male | 19892.51(25542.93,14819.19) | 48408.12(65773.39,34213.14) | 2.73(2.50,2.96) | 855.29(1082.27,642.45) | 821.17(1112.38,578.36) | -0.45(-0.66,-0.23) |
| Guinea | male | 6769.55(10467.39,3998.69) | 11460.20(16561.23,7441.78) | 1.74(1.60,1.88) | 379.49(583.22,224.46) | 362.90(526.97,233.12) | -0.11(-0.25,0.03) |
| Guinea-Bissau | male | 1450.97(2292.71,772.48) | 2517.00(3778.19,1563.24) | 1.74(1.57,1.91) | 646.15(1001.77,350.28) | 587.78(869.49,368.54) | -0.53(-0.63,-0.44) |
| Guyana | male | 2409.48(3060.12,1844.30) | 3026.50(4074.80,2160.29) | 1.08(0.85,1.32) | 1053.21(1330.98,804.24) | 857.87(1144.58,619.47) | -0.48(-0.73,-0.23) |
| Haiti | male | 12139.34(17817.08,6371.96) | 18681.98(28418.46,9401.31) | 1.57(1.35,1.80) | 645.15(934.19,348.33) | 471.33(715.13,246.15) | -0.98(-1.17,-0.80) |
| Honduras | male | 6490.38(8875.67,4441.68) | 16410.25(24531.92,9953.52) | 3.38(3.23,3.54) | 546.52(744.44,376.03) | 527.63(788.32,322.04) | -0.02(-0.20,0.16) |
| Hungary | male | 61328.06(72057.87,50343.28) | 34626.16(44565.05,26616.07) | -3.36(-3.92,-2.79) | 999.83(1174.63,816.77) | 478.80(615.41,370.02) | -4.01(-4.61,-3.41) |
| Iceland | male | 112.07(135.37,89.22) | 166.04(204.34,129.55) | 1.31(1.23,1.40) | 88.61(106.80,70.18) | 69.57(85.29,54.41) | -1.00(-1.09,-0.90) |
| India | male | 905509.27(1101749.51,747450.75) | 1615626.95(2097435.90,1253850.65) | 1.98(1.67,2.29) | 287.95(350.62,238.46) | 248.04(322.17,193.40) | -0.57(-0.87,-0.26) |
| Indonesia | male | 168713.38(215805.04,130301.58) | 272131.85(367548.25,197701.79) | 1.70(1.58,1.83) | 284.68(362.66,219.67) | 222.00(293.10,164.92) | -0.80(-0.91,-0.68) |
| Iran (Islamic Republic of) | male | 4834.02(6336.94,3637.11) | 9465.37(11685.29,7418.72) | 2.78(2.53,3.03) | 31.14(39.83,23.80) | 23.90(29.53,18.84) | -0.55(-0.78,-0.32) |
| Iraq | male | 1829.98(3211.74,1001.13) | 4343.24(6875.49,2551.83) | 2.86(2.73,3.00) | 43.20(74.86,23.71) | 33.34(52.07,19.91) | -1.04(-1.18,-0.89) |
| Ireland | male | 1518.36(1793.91,1237.22) | 3509.18(4184.89,2814.92) | 3.43(2.83,4.04) | 84.27(99.70,68.77) | 107.24(128.10,85.87) | 1.14(0.56,1.73) |
| Israel | male | 2525.27(3072.77,1992.78) | 4488.64(5480.21,3511.15) | 1.48(1.18,1.79) | 117.24(141.92,92.13) | 89.96(109.98,70.40) | -1.43(-1.71,-1.15) |
| Italy | male | 75971.55(84532.04,68223.04) | 39900.64(45748.06,34638.20) | -2.35(-2.55,-2.15) | 204.25(226.08,184.18) | 76.71(87.55,66.82) | -3.52(-3.70,-3.33) |
| Jamaica | male | 1117.42(1366.66,866.96) | 1454.19(2017.65,1016.28) | 0.50(0.00,1.01) | 140.21(171.01,107.68) | 99.27(137.36,69.31) | -1.59(-2.09,-1.09) |
| Japan | male | 98725.79(111944.81,87354.03) | 69785.03(81598.04,59025.41) | -1.12(-1.25,-0.98) | 123.51(138.99,109.53) | 60.44(70.97,50.92) | -2.33(-2.47,-2.19) |
| Jordan | male | 288.86(477.15,168.08) | 984.67(1537.68,591.85) | 3.98(3.60,4.36) | 36.70(62.65,22.00) | 24.86(38.40,15.32) | -1.50(-1.60,-1.40) |
| Kazakhstan | male | 14074.71(17268.17,11007.77) | 60552.62(80979.22,45515.85) | 5.76(4.94,6.59) | 247.93(301.29,197.20) | 703.31(932.78,534.49) | 4.36(3.51,5.21) |
| Kenya | male | 24865.96(47646.94,14784.56) | 72367.85(109653.19,48770.78) | 4.04(3.89,4.19) | 551.75(1075.84,326.23) | 574.65(872.90,397.41) | 0.40(0.19,0.60) |
| Kiribati | male | 104.54(180.27,44.30) | 129.59(247.40,49.74) | 0.81(0.60,1.02) | 465.16(770.73,214.68) | 301.47(564.96,121.57) | -1.57(-1.77,-1.37) |
| Kuwait | male | 108.75(162.99,67.52) | 402.91(629.95,238.68) | 5.10(4.43,5.77) | 19.91(30.21,12.43) | 20.36(32.21,12.18) | 0.44(-0.40,1.29) |
| Kyrgyzstan | male | 6885.09(8365.74,5402.77) | 22576.60(28042.19,17606.44) | 4.33(3.73,4.94) | 481.38(578.00,379.34) | 846.33(1038.63,667.13) | 2.12(1.45,2.79) |
| Lao People's Democratic Republic | male | 3843.08(5805.80,2198.68) | 6672.98(10611.76,3842.05) | 1.67(1.39,1.95) | 320.11(485.17,184.89) | 247.87(393.13,142.67) | -1.16(-1.39,-0.94) |
| Latvia | male | 1900.34(2318.58,1498.53) | 4010.06(5416.28,2832.23) | 2.21(1.43,2.99) | 134.68(163.27,107.25) | 321.05(433.56,226.79) | 2.72(1.94,3.50) |
| Lebanon | male | 432.73(689.87,226.46) | 674.92(1208.74,255.30) | 1.84(1.62,2.06) | 36.04(55.89,19.55) | 28.68(51.31,10.85) | -0.45(-0.74,-0.16) |
| Lesotho | male | 1606.97(3012.31,893.67) | 2459.90(3848.64,1393.26) | 1.27(1.04,1.50) | 318.87(588.13,179.31) | 377.45(600.61,220.17) | 0.55(0.35,0.74) |
| Liberia | male | 3174.98(4647.25,2023.51) | 5146.92(8029.80,2996.60) | 1.31(1.06,1.56) | 503.53(733.90,322.35) | 381.28(582.15,223.95) | -1.38(-1.54,-1.23) |
| Libya | male | 482.97(852.76,245.54) | 1038.71(1736.64,483.82) | 2.75(2.46,3.04) | 43.99(78.83,21.61) | 35.42(59.97,16.92) | -0.70(-0.83,-0.57) |
| Lithuania | male | 3110.90(3731.66,2460.55) | 9435.71(12462.18,7077.69) | 4.64(3.35,5.94) | 166.06(200.26,131.74) | 527.14(694.30,395.29) | 4.88(3.63,6.13) |
| Luxembourg | male | 1006.63(1172.53,841.09) | 881.91(1071.33,696.32) | -0.76(-0.90,-0.62) | 429.05(498.25,358.25) | 194.86(235.05,154.37) | -2.98(-3.09,-2.87) |
| Madagascar | male | 11337.23(17054.78,7247.14) | 21378.44(32997.15,13163.82) | 2.22(2.09,2.36) | 386.80(582.23,244.09) | 317.14(485.00,196.20) | -0.77(-0.87,-0.66) |
| Malawi | male | 12100.27(18283.37,7661.73) | 19062.63(27576.23,12272.91) | 1.21(1.05,1.38) | 573.77(864.62,368.54) | 479.58(688.61,310.17) | -0.84(-1.11,-0.57) |
| Malaysia | male | 6038.37(8393.01,4160.47) | 18525.57(27515.98,11927.42) | 3.73(3.49,3.96) | 110.53(155.67,75.90) | 122.66(180.38,78.61) | 0.05(-0.13,0.24) |
| Maldives | male | 72.94(111.85,39.35) | 163.44(234.47,110.66) | 2.42(1.80,3.04) | 115.26(174.52,63.89) | 66.42(93.12,45.49) | -2.14(-2.37,-1.90) |
| Mali | male | 10607.47(16141.76,6425.96) | 15299.67(25978.11,8642.68) | 1.09(0.87,1.31) | 450.13(684.58,275.16) | 292.69(489.30,168.36) | -1.70(-1.87,-1.53) |
| Malta | male | 323.49(388.66,257.26) | 360.46(438.94,287.38) | 0.49(0.36,0.63) | 163.64(196.79,129.98) | 103.85(126.41,82.62) | -1.53(-1.60,-1.45) |
| Marshall Islands | male | 33.85(51.90,19.10) | 55.49(97.61,27.71) | 1.66(1.62,1.70) | 319.82(483.03,184.79) | 230.14(398.31,117.79) | -1.15(-1.31,-1.00) |
| Mauritania | male | 2456.11(3720.33,1524.77) | 2703.46(4808.61,1405.65) | 0.24(0.08,0.41) | 462.99(700.22,286.26) | 232.08(410.67,123.65) | -2.46(-2.59,-2.34) |
| Mauritius | male | 1506.99(2032.90,1037.19) | 1631.72(2365.83,1064.58) | -0.36(-0.80,0.09) | 357.28(483.39,247.00) | 188.93(271.92,126.03) | -2.85(-3.21,-2.49) |
| Mexico | male | 228821.82(250742.55,206855.44) | 408373.12(515881.61,314687.70) | 1.70(1.52,1.87) | 907.19(994.38,821.97) | 682.36(860.67,524.01) | -1.31(-1.49,-1.14) |
| Micronesia (Federated States of) | male | 96.89(160.14,49.52) | 107.13(200.66,45.21) | 0.38(0.24,0.52) | 330.24(532.43,175.80) | 239.20(436.28,106.22) | -1.13(-1.26,-1.01) |
| Monaco | male | 49.77(65.66,36.79) | 58.48(75.79,42.89) | 0.74(0.66,0.83) | 213.38(279.69,159.04) | 186.03(243.33,136.06) | -0.31(-0.37,-0.25) |
| Mongolia | male | 4672.62(6249.98,3351.66) | 13868.53(18741.85,10018.65) | 4.11(3.88,4.33) | 858.30(1127.46,620.43) | 1010.74(1342.94,742.47) | 0.68(0.35,1.00) |
| Montenegro | male | 372.32(486.29,279.77) | 562.52(771.37,408.65) | 1.20(1.03,1.37) | 119.85(156.82,90.76) | 128.91(176.45,94.15) | 0.05(-0.11,0.20) |
| Morocco | male | 3063.62(4696.21,1695.16) | 6426.81(10173.35,3639.47) | 2.53(2.19,2.88) | 42.27(65.55,22.81) | 38.84(60.31,22.82) | -0.35(-0.65,-0.05) |
| Mozambique | male | 6145.98(9349.61,3756.30) | 10997.49(20277.08,5360.46) | 2.33(2.07,2.59) | 189.59(286.12,117.04) | 181.14(326.27,89.59) | 0.08(-0.24,0.41) |
| Myanmar | male | 76658.40(114317.69,47601.83) | 120676.70(177076.55,78243.40) | 1.58(1.47,1.69) | 551.88(824.83,342.87) | 470.46(683.84,308.47) | -0.58(-0.70,-0.45) |
| Namibia | male | 1132.43(1925.83,636.02) | 2193.86(3264.26,1362.03) | 1.70(1.42,1.98) | 311.75(527.83,173.73) | 306.75(457.01,191.40) | -0.50(-0.75,-0.25) |
| Nauru | male | 10.04(17.88,4.00) | 9.09(16.82,3.66) | -0.55(-0.66,-0.44) | 310.19(537.00,131.14) | 270.01(478.44,119.23) | -0.50(-0.58,-0.42) |
| Nepal | male | 38948.79(58637.12,26457.52) | 70370.19(108423.04,47046.14) | 2.42(2.17,2.66) | 639.93(977.20,434.87) | 608.73(938.84,408.80) | 0.10(-0.18,0.37) |
| Netherlands | male | 10310.89(11948.88,8525.79) | 11533.04(14004.75,9124.08) | 0.04(-0.30,0.39) | 120.78(139.46,100.24) | 82.54(98.61,65.36) | -1.71(-2.04,-1.37) |
| New Zealand | male | 655.88(778.91,547.57) | 896.18(1099.16,739.05) | 1.07(0.98,1.16) | 37.07(43.89,31.04) | 27.53(33.37,22.75) | -1.16(-1.25,-1.07) |
| Nicaragua | male | 4370.20(5290.10,3472.92) | 14515.62(19284.24,10596.86) | 4.29(4.08,4.50) | 507.48(613.73,401.93) | 628.40(827.48,463.10) | 0.84(0.63,1.06) |
| Niger | male | 7446.97(12195.41,4486.91) | 15488.48(26653.38,8199.59) | 2.27(2.08,2.46) | 414.37(675.79,244.70) | 333.91(575.37,178.62) | -1.11(-1.28,-0.93) |
| Nigeria | male | 132710.55(206953.49,78442.18) | 198261.91(333754.39,117783.49) | 1.61(1.46,1.76) | 486.27(755.71,286.23) | 406.41(682.52,243.94) | -0.31(-0.47,-0.15) |
| Niue | male | 2.30(3.55,1.35) | 1.82(2.78,1.10) | -1.28(-1.53,-1.02) | 238.42(367.33,140.34) | 175.51(258.83,107.61) | -1.22(-1.35,-1.08) |
| North Macedonia | male | 1824.02(2268.18,1433.15) | 3167.92(4327.01,2250.33) | 1.70(1.58,1.83) | 183.64(228.84,144.57) | 199.30(270.73,143.20) | 0.00(-0.13,0.14) |
| Northern Mariana Islands | male | 56.19(84.20,34.76) | 59.68(89.55,38.74) | 0.17(-0.26,0.60) | 295.82(426.43,195.79) | 183.71(260.30,123.23) | -1.96(-2.12,-1.80) |
| Norway | male | 2826.40(3143.12,2528.28) | 2690.97(3085.51,2366.27) | 0.02(-0.07,0.11) | 110.96(123.65,99.28) | 67.18(76.88,59.11) | -1.64(-1.77,-1.51) |
| Oman | male | 233.71(390.28,127.19) | 510.77(932.30,261.36) | 3.40(3.11,3.68) | 53.21(89.59,28.70) | 40.32(66.35,22.57) | -0.31(-0.75,0.14) |
| Pakistan | male | 43238.90(74255.82,22374.92) | 68694.12(116816.16,40542.58) | 1.64(1.57,1.70) | 124.41(216.01,62.74) | 92.83(158.80,53.84) | -1.00(-1.15,-0.85) |
| Palau | male | 12.29(21.24,5.96) | 23.05(40.05,11.61) | 2.04(1.61,2.48) | 209.08(355.36,103.49) | 170.63(289.85,88.99) | -0.53(-0.60,-0.46) |
| Palestine | male | 205.03(395.67,104.31) | 453.05(698.14,268.96) | 3.13(2.90,3.36) | 51.03(98.32,26.13) | 36.45(55.46,22.24) | -0.87(-1.07,-0.67) |
| Panama | male | 1462.12(1741.35,1162.14) | 3517.04(4838.03,2500.03) | 3.38(3.13,3.62) | 180.77(215.83,143.00) | 170.30(233.74,121.55) | 0.05(-0.17,0.27) |
| Papua New Guinea | male | 1637.86(2433.32,1048.20) | 4229.44(6731.84,2496.92) | 3.60(3.41,3.78) | 122.27(181.46,79.08) | 116.47(179.15,69.87) | -0.03(-0.12,0.07) |
| Paraguay | male | 2781.21(3410.00,2196.57) | 9259.00(12600.95,6486.56) | 4.19(3.97,4.41) | 230.30(280.63,181.68) | 310.35(422.94,218.99) | 0.99(0.76,1.21) |
| Peru | male | 33823.00(43505.50,25183.35) | 57216.22(82105.22,38061.79) | 1.76(1.55,1.96) | 510.36(658.90,380.41) | 357.74(513.65,236.36) | -1.29(-1.49,-1.10) |
| Philippines | male | 35097.31(46753.85,26262.19) | 76096.13(104073.58,54067.53) | 2.57(2.44,2.70) | 187.28(252.74,137.66) | 166.93(227.64,118.78) | -0.51(-0.67,-0.36) |
| Poland | male | 50386.49(54810.78,46016.70) | 95335.51(121828.79,73653.34) | 2.41(2.01,2.80) | 258.38(280.91,236.33) | 351.68(451.07,271.66) | 1.19(0.83,1.55) |
| Portugal | male | 37954.07(43737.83,31328.64) | 21402.81(24984.05,17417.77) | -2.17(-2.28,-2.07) | 647.91(748.09,537.95) | 261.46(304.33,212.66) | -3.37(-3.49,-3.24) |
| Puerto Rico | male | 11038.36(13033.80,8862.69) | 8579.28(11513.21,6144.58) | -1.72(-2.09,-1.35) | 663.88(785.17,533.00) | 335.39(452.22,238.93) | -3.23(-3.62,-2.84) |
| Qatar | male | 70.00(113.46,38.22) | 416.31(692.21,230.50) | 6.72(6.49,6.94) | 70.59(114.26,40.43) | 45.68(73.58,25.48) | -1.61(-1.97,-1.26) |
| Republic of Korea | male | 166803.26(204149.98,127640.21) | 110208.88(135277.02,86678.83) | -2.54(-2.91,-2.18) | 940.59(1137.66,735.45) | 254.77(311.24,202.09) | -5.54(-5.90,-5.18) |
| Republic of Moldova | male | 21092.42(24300.27,17610.53) | 24238.72(29657.38,19248.50) | 0.55(0.11,0.99) | 1042.49(1196.92,876.13) | 993.58(1222.04,789.40) | -0.06(-0.49,0.38) |
| Romania | male | 70128.32(82692.32,57185.64) | 96513.07(124106.20,72912.38) | 0.45(-0.02,0.91) | 528.08(621.00,434.84) | 664.22(857.56,503.81) | 0.14(-0.35,0.62) |
| Russian Federation | male | 127059.84(143458.11,111267.57) | 448800.05(558204.78,357977.04) | 4.28(3.29,5.28) | 171.11(193.06,151.34) | 491.96(610.71,392.87) | 3.73(2.77,4.70) |
| Rwanda | male | 10743.84(15285.64,6799.35) | 12759.66(19853.12,7869.12) | 0.28(-0.19,0.75) | 727.72(1033.72,464.54) | 399.77(613.55,249.96) | -3.05(-3.45,-2.65) |
| Saint Kitts and Nevis | male | 90.50(111.06,70.95) | 144.70(194.88,96.42) | 1.06(0.44,1.69) | 630.30(775.34,489.70) | 376.24(501.59,256.97) | -2.51(-3.00,-2.01) |
| Saint Lucia | male | 177.68(211.11,141.92) | 316.49(406.95,244.53) | 1.88(1.49,2.28) | 437.92(520.06,350.41) | 290.13(372.13,225.81) | -1.64(-2.05,-1.22) |
| Saint Vincent and the Grenadines | male | 108.54(133.67,83.11) | 223.11(285.19,170.53) | 2.45(2.01,2.89) | 330.16(406.82,252.34) | 311.70(394.32,239.58) | -0.44(-0.86,-0.02) |
| Samoa | male | 111.45(184.03,68.26) | 135.32(205.73,82.14) | 0.72(0.63,0.82) | 225.47(369.59,140.34) | 157.40(235.18,97.40) | -1.25(-1.35,-1.15) |
| San Marino | male | 33.03(42.02,25.03) | 48.59(71.53,30.69) | 1.36(1.27,1.45) | 228.42(288.19,174.02) | 191.22(289.03,118.31) | -0.64(-0.78,-0.51) |
| Sao Tome and Principe | male | 199.38(315.31,115.51) | 336.74(521.52,222.06) | 1.57(1.36,1.78) | 608.06(957.13,356.74) | 546.39(844.75,367.96) | -0.59(-0.81,-0.37) |
| Saudi Arabia | male | 2976.61(5013.80,1565.76) | 5436.46(8358.74,3208.90) | 1.42(1.10,1.74) | 80.34(137.01,42.41) | 45.53(71.08,27.50) | -2.21(-2.44,-1.98) |
| Senegal | male | 6808.70(11204.31,4084.13) | 12765.15(21138.49,7759.74) | 2.29(2.05,2.54) | 372.69(615.72,220.65) | 302.64(487.66,183.53) | -0.66(-0.90,-0.43) |
| Serbia | male | 14969.87(19255.22,11379.88) | 14401.87(19420.53,10440.30) | -0.51(-0.70,-0.32) | 258.46(330.27,199.20) | 220.87(298.13,159.67) | -0.88(-1.06,-0.69) |
| Seychelles | male | 85.39(120.75,56.87) | 210.44(293.70,137.82) | 2.94(2.71,3.17) | 318.39(450.19,211.33) | 317.67(438.43,212.67) | -0.39(-0.67,-0.11) |
| Sierra Leone | male | 4866.73(7450.11,2994.80) | 6254.72(9753.91,3798.63) | 0.49(0.17,0.81) | 466.04(716.71,283.88) | 284.99(438.59,172.99) | -2.17(-2.44,-1.90) |
| Singapore | male | 735.76(1008.87,509.31) | 800.73(1120.80,550.15) | 0.24(-0.11,0.59) | 59.83(83.38,41.55) | 18.94(26.14,13.16) | -4.06(-4.31,-3.82) |
| Slovakia | male | 16683.87(20424.26,12913.77) | 19083.41(26206.04,13795.49) | 1.02(0.75,1.28) | 625.07(763.54,484.90) | 485.31(668.13,352.28) | -0.40(-0.64,-0.15) |
| Slovenia | male | 6858.99(9443.04,4904.69) | 5656.24(7785.10,4042.32) | -0.82(-1.09,-0.56) | 626.04(856.22,449.26) | 326.66(450.39,231.80) | -2.43(-2.70,-2.16) |
| Solomon Islands | male | 345.41(546.55,208.40) | 747.26(1111.11,468.13) | 2.83(2.73,2.93) | 342.75(545.98,206.79) | 313.98(454.62,201.29) | -0.20(-0.29,-0.11) |
| Somalia | male | 7423.51(12122.27,3957.55) | 16096.94(26348.34,9059.22) | 2.62(2.53,2.71) | 496.90(788.71,276.81) | 429.19(687.36,242.89) | -0.50(-0.61,-0.40) |
| South Africa | male | 21730.56(29753.17,15719.95) | 30674.66(37150.58,24984.34) | 0.68(0.09,1.28) | 198.36(272.90,142.29) | 136.24(164.59,110.50) | -1.70(-2.24,-1.15) |
| South Sudan | male | 5481.39(10265.77,3215.02) | 7371.04(12998.67,4060.47) | 1.14(1.02,1.26) | 364.78(683.03,214.16) | 314.82(555.99,176.39) | -0.43(-0.49,-0.37) |
| Spain | male | 96244.20(112371.59,78902.05) | 63245.27(74676.02,51185.31) | -1.40(-1.54,-1.25) | 422.40(492.45,348.42) | 170.66(201.21,138.25) | -3.17(-3.30,-3.04) |
| Sri Lanka | male | 20164.00(27488.58,13459.26) | 26038.18(39830.33,16736.46) | -0.21(-0.97,0.56) | 300.85(410.76,203.76) | 213.50(323.22,138.27) | -2.20(-2.90,-1.50) |
| Sudan | male | 3312.35(5591.26,1584.54) | 5500.09(10588.18,2637.71) | 1.52(1.43,1.62) | 64.00(109.48,30.11) | 50.29(97.10,23.61) | -0.94(-0.99,-0.89) |
| Suriname | male | 612.82(752.47,471.08) | 1189.71(1564.42,858.07) | 2.14(1.95,2.34) | 438.15(531.36,341.11) | 387.28(504.80,281.75) | -0.80(-1.00,-0.60) |
| Sweden | male | 8194.05(8893.05,7549.23) | 8840.35(9930.53,7886.01) | 0.54(0.35,0.72) | 145.71(157.81,134.05) | 109.09(122.44,97.19) | -0.79(-0.96,-0.62) |
| Switzerland | male | 8611.10(9971.85,7062.32) | 8086.11(9614.48,6316.54) | -0.37(-0.47,-0.26) | 203.48(235.87,167.22) | 115.19(136.33,90.87) | -2.16(-2.31,-2.01) |
| Syrian Arab Republic | male | 1455.22(2236.33,854.41) | 2737.96(4521.89,1459.42) | 2.41(2.22,2.59) | 48.24(75.10,27.98) | 40.22(64.14,21.71) | -0.57(-0.76,-0.38) |
| Taiwan (Province of China) | male | 30169.32(37781.29,23668.59) | 43379.67(61564.09,28979.17) | 0.98(0.58,1.38) | 321.06(400.51,251.35) | 247.27(350.61,164.60) | -1.24(-1.56,-0.92) |
| Tajikistan | male | 5043.98(6359.90,3853.00) | 17558.38(23165.03,12715.21) | 4.19(3.91,4.46) | 347.24(437.70,265.91) | 528.89(692.92,392.73) | 1.33(1.16,1.50) |
| Thailand | male | 59194.05(80890.68,41044.36) | 143540.14(214515.86,92241.92) | 3.45(3.26,3.64) | 271.37(367.36,189.49) | 297.18(443.72,193.24) | 0.57(0.43,0.72) |
| Timor-Leste | male | 449.56(894.36,222.51) | 954.04(1673.86,426.41) | 2.47(2.16,2.79) | 204.59(406.31,99.15) | 212.81(375.18,97.30) | -0.10(-0.37,0.18) |
| Togo | male | 3122.48(4654.54,2031.30) | 7169.26(11098.64,4320.89) | 2.51(2.21,2.81) | 440.77(653.78,283.40) | 339.72(523.35,209.69) | -1.32(-1.64,-1.01) |
| Tokelau | male | 0.97(1.50,0.61) | 0.75(1.13,0.45) | -1.11(-1.41,-0.80) | 172.72(270.58,108.48) | 109.57(165.63,65.68) | -1.61(-1.70,-1.52) |
| Tonga | male | 74.52(107.62,47.28) | 89.97(132.02,57.03) | 0.74(0.49,0.98) | 249.34(356.68,158.55) | 223.98(323.31,142.82) | -0.35(-0.58,-0.12) |
| Trinidad and Tobago | male | 1419.34(1697.14,1127.70) | 1864.10(2659.14,1264.37) | 0.95(0.74,1.16) | 318.33(378.41,251.24) | 203.52(287.40,138.36) | -1.60(-1.86,-1.34) |
| Tunisia | male | 1096.90(1842.46,613.03) | 2011.68(3490.88,1077.76) | 2.13(2.11,2.15) | 40.19(66.92,22.54) | 31.47(54.40,17.08) | -0.79(-0.83,-0.75) |
| Turkey | male | 6931.14(11591.90,3852.49) | 11088.24(17091.57,6695.20) | 1.60(1.35,1.85) | 35.71(58.53,20.10) | 25.29(38.42,15.37) | -1.13(-1.35,-0.91) |
| Turkmenistan | male | 5092.13(6238.56,4028.31) | 25085.48(33020.93,18216.76) | 5.94(5.61,6.28) | 505.81(613.72,401.99) | 1043.21(1360.04,765.88) | 2.73(2.41,3.04) |
| Tuvalu | male | 9.54(15.46,5.03) | 10.62(17.03,5.92) | 0.52(0.26,0.78) | 281.64(449.44,152.53) | 193.35(309.21,109.24) | -1.21(-1.41,-1.01) |
| Uganda | male | 15117.23(23370.28,9297.31) | 27405.30(40525.77,17489.82) | 1.63(1.41,1.84) | 425.15(655.97,263.44) | 356.03(523.03,225.22) | -1.02(-1.24,-0.79) |
| Ukraine | male | 65205.62(74772.29,57195.34) | 226511.43(287872.55,174051.05) | 4.10(3.25,4.96) | 226.93(259.62,200.47) | 819.21(1045.83,628.47) | 4.33(3.45,5.22) |
| United Arab Emirates | male | 150.00(267.81,83.69) | 1391.59(2398.64,685.87) | 7.58(7.22,7.95) | 40.25(72.30,21.32) | 30.67(52.54,14.99) | -1.18(-1.56,-0.80) |
| United Kingdom | male | 48956.41(52773.42,44757.25) | 109010.16(117981.88,100416.65) | 2.80(2.23,3.37) | 141.72(152.30,129.88) | 237.43(256.57,219.50) | 1.71(1.14,2.29) |
| United Republic of Tanzania | male | 24052.82(35272.68,15881.02) | 42355.79(64420.52,26289.11) | 1.95(1.73,2.16) | 399.75(581.85,266.51) | 301.59(452.42,186.53) | -0.97(-1.23,-0.70) |
| United States of America | male | 228872.62(260751.59,200418.75) | 336618.50(398691.83,282258.74) | 1.79(1.60,1.98) | 172.06(196.05,149.89) | 144.74(170.62,121.90) | -0.28(-0.42,-0.14) |
| United States Virgin Islands | male | 189.46(242.43,138.64) | 316.19(411.96,227.01) | 2.27(1.94,2.61) | 397.14(506.58,293.57) | 415.93(538.66,299.60) | 0.51(0.18,0.83) |
| Uruguay | male | 3200.12(4247.12,2306.95) | 2790.63(3699.05,1971.18) | -0.87(-1.04,-0.70) | 188.19(248.77,135.46) | 131.33(172.84,94.09) | -1.60(-1.75,-1.45) |
| Uzbekistan | male | 25363.18(31068.38,19881.86) | 114324.35(145998.56,86620.76) | 4.71(4.27,5.15) | 442.52(535.66,350.43) | 843.28(1060.41,654.61) | 1.73(1.24,2.22) |
| Vanuatu | male | 130.10(222.00,69.85) | 259.58(423.88,134.18) | 2.32(2.18,2.47) | 285.76(483.77,155.60) | 240.97(393.55,125.80) | -0.77(-0.90,-0.64) |
| Venezuela (Bolivarian Republic of) | male | 20061.64(23772.28,16136.50) | 46905.11(66261.24,32287.10) | 2.95(2.65,3.25) | 368.35(436.33,297.59) | 313.65(442.07,218.11) | -0.72(-1.00,-0.43) |
| Viet Nam | male | 65522.50(100386.34,39842.35) | 139720.51(205127.39,89414.89) | 2.49(1.91,3.07) | 350.26(536.55,211.61) | 279.33(398.62,180.68) | -1.11(-1.65,-0.57) |
| Yemen | male | 1774.75(3036.35,849.76) | 3786.11(6376.36,1940.71) | 2.55(2.48,2.62) | 65.26(114.43,31.14) | 51.29(88.41,26.30) | -0.93(-0.99,-0.88) |
| Zambia | male | 10539.25(16459.81,6665.97) | 24911.55(36043.83,16181.21) | 2.60(2.38,2.83) | 625.77(976.47,398.74) | 608.40(866.51,402.34) | -0.39(-0.66,-0.13) |
| Zimbabwe | male | 7084.85(10140.70,4617.57) | 11331.69(18122.44,6672.66) | 1.22(0.99,1.45) | 309.32(439.91,202.97) | 307.93(485.35,185.53) | -0.10(-0.40,0.19) |
| Afghanistan | female | 1459.94(2712.77,592.96) | 2467.03(4819.29,965.50) | 1.33(1.14,1.53) | 39.02(69.71,17.08) | 32.75(60.29,14.41) | -0.89(-1.08,-0.69) |
| Albania | female | 870.79(1056.22,701.07) | 915.48(1289.20,626.41) | 0.29(0.00,0.57) | 77.17(93.90,61.43) | 42.81(59.15,29.95) | -1.89(-2.18,-1.59) |
| Algeria | female | 1163.35(1847.13,684.04) | 2057.42(3182.32,1273.21) | 1.79(1.60,1.99) | 17.96(27.96,10.69) | 12.00(18.45,7.54) | -1.51(-1.65,-1.36) |
| American Samoa | female | 4.50(6.57,2.99) | 8.47(12.51,5.23) | 2.09(1.88,2.31) | 34.39(50.87,22.27) | 32.00(47.06,20.34) | -0.25(-0.54,0.05) |
| Andorra | female | 20.69(32.98,12.32) | 43.08(63.03,28.66) | 2.30(2.14,2.45) | 73.90(117.50,43.87) | 65.14(95.03,43.65) | -0.51(-0.64,-0.39) |
| Angola | female | 3105.24(5309.90,1666.96) | 8040.27(12521.25,4858.41) | 3.36(3.23,3.49) | 129.50(222.04,69.36) | 107.59(166.96,64.40) | -0.68(-0.76,-0.60) |
| Antigua and Barbuda | female | 19.54(25.43,14.70) | 38.66(51.47,27.67) | 2.11(1.82,2.41) | 71.77(94.50,53.36) | 68.97(91.14,49.63) | -0.53(-0.84,-0.22) |
| Argentina | female | 10643.06(14033.55,7635.08) | 12377.87(16700.80,8571.11) | 0.76(0.37,1.16) | 61.59(81.43,44.19) | 44.26(60.06,30.39) | -0.90(-1.27,-0.53) |
| Armenia | female | 1147.44(1417.38,881.96) | 2880.24(3737.48,2159.86) | 3.75(3.26,4.23) | 72.11(89.50,55.80) | 121.29(156.14,91.52) | 2.42(1.91,2.94) |
| Australia | female | 1738.34(2371.16,1207.18) | 2572.20(3583.74,1755.83) | 2.04(1.68,2.40) | 18.09(24.74,12.46) | 14.36(19.98,9.62) | -0.17(-0.54,0.21) |
| Austria | female | 7805.52(9247.95,6279.68) | 5595.14(6776.44,4410.37) | -1.33(-1.40,-1.25) | 144.84(171.65,117.07) | 74.63(90.38,59.09) | -2.54(-2.63,-2.45) |
| Azerbaijan | female | 5459.17(6809.19,4177.53) | 11244.01(15984.53,7586.61) | 1.65(1.28,2.02) | 179.97(225.66,137.04) | 215.49(307.37,142.48) | -0.15(-0.64,0.35) |
| Bahamas | female | 116.50(145.50,88.68) | 175.61(236.87,120.76) | 1.28(1.02,1.54) | 124.51(156.85,93.94) | 76.84(103.16,53.80) | -1.76(-2.03,-1.49) |
| Bahrain | female | 23.26(36.69,13.57) | 54.64(88.74,31.43) | 2.70(2.46,2.94) | 27.59(43.69,15.88) | 13.81(21.81,7.94) | -2.78(-3.03,-2.54) |
| Bangladesh | female | 31287.51(48401.43,18412.56) | 47267.32(73992.68,29299.08) | 1.56(1.35,1.77) | 122.97(189.92,71.82) | 68.65(106.87,42.52) | -2.06(-2.27,-1.85) |
| Barbados | female | 74.86(96.70,56.71) | 102.00(137.73,70.59) | 1.19(0.96,1.42) | 52.58(67.23,39.48) | 40.43(54.36,28.15) | -0.90(-1.07,-0.74) |
| Belarus | female | 3220.21(4126.79,2420.72) | 10816.59(15023.05,7236.65) | 5.10(3.89,6.33) | 43.40(55.37,32.89) | 137.41(192.27,92.91) | 4.82(3.49,6.18) |
| Belgium | female | 7802.87(9102.32,6433.87) | 7977.30(9640.40,6379.01) | -0.08(-0.18,0.01) | 110.14(127.67,90.73) | 85.01(102.18,67.92) | -1.14(-1.26,-1.01) |
| Belize | female | 56.15(71.31,42.41) | 174.15(227.89,123.02) | 4.03(3.88,4.18) | 119.52(151.67,89.63) | 111.29(144.19,79.39) | -0.28(-0.52,-0.04) |
| Benin | female | 1684.01(2508.99,1095.48) | 3577.91(5422.30,2273.74) | 2.48(2.35,2.60) | 149.35(226.71,98.23) | 118.97(178.60,76.51) | -0.92(-1.01,-0.82) |
| Bermuda | female | 24.53(31.02,18.50) | 12.90(18.05,8.84) | -3.00(-3.48,-2.52) | 69.76(88.11,52.59) | 20.32(28.23,14.00) | -5.06(-5.56,-4.56) |
| Bhutan | female | 157.73(243.27,88.38) | 250.30(382.09,154.68) | 1.47(1.28,1.66) | 104.60(162.05,59.68) | 83.82(127.84,52.49) | -0.94(-1.10,-0.78) |
| Bolivia (Plurinational State of) | female | 4577.46(7165.53,2476.91) | 10150.15(14749.58,6379.55) | 2.72(2.67,2.78) | 246.06(382.97,135.25) | 211.26(306.22,134.66) | -0.66(-0.72,-0.60) |
| Bosnia and Herzegovina | female | 2313.67(2848.97,1787.85) | 1745.50(2467.07,1218.76) | -1.69(-2.02,-1.36) | 93.89(114.38,73.60) | 56.79(81.43,39.94) | -2.40(-2.79,-2.00) |
| Botswana | female | 361.35(666.83,179.50) | 773.10(1259.35,422.14) | 2.62(2.43,2.82) | 99.90(183.92,49.81) | 82.67(134.86,45.95) | -0.59(-0.83,-0.36) |
| Brazil | female | 42167.58(47964.84,36608.46) | 70812.58(82356.86,60415.93) | 1.91(1.78,2.03) | 78.69(89.68,68.51) | 53.98(62.60,46.16) | -1.22(-1.34,-1.09) |
| Brunei Darussalam | female | 38.56(57.78,23.36) | 76.62(110.31,50.30) | 1.83(1.48,2.18) | 61.15(92.96,37.18) | 39.69(57.12,26.53) | -2.07(-2.41,-1.73) |
| Bulgaria | female | 5580.45(6648.22,4516.72) | 6854.34(9036.69,5059.09) | 0.64(0.53,0.76) | 86.48(102.48,71.04) | 112.14(150.80,82.47) | 0.87(0.75,0.98) |
| Burkina Faso | female | 3758.05(5416.66,2491.11) | 6014.83(9928.90,2240.28) | 1.17(0.81,1.53) | 151.35(217.08,100.35) | 105.14(175.86,36.43) | -1.76(-2.09,-1.44) |
| Burundi | female | 2690.39(4346.50,1486.25) | 3139.25(5347.19,1436.50) | -0.17(-0.56,0.22) | 194.66(311.43,109.43) | 124.28(206.63,55.77) | -2.20(-2.49,-1.92) |
| Cabo Verde | female | 95.05(145.08,61.36) | 158.05(228.40,106.45) | 1.38(1.09,1.66) | 76.85(116.26,49.17) | 64.04(93.21,42.35) | -0.96(-1.20,-0.71) |
| Cambodia | female | 9223.85(13137.49,5965.48) | 18914.54(28377.25,12401.56) | 2.35(2.20,2.50) | 301.95(429.36,197.90) | 253.63(378.14,167.00) | -0.80(-0.94,-0.66) |
| Cameroon | female | 3903.03(5576.20,2566.76) | 7286.99(12181.62,3653.12) | 2.10(1.98,2.23) | 149.71(211.82,99.69) | 99.73(166.53,49.97) | -1.48(-1.60,-1.35) |
| Canada | female | 6213.23(8363.96,4360.83) | 11012.17(15008.03,7702.32) | 2.26(2.16,2.37) | 37.61(51.20,25.96) | 36.06(48.25,25.69) | 0.08(-0.05,0.20) |
| Central African Republic | female | 824.72(1334.44,466.21) | 1683.59(2922.80,903.74) | 2.34(2.16,2.52) | 108.48(174.69,60.66) | 116.78(202.43,64.21) | 0.10(-0.14,0.35) |
| Chad | female | 2336.72(3639.13,1369.48) | 4243.25(6200.32,2680.64) | 2.00(1.93,2.07) | 149.33(234.23,87.78) | 136.92(203.19,85.97) | -0.32(-0.37,-0.26) |
| Chile | female | 7826.26(10415.72,5470.07) | 9048.39(12372.87,6302.23) | 0.77(0.49,1.04) | 137.33(182.75,95.62) | 71.24(96.21,50.02) | -2.04(-2.28,-1.79) |
| China | female | 161447.71(209716.49,120330.71) | 130374.76(170281.56,96780.13) | -1.06(-1.24,-0.88) | 35.04(45.42,26.30) | 12.26(15.90,9.14) | -3.89(-4.06,-3.72) |
| Colombia | female | 6231.16(7629.61,4851.30) | 11316.38(15768.67,7726.97) | 1.72(1.58,1.86) | 63.03(77.47,49.16) | 39.63(55.22,27.10) | -2.01(-2.16,-1.86) |
| Comoros | female | 142.65(245.26,59.77) | 291.64(465.45,141.09) | 2.25(2.01,2.50) | 118.58(202.61,50.83) | 106.03(169.47,49.83) | -0.58(-0.80,-0.35) |
| Congo | female | 765.07(1209.85,450.42) | 1781.24(2937.76,1010.06) | 2.99(2.74,3.24) | 114.41(179.50,67.39) | 109.60(181.09,62.20) | -0.09(-0.43,0.25) |
| Cook Islands | female | 1.03(1.60,0.61) | 1.41(2.18,0.84) | 1.39(1.18,1.59) | 15.98(24.31,9.39) | 11.37(17.45,6.80) | -0.89(-1.13,-0.66) |
| Costa Rica | female | 910.49(1119.07,704.13) | 2745.98(3801.52,1908.23) | 3.35(3.04,3.65) | 95.60(118.20,73.78) | 98.27(135.92,68.41) | -0.44(-0.72,-0.15) |
| Côte d'Ivoire | female | 2834.43(4320.85,1769.56) | 6051.97(9633.49,3356.83) | 2.73(2.57,2.90) | 123.75(186.41,79.16) | 97.77(160.58,55.71) | -0.61(-0.71,-0.50) |
| Croatia | female | 6474.00(7772.26,5165.40) | 2933.64(3939.16,2157.80) | -3.08(-3.28,-2.89) | 183.88(219.98,149.25) | 73.19(100.06,54.22) | -3.56(-3.77,-3.35) |
| Cuba | female | 3417.42(4307.66,2599.26) | 4607.16(6279.15,3217.84) | 0.87(0.60,1.15) | 65.65(82.82,49.71) | 48.84(66.17,33.86) | -1.20(-1.50,-0.91) |
| Cyprus | female | 338.37(499.74,223.83) | 378.35(481.68,282.70) | -0.10(-0.27,0.07) | 81.78(119.94,53.40) | 39.43(50.21,29.20) | -3.02(-3.21,-2.82) |
| Czechia | female | 7079.47(8239.76,5925.21) | 8889.03(11582.28,6778.47) | 1.12(0.97,1.28) | 99.29(114.94,83.19) | 102.20(134.08,77.66) | 0.36(0.22,0.50) |
| Democratic People's Republic of Korea | female | 4640.86(7291.58,2496.37) | 6424.17(10324.98,3444.82) | 1.04(0.91,1.18) | 44.24(68.73,24.23) | 35.20(55.79,18.91) | -0.79(-0.94,-0.63) |
| Democratic Republic of the Congo | female | 8742.42(13180.97,5602.72) | 15437.59(23708.68,9493.93) | 1.78(1.65,1.92) | 85.45(128.51,54.75) | 67.10(102.71,41.11) | -0.95(-1.04,-0.86) |
| Denmark | female | 3235.42(3848.54,2590.63) | 3329.84(4167.88,2573.54) | -0.65(-1.21,-0.09) | 98.38(117.40,77.62) | 72.54(90.13,56.12) | -1.86(-2.41,-1.32) |
| Djibouti | female | 95.75(156.27,55.23) | 295.16(544.52,142.05) | 3.71(3.58,3.85) | 117.80(193.07,69.01) | 92.59(169.05,46.09) | -1.04(-1.14,-0.93) |
| Dominica | female | 36.62(47.63,26.86) | 28.70(39.43,19.85) | -1.04(-1.29,-0.78) | 102.05(132.74,74.96) | 67.39(91.84,46.90) | -1.56(-1.75,-1.38) |
| Dominican Republic | female | 4435.40(5728.42,3226.75) | 8162.92(11652.77,5240.28) | 1.69(1.30,2.07) | 211.62(273.84,153.10) | 164.30(234.61,106.24) | -1.24(-1.68,-0.79) |
| Ecuador | female | 3676.82(4821.35,2689.06) | 10852.23(15070.63,7488.69) | 4.24(3.90,4.57) | 124.71(164.78,91.43) | 135.60(188.27,93.70) | 0.75(0.42,1.07) |
| Egypt | female | 14275.00(22001.32,8952.17) | 25447.10(44490.04,13492.89) | 2.39(2.17,2.61) | 102.48(157.99,65.40) | 95.29(165.53,51.50) | 0.22(0.02,0.42) |
| El Salvador | female | 2313.49(2928.63,1720.70) | 3898.19(5609.32,2588.62) | 2.08(1.83,2.33) | 138.20(175.78,102.19) | 116.78(169.43,76.82) | -0.30(-0.55,-0.06) |
| Equatorial Guinea | female | 106.02(180.58,53.46) | 191.81(334.70,97.21) | 1.85(1.72,1.99) | 82.80(143.36,41.23) | 58.63(102.85,30.01) | -1.42(-1.61,-1.22) |
| Eritrea | female | 1087.63(1918.84,501.37) | 3103.71(4878.99,1845.37) | 3.89(3.76,4.03) | 162.80(287.38,79.11) | 179.65(283.01,108.23) | 0.45(0.37,0.54) |
| Estonia | female | 572.95(742.52,420.20) | 1059.21(1447.65,739.62) | 1.56(0.58,2.55) | 50.82(65.28,37.14) | 104.28(145.63,73.07) | 1.83(0.79,2.88) |
| Eswatini | female | 170.96(269.05,108.62) | 314.97(528.44,156.31) | 2.35(1.86,2.83) | 88.58(140.65,55.90) | 81.24(135.77,40.95) | 0.11(-0.32,0.53) |
| Ethiopia | female | 22101.82(34529.04,12275.61) | 23936.14(32263.32,17588.15) | -0.40(-0.71,-0.10) | 200.78(311.34,114.80) | 109.97(146.73,80.21) | -2.58(-2.79,-2.38) |
| Fiji | female | 79.58(115.57,48.85) | 131.48(199.09,80.69) | 1.97(1.85,2.08) | 35.16(51.48,21.88) | 30.51(45.49,18.96) | -0.35(-0.44,-0.27) |
| Finland | female | 2226.55(2702.88,1772.65) | 3620.95(4498.39,2699.17) | 2.36(1.74,2.99) | 64.84(78.46,51.39) | 82.96(102.69,62.73) | 1.41(0.83,1.99) |
| France | female | 48306.40(57300.83,38812.00) | 31304.77(38597.11,24238.58) | -1.78(-1.91,-1.65) | 132.16(157.80,106.22) | 58.15(71.31,45.20) | -3.18(-3.33,-3.03) |
| Gabon | female | 303.76(483.90,179.64) | 483.99(776.14,287.71) | 1.39(1.09,1.69) | 94.70(150.85,55.58) | 78.23(128.41,45.14) | -0.79(-1.16,-0.42) |
| Gambia | female | 232.74(377.16,128.62) | 623.81(1016.83,353.48) | 3.15(2.78,3.52) | 124.07(202.69,68.05) | 114.13(185.46,64.16) | -0.60(-0.89,-0.31) |
| Georgia | female | 4916.18(6069.21,3806.79) | 3121.80(4108.16,2277.84) | -1.77(-2.02,-1.51) | 134.02(165.76,105.12) | 97.82(127.33,71.80) | -1.27(-4.42,1.99) |
| Germany | female | 92185.59(107817.67,74822.93) | 68800.63(84038.24,54414.53) | -1.59(-1.77,-1.40) | 158.39(184.45,129.71) | 92.75(111.81,73.65) | -2.49(-2.70,-2.28) |
| Ghana | female | 7103.22(11259.61,4271.48) | 10871.15(16144.48,6585.05) | 1.08(0.84,1.33) | 187.17(294.93,112.45) | 104.84(156.46,61.67) | -2.36(-2.58,-2.15) |
| Greece | female | 4379.37(5379.40,3432.99) | 3067.04(3792.52,2364.95) | -1.59(-1.83,-1.36) | 55.60(67.53,44.17) | 30.26(36.97,23.81) | -2.39(-2.70,-2.08) |
| Greenland | female | 25.44(37.06,16.22) | 30.34(45.75,19.42) | 0.85(0.59,1.10) | 114.69(168.54,73.50) | 88.70(130.49,57.80) | -0.79(-0.92,-0.66) |
| Grenada | female | 37.67(48.01,28.35) | 37.74(49.03,27.61) | 0.17(-0.15,0.49) | 105.22(134.62,77.71) | 64.07(83.13,47.12) | -1.72(-2.13,-1.32) |
| Guam | female | 18.61(26.84,12.36) | 29.83(44.65,19.24) | 1.44(1.24,1.63) | 44.73(64.80,29.94) | 31.24(46.33,20.20) | -1.41(-1.78,-1.03) |
| Guatemala | female | 6727.84(8759.72,4891.89) | 15451.59(21038.75,10752.63) | 2.87(2.50,3.25) | 291.86(376.49,214.70) | 234.75(320.26,163.37) | -0.80(-1.16,-0.45) |
| Guinea | female | 2805.18(4491.27,1635.79) | 3878.87(6520.34,2038.93) | 0.79(0.63,0.94) | 156.73(252.17,92.38) | 123.82(209.71,65.17) | -0.87(-0.94,-0.81) |
| Guinea-Bissau | female | 484.60(769.29,279.01) | 896.40(1328.23,560.12) | 2.03(1.94,2.12) | 195.65(301.44,112.36) | 185.10(270.50,116.12) | -0.26(-0.33,-0.18) |
| Guyana | female | 447.94(596.35,323.73) | 557.01(781.73,373.10) | 0.97(0.71,1.24) | 201.22(264.48,145.33) | 150.40(210.60,102.60) | -0.77(-1.03,-0.52) |
| Haiti | female | 4543.66(7077.23,2238.39) | 6973.29(11598.17,3198.11) | 1.57(1.44,1.70) | 236.54(356.97,121.44) | 159.46(264.52,73.49) | -1.25(-1.35,-1.16) |
| Honduras | female | 3564.92(4816.06,2488.36) | 11748.57(18407.06,6357.01) | 4.27(4.11,4.44) | 287.95(386.66,203.75) | 336.88(524.76,186.38) | 0.58(0.36,0.80) |
| Hungary | female | 22787.17(26652.65,18886.94) | 11372.83(14543.52,8631.01) | -3.49(-3.88,-3.10) | 321.59(378.75,265.30) | 133.44(173.49,100.95) | -4.29(-4.71,-3.86) |
| Iceland | female | 50.65(63.54,38.75) | 47.70(61.42,35.48) | -0.52(-0.66,-0.37) | 39.23(49.40,29.74) | 20.65(26.58,15.42) | -2.59(-2.76,-2.42) |
| India | female | 216796.59(307363.65,158382.30) | 409993.85(569470.30,293797.18) | 2.12(1.88,2.36) | 82.71(117.55,60.19) | 65.28(90.29,46.87) | -0.98(-1.23,-0.74) |
| Indonesia | female | 95995.85(120718.13,74408.49) | 137412.83(183524.49,102631.34) | 1.26(1.13,1.38) | 160.75(202.71,123.92) | 114.17(149.36,85.07) | -1.08(-1.19,-0.96) |
| Iran (Islamic Republic of) | female | 1661.04(2245.92,1223.67) | 3028.50(3823.20,2385.21) | 2.66(2.35,2.97) | 12.66(17.58,9.00) | 8.00(10.18,6.31) | -1.14(-1.41,-0.88) |
| Iraq | female | 677.53(1063.96,396.67) | 1433.23(2250.06,839.74) | 2.62(2.47,2.77) | 15.93(25.42,9.29) | 10.92(17.24,6.49) | -1.29(-1.45,-1.13) |
| Ireland | female | 800.87(985.10,633.13) | 1567.08(1931.71,1234.91) | 2.33(1.84,2.82) | 41.59(50.98,32.82) | 46.15(56.80,36.22) | 0.23(-0.29,0.75) |
| Israel | female | 1124.43(1452.12,827.50) | 1825.85(2369.15,1370.33) | 1.09(0.81,1.38) | 44.27(56.72,33.09) | 31.37(40.59,23.74) | -1.68(-1.92,-1.44) |
| Italy | female | 28746.51(33309.63,24661.63) | 15595.94(18827.49,12728.90) | -2.50(-2.74,-2.25) | 64.59(74.13,55.98) | 24.22(28.84,20.06) | -3.78(-4.07,-3.50) |
| Jamaica | female | 342.68(434.99,258.18) | 495.31(696.48,338.43) | 0.99(0.63,1.36) | 38.66(49.07,28.68) | 32.43(45.38,22.00) | -0.90(-1.28,-0.53) |
| Japan | female | 25798.54(30067.43,22039.72) | 21215.36(25380.48,17167.72) | -0.44(-0.62,-0.25) | 27.66(32.09,23.68) | 13.90(16.43,11.58) | -2.08(-2.30,-1.86) |
| Jordan | female | 141.95(227.05,84.04) | 348.87(556.68,205.29) | 2.78(2.57,3.00) | 20.49(32.15,12.04) | 10.80(17.11,6.27) | -2.45(-2.80,-2.09) |
| Kazakhstan | female | 9159.84(11149.78,7271.28) | 29362.10(37368.84,22512.87) | 4.71(4.18,5.25) | 116.96(142.07,93.07) | 277.27(351.71,213.04) | 3.65(3.00,4.30) |
| Kenya | female | 10444.69(16983.10,6152.88) | 26851.57(44071.19,14803.28) | 3.60(3.45,3.74) | 227.59(362.16,135.56) | 208.87(339.66,116.56) | 0.05(-0.08,0.19) |
| Kiribati | female | 28.81(44.61,15.90) | 37.72(65.10,17.94) | 1.02(0.85,1.19) | 124.61(193.15,71.68) | 84.18(139.32,43.81) | -1.38(-1.50,-1.27) |
| Kuwait | female | 22.25(33.15,13.93) | 73.55(114.97,44.84) | 4.90(4.31,5.49) | 8.37(13.26,5.12) | 6.00(9.57,3.62) | -0.26(-1.17,0.66) |
| Kyrgyzstan | female | 4017.75(4934.71,3115.16) | 7931.66(10059.46,6145.88) | 2.16(1.75,2.58) | 223.95(275.45,174.66) | 272.16(344.57,211.50) | 0.50(-0.07,1.08) |
| Lao People's Democratic Republic | female | 1481.64(2385.04,748.07) | 2035.20(3021.98,1271.16) | 0.77(0.48,1.05) | 118.45(188.38,62.51) | 78.55(117.54,48.34) | -1.72(-1.93,-1.51) |
| Latvia | female | 1110.59(1416.59,809.71) | 1746.08(2514.37,1177.91) | 1.41(0.70,2.13) | 55.73(70.81,41.03) | 112.52(163.15,74.58) | 2.31(1.56,3.07) |
| Lebanon | female | 182.22(287.63,99.06) | 278.17(486.07,150.87) | 1.42(1.25,1.59) | 15.30(23.95,8.50) | 9.81(17.18,5.30) | -1.57(-1.75,-1.40) |
| Lesotho | female | 448.01(795.26,210.12) | 758.55(1315.86,345.24) | 2.52(2.20,2.85) | 77.32(136.84,36.20) | 93.36(158.94,42.25) | 1.46(1.10,1.81) |
| Liberia | female | 1052.17(1613.22,666.67) | 1702.53(2581.89,1040.80) | 1.30(1.14,1.45) | 193.08(298.05,122.05) | 142.69(213.47,87.84) | -1.39(-1.51,-1.26) |
| Libya | female | 178.75(304.18,96.94) | 380.54(601.75,225.38) | 2.76(2.37,3.16) | 19.59(34.28,10.42) | 14.17(22.66,8.54) | -1.03(-1.30,-0.76) |
| Lithuania | female | 1470.30(1852.55,1099.58) | 3735.29(4977.57,2718.42) | 4.04(2.98,5.12) | 60.11(75.78,45.52) | 166.37(224.29,120.38) | 4.38(3.34,5.43) |
| Luxembourg | female | 374.84(452.02,298.29) | 318.85(401.35,245.35) | -0.64(-0.74,-0.53) | 142.46(171.03,113.30) | 70.61(88.35,53.95) | -2.45(-2.56,-2.34) |
| Madagascar | female | 4190.05(6196.19,2673.57) | 8825.39(13670.57,5227.40) | 2.56(2.43,2.69) | 146.17(215.87,93.69) | 131.78(201.61,80.43) | -0.43(-0.52,-0.34) |
| Malawi | female | 4204.64(6110.72,2627.44) | 4966.46(7497.42,2948.68) | 0.20(-0.07,0.46) | 186.41(274.58,117.82) | 115.64(175.66,68.72) | -1.93(-2.10,-1.76) |
| Malaysia | female | 1717.58(2466.33,1128.18) | 5306.26(8359.37,3190.01) | 3.40(3.05,3.74) | 33.68(49.19,21.70) | 38.09(58.85,23.02) | -0.24(-0.62,0.14) |
| Maldives | female | 32.60(56.08,14.85) | 38.96(55.04,26.76) | 0.21(-0.11,0.52) | 69.35(117.27,33.73) | 26.25(37.55,17.92) | -3.79(-4.05,-3.53) |
| Mali | female | 4539.42(7139.59,2747.12) | 5788.27(9611.41,3112.55) | 0.47(0.28,0.67) | 193.75(299.92,119.80) | 119.49(196.89,64.63) | -2.02(-2.23,-1.81) |
| Malta | female | 134.70(167.37,105.69) | 125.45(160.23,95.55) | -0.34(-0.46,-0.22) | 59.11(73.47,46.62) | 36.26(45.46,27.76) | -1.79(-1.86,-1.72) |
| Marshall Islands | female | 7.44(11.82,4.34) | 13.67(26.22,5.77) | 1.94(1.85,2.02) | 77.91(120.49,45.30) | 64.30(120.49,28.15) | -0.94(-1.06,-0.82) |
| Mauritania | female | 1019.46(1527.41,665.13) | 1090.11(1655.68,645.04) | 0.11(-0.01,0.23) | 184.23(277.22,118.07) | 95.01(145.74,55.22) | -2.42(-2.53,-2.32) |
| Mauritius | female | 218.51(293.99,155.04) | 304.18(435.59,201.96) | 0.63(0.32,0.94) | 50.16(67.95,35.56) | 33.11(47.82,22.33) | -1.97(-2.24,-1.70) |
| Mexico | female | 55517.98(62753.49,48510.52) | 99945.37(127448.27,78497.30) | 1.62(1.45,1.79) | 220.89(249.73,192.79) | 154.23(196.68,121.36) | -1.67(-1.84,-1.50) |
| Micronesia (Federated States of) | female | 22.77(37.12,12.57) | 25.33(47.42,10.95) | 0.30(0.07,0.53) | 87.29(140.49,49.72) | 59.49(108.22,26.87) | -1.38(-1.61,-1.14) |
| Monaco | female | 18.19(25.46,12.43) | 23.71(32.40,15.92) | 1.18(1.02,1.34) | 65.23(90.81,45.85) | 66.93(90.99,44.70) | 0.33(0.18,0.48) |
| Mongolia | female | 2976.25(4001.70,2111.62) | 7587.13(10408.69,5245.46) | 3.40(3.26,3.55) | 491.98(658.54,348.92) | 519.95(705.46,361.14) | 0.34(-0.01,0.68) |
| Montenegro | female | 100.11(137.44,73.81) | 137.76(179.47,102.95) | 0.79(0.48,1.10) | 29.06(39.62,21.57) | 29.49(39.00,22.17) | -0.24(-0.51,0.03) |
| Morocco | female | 1405.81(2247.66,803.38) | 2513.04(4180.86,1417.53) | 2.09(1.98,2.20) | 19.25(30.89,10.98) | 15.08(24.28,8.85) | -0.71(-0.82,-0.60) |
| Mozambique | female | 2732.96(4339.00,1585.08) | 4166.59(7408.49,1399.03) | 1.60(1.38,1.83) | 80.63(128.61,47.70) | 63.97(112.72,20.53) | -0.67(-0.83,-0.51) |
| Myanmar | female | 12250.37(19209.40,6999.46) | 29275.50(43857.92,18730.67) | 2.99(2.71,3.27) | 84.25(131.51,48.78) | 98.97(147.13,64.26) | 0.48(0.22,0.75) |
| Namibia | female | 341.35(560.87,179.53) | 580.66(1050.49,269.19) | 1.34(1.09,1.60) | 82.43(135.22,43.36) | 66.14(118.21,31.55) | -1.13(-1.35,-0.91) |
| Nauru | female | 1.69(2.97,0.77) | 1.85(3.38,0.81) | 0.37(0.25,0.50) | 70.86(121.84,33.84) | 59.15(104.54,27.31) | -0.55(-0.67,-0.44) |
| Nepal | female | 13589.79(19314.39,8856.03) | 24758.73(36342.25,16366.10) | 2.53(2.20,2.87) | 241.08(340.68,158.43) | 192.52(283.04,126.77) | -0.45(-0.73,-0.16) |
| Netherlands | female | 5010.18(5986.50,4047.50) | 5155.35(6416.42,3966.16) | -0.60(-1.01,-0.19) | 50.69(60.64,41.15) | 33.64(41.47,26.00) | -2.14(-2.54,-1.73) |
| New Zealand | female | 275.11(331.39,228.34) | 322.59(402.22,256.74) | 0.75(0.63,0.87) | 14.28(17.15,11.76) | 8.94(11.12,7.17) | -1.50(-1.61,-1.39) |
| Nicaragua | female | 966.72(1238.00,723.48) | 3489.91(4595.21,2498.21) | 4.73(4.54,4.91) | 104.55(133.36,78.11) | 135.54(177.88,97.45) | 1.10(0.89,1.31) |
| Niger | female | 2399.66(3707.35,1454.59) | 5866.77(9419.87,3192.57) | 2.70(2.53,2.88) | 150.11(230.90,92.75) | 124.35(195.01,67.39) | -1.11(-1.29,-0.92) |
| Nigeria | female | 32411.55(49689.75,20873.10) | 64949.52(105573.82,38002.68) | 2.79(2.54,3.04) | 148.60(228.06,96.13) | 122.14(195.08,73.84) | -0.36(-0.50,-0.21) |
| Niue | female | 0.56(0.90,0.33) | 0.44(0.69,0.24) | -1.30(-1.49,-1.11) | 50.69(81.65,29.56) | 39.66(63.16,21.72) | -1.05(-1.14,-0.95) |
| North Macedonia | female | 510.49(696.73,396.18) | 847.38(1151.44,595.93) | 1.61(1.46,1.75) | 49.52(66.72,38.73) | 51.89(70.91,36.74) | 0.03(-0.09,0.15) |
| Northern Mariana Islands | female | 9.14(13.86,5.77) | 14.99(22.38,9.35) | 1.61(1.31,1.92) | 88.93(131.22,56.53) | 51.39(74.27,33.59) | -2.08(-2.30,-1.87) |
| Norway | female | 1126.51(1289.74,974.92) | 1061.92(1264.53,896.30) | -0.18(-0.26,-0.10) | 39.77(45.45,34.75) | 25.64(30.33,21.71) | -1.57(-1.69,-1.44) |
| Oman | female | 83.22(137.68,46.42) | 138.32(228.45,77.43) | 2.24(2.03,2.45) | 25.91(43.57,14.35) | 16.74(27.07,9.60) | -1.00(-1.32,-0.68) |
| Pakistan | female | 38969.44(65327.13,22336.42) | 69437.07(105919.82,43811.59) | 1.75(1.68,1.83) | 140.82(244.15,78.35) | 115.01(179.48,71.59) | -0.96(-1.09,-0.84) |
| Palau | female | 2.09(3.81,0.94) | 3.77(6.80,1.79) | 1.97(1.88,2.05) | 38.28(68.19,17.12) | 32.16(57.22,15.78) | -0.45(-0.53,-0.37) |
| Palestine | female | 98.49(168.69,50.52) | 211.28(331.77,126.53) | 3.05(2.75,3.36) | 20.11(34.79,10.61) | 16.91(27.01,10.21) | -0.23(-0.50,0.05) |
| Panama | female | 560.38(696.20,423.66) | 1321.33(1800.05,930.48) | 3.42(3.24,3.61) | 71.68(89.50,53.86) | 62.70(85.38,44.07) | -0.09(-0.25,0.07) |
| Papua New Guinea | female | 154.83(248.84,88.15) | 404.48(677.29,223.39) | 3.65(3.43,3.86) | 13.08(21.17,7.42) | 12.65(20.83,7.10) | 0.06(-0.05,0.17) |
| Paraguay | female | 561.14(750.66,405.43) | 1125.11(1669.42,722.98) | 2.48(2.18,2.78) | 45.07(60.34,32.22) | 37.42(55.08,24.09) | -0.57(-0.86,-0.28) |
| Peru | female | 11590.68(15385.17,8460.83) | 19358.72(27920.28,12564.88) | 1.34(1.04,1.64) | 173.05(227.07,125.67) | 115.79(168.60,74.93) | -1.80(-2.06,-1.53) |
| Philippines | female | 9882.28(14288.30,7178.31) | 19542.79(26565.24,14086.52) | 2.24(2.06,2.43) | 56.26(83.19,40.23) | 43.57(59.04,31.47) | -1.04(-1.24,-0.83) |
| Poland | female | 21182.95(23323.72,19306.74) | 29944.20(38497.10,22997.73) | 1.62(1.32,1.91) | 86.34(94.59,78.91) | 96.94(125.65,74.02) | 0.77(0.49,1.06) |
| Portugal | female | 12091.59(14405.48,9743.04) | 5277.55(6460.10,4109.42) | -3.35(-3.54,-3.17) | 181.61(215.51,146.76) | 55.22(67.46,43.03) | -4.64(-4.84,-4.44) |
| Puerto Rico | female | 1956.45(2420.82,1478.62) | 2080.19(2958.55,1409.12) | -0.59(-0.90,-0.28) | 102.10(125.88,77.00) | 61.44(87.06,42.22) | -2.53(-2.85,-2.22) |
| Qatar | female | 10.96(17.81,6.57) | 57.04(97.33,31.47) | 6.32(6.05,6.59) | 29.13(47.51,17.02) | 28.89(48.12,15.85) | 0.68(0.23,1.14) |
| Republic of Korea | female | 29973.42(37730.30,22645.90) | 22269.06(27847.62,17152.74) | -1.94(-2.31,-1.58) | 158.64(199.42,119.34) | 49.35(61.81,38.06) | -4.97(-5.34,-4.60) |
| Republic of Moldova | female | 21094.81(25353.65,16460.46) | 15079.11(19105.48,11285.27) | -1.15(-1.55,-0.75) | 790.99(949.54,620.09) | 483.86(614.61,364.69) | -1.65(-2.08,-1.22) |
| Romania | female | 34087.03(40238.05,27955.07) | 41117.42(52968.15,31678.36) | 0.22(-0.18,0.62) | 226.82(266.69,188.28) | 238.98(311.09,181.18) | -0.26(-0.66,0.14) |
| Russian Federation | female | 64798.45(75780.42,54732.45) | 217645.45(279054.41,168690.73) | 4.43(3.42,5.45) | 60.09(70.00,50.62) | 190.84(245.86,147.11) | 4.22(3.13,5.32) |
| Rwanda | female | 4714.67(7220.47,2707.87) | 4581.28(6824.35,2733.79) | -0.45(-0.96,0.05) | 267.73(406.15,160.19) | 119.84(178.13,71.42) | -3.66(-4.07,-3.26) |
| Saint Kitts and Nevis | female | 26.50(34.47,19.30) | 21.65(31.51,13.75) | -0.51(-1.08,0.08) | 141.94(182.98,103.50) | 58.34(83.85,37.20) | -3.06(-3.41,-2.72) |
| Saint Lucia | female | 76.60(95.29,58.55) | 93.47(122.64,67.85) | 0.27(-0.29,0.84) | 160.34(199.97,121.08) | 82.60(108.89,60.01) | -2.81(-3.35,-2.27) |
| Saint Vincent and the Grenadines | female | 25.13(32.35,18.48) | 32.56(43.52,22.68) | 0.66(0.13,1.21) | 66.07(85.07,48.19) | 49.08(65.27,34.46) | -1.36(-1.83,-0.90) |
| Samoa | female | 25.41(39.75,15.31) | 36.19(60.16,19.70) | 1.27(1.18,1.36) | 53.52(84.35,32.24) | 45.84(75.66,25.24) | -0.50(-0.60,-0.39) |
| San Marino | female | 15.61(20.98,11.27) | 26.56(41.00,16.02) | 2.24(2.03,2.45) | 97.99(130.77,71.83) | 95.74(150.81,57.31) | 0.28(0.08,0.49) |
| Sao Tome and Principe | female | 55.53(83.90,34.24) | 72.46(121.30,38.09) | 0.41(0.11,0.71) | 159.66(240.22,98.96) | 118.21(196.67,60.93) | -1.49(-1.89,-1.08) |
| Saudi Arabia | female | 1061.45(1968.45,518.99) | 1625.18(2600.78,947.56) | 0.91(0.64,1.17) | 42.53(81.16,20.60) | 20.84(33.22,12.47) | -2.69(-2.84,-2.55) |
| Senegal | female | 2005.15(3105.52,1237.36) | 3853.89(6280.78,2087.44) | 2.31(2.10,2.53) | 111.81(170.92,69.02) | 88.77(144.98,48.45) | -0.78(-0.99,-0.56) |
| Serbia | female | 3980.36(5195.52,3023.54) | 3613.97(5030.23,2616.98) | -0.89(-1.13,-0.65) | 64.20(83.31,49.27) | 48.01(67.24,34.80) | -1.46(-1.65,-1.26) |
| Seychelles | female | 18.62(25.48,12.58) | 39.41(56.06,26.80) | 2.65(2.40,2.91) | 62.63(86.73,42.22) | 66.88(94.38,45.22) | 0.20(-0.10,0.50) |
| Sierra Leone | female | 1418.95(2220.59,879.82) | 2230.07(3796.53,1107.97) | 1.32(1.14,1.51) | 142.09(223.50,87.77) | 107.60(181.37,51.67) | -1.27(-1.49,-1.05) |
| Singapore | female | 189.07(264.09,131.51) | 308.67(432.63,211.31) | 1.82(1.62,2.01) | 14.69(20.86,10.12) | 7.64(10.69,5.25) | -2.17(-2.30,-2.04) |
| Slovakia | female | 5254.23(6462.44,4207.84) | 6138.52(8332.68,4449.58) | 1.16(0.78,1.54) | 169.92(209.12,135.69) | 141.78(194.34,102.00) | -0.07(-0.42,0.29) |
| Slovenia | female | 3159.53(4344.17,2222.21) | 1583.30(2197.78,1133.68) | -2.79(-3.04,-2.54) | 239.25(328.88,167.85) | 87.41(120.30,62.00) | -3.95(-4.22,-3.67) |
| Solomon Islands | female | 87.22(140.70,50.51) | 182.56(275.80,117.08) | 2.65(2.54,2.77) | 103.43(168.99,61.10) | 85.45(127.09,55.44) | -0.57(-0.67,-0.47) |
| Somalia | female | 2708.82(4701.26,1317.74) | 6290.34(11125.28,3238.95) | 2.94(2.82,3.06) | 170.51(291.29,84.49) | 147.20(259.66,77.90) | -0.46(-0.55,-0.36) |
| South Africa | female | 7927.41(10560.40,6189.91) | 10164.68(12866.61,7924.98) | 0.89(0.35,1.44) | 58.75(79.54,45.78) | 37.07(47.04,28.90) | -1.47(-1.90,-1.04) |
| South Sudan | female | 1406.13(2185.03,829.33) | 2282.20(3910.40,1265.68) | 1.92(1.68,2.16) | 125.23(199.14,74.11) | 110.40(184.16,63.58) | -0.26(-0.34,-0.17) |
| Spain | female | 33075.68(39416.74,26715.08) | 19596.76(23955.14,15723.82) | -2.05(-2.18,-1.91) | 122.02(143.50,100.34) | 44.97(54.19,36.67) | -3.70(-3.85,-3.54) |
| Sri Lanka | female | 2872.91(4114.68,1960.97) | 4464.73(6855.06,2807.21) | 1.33(1.05,1.61) | 47.14(66.64,32.14) | 31.37(47.69,20.09) | -1.57(-1.84,-1.31) |
| Sudan | female | 1241.13(2153.80,633.66) | 1900.81(3255.03,977.30) | 1.28(1.14,1.43) | 26.19(46.97,12.93) | 20.10(35.04,10.32) | -0.92(-0.96,-0.87) |
| Suriname | female | 213.95(275.31,158.86) | 346.53(472.27,239.17) | 1.34(1.06,1.62) | 148.93(190.76,110.54) | 105.22(142.51,72.99) | -1.67(-1.94,-1.40) |
| Sweden | female | 2732.79(3078.02,2395.50) | 2876.86(3422.21,2388.14) | 0.43(0.28,0.57) | 44.28(49.72,39.09) | 34.32(40.62,28.54) | -0.65(-0.81,-0.50) |
| Switzerland | female | 3144.92(3742.96,2498.17) | 3280.18(4113.28,2534.12) | -0.22(-0.44,0.00) | 67.32(80.24,52.76) | 44.66(55.21,34.53) | -1.82(-2.05,-1.58) |
| Syrian Arab Republic | female | 597.31(922.99,355.97) | 1080.77(1784.07,623.17) | 2.23(1.96,2.50) | 22.43(34.69,13.28) | 17.72(28.84,10.58) | -0.77(-1.02,-0.52) |
| Taiwan (Province of China) | female | 4709.68(6585.76,3279.86) | 6790.77(10122.34,4362.31) | 0.68(0.23,1.13) | 61.26(86.10,42.82) | 32.83(48.74,21.06) | -2.76(-3.21,-2.32) |
| Tajikistan | female | 3409.36(4288.12,2636.50) | 9192.02(12346.95,6549.65) | 3.09(2.80,3.37) | 208.37(261.42,159.96) | 282.59(375.49,206.65) | 0.79(0.61,0.98) |
| Thailand | female | 24861.99(34733.15,16878.45) | 37979.67(56627.69,23996.87) | 1.37(1.26,1.47) | 112.40(158.00,76.13) | 68.70(102.62,44.07) | -1.80(-1.91,-1.70) |
| Timor-Leste | female | 155.70(245.78,88.63) | 306.85(493.44,169.21) | 2.17(1.94,2.40) | 85.83(133.83,49.92) | 71.10(114.09,40.08) | -1.01(-1.25,-0.77) |
| Togo | female | 1185.68(1741.90,794.36) | 2523.32(3993.85,1441.33) | 2.45(2.35,2.55) | 151.70(221.31,101.39) | 103.31(163.78,58.22) | -1.53(-1.63,-1.43) |
| Tokelau | female | 0.50(0.86,0.25) | 0.32(0.53,0.16) | -1.81(-2.18,-1.43) | 71.39(121.99,35.96) | 46.61(78.83,24.43) | -1.48(-1.60,-1.36) |
| Tonga | female | 21.79(32.11,14.11) | 22.65(34.40,14.08) | 0.04(-0.08,0.16) | 68.92(101.79,45.35) | 52.91(80.49,32.82) | -1.00(-1.13,-0.87) |
| Trinidad and Tobago | female | 360.04(458.79,271.43) | 506.24(724.25,326.58) | 1.13(0.94,1.32) | 77.82(99.42,58.15) | 54.84(78.35,35.50) | -1.30(-1.47,-1.13) |
| Tunisia | female | 392.72(651.38,223.54) | 710.49(1195.76,393.77) | 2.17(2.11,2.23) | 15.24(24.95,8.64) | 10.76(18.06,6.04) | -1.03(-1.09,-0.96) |
| Turkey | female | 2571.74(4273.98,1440.59) | 3976.34(6132.96,2394.61) | 1.42(1.18,1.66) | 13.25(21.27,7.57) | 8.48(13.06,5.13) | -1.57(-1.82,-1.31) |
| Turkmenistan | female | 3256.54(4005.27,2571.86) | 9012.11(11995.98,6501.16) | 3.61(3.34,3.88) | 270.98(331.99,212.11) | 366.86(484.86,266.16) | 1.02(0.76,1.27) |
| Tuvalu | female | 3.23(5.53,1.58) | 2.84(5.03,1.41) | -0.38(-0.58,-0.18) | 76.41(130.05,38.07) | 50.88(89.17,25.65) | -1.32(-1.48,-1.15) |
| Uganda | female | 4189.75(6636.25,2275.75) | 9103.51(13909.44,4768.76) | 2.22(1.90,2.54) | 117.87(183.27,63.85) | 105.76(163.02,54.81) | -0.76(-1.05,-0.47) |
| Ukraine | female | 27873.18(33061.68,23549.05) | 72626.68(96522.65,52380.00) | 3.57(2.76,4.38) | 68.53(81.29,58.10) | 213.52(287.83,152.16) | 4.20(3.28,5.13) |
| United Arab Emirates | female | 29.57(50.52,16.56) | 165.53(288.47,84.24) | 6.02(5.80,6.25) | 19.14(34.22,10.15) | 13.05(23.79,6.37) | -1.10(-1.85,-0.33) |
| United Kingdom | female | 25250.01(28451.27,22214.71) | 47966.91(54352.20,42322.88) | 2.28(1.87,2.69) | 66.66(75.04,58.80) | 100.93(113.95,88.84) | 1.42(0.99,1.85) |
| United Republic of Tanzania | female | 8481.61(12651.01,5267.51) | 14548.85(23298.13,7280.29) | 1.88(1.76,1.99) | 138.90(207.26,86.97) | 103.84(169.14,50.84) | -0.90(-1.03,-0.76) |
| United States of America | female | 82908.74(97275.78,69824.72) | 138978.45(165703.40,115731.38) | 2.37(2.18,2.56) | 54.11(63.50,45.08) | 56.13(67.14,46.58) | 0.60(0.43,0.78) |
| United States Virgin Islands | female | 54.41(72.48,38.30) | 66.84(91.17,45.66) | 0.55(0.44,0.67) | 109.73(146.20,78.47) | 69.58(94.66,47.87) | -1.76(-1.87,-1.65) |
| Uruguay | female | 828.97(1135.58,587.69) | 601.49(836.38,416.08) | -1.28(-1.60,-0.97) | 41.84(57.02,29.59) | 22.70(31.43,15.64) | -2.24(-2.53,-1.96) |
| Uzbekistan | female | 16437.17(20217.09,12824.12) | 62453.50(80970.95,47402.85) | 3.72(3.18,4.26) | 241.21(297.77,186.66) | 428.38(544.79,325.22) | 1.15(0.54,1.77) |
| Vanuatu | female | 24.14(39.11,13.25) | 54.35(89.68,28.56) | 2.62(2.39,2.84) | 68.61(111.45,38.20) | 57.17(94.47,30.56) | -0.99(-1.22,-0.76) |
| Venezuela (Bolivarian Republic of) | female | 4866.45(5951.43,3755.64) | 8450.55(11862.10,5515.52) | 1.63(1.25,2.01) | 86.62(106.27,66.25) | 53.23(74.43,34.99) | -2.07(-2.47,-1.67) |
| Viet Nam | female | 16645.42(25070.32,9975.15) | 22437.10(32851.31,14309.76) | 0.76(0.34,1.19) | 71.14(108.32,42.68) | 41.99(62.22,26.72) | -2.09(-2.48,-1.70) |
| Yemen | female | 763.39(1337.47,381.36) | 1570.23(2514.65,868.94) | 2.38(2.29,2.47) | 28.38(50.93,14.07) | 21.07(34.29,11.38) | -1.19(-1.27,-1.12) |
| Zambia | female | 3479.14(5252.34,2204.27) | 6788.48(10443.92,4280.81) | 1.94(1.66,2.22) | 218.73(325.85,138.15) | 168.87(259.93,107.03) | -1.25(-1.45,-1.05) |
| Zimbabwe | female | 1746.97(2647.88,1095.10) | 3523.06(6665.97,1235.80) | 3.61(2.99,4.23) | 77.40(116.98,48.26) | 79.17(150.84,27.49) | 1.39(0.75,2.04) |

**Supplementary table 6. Age distribution of incidence rate for cirrhosis and other chronic liver diseases due to alcohol use in different countries in 2019.**

| **2019Incidence rate** | **15 to 19** | **20 to 24** | **25 to 29** | **30 to 34** | **35 to 39** | **40 to 44** | **45 to 49** | **50 to 54** | **55 to 59** | **60 to 64** | **65 to 69** | **70 to 74** | **75 to 79** | **80 plus** | **80-84** | **85-89** | **90-94** | **all ages** |
| --- | --- | --- | --- | --- | --- | --- | --- | --- | --- | --- | --- | --- | --- | --- | --- | --- | --- | --- |
| Afghanistan | 0.006507 | 0.035071 | 0.07677 | 0.303822 | 1.030571 | 2.313419 | 3.894122 | 4.693642 | 4.476297 | 3.531423 | 2.019407 | 0.989547 | 0.578984 | 0.53063 | 0.433828 | 0.556789 | 0.929801 | 0.593863 |
| Albania | 0.032502 | 0.95192 | 2.898238 | 7.88533 | 15.94698 | 25.98901 | 33.98841 | 33.25748 | 18.12259 | 1.601546 | 0.017638 | 0 | 0 | 0.054957 | 0 | 0 | 0 | 9.026595 |
| Algeria | 0.00707 | 0.043572 | 0.096794 | 0.303851 | 0.881215 | 1.842841 | 3.159763 | 4.161048 | 4.642091 | 4.302657 | 2.894092 | 1.639875 | 1.153201 | 0.631859 | 0.678661 | 0.458602 | 0.780536 | 1.065109 |
| American Samoa | 0.01688 | 0.268668 | 0.716977 | 2.117536 | 4.474248 | 6.275247 | 5.967347 | 3.387149 | 1.800349 | 0.984667 | 0.482526 | 0.091741 | 0.058041 | 0.413987 | 0.143477 | 0.103856 | 1.409559 | 1.47609 |
| Andorra | 0.036759 | 1.414792 | 4.530046 | 11.18185 | 22.42144 | 34.91784 | 38.49499 | 26.68523 | 7.411659 | 0.022042 | 0 | 0 | 0 | 0.509423 | 0 | 0.100704 | 0.755261 | 12.76013 |
| Angola | 0.038817 | 0.49899 | 1.028398 | 2.346444 | 4.311372 | 6.497568 | 9.35593 | 11.92127 | 12.81781 | 7.770063 | 2.355249 | 0.94915 | 0.73757 | 0.746722 | 0.496445 | 0.765152 | 1.763312 | 1.831196 |
| Antigua and Barbuda | 0.018322 | 0.548354 | 1.799509 | 5.359806 | 11.97313 | 19.88185 | 26.22393 | 26.71556 | 16.79627 | 6.598328 | 1.773703 | 0.179417 | 0.012403 | 0.123881 | 0 | 0.00843 | 0.363137 | 8.166513 |
| Argentina | 0.018243 | 0.439958 | 1.353202 | 4.287565 | 11.65728 | 23.7419 | 41.27394 | 43.82772 | 21.34677 | 2.284782 | 0.07549 | 0 | 0.000206 | 0.144509 | 0 | 0 | 0.10603 | 8.711984 |
| Armenia | 0.061003 | 1.299707 | 3.492876 | 10.44063 | 26.20493 | 44.85544 | 63.97614 | 69.99264 | 66.49111 | 44.28517 | 13.77353 | 2.301093 | 4.367497 | 2.396538 | 3.639176 | 0.079765 | 0.161185 | 21.97262 |
| Australia | 0.006929 | 0.132309 | 0.377773 | 1.161516 | 3.216953 | 6.713712 | 9.197912 | 6.933011 | 2.582874 | 0.415487 | 0.033947 | 0.001212 | 0.006056 | 0.255281 | 0.016931 | 0.073058 | 0.743236 | 2.025593 |
| Austria | 0.045854 | 1.773298 | 5.750281 | 14.61956 | 33.89944 | 60.73523 | 67.85357 | 37.69308 | 2.613182 | 0 | 0 | 0 | 0 | 0.007471 | 0 | 0 | 0 | 15.82468 |
| Azerbaijan | 0.074001 | 1.542591 | 4.020512 | 10.86631 | 22.36666 | 37.99994 | 64.13033 | 94.40265 | 96.3081 | 41.60686 | 1.83153 | 0.000249 | 0 | 0.511362 | 0.003645 | 1.068105 | 6.619579 | 23.97684 |
| Bahamas | 0.02086 | 0.647985 | 2.239152 | 7.111597 | 16.19418 | 25.01 | 27.41951 | 20.36824 | 10.54409 | 3.605946 | 0.809456 | 0.110422 | 0.000245 | 0.0191 | 0 | 0 | 0.010122 | 7.935554 |
| Bahrain | 0.008005 | 0.050323 | 0.116799 | 0.400528 | 1.327907 | 3.114504 | 5.399526 | 7.082653 | 7.805396 | 7.547394 | 5.703826 | 3.513042 | 2.26117 | 0.774729 | 0.605894 | 0.578255 | 4.395468 | 2.46743 |
| Bangladesh | 0.027885 | 0.585096 | 1.792086 | 4.59225 | 8.471353 | 12.10991 | 14.53394 | 12.57156 | 8.033454 | 2.511722 | 0.412723 | 0.458708 | 5.069334 | 4.989623 | 5.185997 | 4.127084 | 5.557036 | 3.796297 |
| Barbados | 0.018922 | 0.555811 | 1.810187 | 5.4371 | 12.06206 | 18.95618 | 20.54411 | 15.839 | 8.257596 | 2.944366 | 0.934858 | 0.200272 | 0.037066 | 0.03921 | 0.00221 | 0.007292 | 0.107544 | 6.06704 |
| Belarus | 0.054945 | 2.400459 | 10.40908 | 28.47928 | 40.67424 | 37.0731 | 22.16945 | 3.127596 | 0.051163 | 0 | 0 | 0 | 0 | 0.020355 | 0 | 0 | 0.004793 | 10.61619 |
| Belgium | 0.04452 | 1.645524 | 4.891425 | 12.04889 | 26.90471 | 48.38999 | 55.4744 | 32.06563 | 3.11797 | 0 | 0 | 0 | 0 | 0.922613 | 0 | 0.003018 | 1.680049 | 12.31684 |
| Belize | 0.02233 | 0.699554 | 2.477418 | 7.932122 | 19.68834 | 34.53089 | 40.52602 | 28.57166 | 11.23796 | 3.687337 | 0.556443 | 0.019215 | 2.59E-05 | 0.058377 | 0 | 0 | 0.072149 | 8.071859 |
| Benin | 0.028934 | 0.472524 | 1.271315 | 3.43629 | 6.074107 | 7.978331 | 13.05018 | 19.82436 | 15.82214 | 4.84774 | 2.403115 | 1.344267 | 1.275545 | 0.451921 | 0.231229 | 0.28416 | 1.290813 | 2.316604 |
| Bermuda | 0.019598 | 0.545415 | 1.640071 | 4.48819 | 9.831447 | 16.41062 | 19.44181 | 16.11942 | 9.847731 | 4.251192 | 1.730891 | 0.474986 | 0.092422 | 0.034985 | 0.012535 | 0.004461 | 0.031699 | 6.336913 |
| Bhutan | 0.058049 | 1.59395 | 4.993544 | 9.83308 | 14.21197 | 16.96865 | 17.73341 | 14.84135 | 10.54768 | 6.076592 | 1.891341 | 0.388686 | 0.512546 | 1.2751 | 0.686384 | 1.392727 | 2.819959 | 5.841047 |
| Bolivia (Plurinational State of) | 0.020117 | 0.587966 | 1.980583 | 6.611463 | 16.48458 | 31.36087 | 47.86527 | 54.28178 | 47.15224 | 29.88771 | 8.471442 | 0.471764 | 0.149666 | 0.336819 | 0.07164 | 0.283555 | 0.75938 | 10.81188 |
| Bosnia and Herzegovina | 0.03339 | 0.955929 | 2.809298 | 7.733477 | 17.55641 | 30.40943 | 38.22091 | 29.83971 | 8.546622 | 0.467398 | 0 | 0 | 0 | 0.008876 | 0 | 0 | 0 | 9.769256 |
| Botswana | 0.032185 | 0.615435 | 1.737896 | 4.441306 | 8.897834 | 14.55653 | 17.97664 | 13.41896 | 6.330615 | 2.151004 | 0.649427 | 0.280385 | 0.230088 | 0.302678 | 0.06013 | 0.200011 | 1.605834 | 3.993125 |
| Brazil | 0.025557 | 1.209957 | 3.812295 | 10.38419 | 21.48353 | 31.20938 | 33.22438 | 24.29346 | 11.74002 | 4.491479 | 1.48959 | 0.323282 | 0.251486 | 1.058084 | 0.386847 | 1.134776 | 2.132755 | 9.639669 |
| Brunei Darussalam | 0.026263 | 0.563586 | 1.466492 | 3.652631 | 8.34968 | 13.30619 | 13.72447 | 7.923795 | 2.342008 | 0.074815 | 0.000397 | 0 | 0 | 0.128436 | 0 | 0.001352 | 1.392467 | 3.860465 |
| Bulgaria | 0.05387 | 1.713232 | 6.121741 | 21.08086 | 45.12892 | 64.79352 | 54.73824 | 18.16923 | 2.255727 | 0.010626 | 0 | 0 | 0 | 0.034496 | 0 | 0 | 0.006693 | 15.53711 |
| Burkina Faso | 0.026787 | 0.41109 | 0.999132 | 2.647864 | 4.814182 | 6.36155 | 9.932841 | 15.41312 | 15.72157 | 7.074128 | 3.639654 | 2.441745 | 1.887256 | 1.300658 | 0.925221 | 1.34265 | 2.445183 | 2.031926 |
| Burundi | 0.025645 | 0.457196 | 1.182075 | 3.079072 | 6.174195 | 8.896347 | 12.60484 | 15.74843 | 15.76573 | 11.08526 | 7.020518 | 5.275117 | 1.70595 | 1.20084 | 0.278065 | 1.346332 | 5.254773 | 2.469635 |
| Cabo Verde | 0.035879 | 0.618392 | 1.761486 | 4.737751 | 7.986229 | 9.262028 | 11.22319 | 10.94855 | 7.273759 | 4.414149 | 3.083368 | 2.021464 | 1.692418 | 0.523667 | 0.510169 | 0.208304 | 0.448731 | 3.347336 |
| Cambodia | 0.046349 | 0.961865 | 2.900882 | 8.979051 | 19.95863 | 29.9135 | 35.27286 | 32.64992 | 25.14154 | 13.5204 | 4.497183 | 1.111766 | 0.850101 | 1.329516 | 0.904114 | 1.390398 | 2.634873 | 8.804686 |
| Cameroon | 0.031696 | 0.503656 | 1.284898 | 3.386108 | 6.010483 | 7.958074 | 12.7886 | 18.85531 | 15.59236 | 5.722297 | 2.7796 | 1.690543 | 1.311336 | 0.601849 | 0.383137 | 0.494001 | 1.670167 | 2.518975 |
| Canada | 0.013093 | 0.506559 | 1.816944 | 4.997819 | 11.23684 | 19.72755 | 25.79198 | 21.08409 | 6.494313 | 0.027756 | 0 | 0 | 0 | 0.162295 | 0 | 0.001603 | 0.050928 | 6.074292 |
| Central African Republic | 0.053309 | 0.825029 | 1.876006 | 4.254998 | 6.838563 | 8.421395 | 11.21293 | 13.00288 | 12.72854 | 5.449954 | 1.384658 | 0.471145 | 0.585447 | 0.819729 | 0.543407 | 0.967787 | 2.223956 | 2.513269 |
| Chad | 0.02962 | 0.423455 | 1.032899 | 2.858744 | 5.38494 | 6.992562 | 11.77284 | 18.64603 | 14.81431 | 3.602011 | 1.48874 | 0.847444 | 1.074014 | 0.530365 | 0.321574 | 0.411639 | 1.308069 | 1.80715 |
| Chile | 0.021131 | 0.55526 | 1.897365 | 7.137125 | 19.83768 | 40.15791 | 62.22139 | 59.41548 | 25.15943 | 1.371712 | 0.004229 | 0 | 0 | 0.091759 | 0 | 0 | 0.060121 | 14.3822 |
| China | 0.010116 | 0.280554 | 1.004375 | 3.876254 | 9.524134 | 14.1217 | 14.48457 | 10.55995 | 5.938999 | 2.446852 | 0.745445 | 0.146217 | 0.046145 | 0.131603 | 0.018138 | 0.117233 | 0.825281 | 4.87292 |
| Colombia | 0.01462 | 0.606202 | 1.929064 | 4.659108 | 9.372543 | 14.63077 | 18.89949 | 20.03622 | 15.88047 | 8.159734 | 1.854701 | 0.070394 | 0.022348 | 0.198654 | 0 | 0.000428 | 0.298817 | 5.646317 |
| Comoros | 0.031187 | 0.546315 | 1.416463 | 3.534198 | 6.581214 | 9.175459 | 12.05644 | 14.04519 | 13.18784 | 8.047623 | 4.91893 | 3.135477 | 0.706168 | 0.846967 | 0.06679 | 0.649299 | 3.900868 | 3.339445 |
| Congo | 0.033943 | 0.455135 | 0.981098 | 2.290229 | 4.147201 | 6.064673 | 8.964747 | 11.4106 | 12.09353 | 6.975769 | 2.031118 | 0.690783 | 0.580116 | 0.458047 | 0.297953 | 0.405247 | 1.245831 | 2.276604 |
| Cook Islands | 0.011184 | 0.186001 | 0.463386 | 1.133803 | 2.175073 | 3.137647 | 3.509237 | 2.446599 | 1.014124 | 0.323979 | 0.103157 | 0.023208 | 0.015163 | 0.086783 | 0.00913 | 0.035406 | 0.33149 | 0.913236 |
| Costa Rica | 0.018663 | 0.853814 | 3.330299 | 9.589591 | 22.20263 | 36.29448 | 44.91703 | 40.89688 | 24.03475 | 7.780877 | 0.40529 | 0 | 0 | 0.078009 | 0 | 0 | 0.002972 | 11.78335 |
| Côte d'Ivoire | 0.032489 | 0.496168 | 1.253097 | 3.431759 | 6.177118 | 8.096219 | 13.55398 | 20.817 | 16.99055 | 5.218925 | 2.272136 | 1.222126 | 1.330306 | 0.602681 | 0.283863 | 0.449425 | 1.895186 | 2.811953 |
| Croatia | 0.044296 | 1.342251 | 4.285787 | 12.89334 | 31.23669 | 59.76867 | 72.2572 | 44.73808 | 6.783216 | 0.032119 | 0 | 0 | 0 | 0.015881 | 0 | 0 | 0 | 15.98507 |
| Cuba | 0.019521 | 0.589209 | 1.964863 | 6.137074 | 15.91128 | 26.37556 | 26.59465 | 16.31989 | 6.448126 | 2.432204 | 0.277631 | 0.005953 | 0 | 0.008882 | 0 | 0 | 0.000214 | 7.566821 |
| Cyprus | 0.027439 | 1.071376 | 3.420302 | 8.199479 | 16.11431 | 27.03175 | 36.01592 | 32.37911 | 14.1638 | 0.450522 | 0.001148 | 0 | 0 | 1.722584 | 0.055264 | 2.263174 | 12.28214 | 9.954107 |
| Czechia | 0.04667 | 1.482531 | 4.788695 | 14.59869 | 30.664 | 42.84429 | 36.96078 | 14.3557 | 0.59208 | 0.000139 | 0 | 0 | 0 | 0.081995 | 0 | 0 | 0.048347 | 11.0764 |
| Democratic People's Republic of Korea | 0.012717 | 0.331002 | 1.180316 | 4.239556 | 9.645158 | 13.3464 | 13.53427 | 9.456182 | 4.684832 | 2.147309 | 0.707042 | 0.098877 | 0.021888 | 0.089299 | 0.006586 | 0.043411 | 0.433622 | 4.282628 |
| Democratic Republic of the Congo | 0.050776 | 0.707937 | 1.527907 | 3.59176 | 6.380476 | 8.631302 | 11.89255 | 14.51125 | 14.78018 | 7.144962 | 1.717728 | 0.637658 | 0.584704 | 0.556027 | 0.319648 | 0.469445 | 1.501855 | 2.437585 |
| Denmark | 0.041416 | 1.508048 | 4.589808 | 11.13679 | 23.88605 | 40.78165 | 40.15996 | 16.24762 | 0.258419 | 0 | 0 | 0 | 0 | 0.165478 | 0 | 0.001188 | 0.129104 | 8.954853 |
| Djibouti | 0.03288 | 0.552796 | 1.352214 | 3.151423 | 5.929533 | 8.628542 | 11.83534 | 13.98385 | 13.43989 | 8.119264 | 4.488318 | 2.554422 | 0.389062 | 0.466648 | 0.005395 | 0.252358 | 3.103728 | 3.189611 |
| Dominica | 0.01739 | 0.518813 | 1.729924 | 5.395995 | 12.54647 | 18.79018 | 20.43107 | 16.9451 | 9.977624 | 4.202251 | 1.186675 | 0.171974 | 0.020957 | 0.160885 | 0.00291 | 0.050638 | 0.495753 | 5.738666 |
| Dominican Republic | 0.025597 | 0.819074 | 2.94615 | 9.424513 | 21.17373 | 34.04603 | 43.29905 | 41.94984 | 28.85054 | 11.822 | 2.182157 | 0.141417 | 0.099535 | 0.699161 | 0.22283 | 0.547174 | 1.269927 | 10.62581 |
| Ecuador | 0.019669 | 0.640287 | 2.075012 | 5.988444 | 13.69549 | 27.15865 | 53.64088 | 78.57894 | 76.87959 | 47.43345 | 11.75487 | 0.46953 | 0.009218 | 0.215677 | 0 | 0.00905 | 0.15546 | 14.56445 |
| Egypt | 0.016156 | 0.085856 | 0.181524 | 0.649064 | 2.113369 | 5.070759 | 9.974843 | 14.47274 | 17.27334 | 16.39881 | 10.99234 | 5.218288 | 2.37911 | 2.40742 | 2.399408 | 2.47455 | 1.903978 | 2.984579 |
| El Salvador | 0.022429 | 1.334934 | 6.262629 | 16.02059 | 26.72523 | 34.8051 | 41.11012 | 39.29787 | 26.39359 | 9.575638 | 0.556332 | 0.001762 | 0.001338 | 0.268097 | 0.000178 | 0.000527 | 0.168645 | 10.89185 |
| Equatorial Guinea | 0.044436 | 0.552468 | 1.106708 | 2.493527 | 4.504897 | 6.70111 | 9.706671 | 12.10843 | 12.28073 | 7.262358 | 2.411573 | 0.754739 | 0.423764 | 0.438111 | 0.232751 | 0.430193 | 1.1562 | 1.750051 |
| Eritrea | 0.033383 | 0.645635 | 1.761934 | 4.589639 | 8.9893 | 12.69654 | 16.39204 | 17.99909 | 16.08027 | 9.204857 | 5.774502 | 4.70679 | 0.918507 | 0.366872 | 0.01655 | 0.31362 | 2.958728 | 3.449142 |
| Estonia | 0.051469 | 1.970771 | 8.553505 | 24.41933 | 37.87396 | 35.28381 | 15.88932 | 0.453693 | 0.000858 | 0 | 0 | 0 | 0 | 0.015185 | 0 | 0 | 0.006051 | 8.667036 |
| Eswatini | 0.028126 | 0.530139 | 1.498219 | 3.703358 | 6.732845 | 9.74413 | 10.80251 | 7.220694 | 3.525031 | 1.482947 | 0.579092 | 0.310838 | 0.359221 | 0.252537 | 0.164781 | 0.29881 | 0.667564 | 2.174622 |
| Ethiopia | 0.022086 | 0.325344 | 0.701971 | 1.84316 | 3.907193 | 6.368829 | 9.242643 | 11.57009 | 12.78324 | 10.81266 | 7.661097 | 4.999132 | 2.387771 | 2.355217 | 1.212831 | 2.916029 | 6.439986 | 1.789887 |
| Fiji | 0.014134 | 0.241972 | 0.68339 | 2.001531 | 3.872482 | 5.259019 | 5.092386 | 3.383593 | 2.033041 | 0.879381 | 0.465665 | 0.120305 | 0.063674 | 0.203979 | 0.064628 | 0.033912 | 1.334863 | 1.453093 |
| Finland | 0.040005 | 1.586817 | 5.485118 | 15.39005 | 31.92295 | 43.78512 | 30.8788 | 3.642633 | 0.000454 | 0 | 0 | 0 | 0 | 0.109126 | 0 | 0 | 0.108145 | 8.232479 |
| France | 0.033785 | 1.301602 | 4.142483 | 10.49825 | 23.73656 | 42.65148 | 44.448 | 22.38559 | 1.811364 | 0.002093 | 0 | 0 | 0 | 0.865534 | 0 | 0.010713 | 1.681733 | 9.722539 |
| Gabon | 0.025384 | 0.350142 | 0.784721 | 1.920096 | 3.61936 | 5.404119 | 7.664546 | 9.260752 | 9.41763 | 5.591916 | 2.082432 | 1.011723 | 0.766068 | 0.645759 | 0.451543 | 0.610797 | 1.309074 | 2.017694 |
| Gambia | 0.029785 | 0.491456 | 1.357382 | 3.624702 | 6.330023 | 8.132638 | 13.09873 | 20.50112 | 17.71728 | 5.389347 | 2.231452 | 1.086695 | 0.959967 | 0.370106 | 0.179783 | 0.262791 | 1.453625 | 2.533049 |
| Georgia | 0.046625 | 1.30791 | 4.957473 | 20.28173 | 41.41447 | 49.27071 | 38.4311 | 22.41111 | 16.21672 | 7.582758 | 0.449756 | 0 | 0 | 0.083574 | 0 | 0.000746 | 0.096948 | 13.44434 |
| Germany | 0.043194 | 1.705686 | 5.707505 | 15.19165 | 34.94525 | 60.60997 | 64.14597 | 33.8365 | 2.539892 | 0.00047 | 0 | 0 | 0 | 0.08914 | 0 | 0 | 0.088353 | 14.30839 |
| Ghana | 0.02702 | 0.421718 | 1.071897 | 2.902846 | 5.2302 | 6.681032 | 9.977497 | 14.40707 | 13.07027 | 6.117224 | 3.537593 | 2.43132 | 1.054123 | 0.496473 | 0.228269 | 0.479693 | 1.61182 | 2.484841 |
| Greece | 0.032723 | 1.190478 | 3.620749 | 8.322853 | 16.38238 | 27.27883 | 29.39708 | 17.67448 | 4.404103 | 0.191941 | 0.005001 | 0 | 0 | 0.194946 | 0 | 0.012892 | 0.440719 | 7.832479 |
| Greenland | 0.011848 | 0.478079 | 1.944241 | 5.66219 | 11.92109 | 16.55664 | 14.80106 | 5.582223 | 0.179378 | 0 | 0 | 0 | 0 | 0.037881 | 0 | 0 | 0.059279 | 3.67466 |
| Grenada | 0.019949 | 0.620206 | 2.20758 | 8.033531 | 20.07839 | 29.50426 | 31.704 | 28.11147 | 19.59462 | 6.536981 | 0.55186 | 0.034232 | 0.00284 | 0.156032 | 0 | 0 | 0.658901 | 9.393549 |
| Guam | 0.028525 | 0.516069 | 1.427866 | 3.832902 | 7.102755 | 8.794167 | 7.284779 | 3.174725 | 1.635211 | 0.73831 | 0.436077 | 0.141954 | 0.111798 | 0.57973 | 0.664136 | 0.226898 | 0.22471 | 2.123666 |
| Guatemala | 0.026653 | 1.688162 | 7.795814 | 22.69309 | 41.15116 | 53.90036 | 59.79864 | 47.14035 | 25.89988 | 4.550644 | 0.030978 | 0 | 0.037633 | 0.131016 | 0 | 0 | 0.881824 | 12.87519 |
| Guinea | 0.024292 | 0.345278 | 0.839959 | 2.210719 | 4.116 | 5.506283 | 9.009914 | 14.26537 | 12.4781 | 4.568787 | 2.116273 | 1.210092 | 1.24264 | 0.738113 | 0.421381 | 0.52308 | 1.597029 | 1.686573 |
| Guinea-Bissau | 0.034634 | 0.548674 | 1.380291 | 3.480701 | 5.760317 | 6.801739 | 11.0182 | 16.99466 | 13.35462 | 4.089841 | 1.722433 | 1.053937 | 1.340303 | 0.550499 | 0.399965 | 0.376626 | 1.359574 | 2.186258 |
| Guyana | 0.025747 | 0.884091 | 3.539027 | 13.46041 | 32.80139 | 50.54035 | 59.4981 | 43.80224 | 12.41398 | 1.835216 | 0.166588 | 0.009173 | 9.89E-06 | 0.248435 | 0 | 0.028365 | 0.81648 | 12.71212 |
| Haiti | 0.014634 | 0.444294 | 1.670451 | 6.459915 | 16.96416 | 30.22016 | 40.04219 | 35.71714 | 17.88918 | 5.338754 | 1.049523 | 0.165727 | 0.086538 | 0.482041 | 0.118962 | 0.57365 | 1.858843 | 7.258442 |
| Honduras | 0.014893 | 0.710301 | 3.223607 | 10.61206 | 25.41948 | 42.00736 | 50.22344 | 41.90644 | 19.34579 | 2.785856 | 0.183751 | 0.014678 | 0.01207 | 0.202745 | 0.068616 | 0.15664 | 0.364486 | 9.18783 |
| Hungary | 0.049236 | 1.558289 | 4.655981 | 13.56779 | 33.4023 | 62.46174 | 64.45225 | 26.32229 | 0.626981 | 0 | 0 | 0 | 0 | 0.012382 | 0 | 0 | 0.002262 | 15.71553 |
| Iceland | 0.0221 | 0.771152 | 2.455114 | 5.426991 | 9.550316 | 12.95609 | 13.00843 | 8.232907 | 1.524021 | 0.00276 | 0 | 0 | 0 | 0.078129 | 0 | 0 | 0.071294 | 3.528154 |
| India | 0.056243 | 1.411916 | 3.8034 | 8.119361 | 13.23517 | 15.93234 | 15.03163 | 10.83567 | 7.153408 | 4.474873 | 2.484428 | 1.403897 | 1.748106 | 2.734059 | 1.978507 | 3.533968 | 4.9537 | 4.958705 |
| Indonesia | 0.012605 | 0.2097 | 0.593882 | 2.397644 | 6.18277 | 9.401438 | 10.89971 | 10.60837 | 9.562351 | 7.365332 | 5.21831 | 3.771869 | 4.069336 | 7.024943 | 5.43226 | 9.16137 | 11.59915 | 3.784462 |
| Iran (Islamic Republic of) | 0.005883 | 0.040589 | 0.091865 | 0.284849 | 0.783375 | 1.597468 | 2.616397 | 3.377492 | 3.702274 | 3.319086 | 2.442084 | 1.654037 | 1.139905 | 0.833346 | 0.822096 | 0.792382 | 0.840476 | 0.970306 |
| Iraq | 0.00725 | 0.044788 | 0.101687 | 0.326741 | 0.978574 | 2.038226 | 3.356503 | 4.14173 | 4.124363 | 3.008427 | 1.508005 | 0.706407 | 0.426941 | 0.209818 | 0.264367 | 0.057703 | 0.12174 | 0.746074 |
| Ireland | 0.034786 | 1.3338 | 4.206162 | 9.95933 | 19.26146 | 26.57667 | 23.25972 | 11.53451 | 1.746647 | 0.008519 | 0 | 0 | 0 | 0.246857 | 0 | 0.018366 | 0.416741 | 7.041324 |
| Israel | 0.025579 | 0.92416 | 2.874898 | 6.74984 | 13.38316 | 22.36243 | 29.24808 | 24.67484 | 10.58988 | 0.654143 | 0.006805 | 0 | 0 | 0.945662 | 0.005139 | 0.16522 | 3.17383 | 6.388377 |
| Italy | 0.016603 | 0.518233 | 1.585279 | 5.101989 | 15.27828 | 30.42067 | 36.77572 | 28.66928 | 14.16552 | 4.00642 | 0.251721 | 0.000741 | 0 | 0.087012 | 0 | 0.002258 | 0.057248 | 10.1761 |
| Jamaica | 0.013594 | 0.361737 | 1.022764 | 2.570734 | 5.068762 | 7.775184 | 10.06297 | 9.960217 | 6.595181 | 3.096701 | 0.792941 | 0.073393 | 0.013955 | 0.120229 | 0.00081 | 0 | 0.123738 | 2.772341 |
| Japan | 0.019262 | 0.447379 | 1.327714 | 4.401124 | 12.69601 | 20.91348 | 17.52663 | 6.244797 | 0.965341 | 0.053943 | 0.002217 | 0 | 0 | 0.088545 | 0 | 0.000256 | 0.030277 | 4.406279 |
| Jordan | 0.006394 | 0.039273 | 0.087905 | 0.281622 | 0.805986 | 1.712729 | 3.000318 | 4.065411 | 4.623686 | 3.924116 | 2.319389 | 1.25868 | 0.510941 | 0.202291 | 0.234496 | 0.102216 | 0.121134 | 0.816879 |
| Kazakhstan | 0.092606 | 2.744718 | 9.312584 | 27.76505 | 52.2794 | 69.88186 | 97.06819 | 103.1513 | 60.67698 | 8.622911 | 0.039483 | 0 | 0 | 1.020258 | 0 | 0 | 7.280747 | 26.86921 |
| Kenya | 0.034799 | 0.710762 | 1.98211 | 5.63557 | 11.95421 | 18.58988 | 24.54543 | 27.47985 | 26.62211 | 21.33623 | 16.18107 | 10.45811 | 4.9406 | 4.807376 | 2.442866 | 5.914918 | 12.67743 | 5.350069 |
| Kiribati | 0.023149 | 0.415911 | 1.099573 | 3.106958 | 6.582704 | 8.864897 | 7.610005 | 3.887845 | 2.104282 | 0.993329 | 0.494712 | 0.200532 | 0.195764 | 0.250972 | 0.140557 | 0.251153 | 1.040141 | 1.888505 |
| Kuwait | 0.007396 | 0.046495 | 0.104166 | 0.316523 | 0.908113 | 1.900581 | 3.014342 | 3.581249 | 3.260763 | 2.426305 | 1.397759 | 0.482868 | 0.375246 | 0.175823 | 0.209918 | 0.084897 | 0.091962 | 1.007427 |
| Kyrgyzstan | 0.093026 | 2.603991 | 8.889507 | 29.48087 | 51.84793 | 59.66989 | 62.99591 | 45.83737 | 18.88444 | 0.746448 | 0 | 0 | 0 | 0.00157 | 0 | 0 | 0 | 16.28294 |
| Lao People's Democratic Republic | 0.02166 | 0.382027 | 1.161687 | 3.788953 | 8.898157 | 14.17145 | 16.97081 | 15.39265 | 11.11309 | 5.610124 | 2.188914 | 0.660691 | 0.565979 | 0.679798 | 0.445626 | 0.6475 | 1.35741 | 3.876986 |
| Latvia | 0.045586 | 1.777864 | 7.351823 | 21.66093 | 33.92864 | 29.68714 | 12.76922 | 0.671774 | 0.011449 | 0 | 0 | 0 | 0 | 0.005822 | 0 | 0 | 0 | 7.231419 |
| Lebanon | 0.00799 | 0.047494 | 0.102179 | 0.305336 | 0.831164 | 1.677796 | 2.950646 | 4.202238 | 4.977446 | 4.723538 | 3.38126 | 2.068313 | 1.320017 | 0.686183 | 0.730129 | 0.533622 | 0.673556 | 1.141877 |
| Lesotho | 0.028804 | 0.579438 | 1.771057 | 4.692923 | 8.449662 | 11.64571 | 12.70614 | 7.782085 | 2.885334 | 1.002119 | 0.402851 | 0.233367 | 0.343788 | 0.358178 | 0.242903 | 0.38394 | 0.883221 | 2.771306 |
| Liberia | 0.02386 | 0.332728 | 0.78788 | 2.09871 | 4.060818 | 5.856802 | 9.267436 | 13.44209 | 12.02256 | 5.417771 | 2.920026 | 1.897323 | 1.53194 | 0.716384 | 0.457709 | 0.497427 | 1.602769 | 2.025244 |
| Libya | 0.006575 | 0.040728 | 0.090737 | 0.281707 | 0.788603 | 1.598652 | 2.734546 | 3.676094 | 4.066393 | 3.695481 | 2.611948 | 1.575757 | 0.853986 | 0.368448 | 0.377312 | 0.22936 | 0.356725 | 0.986451 |
| Lithuania | 0.061371 | 2.774413 | 12.81521 | 39.14364 | 49.89678 | 30.89446 | 8.565389 | 0.15817 | 0 | 0 | 0 | 0 | 0 | 0.011086 | 0 | 0 | 0 | 9.090854 |
| Luxembourg | 0.045219 | 1.733045 | 5.637139 | 14.29961 | 30.73149 | 51.98696 | 57.55358 | 33.6386 | 3.992747 | 0.00082 | 0 | 0 | 0 | 0.284905 | 0 | 0 | 0.273504 | 15.16259 |
| Madagascar | 0.031885 | 0.585424 | 1.544329 | 3.756226 | 7.120788 | 9.995238 | 13.28375 | 15.22038 | 13.64918 | 7.338026 | 4.154215 | 3.358648 | 0.535694 | 0.643091 | 0.015871 | 0.32998 | 3.758296 | 2.797723 |
| Malawi | 0.035885 | 0.626801 | 1.564295 | 3.844338 | 7.198947 | 10.58225 | 14.85493 | 18.01015 | 19.00329 | 13.78002 | 8.5018 | 5.950849 | 1.502296 | 1.03498 | 0.085718 | 0.545514 | 4.338562 | 2.868003 |
| Malaysia | 0.019819 | 0.30447 | 0.805608 | 2.515712 | 6.363151 | 10.56523 | 13.1973 | 12.82226 | 9.602321 | 5.948698 | 3.033451 | 1.026154 | 0.472023 | 0.411219 | 0.418857 | 0.398853 | 0.260598 | 3.570082 |
| Maldives | 0.016517 | 0.261768 | 0.684698 | 1.871839 | 4.052832 | 6.367774 | 8.340019 | 9.103938 | 8.176022 | 5.512598 | 3.138902 | 1.466766 | 0.648227 | 0.2355 | 0.258294 | 0.135228 | 0.141265 | 2.630498 |
| Mali | 0.028938 | 0.459112 | 1.229564 | 3.442731 | 6.313147 | 8.16129 | 12.5001 | 17.78099 | 13.72816 | 4.421969 | 2.102393 | 1.296616 | 1.233739 | 0.639842 | 0.345483 | 0.490814 | 1.750965 | 2.11127 |
| Malta | 0.028792 | 1.100091 | 3.46117 | 8.22972 | 15.37284 | 21.86306 | 21.52824 | 13.71047 | 3.837529 | 0.014455 | 0 | 0 | 0 | 0.092111 | 0 | 0 | 0.19751 | 5.965956 |
| Marshall Islands | 0.018126 | 0.344408 | 1.050131 | 3.345387 | 7.082964 | 10.03872 | 9.910755 | 5.450998 | 2.307402 | 0.865172 | 0.365191 | 0.112236 | 0.101959 | 0.40477 | 0.251159 | 0.382958 | 1.182878 | 2.407274 |
| Mauritania | 0.027978 | 0.402479 | 1.010793 | 2.693503 | 4.949868 | 6.729572 | 10.38793 | 14.63087 | 12.08071 | 4.41986 | 2.029127 | 0.927877 | 0.632305 | 0.335742 | 0.0966 | 0.241612 | 1.065173 | 2.131158 |
| Mauritius | 0.02439 | 0.452201 | 1.393218 | 4.494435 | 9.371059 | 12.00789 | 10.20303 | 6.359235 | 3.755787 | 1.530684 | 0.7612 | 0.391457 | 0.409717 | 0.228276 | 0.306977 | 0.052408 | 0.075652 | 3.620984 |
| Mexico | 0.017893 | 0.942432 | 4.244226 | 15.37519 | 36.06716 | 57.04141 | 72.26526 | 71.53099 | 49.96912 | 19.39553 | 3.073571 | 0.477924 | 0.282957 | 0.922878 | 0.25063 | 0.842352 | 1.917057 | 19.29465 |
| Micronesia (Federated States of) | 0.021356 | 0.417514 | 1.289495 | 4.099987 | 8.489684 | 11.44965 | 9.902305 | 4.708465 | 2.140167 | 0.915882 | 0.470562 | 0.125605 | 0.071557 | 0.330907 | 0.126557 | 0.222629 | 1.477642 | 2.490992 |
| Monaco | 0.036385 | 1.462799 | 5.225563 | 14.74121 | 31.18182 | 45.94315 | 40.90895 | 18.81132 | 1.409925 | 0 | 0 | 0 | 0 | 0.268695 | 0 | 0.015517 | 0.275207 | 10.20085 |
| Mongolia | 0.087667 | 2.574879 | 7.921135 | 21.16689 | 42.0649 | 67.0377 | 110.7914 | 161.3972 | 137.1567 | 35.37071 | 0.228072 | 0 | 0 | 0.670045 | 0 | 1.25889 | 7.122435 | 33.73336 |
| Montenegro | 0.033084 | 0.92188 | 2.599953 | 6.42379 | 11.79637 | 15.11797 | 13.10873 | 6.204231 | 1.204682 | 0.051123 | 0.00167 | 0 | 0 | 0.025414 | 0 | 0 | 0.071346 | 3.975496 |
| Morocco | 0.006923 | 0.041416 | 0.091772 | 0.299587 | 0.876586 | 1.852737 | 3.283401 | 4.566322 | 5.07972 | 4.35072 | 2.622526 | 1.157563 | 0.528238 | 0.394326 | 0.372158 | 0.288617 | 0.430435 | 1.171549 |
| Mozambique | 0.032658 | 0.454397 | 0.954141 | 2.0993 | 3.967522 | 5.804073 | 6.887375 | 7.26889 | 8.750534 | 9.053893 | 6.097527 | 4.703769 | 2.160551 | 1.096834 | 0.274938 | 0.975249 | 4.492907 | 1.452431 |
| Myanmar | 0.040006 | 0.909147 | 2.90429 | 9.57326 | 19.18199 | 20.36814 | 14.37823 | 7.4343 | 3.825916 | 1.124644 | 0.190417 | 0.022 | 0.020942 | 0.27633 | 0.011945 | 0.173168 | 1.149967 | 5.215998 |
| Namibia | 0.028371 | 0.524212 | 1.43179 | 3.657296 | 7.573987 | 13.50604 | 18.31841 | 14.97117 | 7.333956 | 2.341774 | 0.816852 | 0.37292 | 0.345152 | 0.401082 | 0.165909 | 0.411853 | 1.091379 | 3.269835 |
| Nauru | 0.023677 | 0.469571 | 1.415177 | 4.122031 | 8.031068 | 10.16132 | 8.366604 | 3.868015 | 1.9028 | 0.856729 | 0.391055 | 0.059393 | 0.04496 | 0.264725 | 0.137051 | 0.210925 | 0.725885 | 2.176066 |
| Nepal | 0.08472 | 2.258137 | 6.68834 | 14.28768 | 22.22303 | 27.40952 | 29.29104 | 24.40468 | 16.31907 | 8.515546 | 2.050611 | 0.294614 | 0.541809 | 1.187443 | 0.67575 | 1.207712 | 2.882655 | 8.017622 |
| Netherlands | 0.031008 | 1.146304 | 3.423536 | 7.624283 | 14.10997 | 21.56108 | 25.51662 | 19.56537 | 6.853449 | 0.195111 | 0.001286 | 0 | 0 | 2.164232 | 0.003154 | 0.593692 | 8.096586 | 6.753724 |
| New Zealand | 0.005023 | 0.103469 | 0.281491 | 0.830513 | 2.226218 | 4.457455 | 6.248158 | 5.867978 | 3.514982 | 1.209348 | 0.314655 | 0.075796 | 0.06328 | 0.227927 | 0.092212 | 0.284264 | 0.377379 | 1.630136 |
| Nicaragua | 0.021442 | 1.250243 | 6.399766 | 18.64624 | 33.31124 | 42.19828 | 44.76989 | 34.35768 | 18.27412 | 4.603533 | 0.064118 | 0 | 0 | 0.007135 | 0 | 0 | 0.001459 | 11.33563 |
| Niger | 0.031532 | 0.489756 | 1.33974 | 3.867762 | 6.924646 | 8.538536 | 12.93133 | 19.48876 | 15.37431 | 4.555225 | 2.529345 | 1.417304 | 1.265649 | 0.806865 | 0.502381 | 0.658304 | 2.125457 | 2.023055 |
| Nigeria | 0.023804 | 0.358181 | 0.800069 | 2.137653 | 4.559711 | 7.501412 | 11.2661 | 14.2323 | 13.66369 | 9.844845 | 6.741134 | 4.575326 | 3.755891 | 3.838073 | 2.909782 | 4.261842 | 6.163526 | 2.199703 |
| Niue | 0.017934 | 0.327684 | 0.991665 | 3.025499 | 6.444747 | 8.997868 | 8.38389 | 5.067188 | 2.410712 | 1.10192 | 0.526426 | 0.101821 | 0.049784 | 0.335943 | 0.044336 | 0.05776 | 1.389563 | 2.349022 |
| North Macedonia | 0.032165 | 0.895558 | 2.645386 | 7.240274 | 16.02966 | 28.43895 | 34.86484 | 27.19241 | 8.567196 | 0.306122 | 0 | 0 | 0 | 0.021769 | 0 | 0 | 0.127919 | 9.190491 |
| Northern Mariana Islands | 0.031767 | 0.504696 | 1.363012 | 4.432755 | 10.7602 | 12.611 | 17.22204 | 9.767471 | 4.417046 | 2.573921 | 1.016142 | 0.187091 | 0.113929 | 1.101217 | 0.213725 | 0.391917 | 4.744127 | 4.727604 |
| Norway | 0.030494 | 1.108911 | 3.105486 | 7.156969 | 14.4951 | 21.37011 | 21.8568 | 14.95816 | 5.216278 | 0.484032 | 0.048891 | 0.001282 | 0.002297 | 0.767691 | 0.038411 | 0.484217 | 1.663095 | 6.109211 |
| Oman | 0.007106 | 0.049353 | 0.119123 | 0.387025 | 1.184444 | 2.812431 | 5.606358 | 8.278611 | 9.721811 | 8.87937 | 5.45878 | 2.873468 | 1.587067 | 0.359005 | 0.332502 | 0.239226 | 0.607395 | 1.420715 |
| Pakistan | 0.021103 | 0.253816 | 0.548756 | 1.137238 | 2.160324 | 3.261877 | 4.322868 | 4.669751 | 4.386827 | 3.209283 | 1.593798 | 0.766263 | 0.937104 | 1.807464 | 1.265131 | 2.342861 | 3.270168 | 0.983273 |
| Palau | 0.019745 | 0.41834 | 1.402539 | 4.242214 | 8.194458 | 9.888621 | 7.942571 | 3.760812 | 1.923578 | 0.943219 | 0.408089 | 0.086726 | 0.047058 | 0.235072 | 0.050645 | 0.078776 | 1.132614 | 3.15125 |
| Palestine | 0.006642 | 0.040451 | 0.089251 | 0.274483 | 0.774755 | 1.717665 | 3.521273 | 5.556418 | 6.571009 | 5.773405 | 3.050672 | 0.840044 | 0.398055 | 0.38599 | 0.350069 | 0.389506 | 0.321078 | 0.839859 |
| Panama | 0.015361 | 0.657781 | 2.291338 | 5.931246 | 13.04407 | 22.21997 | 30.5891 | 32.0484 | 23.63085 | 10.1144 | 1.19756 | 0.002608 | 0 | 0.024062 | 0.002052 | 0.001384 | 0.001498 | 7.843957 |
| Papua New Guinea | 0.011951 | 0.226683 | 0.664801 | 1.78839 | 2.867292 | 2.670001 | 1.669267 | 0.640928 | 0.28973 | 0.121518 | 0.048222 | 0.010245 | 0.009633 | 0.04439 | 0.020132 | 0.02307 | 0.140073 | 0.654672 |
| Paraguay | 0.029119 | 0.971553 | 2.835309 | 7.063612 | 15.29926 | 23.80148 | 26.38779 | 20.67729 | 10.1051 | 3.202905 | 1.113908 | 0.304444 | 0.150356 | 0.182098 | 0.029489 | 0.010152 | 0.094055 | 6.14454 |
| Peru | 0.024932 | 0.706132 | 2.151542 | 6.300605 | 14.97845 | 28.1915 | 44.2298 | 53.332 | 49.46716 | 30.25378 | 7.761015 | 0.391694 | 0.040982 | 0.225917 | 0.003674 | 0.035572 | 0.191885 | 12.2123 |
| Philippines | 0.013752 | 0.277269 | 0.839328 | 2.93436 | 7.149516 | 11.34418 | 13.20787 | 11.05494 | 7.680752 | 4.644563 | 2.286879 | 1.03723 | 0.835685 | 1.068263 | 0.856228 | 1.260444 | 1.442864 | 3.119238 |
| Poland | 0.049833 | 1.557962 | 5.035975 | 15.70594 | 31.12252 | 35.90477 | 24.71268 | 7.20595 | 0.660038 | 0.024653 | 0.004817 | 0 | 0 | 0.062341 | 0 | 0.002977 | 0.09561 | 9.168257 |
| Portugal | 0.025111 | 1.07478 | 3.78031 | 10.68237 | 24.07939 | 40.04556 | 38.64229 | 17.17724 | 1.684666 | 0.015538 | 0.000177 | 0 | 0 | 0.359546 | 0 | 0.009895 | 1.001048 | 9.983802 |
| Puerto Rico | 0.020409 | 0.623404 | 2.133821 | 7.104462 | 18.49559 | 34.80595 | 43.33342 | 32.66745 | 14.78293 | 6.573738 | 1.687517 | 0.11202 | 0.02563 | 0.073684 | 0.000149 | 0 | 0.018505 | 10.61977 |
| Qatar | 0.008792 | 0.063106 | 0.151849 | 0.526724 | 1.634616 | 3.726973 | 7.454427 | 11.16085 | 13.29924 | 13.04399 | 9.277157 | 4.855998 | 2.690582 | 1.114778 | 0.841072 | 1.089323 | 6.265376 | 2.335908 |
| Republic of Korea | 0.091874 | 1.82303 | 4.22679 | 10.20634 | 25.01216 | 48.6777 | 52.11883 | 26.20194 | 6.93373 | 1.434261 | 0.04067 | 0 | 0 | 0.315657 | 0 | 0.005044 | 0.465969 | 14.14375 |
| Republic of Moldova | 0.054202 | 2.239732 | 10.23618 | 39.92175 | 98.47331 | 175.3799 | 189.6696 | 76.29688 | 1.980276 | 0 | 0 | 0 | 0 | 0.004012 | 0 | 0 | 0 | 43.58806 |
| Romania | 0.054396 | 1.786501 | 5.920002 | 18.91113 | 46.15991 | 91.25437 | 125.776 | 83.4856 | 11.01451 | 0 | 0 | 0 | 0 | 0.026386 | 0 | 0 | 0 | 28.59289 |
| Russian Federation | 0.052859 | 2.238399 | 10.24723 | 29.36682 | 40.5413 | 33.38769 | 18.17399 | 3.182713 | 0.072812 | 0.000358 | 0.000455 | 0 | 0 | 0.121978 | 0 | 0.020327 | 0.469977 | 10.37689 |
| Rwanda | 0.036464 | 0.640375 | 1.635376 | 4.268239 | 8.578097 | 12.76181 | 17.83959 | 21.69149 | 21.8893 | 14.8158 | 9.228026 | 6.257166 | 1.135542 | 0.866371 | 0.040839 | 0.588513 | 4.814295 | 4.010038 |
| Saint Kitts and Nevis | 0.019119 | 0.564483 | 2.003186 | 7.467036 | 20.27198 | 35.89589 | 45.3101 | 37.54526 | 17.43771 | 5.535394 | 1.263997 | 0.085166 | 0.007596 | 0.202539 | 0 | 0.003305 | 0.802135 | 12.45354 |
| Saint Lucia | 0.020957 | 0.648164 | 2.271872 | 7.135953 | 15.88847 | 24.6961 | 28.02697 | 23.19078 | 12.31208 | 5.092006 | 1.156831 | 0.113161 | 0.094037 | 0.288295 | 0.008701 | 0.007494 | 1.246473 | 8.586494 |
| Saint Vincent and the Grenadines | 0.018133 | 0.565446 | 1.938474 | 6.241551 | 14.9562 | 23.30987 | 23.2921 | 14.26451 | 6.228553 | 2.575738 | 0.536314 | 0.062441 | 0.008281 | 0.014386 | 0.005065 | 0.00079 | 0.005672 | 6.20076 |
| Samoa | 0.019404 | 0.348258 | 1.01036 | 3.093267 | 6.557528 | 9.469184 | 9.052554 | 4.726908 | 2.153532 | 0.847096 | 0.338078 | 0.058782 | 0.040665 | 0.366243 | 0.105002 | 0.110851 | 1.355616 | 1.901034 |
| San Marino | 0.040962 | 1.691042 | 6.312772 | 18.41616 | 42.40401 | 65.84792 | 62.33179 | 31.82684 | 3.37974 | 0 | 0 | 0 | 0 | 0.439646 | 0 | 0.03365 | 0.452205 | 16.32496 |
| Sao Tome and Principe | 0.05548 | 0.848183 | 2.056761 | 4.863427 | 8.235278 | 10.87792 | 16.28042 | 22.89498 | 19.94023 | 9.260285 | 4.399042 | 2.716238 | 2.21238 | 1.206062 | 0.733062 | 1.175469 | 2.864675 | 4.062628 |
| Saudi Arabia | 0.007773 | 0.048187 | 0.110192 | 0.368737 | 1.148125 | 2.726564 | 5.306619 | 7.881244 | 10.05779 | 10.62577 | 7.98804 | 3.500997 | 1.384522 | 0.758966 | 0.646421 | 0.745167 | 0.914594 | 1.840771 |
| Senegal | 0.0343 | 0.518497 | 1.328112 | 3.55105 | 6.292948 | 8.008144 | 12.51297 | 18.20435 | 13.87936 | 3.831717 | 1.890155 | 1.016744 | 0.876881 | 0.534917 | 0.253092 | 0.376089 | 1.604266 | 2.550548 |
| Serbia | 0.033474 | 0.986126 | 2.937111 | 8.212435 | 17.98193 | 29.23865 | 32.47588 | 20.80601 | 5.108525 | 0.172437 | 0.00062 | 0 | 0 | 0.020504 | 0 | 0 | 0.057256 | 7.825025 |
| Seychelles | 0.022076 | 0.414808 | 1.461052 | 5.99684 | 15.03931 | 21.45498 | 19.57139 | 12.80176 | 8.331248 | 5.297578 | 2.895501 | 1.224869 | 0.721595 | 0.483497 | 0.151451 | 0.199012 | 1.236835 | 6.710365 |
| Sierra Leone | 0.030712 | 0.485275 | 1.257119 | 3.245013 | 5.464474 | 6.796562 | 10.83395 | 16.36444 | 14.10858 | 5.197304 | 2.539365 | 1.631342 | 1.431077 | 0.815491 | 0.552802 | 0.679153 | 1.90596 | 2.241935 |
| Singapore | 0.017276 | 0.282327 | 0.654392 | 1.386818 | 2.78478 | 4.233893 | 5.143128 | 3.85902 | 1.414734 | 0.142539 | 0.003081 | 0 | 0 | 0.004331 | 0 | 0 | 0.004696 | 1.670763 |
| Slovakia | 0.056664 | 1.819784 | 6.365614 | 22.56836 | 51.14436 | 67.28361 | 49.62944 | 14.05491 | 0.315846 | 0 | 0 | 0 | 0 | 0.004791 | 0 | 0 | 0 | 16.50468 |
| Slovenia | 0.041609 | 1.251271 | 4.045642 | 13.00674 | 31.93558 | 58.05606 | 65.34047 | 37.74477 | 4.795841 | 0.004776 | 0 | 0 | 0 | 0.005393 | 0 | 0 | 0 | 15.60908 |
| Solomon Islands | 0.029596 | 0.704286 | 2.250488 | 6.11721 | 10.53699 | 10.35193 | 5.129374 | 1.180361 | 0.398557 | 0.140256 | 0.046346 | 0.008285 | 0.00548 | 0.143221 | 0.003889 | 0.06234 | 2.057754 | 2.180343 |
| Somalia | 0.028534 | 0.459995 | 1.106744 | 2.71338 | 5.235963 | 7.602374 | 11.20644 | 14.26542 | 15.07781 | 10.661 | 6.993307 | 4.893736 | 1.869753 | 0.980592 | 0.386063 | 1.107348 | 4.659173 | 2.003802 |
| South Africa | 0.015189 | 0.25086 | 0.595844 | 1.61406 | 3.6155 | 6.25391 | 8.420826 | 7.708 | 4.742591 | 2.314016 | 1.260874 | 0.811841 | 0.880335 | 1.081085 | 0.801329 | 1.247274 | 1.75723 | 2.044406 |
| South Sudan | 0.02465 | 0.381142 | 0.935835 | 2.307894 | 4.615016 | 7.029377 | 10.07425 | 12.23996 | 11.832 | 7.943246 | 5.024243 | 3.576546 | 0.910035 | 1.002715 | 0.087709 | 0.771915 | 3.785409 | 2.040662 |
| Spain | 0.04133 | 1.415843 | 3.969569 | 9.154321 | 20.35817 | 41.50203 | 51.5265 | 32.5645 | 7.237214 | 0.207496 | 0.000776 | 0 | 0 | 0.987757 | 0 | 0.014988 | 2.801064 | 13.18233 |
| Sri Lanka | 0.020534 | 0.356569 | 1.129474 | 4.180551 | 10.4694 | 15.71146 | 16.92228 | 13.68882 | 10.1498 | 6.575972 | 3.303885 | 1.473763 | 1.27782 | 0.502443 | 0.603849 | 0.173475 | 0.296314 | 5.232079 |
| Sudan | 0.004987 | 0.028846 | 0.060304 | 0.186932 | 0.540887 | 1.19052 | 2.219016 | 3.11024 | 3.609854 | 3.505886 | 2.498063 | 1.497568 | 0.845817 | 0.56538 | 0.516192 | 0.472647 | 0.654068 | 0.484505 |
| Suriname | 0.01995 | 0.598542 | 2.043424 | 6.862425 | 16.48746 | 27.40782 | 38.05414 | 37.37254 | 22.03095 | 8.339348 | 1.709064 | 0.083133 | 0.00642 | 0.156285 | 0 | 0.036872 | 0.263931 | 10.01511 |
| Sweden | 0.026187 | 0.974142 | 2.73926 | 6.641419 | 14.78616 | 25.29931 | 29.9965 | 22.0798 | 6.332946 | 0.197016 | 0.016488 | 0 | 0.000636 | 0.381715 | 0.020645 | 0.244714 | 0.757613 | 6.982269 |
| Switzerland | 0.028932 | 1.108048 | 3.464355 | 8.319507 | 17.2295 | 28.84714 | 33.4821 | 23.25836 | 6.017439 | 0.018838 | 0 | 0 | 0 | 0.133555 | 0 | 0.005437 | 0.155996 | 8.692245 |
| Syrian Arab Republic | 0.00733 | 0.04325 | 0.094581 | 0.29557 | 0.905062 | 2.102144 | 3.928283 | 5.504392 | 6.229215 | 5.482501 | 3.710769 | 2.118905 | 1.47263 | 0.626958 | 0.804344 | 0.235029 | 0.44332 | 1.468582 |
| Taiwan (Province of China) | 0.011982 | 0.348249 | 1.615361 | 8.20747 | 17.1209 | 17.3746 | 13.13655 | 9.148085 | 7.157821 | 5.766587 | 2.997469 | 0.353426 | 0.060607 | 1.551394 | 0 | 0 | 4.186542 | 6.451926 |
| Tajikistan | 0.092532 | 2.187887 | 5.861434 | 14.86441 | 27.82168 | 40.67325 | 62.98062 | 89.58077 | 82.74351 | 25.55771 | 0.205796 | 0 | 0 | 0.82364 | 0 | 0.042239 | 4.176348 | 16.1961 |
| Thailand | 0.022875 | 0.464931 | 1.828047 | 7.705826 | 16.44654 | 18.11875 | 14.10517 | 9.710442 | 7.340938 | 4.407828 | 1.781922 | 0.520099 | 0.139052 | 0.059345 | 0.037384 | 0.02128 | 0.040264 | 6.272295 |
| Timor-Leste | 0.022462 | 0.376244 | 1.122809 | 3.804323 | 9.155965 | 14.55 | 17.29775 | 15.49847 | 10.47239 | 5.176326 | 2.058034 | 0.660295 | 0.492112 | 0.487433 | 0.341271 | 0.467346 | 1.07362 | 3.074109 |
| Togo | 0.033068 | 0.51734 | 1.351649 | 3.589461 | 6.508815 | 8.330075 | 12.52021 | 18.67816 | 14.3437 | 4.534674 | 2.545816 | 1.834685 | 1.379354 | 0.649435 | 0.424864 | 0.554007 | 1.651132 | 2.876957 |
| Tokelau | 0.015527 | 0.238945 | 0.710019 | 1.676934 | 4.709775 | 5.1868 | 5.978752 | 3.315781 | 1.64744 | 0.661111 | 0.377391 | 0.069001 | 0.059032 | 0.330594 | 0.086613 | 0.095023 | 0.851317 | 1.33337 |
| Tonga | 0.018921 | 0.334382 | 1.031981 | 3.596129 | 8.535611 | 12.65714 | 12.88465 | 7.508457 | 3.012509 | 1.372425 | 0.854182 | 0.422039 | 0.145593 | 0.550388 | 0.213862 | 0.342855 | 1.028758 | 2.613414 |
| Trinidad and Tobago | 0.017339 | 0.498988 | 1.623926 | 4.8864 | 11.24502 | 17.74665 | 19.14749 | 13.24035 | 5.252561 | 1.116897 | 0.176335 | 0.027104 | 0.001874 | 0.039612 | 0.000608 | 6.04E-05 | 0.020683 | 5.190204 |
| Tunisia | 0.007368 | 0.049353 | 0.119023 | 0.388725 | 1.126265 | 2.39594 | 4.28513 | 6.120972 | 7.126607 | 6.538904 | 4.580644 | 2.656775 | 1.431497 | 0.525982 | 0.584475 | 0.320761 | 0.483485 | 1.88784 |
| Turkey | 0.007391 | 0.046663 | 0.104074 | 0.314127 | 0.853088 | 1.803249 | 3.300996 | 4.517739 | 5.049296 | 4.551316 | 2.918716 | 1.237935 | 0.525034 | 0.287334 | 0.152369 | 0.188797 | 0.675238 | 1.327541 |
| Turkmenistan | 0.195363 | 5.039266 | 13.6264 | 32.95799 | 47.67016 | 49.74649 | 53.28051 | 42.90244 | 17.17975 | 0.624209 | 0 | 0 | 0 | 0.157343 | 0 | 0 | 0.283421 | 16.77467 |
| Tuvalu | 0.017308 | 0.316122 | 0.938313 | 2.856128 | 5.854925 | 7.893476 | 7.094744 | 3.594397 | 1.835369 | 0.844746 | 0.402043 | 0.068907 | 0.050202 | 0.262501 | 0.089491 | 0.116565 | 1.042156 | 1.771264 |
| Uganda | 0.028473 | 0.516175 | 1.302318 | 3.285486 | 6.357514 | 9.392192 | 13.60757 | 16.69228 | 16.79528 | 12.64059 | 8.445292 | 5.427144 | 1.271963 | 0.995889 | 0.156137 | 0.903855 | 4.134683 | 2.344815 |
| Ukraine | 0.085281 | 3.772282 | 15.49857 | 37.43264 | 41.40629 | 21.3276 | 4.858644 | 0.258257 | 0.002252 | 0 | 5.76E-06 | 0 | 0 | 0.025319 | 0 | 0.01076 | 0.077608 | 9.646607 |
| United Arab Emirates | 0.006414 | 0.039601 | 0.09592 | 0.325494 | 0.852408 | 2.003921 | 4.090118 | 6.334128 | 6.666397 | 4.098053 | 1.40433 | 0.665778 | 0.430661 | 0.297201 | 0.141347 | 0.279716 | 1.76503 | 1.64403 |
| United Kingdom | 0.032095 | 1.878037 | 7.824848 | 23.53704 | 48.57525 | 60.24198 | 39.21476 | 7.735061 | 0.056155 | 0 | 0 | 0 | 0 | 0.113839 | 0 | 0.001653 | 0.304422 | 12.52911 |
| United Republic of Tanzania | 0.026019 | 0.467858 | 1.229188 | 3.169467 | 5.914257 | 8.525571 | 11.90249 | 14.81126 | 15.32028 | 10.47655 | 6.133415 | 3.870267 | 0.980582 | 0.870325 | 0.099073 | 0.608048 | 4.063711 | 2.491659 |
| United States of America | 0.009189 | 0.410121 | 1.82665 | 6.23308 | 14.7276 | 22.60424 | 24.43428 | 17.43521 | 5.458825 | 0.285217 | 0.005005 | 1.81E-06 | 1.14E-06 | 0.047203 | 8.37E-05 | 0.018185 | 0.053713 | 5.958907 |
| United States Virgin Islands | 0.022325 | 0.660795 | 2.341522 | 8.434968 | 20.97357 | 32.9375 | 36.0165 | 28.94053 | 16.14576 | 7.376978 | 2.388678 | 0.272144 | 0.035168 | 0.107708 | 0.00974 | 0.09605 | 0.209199 | 10.18548 |
| Uruguay | 0.015347 | 0.356672 | 1.017244 | 2.74474 | 6.649341 | 13.11741 | 22.21428 | 25.68318 | 14.84833 | 1.734032 | 0.124321 | 0.002904 | 0.001873 | 0.105431 | 0.001481 | 0.004306 | 0.136607 | 5.382025 |
| Uzbekistan | 0.13544 | 3.694664 | 10.23164 | 23.06183 | 38.34886 | 54.47657 | 89.01101 | 112.9438 | 74.13182 | 6.907504 | 0 | 0 | 0 | 0.420344 | 0 | 0 | 12.70074 | 22.82733 |
| Vanuatu | 0.023842 | 0.48088 | 1.426969 | 4.504249 | 10.29207 | 15.17028 | 13.26179 | 5.15349 | 1.968779 | 0.672979 | 0.254044 | 0.04839 | 0.080953 | 0.35214 | 0.225592 | 0.383903 | 1.342232 | 2.677714 |
| Venezuela (Bolivarian Republic of) | 0.014652 | 0.628797 | 2.340021 | 6.874804 | 16.26712 | 26.12527 | 29.31009 | 21.68073 | 9.701585 | 3.307119 | 0.2951 | 0 | 0 | 0.157736 | 0 | 0 | 0.214567 | 7.545434 |
| Viet Nam | 0.026169 | 0.489939 | 1.482778 | 4.897029 | 12.58837 | 19.96061 | 20.66872 | 16.07779 | 12.44383 | 7.680261 | 4.461661 | 2.527952 | 1.174442 | 0.269217 | 0.254628 | 0.170091 | 0.224508 | 6.685884 |
| Yemen | 0.006269 | 0.036541 | 0.08189 | 0.29269 | 0.947302 | 2.121223 | 3.720932 | 4.738082 | 4.8826 | 3.997679 | 2.281466 | 1.03883 | 0.535406 | 0.36153 | 0.321838 | 0.292551 | 0.496585 | 0.652799 |
| Zambia | 0.0396 | 0.713334 | 1.769975 | 4.247774 | 8.195932 | 12.49234 | 18.01021 | 21.64014 | 22.4843 | 16.16297 | 9.698005 | 6.285984 | 1.282866 | 0.739637 | 0.025282 | 0.486704 | 4.869674 | 3.446479 |
| Zimbabwe | 0.029401 | 0.48662 | 1.166119 | 2.594954 | 5.067529 | 8.533161 | 12.02127 | 12.15633 | 9.126106 | 4.765431 | 1.671558 | 0.642812 | 0.533218 | 0.594379 | 0.411325 | 0.644127 | 1.223172 | 2.170483 |

**Supplementary table 7. Age distribution of deaths rate for cirrhosis and other chronic liver diseases due to alcohol use in different countries in 2019.**

| **2019Death rate** | **15 to 19** | **20 to 24** | **25 to 29** | **30 to 34** | **35 to 39** | **40 to 44** | **45 to 49** | **50 to 54** | **55 to 59** | **60 to 64** | **65 to 69** | **70 to 74** | **75 to 79** | **80 plus** | **80-84** | **85-89** | **90-94** | **all ages** |
| --- | --- | --- | --- | --- | --- | --- | --- | --- | --- | --- | --- | --- | --- | --- | --- | --- | --- | --- |
| Afghanistan | 0.003746 | 0.027227 | 0.064219 | 0.162224 | 0.375107 | 0.850927 | 1.628694 | 3.220817 | 5.192074 | 7.302325 | 8.662223 | 10.2788 | 11.03185 | 14.57761 | 12.89116 | 16.534 | 20.63343 | 0.585421 |
| Albania | 0.005389 | 0.125319 | 0.351825 | 0.78266 | 1.262842 | 2.024531 | 2.991905 | 4.944517 | 6.88407 | 11.42775 | 15.30861 | 20.67507 | 27.03923 | 35.65616 | 33.79009 | 36.95137 | 40.71827 | 5.452721 |
| Algeria | 0.002067 | 0.015443 | 0.032475 | 0.083065 | 0.177314 | 0.341545 | 0.62003 | 1.193537 | 1.99525 | 3.089003 | 4.010279 | 4.994964 | 6.273499 | 11.59298 | 8.360788 | 17.01015 | 25.4397 | 0.699896 |
| American Samoa | 0.006696 | 0.088108 | 0.265167 | 0.522537 | 1.392068 | 2.188573 | 3.925402 | 5.143217 | 7.703375 | 8.400961 | 8.686027 | 8.428807 | 10.23248 | 12.54632 | 9.750938 | 14.3782 | 17.19601 | 2.074079 |
| Andorra | 0.000502 | 0.032335 | 0.20471 | 0.838392 | 2.07918 | 4.050451 | 6.399743 | 9.700404 | 12.91898 | 15.52565 | 17.05636 | 18.07451 | 19.20796 | 26.59765 | 21.43558 | 26.09956 | 32.74736 | 7.047626 |
| Angola | 0.01384 | 0.198207 | 0.537059 | 1.141135 | 2.598526 | 5.845234 | 9.913502 | 15.93188 | 25.28247 | 41.37576 | 50.3504 | 50.47468 | 56.65159 | 54.10951 | 53.868 | 54.24447 | 52.08376 | 3.651294 |
| Antigua and Barbuda | 0.00192 | 0.065139 | 0.243867 | 0.568034 | 1.629925 | 2.719298 | 5.131815 | 9.352063 | 15.36451 | 22.59261 | 23.11 | 26.94812 | 30.32353 | 40.94355 | 40.15992 | 39.37009 | 44.61418 | 6.087937 |
| Argentina | 0.002369 | 0.050699 | 0.134892 | 0.429053 | 1.264966 | 2.935934 | 5.938441 | 10.76912 | 16.89616 | 21.51293 | 25.73563 | 26.24876 | 24.16474 | 26.01295 | 23.54972 | 26.71564 | 30.73204 | 5.80634 |
| Armenia | 0.004914 | 0.09061 | 0.316291 | 1.043533 | 3.04324 | 7.546135 | 12.60149 | 17.42426 | 22.41551 | 29.23439 | 43.45319 | 59.7171 | 84.76273 | 151.72 | 126.7925 | 199.1609 | 179.5851 | 15.54872 |
| Australia | 0.000217 | 0.00522 | 0.026517 | 0.112926 | 0.349833 | 0.877537 | 1.578007 | 2.838613 | 4.217012 | 4.49771 | 4.269416 | 4.430126 | 3.901268 | 5.808797 | 4.424297 | 5.742206 | 8.512422 | 1.644747 |
| Austria | 0.000534 | 0.02884 | 0.166855 | 0.748956 | 1.766601 | 4.039651 | 7.821026 | 13.25893 | 20.73555 | 25.81708 | 27.50917 | 26.32506 | 22.39678 | 24.52964 | 23.35983 | 25.19076 | 26.73292 | 9.928508 |
| Azerbaijan | 0.013911 | 0.30959 | 0.745049 | 1.800488 | 3.776033 | 6.907608 | 10.83516 | 17.6743 | 28.86807 | 44.46336 | 66.42446 | 85.05745 | 106.4458 | 161.1449 | 138.1571 | 233.1575 | 252.1456 | 12.10475 |
| Bahamas | 0.002973 | 0.104207 | 0.404801 | 1.382298 | 3.546705 | 6.341936 | 9.662205 | 16.35328 | 18.6488 | 23.85158 | 26.46353 | 25.7843 | 26.81404 | 29.59131 | 28.27208 | 29.19408 | 34.01814 | 6.882745 |
| Bahrain | 0.001355 | 0.015973 | 0.028846 | 0.072578 | 0.157767 | 0.317659 | 0.797541 | 1.516098 | 2.702836 | 3.350803 | 5.312789 | 7.274481 | 10.43903 | 16.92565 | 14.67223 | 21.17722 | 29.69993 | 0.790742 |
| Bangladesh | 0.005299 | 0.109217 | 0.398766 | 0.875098 | 1.951201 | 3.163523 | 5.607033 | 11.04273 | 16.3611 | 16.28636 | 18.04929 | 21.23319 | 17.98941 | 48.30725 | 39.26278 | 59.21317 | 71.71882 | 4.028188 |
| Barbados | 0.002165 | 0.051643 | 0.199778 | 0.765971 | 1.5554 | 3.173705 | 5.795354 | 8.304687 | 12.96229 | 17.75819 | 19.88881 | 22.41386 | 21.64579 | 27.22107 | 25.01798 | 28.7988 | 31.4429 | 6.958627 |
| Belarus | 0.002021 | 0.11803 | 0.886662 | 3.220273 | 6.986767 | 10.24074 | 13.76352 | 18.6527 | 26.45586 | 31.35597 | 26.91979 | 18.81777 | 14.09767 | 14.45922 | 13.45935 | 14.68562 | 17.23456 | 11.08475 |
| Belgium | 0.000651 | 0.032735 | 0.153927 | 0.535324 | 1.409439 | 3.530591 | 6.669316 | 12.03438 | 17.54024 | 21.98407 | 23.05569 | 21.82974 | 21.97638 | 30.38088 | 24.13498 | 30.90046 | 41.09654 | 8.960834 |
| Belize | 0.005204 | 0.145222 | 0.566335 | 1.84948 | 3.793485 | 7.173214 | 13.72369 | 21.34437 | 31.67186 | 35.5953 | 36.37977 | 39.92967 | 38.52493 | 45.69561 | 44.78925 | 44.5049 | 47.15873 | 6.532003 |
| Benin | 0.011837 | 0.159859 | 0.546556 | 1.47083 | 3.061854 | 6.55781 | 9.03132 | 15.14327 | 25.50933 | 42.80666 | 43.35499 | 42.80571 | 43.99209 | 53.69204 | 53.16479 | 51.76266 | 55.58519 | 3.569124 |
| Bermuda | 0.001402 | 0.032155 | 0.086361 | 0.262154 | 0.740339 | 1.80805 | 3.58249 | 5.76296 | 8.141778 | 10.22249 | 12.47809 | 13.56698 | 15.67676 | 19.76399 | 18.17399 | 20.51796 | 22.33146 | 5.292591 |
| Bhutan | 0.016515 | 0.262613 | 0.92117 | 2.101146 | 4.099883 | 6.72595 | 10.15411 | 14.94565 | 20.22537 | 25.89763 | 31.58058 | 38.6946 | 43.93668 | 55.79743 | 52.26368 | 60.76173 | 61.20284 | 6.024347 |
| Bolivia (Plurinational State of) | 0.004445 | 0.117595 | 0.386162 | 1.188079 | 3.075704 | 6.991898 | 13.97539 | 25.70563 | 41.84578 | 63.19382 | 80.17528 | 91.57868 | 99.49048 | 109.7591 | 105.0366 | 116.7475 | 112.9711 | 11.29277 |
| Bosnia and Herzegovina | 0.002959 | 0.07355 | 0.205453 | 0.569305 | 1.394652 | 3.097717 | 5.810753 | 9.512186 | 15.03567 | 21.05872 | 23.34757 | 25.42228 | 28.82556 | 24.25609 | 26.18485 | 21.05757 | 19.78778 | 8.478056 |
| Botswana | 0.005914 | 0.100461 | 0.338016 | 1.238941 | 2.79105 | 5.729989 | 9.802539 | 16.25348 | 23.29705 | 27.74922 | 31.88888 | 31.59471 | 31.40311 | 33.57812 | 33.1425 | 34.19764 | 34.93487 | 4.545366 |
| Brazil | 0.004237 | 0.151637 | 0.594779 | 1.809067 | 4.144372 | 7.758573 | 12.45269 | 18.56562 | 24.03228 | 27.24897 | 28.74431 | 29.37657 | 28.44081 | 31.713 | 29.24397 | 32.29016 | 34.65112 | 8.142447 |
| Brunei Darussalam | 0.001181 | 0.021542 | 0.081242 | 0.336127 | 0.94654 | 2.342685 | 4.074134 | 5.202067 | 7.16641 | 8.534446 | 9.759973 | 10.11862 | 9.945305 | 13.35217 | 11.71523 | 16.68505 | 19.75815 | 1.909808 |
| Bulgaria | 0.009451 | 0.255992 | 0.803353 | 2.789393 | 6.286644 | 11.32994 | 20.10916 | 30.7358 | 41.0958 | 46.84885 | 45.54399 | 39.64036 | 36.74496 | 27.08306 | 28.15166 | 25.89636 | 23.91026 | 19.38776 |
| Burkina Faso | 0.011026 | 0.13001 | 0.404438 | 0.972937 | 1.95672 | 4.447457 | 5.589499 | 10.47709 | 16.88613 | 36.89355 | 42.45059 | 40.24519 | 47.47402 | 59.32416 | 57.72464 | 58.80185 | 62.80561 | 3.056541 |
| Burundi | 0.009425 | 0.133659 | 0.41806 | 0.856201 | 2.228499 | 6.18339 | 10.22807 | 17.23773 | 25.68105 | 42.46767 | 54.2691 | 50.98924 | 53.52614 | 50.6623 | 54.36536 | 43.01863 | 41.23761 | 3.793215 |
| Cabo Verde | 0.009507 | 0.130049 | 0.364627 | 1.011312 | 2.176674 | 5.321176 | 8.126375 | 13.6628 | 23.29571 | 33.44143 | 30.84892 | 32.26217 | 31.40759 | 46.97796 | 41.56722 | 49.98669 | 53.53856 | 5.60438 |
| Cambodia | 0.021819 | 0.435928 | 1.13073 | 3.272701 | 7.014273 | 12.86421 | 19.80346 | 28.55928 | 40.68844 | 52.01818 | 61.03971 | 67.71158 | 70.94409 | 79.41116 | 76.7561 | 84.16479 | 81.14878 | 10.58116 |
| Cameroon | 0.011923 | 0.154575 | 0.484819 | 1.309005 | 2.818711 | 6.075023 | 8.020385 | 13.34228 | 22.16185 | 38.31048 | 38.84061 | 37.63324 | 37.60362 | 45.33287 | 45.05837 | 43.96739 | 47.22351 | 3.408398 |
| Canada | 0.000429 | 0.018046 | 0.091284 | 0.289184 | 0.693681 | 1.528311 | 2.947839 | 5.36359 | 8.720161 | 11.54163 | 12.15378 | 12.55286 | 12.75952 | 16.384 | 13.74136 | 15.89684 | 20.7862 | 4.571599 |
| Central African Republic | 0.013273 | 0.147531 | 0.357708 | 0.785489 | 2.149254 | 6.13143 | 11.44193 | 19.17022 | 30.46114 | 45.74186 | 48.83804 | 42.70694 | 41.39184 | 32.2541 | 33.32796 | 30.09863 | 27.61365 | 4.067792 |
| Chad | 0.015562 | 0.156228 | 0.48775 | 1.3098 | 2.97029 | 7.079968 | 10.17885 | 17.20356 | 29.80786 | 50.39281 | 51.13396 | 48.44058 | 44.86112 | 51.35979 | 51.6798 | 49.11587 | 51.37253 | 3.521561 |
| Chile | 0.001419 | 0.031758 | 0.163328 | 0.625569 | 1.947127 | 4.476166 | 8.420965 | 14.54896 | 22.01133 | 28.94816 | 35.70575 | 35.54782 | 33.99851 | 30.96479 | 30.31444 | 30.71826 | 32.3606 | 8.867955 |
| China | 0.000594 | 0.017606 | 0.060801 | 0.240715 | 0.693771 | 1.381893 | 1.994015 | 2.994486 | 4.20145 | 5.341647 | 6.272315 | 7.476577 | 8.5911 | 10.39887 | 9.62312 | 12.32024 | 9.764869 | 2.14774 |
| Colombia | 0.001449 | 0.044077 | 0.119805 | 0.360108 | 0.711709 | 1.372091 | 2.248807 | 3.895277 | 6.567501 | 10.2761 | 13.34239 | 16.7455 | 19.96073 | 25.55957 | 22.92948 | 25.39339 | 29.04136 | 3.084041 |
| Comoros | 0.005597 | 0.090447 | 0.320403 | 0.64368 | 1.597598 | 4.042521 | 6.689579 | 11.44663 | 17.37138 | 28.81632 | 39.06592 | 38.41309 | 44.25342 | 44.58273 | 46.66938 | 39.42718 | 38.38866 | 4.68872 |
| Congo | 0.00919 | 0.136221 | 0.402161 | 0.89942 | 2.178733 | 5.143243 | 8.855164 | 14.0038 | 22.02279 | 34.20614 | 41.2646 | 40.98651 | 47.34897 | 45.61959 | 42.93877 | 49.28099 | 52.37607 | 4.127203 |
| Cook Islands | 0.001764 | 0.024329 | 0.136734 | 0.259823 | 0.951097 | 1.526866 | 3.318054 | 4.598891 | 7.178538 | 8.300905 | 8.138981 | 7.504237 | 8.455779 | 9.749981 | 7.049362 | 11.93827 | 13.55483 | 2.681568 |
| Costa Rica | 0.002348 | 0.088742 | 0.2831 | 0.989759 | 2.34887 | 4.828904 | 8.424112 | 14.32336 | 22.06991 | 29.67079 | 33.78432 | 36.6764 | 40.45772 | 42.28104 | 38.62195 | 43.67014 | 44.52153 | 7.886191 |
| Côte d'Ivoire | 0.012561 | 0.154343 | 0.497185 | 1.337929 | 2.91206 | 6.403126 | 8.695572 | 14.0401 | 23.54336 | 40.29828 | 41.97864 | 39.78991 | 40.51984 | 50.52778 | 50.62761 | 48.6811 | 51.10865 | 3.664427 |
| Croatia | 0.001093 | 0.03344 | 0.186554 | 0.810496 | 2.182931 | 4.90953 | 9.3886 | 16.3659 | 24.68004 | 32.10169 | 35.56523 | 36.57048 | 33.2061 | 31.91665 | 33.13329 | 30.56831 | 28.96891 | 13.40718 |
| Cuba | 0.001852 | 0.038075 | 0.144174 | 0.512087 | 1.487647 | 4.138907 | 7.70923 | 12.59846 | 17.53626 | 21.0063 | 21.05651 | 22.39096 | 21.48525 | 20.24019 | 20.07001 | 19.9479 | 20.36139 | 7.939976 |
| Cyprus | 0.000328 | 0.016958 | 0.110517 | 0.37037 | 0.961697 | 1.663521 | 2.808797 | 5.143273 | 7.817003 | 11.15174 | 13.90067 | 16.54384 | 22.18131 | 38.88211 | 33.00019 | 44.0531 | 68.05987 | 4.793209 |
| Czechia | 0.00188 | 0.06349 | 0.31079 | 1.19189 | 2.898217 | 5.518091 | 10.55096 | 16.73515 | 24.82519 | 30.04184 | 29.29633 | 24.90645 | 21.09226 | 17.99657 | 17.59005 | 18.4921 | 17.33073 | 10.915 |
| Democratic People's Republic of Korea | 0.002254 | 0.055777 | 0.190334 | 0.623622 | 1.528403 | 3.042554 | 5.352777 | 8.36722 | 11.74997 | 13.89456 | 15.06023 | 14.86073 | 14.29669 | 13.89239 | 13.86899 | 13.71063 | 13.96602 | 4.263005 |
| Democratic Republic of the Congo | 0.01067 | 0.149045 | 0.428984 | 0.947012 | 2.283349 | 5.27959 | 8.68638 | 14.27555 | 21.7517 | 32.99728 | 36.72376 | 34.79491 | 36.22073 | 32.78955 | 32.40755 | 32.75269 | 32.70732 | 3.141329 |
| Denmark | 0.000328 | 0.02022 | 0.13158 | 0.494587 | 1.278708 | 3.159376 | 6.643347 | 12.12219 | 18.48694 | 22.78245 | 19.96931 | 18.24427 | 14.30822 | 15.67689 | 13.72475 | 15.0684 | 19.59725 | 7.591684 |
| Djibouti | 0.007766 | 0.11299 | 0.341596 | 0.663186 | 1.65428 | 4.375694 | 7.634134 | 13.56085 | 21.07538 | 34.80557 | 46.52306 | 46.91188 | 50.11852 | 49.44355 | 51.95669 | 43.5855 | 41.26292 | 4.16295 |
| Dominica | 0.002953 | 0.069928 | 0.248673 | 0.775112 | 2.206834 | 3.868378 | 6.383651 | 10.99767 | 16.11746 | 19.91339 | 23.80256 | 25.97653 | 25.85214 | 29.10012 | 27.89707 | 28.72283 | 34.15615 | 6.601237 |
| Dominican Republic | 0.009274 | 0.243687 | 0.696675 | 2.048968 | 4.49896 | 7.793494 | 13.43843 | 23.12775 | 31.25675 | 46.99355 | 50.83335 | 66.41339 | 65.69319 | 109.9391 | 84.68685 | 134.5017 | 150.6112 | 10.58594 |
| Ecuador | 0.00387 | 0.108044 | 0.396096 | 1.201546 | 2.866233 | 5.328808 | 9.757967 | 16.6054 | 26.75774 | 38.8704 | 47.51691 | 55.32708 | 62.25353 | 92.20812 | 78.1684 | 100.9275 | 132.058 | 8.72946 |
| Egypt | 0.004574 | 0.038532 | 0.094807 | 0.259893 | 0.577446 | 1.225059 | 2.700497 | 6.507861 | 12.13704 | 19.87255 | 28.88321 | 43.20036 | 57.1707 | 94.00146 | 77.3274 | 110.6303 | 150.6627 | 3.629044 |
| El Salvador | 0.004098 | 0.165703 | 0.851001 | 3.10469 | 5.946969 | 9.254796 | 13.13978 | 19.20213 | 26.32162 | 33.28652 | 36.28623 | 42.04168 | 44.34414 | 53.06996 | 48.46255 | 56.34345 | 55.41215 | 8.627715 |
| Equatorial Guinea | 0.006545 | 0.090364 | 0.249439 | 0.518633 | 1.181828 | 2.602435 | 4.476058 | 7.110726 | 11.15788 | 18.00496 | 23.68028 | 23.79061 | 29.33279 | 29.79099 | 28.42115 | 31.81903 | 30.86279 | 1.547496 |
| Eritrea | 0.01111 | 0.185682 | 0.573356 | 1.172689 | 3.163752 | 8.353108 | 14.22752 | 22.664 | 33.56737 | 50.35133 | 65.35766 | 58.2144 | 66.56418 | 65.53865 | 67.88409 | 60.70123 | 52.37924 | 5.035848 |
| Estonia | 0.002214 | 0.082488 | 0.500204 | 2.251838 | 4.790266 | 9.666291 | 15.15394 | 21.21216 | 27.3599 | 28.17319 | 24.56376 | 16.61274 | 11.36254 | 8.777501 | 8.37785 | 8.643399 | 10.06213 | 10.32104 |
| Eswatini | 0.007806 | 0.121448 | 0.414679 | 1.335507 | 3.02357 | 6.430192 | 11.57761 | 18.60126 | 26.041 | 29.8748 | 33.80722 | 31.62318 | 32.51669 | 35.3271 | 34.46009 | 36.62759 | 38.86009 | 4.142074 |
| Ethiopia | 0.007265 | 0.092607 | 0.278744 | 0.596554 | 1.455383 | 3.691308 | 6.562606 | 11.95432 | 19.34793 | 32.91701 | 48.14751 | 51.34279 | 60.09391 | 59.67249 | 61.43319 | 55.71255 | 52.49005 | 3.11161 |
| Fiji | 0.005712 | 0.074599 | 0.232029 | 0.395423 | 1.203823 | 1.924828 | 3.413471 | 4.686015 | 6.664151 | 8.362405 | 9.135484 | 8.670939 | 10.65968 | 12.1169 | 10.47087 | 14.83054 | 15.14505 | 1.897096 |
| Finland | 0.000328 | 0.03565 | 0.217616 | 0.895801 | 2.459644 | 5.461726 | 11.46297 | 18.96989 | 25.71717 | 29.69735 | 25.42638 | 18.12713 | 12.88893 | 10.2768 | 9.932402 | 9.939953 | 10.80347 | 9.96813 |
| France | 0.000418 | 0.021796 | 0.120156 | 0.446391 | 1.314805 | 3.298659 | 6.563933 | 10.94228 | 15.46773 | 18.93501 | 19.19962 | 17.76841 | 17.3327 | 20.09295 | 16.8468 | 19.39519 | 24.86031 | 7.412145 |
| Gabon | 0.010793 | 0.153611 | 0.425048 | 0.936952 | 2.282737 | 5.049078 | 8.559535 | 13.54367 | 22.33647 | 35.02156 | 45.23605 | 43.12515 | 47.2722 | 45.58142 | 42.56168 | 49.64467 | 48.91267 | 4.994031 |
| Gambia | 0.011651 | 0.151374 | 0.462003 | 1.248537 | 2.728271 | 6.010319 | 8.506453 | 14.67816 | 26.17077 | 44.82647 | 45.50815 | 43.73154 | 42.9092 | 51.61778 | 49.80915 | 53.58644 | 56.09664 | 3.870043 |
| Georgia | 0.008611 | 0.22565 | 0.806053 | 3.061743 | 9.465456 | 17.14035 | 27.67445 | 35.3271 | 39.12985 | 39.80253 | 44.62248 | 50.55779 | 55.75506 | 46.35304 | 51.70942 | 42.18473 | 37.88798 | 18.76259 |
| Germany | 0.000588 | 0.036675 | 0.228698 | 0.85835 | 2.282634 | 5.073459 | 9.084084 | 15.27065 | 21.08565 | 26.11512 | 26.91743 | 26.65614 | 25.87754 | 29.1313 | 26.79973 | 30.39934 | 34.69004 | 11.59542 |
| Ghana | 0.012982 | 0.195919 | 0.566591 | 1.583231 | 3.247381 | 6.72549 | 8.884714 | 15.71401 | 25.88927 | 45.17111 | 48.09192 | 45.71137 | 48.23321 | 58.68289 | 58.27743 | 58.78178 | 56.43978 | 5.013223 |
| Greece | 0.000592 | 0.030537 | 0.1126 | 0.343642 | 0.860897 | 1.984808 | 4.072233 | 6.528359 | 8.805173 | 10.72733 | 12.51563 | 14.01504 | 16.23073 | 19.77906 | 16.87642 | 20.37506 | 25.54503 | 5.840603 |
| Greenland | 0.000439 | 0.021968 | 0.189886 | 0.756918 | 1.915369 | 4.000473 | 6.648013 | 10.37596 | 13.74971 | 14.13415 | 11.89659 | 9.821779 | 7.573284 | 6.682885 | 6.374845 | 6.509829 | 8.426075 | 4.538201 |
| Grenada | 0.002956 | 0.080048 | 0.250352 | 1.066591 | 2.85833 | 5.285477 | 9.457778 | 14.96572 | 21.94658 | 30.43154 | 37.23258 | 37.22577 | 36.23434 | 30.15785 | 32.30655 | 27.42101 | 25.94349 | 8.036564 |
| Guam | 0.009145 | 0.10519 | 0.32065 | 0.854636 | 2.452613 | 3.029103 | 4.168756 | 7.220193 | 10.12098 | 10.57036 | 11.03988 | 9.526505 | 12.15363 | 18.44367 | 12.58575 | 18.61502 | 25.78231 | 3.334613 |
| Guatemala | 0.009394 | 0.390375 | 1.892408 | 5.734493 | 12.28375 | 19.80233 | 25.81319 | 38.84083 | 47.82006 | 56.57879 | 63.72469 | 60.70178 | 57.57529 | 81.11227 | 71.93556 | 95.74316 | 119.3734 | 10.77001 |
| Guinea | 0.015125 | 0.165075 | 0.525319 | 1.385512 | 2.957827 | 6.577466 | 9.1261 | 14.89268 | 26.46159 | 45.17155 | 45.13962 | 44.12194 | 41.90818 | 50.46975 | 50.69529 | 47.7824 | 51.09925 | 4.01882 |
| Guinea-Bissau | 0.019789 | 0.237105 | 0.729489 | 1.9902 | 4.583565 | 10.98867 | 15.62292 | 24.39532 | 42.80782 | 67.63338 | 66.51785 | 60.80906 | 56.18137 | 64.15738 | 63.45742 | 64.36117 | 64.15833 | 5.493939 |
| Guyana | 0.007315 | 0.250534 | 0.898653 | 3.943327 | 9.111125 | 17.82773 | 27.8176 | 40.12404 | 54.06267 | 67.67604 | 64.85387 | 63.98543 | 58.00413 | 56.63243 | 54.68164 | 52.85727 | 67.6502 | 14.22838 |
| Haiti | 0.004532 | 0.106694 | 0.38256 | 1.324115 | 3.555766 | 8.265767 | 14.8909 | 24.74895 | 35.6122 | 46.54144 | 50.37559 | 54.02765 | 51.78358 | 52.26283 | 52.49774 | 51.61369 | 51.73886 | 6.562728 |
| Honduras | 0.006497 | 0.163389 | 0.496137 | 1.533624 | 3.978751 | 8.689872 | 16.75322 | 30.23812 | 48.91691 | 67.3297 | 77.04552 | 88.98359 | 105.1296 | 121.4983 | 110.2931 | 133.496 | 151.5352 | 10.25943 |
| Hungary | 0.001767 | 0.066324 | 0.362899 | 1.436463 | 3.508549 | 7.049843 | 16.0507 | 28.49079 | 37.78428 | 47.08188 | 47.30737 | 37.6844 | 29.27541 | 16.60666 | 18.75682 | 14.86798 | 12.3206 | 16.56886 |
| Iceland | 0.000108 | 0.00708 | 0.045234 | 0.193422 | 0.571672 | 1.291826 | 2.240839 | 3.752415 | 5.544643 | 6.700985 | 7.009504 | 6.761869 | 6.30485 | 5.989671 | 5.92111 | 5.641908 | 6.281073 | 2.229327 |
| India | 0.009922 | 0.207196 | 0.688356 | 1.9273 | 3.949651 | 5.624679 | 8.200398 | 10.87166 | 14.63546 | 17.32019 | 19.86577 | 21.73787 | 22.68833 | 27.95195 | 26.60965 | 27.86712 | 34.88834 | 4.424172 |
| Indonesia | 0.011105 | 0.191515 | 0.39072 | 1.079991 | 2.145189 | 3.724419 | 6.057186 | 9.600897 | 15.45295 | 24.30403 | 31.19539 | 38.65369 | 45.967 | 60.01305 | 54.66727 | 67.2142 | 75.6094 | 5.492337 |
| Iran (Islamic Republic of) | 0.001398 | 0.010309 | 0.022805 | 0.06106 | 0.127914 | 0.243759 | 0.457036 | 0.907894 | 1.642331 | 2.476038 | 3.124165 | 3.841483 | 5.006308 | 7.486929 | 6.028446 | 8.178165 | 11.30709 | 0.574784 |
| Iraq | 0.00172 | 0.011548 | 0.023024 | 0.062756 | 0.142288 | 0.374226 | 0.777411 | 1.467491 | 2.654104 | 3.513159 | 4.359116 | 5.054547 | 5.437741 | 6.663048 | 5.860239 | 6.990845 | 9.117235 | 0.474771 |
| Ireland | 0.000631 | 0.027755 | 0.128973 | 0.423416 | 1.15753 | 2.419488 | 4.03065 | 6.730379 | 8.708252 | 9.888252 | 10.71725 | 9.654518 | 11.30409 | 13.13173 | 11.06234 | 13.56685 | 17.51183 | 3.643974 |
| Israel | 0.000604 | 0.017282 | 0.096876 | 0.293495 | 0.602279 | 1.219357 | 2.324043 | 4.328766 | 5.529754 | 7.684284 | 10.21846 | 12.91154 | 17.87786 | 26.45831 | 21.24948 | 27.19849 | 34.50384 | 2.973158 |
| Italy | 0.000348 | 0.010185 | 0.042332 | 0.160494 | 0.45475 | 1.102834 | 2.205009 | 3.639594 | 5.18741 | 6.800066 | 8.66516 | 10.48041 | 12.03564 | 14.93776 | 13.93277 | 15.49402 | 15.87424 | 4.090618 |
| Jamaica | 0.002024 | 0.049959 | 0.161143 | 0.442915 | 0.826354 | 1.476973 | 2.575994 | 4.535317 | 7.419766 | 9.318411 | 10.34636 | 14.06478 | 14.76675 | 16.45855 | 16.10542 | 16.24113 | 15.90456 | 2.590838 |
| Japan | 0.000354 | 0.006859 | 0.019767 | 0.087486 | 0.277991 | 0.753482 | 1.537249 | 2.820333 | 4.269666 | 5.464902 | 6.029206 | 6.738028 | 7.973206 | 12.5769 | 9.897826 | 12.56953 | 15.36339 | 3.436933 |
| Jordan | 0.001069 | 0.007883 | 0.018068 | 0.053529 | 0.11193 | 0.250918 | 0.49417 | 1.032892 | 1.695055 | 2.808723 | 4.284376 | 4.658336 | 5.949227 | 8.667203 | 7.351658 | 9.733645 | 13.04327 | 0.420075 |
| Kazakhstan | 0.00805 | 0.289623 | 1.376634 | 4.749012 | 10.47002 | 16.61192 | 21.64267 | 30.08867 | 44.01761 | 57.68829 | 73.5465 | 78.74948 | 76.09353 | 80.81213 | 81.00294 | 76.72417 | 86.38123 | 15.52612 |
| Kenya | 0.007995 | 0.164249 | 0.548317 | 1.208727 | 3.051689 | 7.57043 | 13.77287 | 24.12706 | 38.03653 | 62.80314 | 86.83953 | 89.53427 | 105.896 | 106.2926 | 110.173 | 99.7961 | 85.27059 | 6.721917 |
| Kiribati | 0.016914 | 0.20348 | 0.741624 | 1.688859 | 4.58747 | 6.583683 | 11.52896 | 13.79738 | 19.95088 | 21.04098 | 21.07139 | 19.8353 | 25.18788 | 27.58024 | 25.59194 | 31.52235 | 30.66685 | 3.977069 |
| Kuwait | 0.000548 | 0.004147 | 0.006807 | 0.024318 | 0.071124 | 0.178396 | 0.431616 | 0.97051 | 1.61839 | 2.494068 | 2.740237 | 3.510471 | 3.895044 | 6.776551 | 5.150495 | 7.286506 | 8.668641 | 0.365725 |
| Kyrgyzstan | 0.014464 | 0.450897 | 1.735058 | 6.004208 | 13.69686 | 22.79641 | 29.40523 | 39.18187 | 53.56222 | 61.08926 | 73.29897 | 67.66157 | 61.94937 | 56.61467 | 52.7025 | 60.00494 | 66.07757 | 13.47354 |
| Lao People's Democratic Republic | 0.004935 | 0.100652 | 0.286124 | 0.999652 | 2.403181 | 4.930306 | 8.12306 | 12.73498 | 18.47394 | 23.82047 | 24.67622 | 25.27729 | 23.38688 | 26.14392 | 24.74243 | 27.70058 | 29.3665 | 3.739054 |
| Latvia | 0.002142 | 0.071928 | 0.510491 | 2.362058 | 5.433621 | 9.946089 | 14.01666 | 18.67678 | 20.62844 | 22.30712 | 19.4705 | 16.07703 | 13.50724 | 10.21411 | 9.854641 | 10.45721 | 10.38807 | 9.515338 |
| Lebanon | 0.002209 | 0.015703 | 0.030238 | 0.069173 | 0.140249 | 0.268251 | 0.503962 | 0.997326 | 1.784744 | 2.762672 | 3.777565 | 4.933389 | 5.767967 | 8.966481 | 7.325621 | 9.96824 | 13.3488 | 0.795973 |
| Lesotho | 0.007057 | 0.112622 | 0.336442 | 1.058689 | 2.492982 | 5.682262 | 10.91035 | 18.70988 | 27.49938 | 32.31183 | 34.48478 | 31.15516 | 30.34425 | 31.03283 | 30.75887 | 31.00296 | 32.85552 | 4.825595 |
| Liberia | 0.015759 | 0.199315 | 0.610098 | 1.528164 | 3.279444 | 7.070336 | 9.823032 | 16.43232 | 27.34171 | 47.01882 | 48.79154 | 50.32982 | 53.56041 | 71.96095 | 68.21397 | 72.73434 | 80.28457 | 4.547275 |
| Libya | 0.001843 | 0.014601 | 0.030351 | 0.087083 | 0.203176 | 0.399062 | 0.797626 | 1.48375 | 2.540332 | 3.904421 | 5.16019 | 6.240341 | 7.853053 | 10.24101 | 8.388092 | 10.7051 | 13.35958 | 0.777817 |
| Lithuania | 0.001691 | 0.076149 | 1.015295 | 4.194131 | 10.45082 | 17.36808 | 22.30387 | 27.16721 | 32.19063 | 35.25698 | 31.55511 | 19.30214 | 14.07342 | 10.74445 | 10.50442 | 10.69456 | 11.45677 | 14.30978 |
| Luxembourg | 0.000766 | 0.041936 | 0.239903 | 0.87277 | 1.964086 | 3.769005 | 6.293286 | 10.29164 | 15.6987 | 18.70724 | 20.63583 | 20.33256 | 20.65186 | 24.26853 | 21.35758 | 24.05101 | 29.33375 | 7.016 |
| Madagascar | 0.008831 | 0.140709 | 0.449096 | 0.881274 | 2.076656 | 5.310985 | 8.962952 | 14.53554 | 22.42088 | 35.65449 | 48.00919 | 45.7388 | 49.9873 | 51.03262 | 52.4631 | 46.66478 | 46.71896 | 3.598341 |
| Malawi | 0.013395 | 0.201455 | 0.574704 | 1.091679 | 2.653282 | 6.656903 | 11.23667 | 18.5326 | 27.83444 | 44.82313 | 61.98101 | 60.50045 | 68.61671 | 63.47801 | 68.21783 | 57.57634 | 46.47293 | 4.359142 |
| Malaysia | 0.001967 | 0.029981 | 0.084342 | 0.35076 | 0.937199 | 1.820753 | 3.31653 | 5.352308 | 9.024323 | 12.39681 | 14.48069 | 18.2509 | 17.49025 | 20.33543 | 18.31306 | 21.35986 | 26.73196 | 2.668439 |
| Maldives | 0.001077 | 0.019686 | 0.083534 | 0.345551 | 0.833857 | 1.403946 | 2.195774 | 3.149617 | 4.839963 | 6.374344 | 7.587855 | 9.130412 | 11.99176 | 18.03279 | 15.9873 | 20.01263 | 22.02045 | 1.278257 |
| Mali | 0.010972 | 0.125754 | 0.430389 | 1.167808 | 2.566349 | 5.761769 | 7.893871 | 13.05263 | 22.13945 | 37.64836 | 38.36509 | 38.1086 | 36.32609 | 43.33963 | 42.84387 | 41.99192 | 44.89584 | 3.120831 |
| Malta | 0.000513 | 0.022913 | 0.153063 | 0.531222 | 1.311718 | 2.395321 | 3.885026 | 5.309126 | 6.973855 | 8.778914 | 10.18986 | 10.63896 | 10.18359 | 11.27175 | 10.90886 | 10.8881 | 12.07769 | 4.206481 |
| Marshall Islands | 0.010333 | 0.161147 | 0.601208 | 1.277922 | 3.345018 | 5.213286 | 8.85486 | 11.38802 | 16.08756 | 17.45944 | 18.38584 | 17.08621 | 19.48326 | 23.31624 | 20.3735 | 27.84235 | 31.79838 | 3.432903 |
| Mauritania | 0.007571 | 0.099513 | 0.338647 | 0.927017 | 1.916471 | 4.105488 | 5.652333 | 9.537964 | 16.61694 | 29.3216 | 30.54915 | 31.93364 | 34.54175 | 45.49768 | 42.73193 | 46.57445 | 50.25087 | 3.27617 |
| Mauritius | 0.003087 | 0.074647 | 0.255344 | 0.952241 | 2.266274 | 4.103916 | 6.319926 | 9.546581 | 10.666 | 14.53176 | 13.95503 | 11.95041 | 11.95605 | 13.17901 | 10.79697 | 13.70406 | 17.4311 | 4.855913 |
| Mexico | 0.002803 | 0.137176 | 0.718669 | 2.810579 | 7.155624 | 13.31561 | 19.88112 | 30.58906 | 43.03391 | 52.87859 | 61.3921 | 64.09581 | 68.91817 | 77.03643 | 72.40959 | 79.47701 | 84.3907 | 13.5322 |
| Micronesia (Federated States of) | 0.010427 | 0.168412 | 0.591225 | 1.218491 | 2.85137 | 4.6055 | 9.030379 | 12.04482 | 17.35315 | 18.0601 | 17.99369 | 16.85838 | 19.32267 | 21.59501 | 18.6108 | 25.09476 | 28.99791 | 3.79949 |
| Monaco | 0.000768 | 0.042539 | 0.250564 | 1.042894 | 2.373243 | 4.533688 | 6.809162 | 9.972393 | 13.03429 | 15.17265 | 16.32707 | 17.12545 | 17.79614 | 22.6546 | 19.574 | 21.91464 | 27.18119 | 8.598883 |
| Mongolia | 0.014205 | 0.554231 | 2.142588 | 5.966674 | 12.53466 | 21.79484 | 31.3811 | 45.17246 | 66.36911 | 94.91374 | 120.2359 | 139.1721 | 171.8897 | 287.8717 | 240.5775 | 381.479 | 451.2397 | 20.01594 |
| Montenegro | 0.001003 | 0.038848 | 0.163343 | 0.524312 | 1.277625 | 2.212206 | 3.831937 | 6.38373 | 9.312816 | 11.49606 | 11.94006 | 9.721308 | 9.199424 | 8.624176 | 7.89971 | 9.425763 | 10.82676 | 3.872435 |
| Morocco | 0.002007 | 0.01296 | 0.025856 | 0.066321 | 0.157746 | 0.353753 | 0.751302 | 1.560347 | 2.805688 | 4.290541 | 5.601491 | 7.288306 | 8.145668 | 12.64651 | 10.51886 | 13.86215 | 17.6373 | 0.969883 |
| Mozambique | 0.011603 | 0.153441 | 0.375808 | 0.409561 | 0.879817 | 2.262864 | 4.935583 | 9.608633 | 10.7589 | 13.51944 | 25.70343 | 26.06327 | 27.4953 | 37.5388 | 38.59424 | 32.90302 | 34.52398 | 1.698569 |
| Myanmar | 0.009145 | 0.265056 | 0.960397 | 3.074009 | 7.042644 | 13.29372 | 21.00125 | 21.29768 | 27.32158 | 27.14391 | 23.98773 | 20.01877 | 14.83114 | 12.74857 | 13.09489 | 11.88985 | 12.52752 | 7.51759 |
| Namibia | 0.005447 | 0.08468 | 0.266657 | 0.88938 | 2.089889 | 4.257784 | 7.90178 | 13.95771 | 20.49851 | 24.7937 | 28.50027 | 29.86358 | 31.4657 | 34.35152 | 33.88612 | 34.86623 | 34.92825 | 3.773082 |
| Nauru | 0.011222 | 0.180451 | 0.646184 | 1.364789 | 3.62778 | 5.741851 | 9.841806 | 12.47734 | 17.14204 | 18.04201 | 17.12617 | 16.49306 | 20.34289 | 31.23745 | 28.03325 | 37.17082 | 33.58247 | 2.707765 |
| Nepal | 0.025472 | 0.478935 | 1.374301 | 3.063365 | 6.640097 | 11.18052 | 17.92343 | 26.55338 | 36.94743 | 48.06897 | 58.74336 | 73.45123 | 83.06569 | 102.4909 | 95.89932 | 108.885 | 126.0887 | 10.45283 |
| Netherlands | 0.00033 | 0.013987 | 0.085521 | 0.241547 | 0.498151 | 1.145794 | 2.319915 | 4.278664 | 6.612689 | 8.566145 | 9.452636 | 10.27384 | 12.25933 | 25.10204 | 16.36067 | 25.74821 | 42.34098 | 4.335243 |
| New Zealand | 0.000269 | 0.005249 | 0.016862 | 0.050668 | 0.141241 | 0.362892 | 0.778528 | 1.370481 | 2.191076 | 2.872662 | 3.106759 | 3.096868 | 3.282026 | 5.499678 | 3.7542 | 5.470901 | 8.866619 | 1.116471 |
| Nicaragua | 0.004783 | 0.152707 | 0.557637 | 1.619876 | 3.895359 | 8.193531 | 15.05361 | 26.3041 | 40.3742 | 52.00383 | 65.60901 | 76.9913 | 82.86806 | 89.68112 | 89.19081 | 90.29385 | 92.01233 | 9.532247 |
| Niger | 0.011991 | 0.132978 | 0.461922 | 1.260103 | 2.740975 | 6.028931 | 8.182142 | 13.87551 | 24.56866 | 42.16641 | 41.63828 | 41.46943 | 41.18012 | 48.12874 | 47.75211 | 45.93423 | 50.62446 | 2.942191 |
| Nigeria | 0.010374 | 0.139912 | 0.471474 | 1.351713 | 2.973828 | 6.050511 | 8.66411 | 14.71969 | 25.16617 | 44.03516 | 49.47418 | 53.88668 | 58.85914 | 77.30107 | 73.40238 | 78.56143 | 85.39094 | 4.08435 |
| Niue | 0.009875 | 0.117044 | 0.344372 | 0.638205 | 1.867069 | 3.114217 | 5.546411 | 8.053223 | 12.56941 | 14.72694 | 14.7154 | 13.99402 | 17.02252 | 20.7046 | 17.34467 | 23.04642 | 26.13307 | 4.720235 |
| North Macedonia | 0.003439 | 0.063802 | 0.241239 | 0.585114 | 1.409605 | 2.926568 | 5.444361 | 8.899037 | 14.79479 | 18.85053 | 22.28918 | 22.89415 | 27.76577 | 24.49011 | 24.00167 | 25.53771 | 25.29675 | 6.81825 |
| Northern Mariana Islands | 0.00947 | 0.12272 | 0.341061 | 0.798669 | 2.327985 | 2.879581 | 7.616888 | 7.598012 | 14.52785 | 14.60797 | 17.19924 | 16.25192 | 22.14886 | 28.51955 | 21.42424 | 33.84395 | 43.14015 | 5.618673 |
| Norway | 0.000334 | 0.012908 | 0.047672 | 0.156642 | 0.475199 | 1.147929 | 2.072103 | 3.578918 | 5.208579 | 7.096825 | 7.864725 | 8.177259 | 8.590532 | 12.18213 | 9.021367 | 11.92536 | 16.53864 | 2.820967 |
| Oman | 0.00168 | 0.012361 | 0.020223 | 0.062834 | 0.172582 | 0.300007 | 0.675639 | 1.559383 | 3.115671 | 4.573637 | 7.254716 | 8.832493 | 9.841873 | 13.7931 | 13.51806 | 12.84744 | 16.84575 | 0.468419 |
| Pakistan | 0.008878 | 0.140005 | 0.377374 | 0.83756 | 1.652317 | 2.662572 | 4.30173 | 6.595999 | 9.820891 | 13.51103 | 16.98204 | 21.47987 | 23.7155 | 31.29171 | 29.18054 | 32.66987 | 39.28508 | 1.969626 |
| Palau | 0.00802 | 0.126184 | 0.450528 | 0.870156 | 2.512701 | 3.861374 | 6.375594 | 8.058615 | 10.95389 | 12.19298 | 12.24829 | 12.20726 | 14.3017 | 18.80427 | 14.77811 | 23.18157 | 25.39574 | 4.479713 |
| Palestine | 0.001592 | 0.009979 | 0.021595 | 0.048332 | 0.12983 | 0.234593 | 0.556518 | 1.323897 | 2.689048 | 4.205629 | 6.052342 | 8.174351 | 9.570706 | 12.86595 | 11.8027 | 13.30376 | 17.28133 | 0.516742 |
| Panama | 0.003469 | 0.07584 | 0.186569 | 0.580769 | 1.050961 | 2.316885 | 4.06866 | 7.693082 | 12.98701 | 17.46021 | 21.8177 | 25.852 | 29.0859 | 35.65617 | 30.31756 | 36.51884 | 44.43739 | 4.516271 |
| Papua New Guinea | 0.005541 | 0.073771 | 0.262487 | 0.593628 | 1.62566 | 2.747782 | 4.430844 | 5.239464 | 7.073282 | 7.351988 | 7.895411 | 6.829877 | 6.512887 | 6.133318 | 6.177684 | 6.333431 | 5.541289 | 1.262691 |
| Paraguay | 0.004875 | 0.125642 | 0.360157 | 1.051756 | 2.329079 | 5.106163 | 8.614695 | 14.04729 | 18.67957 | 23.90044 | 26.98017 | 28.46662 | 28.02831 | 33.31593 | 29.53488 | 35.91874 | 35.41836 | 5.003106 |
| Peru | 0.005516 | 0.137147 | 0.487822 | 1.292594 | 2.845291 | 5.567416 | 9.569412 | 15.92618 | 23.81138 | 33.37394 | 42.31015 | 49.47879 | 55.62271 | 61.96021 | 56.14328 | 65.65569 | 64.70861 | 8.350942 |
| Philippines | 0.002198 | 0.05765 | 0.185059 | 0.691154 | 1.642867 | 3.08997 | 5.31874 | 7.982669 | 11.54612 | 14.34908 | 16.4915 | 16.74283 | 16.05906 | 17.45712 | 16.23161 | 18.11382 | 21.22052 | 2.684661 |
| Poland | 0.001528 | 0.078651 | 0.537988 | 1.968824 | 4.656017 | 8.54288 | 13.31376 | 18.90233 | 24.75562 | 27.06181 | 26.81406 | 21.08439 | 19.93461 | 17.61458 | 16.22394 | 17.62049 | 20.84331 | 10.72219 |
| Portugal | 0.000821 | 0.041179 | 0.215296 | 0.750818 | 2.107445 | 5.156549 | 9.106262 | 14.0993 | 17.39272 | 18.76496 | 20.39764 | 21.40265 | 20.19849 | 21.37399 | 18.69605 | 21.84303 | 26.40975 | 9.373643 |
| Puerto Rico | 0.001251 | 0.050186 | 0.199729 | 0.820483 | 2.157101 | 4.410816 | 9.224725 | 16.31347 | 22.77442 | 27.66645 | 29.33314 | 31.12648 | 31.73489 | 37.04215 | 34.11712 | 37.41147 | 38.48333 | 12.03598 |
| Qatar | 0.002475 | 0.012422 | 0.017639 | 0.054578 | 0.145336 | 0.318957 | 0.736069 | 1.678167 | 3.42841 | 4.935396 | 7.626299 | 12.46533 | 17.01381 | 44.06694 | 38.28011 | 63.68002 | 70.57447 | 0.514763 |
| Republic of Korea | 0.002688 | 0.045588 | 0.148443 | 0.64834 | 1.694121 | 4.716282 | 8.823535 | 13.53885 | 16.29572 | 18.01129 | 18.93683 | 22.72308 | 27.36505 | 43.90475 | 34.61289 | 48.85557 | 67.62098 | 9.036924 |
| Republic of Moldova | 0.005502 | 0.213323 | 1.07284 | 3.999774 | 11.00142 | 21.58735 | 38.06889 | 58.8348 | 85.50023 | 106.7206 | 113.1536 | 102.7213 | 84.44629 | 61.15223 | 70.95072 | 49.58979 | 46.81043 | 35.97117 |
| Romania | 0.006495 | 0.215404 | 0.978029 | 2.304364 | 5.280969 | 11.22724 | 21.99932 | 36.7698 | 54.94433 | 65.78182 | 70.70349 | 73.54105 | 69.58355 | 51.95893 | 59.21442 | 46.5578 | 32.19082 | 26.37956 |
| Russian Federation | 0.001723 | 0.128636 | 1.229984 | 5.228008 | 11.89187 | 16.13797 | 19.69254 | 24.48276 | 29.46684 | 33.97462 | 29.51366 | 24.56033 | 19.94629 | 14.5651 | 15.59033 | 12.96535 | 12.84934 | 13.36525 |
| Rwanda | 0.010007 | 0.151061 | 0.464723 | 0.997499 | 2.502453 | 6.018475 | 9.705199 | 16.15475 | 23.62763 | 37.60529 | 51.47791 | 48.64734 | 54.88556 | 59.26945 | 62.01415 | 52.99802 | 50.99941 | 4.507311 |
| Saint Kitts and Nevis | 0.002845 | 0.061028 | 0.175191 | 0.528964 | 1.591499 | 4.195133 | 9.451404 | 17.93288 | 28.00533 | 34.58551 | 39.80534 | 40.85153 | 41.45861 | 36.64521 | 39.27965 | 35.21433 | 29.10806 | 9.472822 |
| Saint Lucia | 0.002908 | 0.095184 | 0.353763 | 1.194208 | 2.817186 | 5.757224 | 9.323142 | 14.77804 | 19.89671 | 25.14624 | 26.34687 | 29.73961 | 29.2813 | 39.88952 | 32.66317 | 43.50189 | 57.9517 | 8.087499 |
| Saint Vincent and the Grenadines | 0.002933 | 0.066945 | 0.285436 | 0.926551 | 2.647289 | 5.644633 | 12.19646 | 16.52868 | 21.1312 | 21.44164 | 24.37354 | 26.40644 | 28.97381 | 30.48459 | 28.35918 | 33.45597 | 35.18575 | 7.568875 |
| Samoa | 0.007619 | 0.110858 | 0.360244 | 0.747638 | 2.01142 | 3.336115 | 5.983182 | 7.987594 | 11.52445 | 12.56816 | 13.36372 | 12.62813 | 15.09593 | 18.95095 | 15.56492 | 21.89341 | 24.59147 | 2.528988 |
| San Marino | 0.001005 | 0.043769 | 0.221001 | 0.729727 | 1.847968 | 3.686453 | 6.218621 | 9.928343 | 13.95918 | 18.41073 | 21.99134 | 26.88852 | 33.60876 | 58.03128 | 43.48041 | 59.83827 | 80.20674 | 10.24789 |
| Sao Tome and Principe | 0.019529 | 0.272539 | 0.69479 | 1.617534 | 3.547499 | 7.449868 | 12.16141 | 20.07198 | 35.32072 | 57.42041 | 63.73057 | 66.13845 | 66.66002 | 86.5186 | 78.55236 | 94.30336 | 105.3822 | 6.621945 |
| Saudi Arabia | 0.000927 | 0.009425 | 0.020512 | 0.055194 | 0.151362 | 0.364229 | 0.814837 | 1.730255 | 3.15078 | 4.94024 | 6.96985 | 12.12701 | 15.80826 | 24.17698 | 19.30159 | 26.09352 | 34.55278 | 0.69289 |
| Senegal | 0.010185 | 0.138915 | 0.443074 | 1.11151 | 2.369506 | 5.082319 | 6.856507 | 11.58897 | 20.15659 | 35.98759 | 36.04516 | 35.22228 | 34.96672 | 42.97732 | 42.20596 | 42.47965 | 43.93368 | 3.649934 |
| Serbia | 0.001274 | 0.040012 | 0.171153 | 0.631906 | 1.778745 | 3.286683 | 6.381109 | 10.42011 | 15.83809 | 18.99756 | 23.40337 | 20.66407 | 21.14992 | 21.76867 | 21.47243 | 22.0005 | 24.01728 | 7.778431 |
| Seychelles | 0.002533 | 0.080033 | 0.331474 | 1.599793 | 3.553204 | 7.900407 | 12.33805 | 16.40025 | 20.90733 | 23.7347 | 25.74754 | 22.74449 | 25.75245 | 30.70727 | 27.51093 | 32.25738 | 37.79816 | 7.570072 |
| Sierra Leone | 0.01122 | 0.126762 | 0.439538 | 1.169957 | 2.501035 | 5.650615 | 7.427404 | 12.60015 | 21.00247 | 36.18413 | 36.30306 | 34.03766 | 33.81268 | 41.52211 | 41.31553 | 39.60517 | 43.24371 | 3.278585 |
| Singapore | 0.000491 | 0.005223 | 0.009398 | 0.026369 | 0.071148 | 0.224869 | 0.5193 | 0.917694 | 1.614488 | 2.052197 | 2.477468 | 2.928454 | 3.09278 | 4.50432 | 3.952157 | 4.660061 | 5.311748 | 0.738085 |
| Slovakia | 0.002643 | 0.095233 | 0.591573 | 2.046449 | 5.245345 | 10.73263 | 17.35732 | 26.94658 | 35.7039 | 45.09936 | 41.08721 | 35.03421 | 29.00099 | 23.29989 | 24.80188 | 22.43632 | 18.85347 | 15.36791 |
| Slovenia | 0.000689 | 0.037775 | 0.22214 | 0.859382 | 2.493977 | 4.704456 | 9.232525 | 16.80725 | 24.55174 | 32.36656 | 36.81153 | 34.97324 | 36.97961 | 24.51129 | 28.9141 | 20.58134 | 18.5482 | 13.21421 |
| Solomon Islands | 0.010735 | 0.223607 | 0.853058 | 2.255738 | 5.630804 | 9.19331 | 14.55727 | 16.42113 | 20.58288 | 19.12187 | 18.47785 | 15.35805 | 14.95834 | 13.74433 | 13.42482 | 14.56323 | 14.78509 | 3.656875 |
| Somalia | 0.01265 | 0.145976 | 0.40707 | 0.847197 | 2.244418 | 6.574997 | 11.42552 | 19.14682 | 28.67765 | 44.872 | 58.77039 | 53.67213 | 56.8149 | 53.78778 | 57.10007 | 46.3804 | 41.9486 | 3.483758 |
| South Africa | 0.001441 | 0.042304 | 0.192282 | 0.644113 | 1.288892 | 2.05635 | 3.122539 | 5.745887 | 9.265527 | 12.36112 | 13.86591 | 13.30946 | 13.13057 | 18.18523 | 15.96139 | 19.53634 | 24.49929 | 2.429848 |
| South Sudan | 0.008686 | 0.106716 | 0.328253 | 0.665652 | 1.620975 | 4.132392 | 7.270351 | 13.14761 | 21.58203 | 35.78541 | 51.64988 | 52.29091 | 59.70073 | 57.83341 | 60.72648 | 50.48296 | 51.5163 | 3.569037 |
| Spain | 0.00088 | 0.03256 | 0.108101 | 0.325453 | 0.828433 | 2.25572 | 5.315198 | 9.494206 | 12.23034 | 14.8443 | 16.53621 | 18.71206 | 21.72278 | 29.24942 | 24.1799 | 29.34493 | 37.43432 | 7.511051 |
| Sri Lanka | 0.002583 | 0.057735 | 0.222826 | 0.792357 | 2.278475 | 4.086726 | 6.37654 | 9.202605 | 11.69257 | 13.61753 | 16.38923 | 18.09254 | 17.63102 | 24.48657 | 20.87882 | 28.17349 | 32.28941 | 4.694293 |
| Sudan | 0.003413 | 0.023872 | 0.050214 | 0.123158 | 0.253017 | 0.522388 | 1.016537 | 2.116492 | 3.6824 | 5.756578 | 7.537994 | 9.802291 | 11.60682 | 15.75832 | 13.98221 | 17.18748 | 19.28736 | 0.676507 |
| Suriname | 0.004956 | 0.117493 | 0.440696 | 1.319907 | 3.655234 | 6.295767 | 10.17026 | 17.77886 | 28.4259 | 33.64175 | 37.71788 | 45.31644 | 51.16445 | 46.94082 | 48.70973 | 45.74311 | 41.31031 | 9.248752 |
| Sweden | 0.000373 | 0.02032 | 0.059769 | 0.158923 | 0.444397 | 1.107588 | 2.654251 | 5.033752 | 8.703246 | 13.23124 | 15.18693 | 14.26764 | 12.93183 | 16.05363 | 13.38921 | 15.56998 | 20.46576 | 4.864481 |
| Switzerland | 0.000285 | 0.01382 | 0.07458 | 0.272112 | 0.725641 | 1.921326 | 3.480153 | 5.955742 | 9.038493 | 12.24525 | 14.59751 | 14.54003 | 15.49098 | 19.34929 | 16.94229 | 18.95169 | 23.45394 | 5.303642 |
| Syrian Arab Republic | 0.003372 | 0.019539 | 0.041451 | 0.093439 | 0.197237 | 0.385984 | 0.771282 | 1.560106 | 2.751449 | 4.60234 | 5.892225 | 7.507579 | 9.113629 | 15.9223 | 12.81347 | 20.44982 | 28.11982 | 1.020576 |
| Taiwan (Province of China) | 0.001042 | 0.02782 | 0.183375 | 1.023747 | 3.235156 | 5.959301 | 8.93948 | 11.0397 | 12.66527 | 12.84938 | 14.52204 | 18.45745 | 21.26418 | 29.69374 | 24.75588 | 31.96391 | 34.23722 | 7.312634 |
| Tajikistan | 0.023121 | 0.566098 | 1.624012 | 4.029537 | 8.015436 | 12.6309 | 16.63349 | 22.74967 | 35.59335 | 51.61052 | 66.48818 | 81.46501 | 90.44803 | 95.08906 | 96.20773 | 99.73298 | 81.9067 | 8.341911 |
| Thailand | 0.002284 | 0.067544 | 0.346091 | 1.94556 | 5.152371 | 8.320538 | 10.92194 | 13.7809 | 16.16292 | 17.95905 | 19.60323 | 20.38326 | 21.41965 | 25.16224 | 23.78639 | 24.59505 | 27.22025 | 8.174573 |
| Timor-Leste | 0.003836 | 0.071428 | 0.194087 | 0.692738 | 1.732203 | 3.634596 | 6.604033 | 11.40201 | 17.08071 | 22.05182 | 23.85762 | 24.1859 | 23.33829 | 25.26845 | 23.68937 | 27.16969 | 29.42451 | 3.17209 |
| Togo | 0.011563 | 0.144785 | 0.451549 | 1.231085 | 2.582919 | 6.049329 | 8.143659 | 13.61419 | 23.15098 | 38.08955 | 37.85469 | 33.92325 | 34.88808 | 42.12751 | 42.59101 | 39.63733 | 41.6172 | 3.791783 |
| Tokelau | 0.007148 | 0.107783 | 0.353958 | 0.579298 | 1.815548 | 2.227413 | 4.441776 | 5.546459 | 8.062516 | 10.17272 | 10.36964 | 10.91689 | 12.6465 | 16.57632 | 13.27445 | 18.75173 | 23.11775 | 2.530375 |
| Tonga | 0.008519 | 0.10621 | 0.2893 | 0.581293 | 2.248766 | 4.077532 | 7.286074 | 10.38253 | 17.18794 | 19.1418 | 18.57211 | 16.96726 | 20.83983 | 25.89644 | 21.34975 | 27.83923 | 30.02606 | 3.670989 |
| Trinidad and Tobago | 0.002772 | 0.086312 | 0.284242 | 0.848657 | 1.959378 | 4.29989 | 6.621789 | 10.80205 | 14.87181 | 17.02459 | 18.2097 | 17.63241 | 18.81338 | 20.56104 | 19.87387 | 19.56635 | 22.56072 | 5.803531 |
| Tunisia | 0.001532 | 0.01262 | 0.030268 | 0.083193 | 0.174236 | 0.340033 | 0.613896 | 1.138865 | 2.034677 | 3.07573 | 4.185184 | 5.339518 | 6.523741 | 10.18182 | 8.658747 | 11.17975 | 13.73027 | 0.955082 |
| Turkey | 0.000796 | 0.006522 | 0.01482 | 0.03744 | 0.092237 | 0.201745 | 0.452565 | 0.94338 | 1.655933 | 2.696497 | 3.533467 | 4.917931 | 5.36094 | 7.170466 | 5.963881 | 7.916699 | 9.426825 | 0.769458 |
| Turkmenistan | 0.044171 | 1.501327 | 4.536548 | 10.6298 | 20.77562 | 31.53274 | 37.76557 | 47.73312 | 60.38315 | 77.52149 | 72.44776 | 79.21121 | 69.59717 | 58.74846 | 61.54187 | 55.627 | 52.73585 | 18.49698 |
| Tuvalu | 0.008807 | 0.146108 | 0.527162 | 1.092369 | 2.863612 | 4.410602 | 7.278698 | 9.27049 | 12.97604 | 14.06753 | 14.65055 | 13.67792 | 16.57468 | 20.32022 | 16.90268 | 23.82245 | 28.31252 | 3.550483 |
| Uganda | 0.008777 | 0.134594 | 0.419364 | 0.817502 | 2.026337 | 5.036692 | 8.445852 | 14.29676 | 21.72662 | 34.47415 | 47.93583 | 46.0763 | 54.72395 | 53.83158 | 57.57041 | 46.77343 | 41.09007 | 2.942544 |
| Ukraine | 0.006179 | 0.353813 | 2.283787 | 8.586786 | 19.47018 | 27.84407 | 34.11847 | 37.89329 | 42.56247 | 39.84164 | 31.32695 | 22.15535 | 15.24831 | 9.635066 | 9.324542 | 9.252534 | 11.67892 | 18.69085 |
| United Arab Emirates | 0.000246 | 0.003962 | 0.010937 | 0.039131 | 0.099141 | 0.275926 | 0.657102 | 1.534031 | 2.69507 | 5.189148 | 6.947995 | 7.061182 | 5.965511 | 7.047909 | 6.179838 | 8.773836 | 10.4199 | 0.48379 |
| United Kingdom | 0.000941 | 0.06181 | 0.569633 | 1.712706 | 3.540043 | 6.515973 | 9.732351 | 14.41022 | 19.29472 | 20.26693 | 18.62195 | 16.27287 | 14.6412 | 17.29251 | 14.68001 | 16.85956 | 23.10006 | 7.881284 |
| United Republic of Tanzania | 0.007558 | 0.117494 | 0.383948 | 0.832135 | 2.028857 | 4.796962 | 7.61456 | 12.65305 | 19.13692 | 32.58145 | 44.36537 | 42.20406 | 46.27776 | 45.24089 | 46.44508 | 41.4279 | 39.86786 | 3.368165 |
| United States of America | 0.000503 | 0.020715 | 0.120234 | 0.447077 | 1.118416 | 2.528081 | 4.832928 | 9.081223 | 13.44041 | 15.60711 | 14.2134 | 13.73621 | 13.18419 | 13.5346 | 13.01302 | 13.17778 | 14.23375 | 5.287239 |
| United States Virgin Islands | 0.00264 | 0.060067 | 0.205433 | 0.741753 | 2.458832 | 6.194303 | 12.48626 | 19.80193 | 26.73982 | 31.28455 | 35.48035 | 39.83466 | 41.04232 | 44.76882 | 43.86609 | 44.71868 | 47.65091 | 13.89008 |
| Uruguay | 0.001027 | 0.024266 | 0.067993 | 0.194646 | 0.538119 | 1.302539 | 2.673706 | 5.091692 | 9.091269 | 12.28282 | 15.28222 | 14.98847 | 14.48255 | 15.46378 | 13.13925 | 15.83555 | 19.25873 | 4.022093 |
| Uzbekistan | 0.028087 | 0.897717 | 3.270009 | 8.107425 | 13.9565 | 19.09617 | 25.35963 | 38.09696 | 56.50118 | 88.14586 | 92.66123 | 108.0376 | 113.0505 | 100.5757 | 102.8547 | 91.93747 | 85.50526 | 15.10906 |
| Vanuatu | 0.010142 | 0.14827 | 0.545526 | 1.188835 | 3.304864 | 5.353021 | 9.285241 | 11.87475 | 16.73441 | 17.84826 | 18.77443 | 16.84785 | 19.20117 | 22.28183 | 20.0451 | 26.63496 | 30.72881 | 3.134947 |
| Venezuela (Bolivarian Republic of) | 0.002631 | 0.078495 | 0.224993 | 0.656032 | 1.803041 | 4.060639 | 8.467018 | 14.88737 | 21.85333 | 27.35956 | 30.33483 | 31.59901 | 32.33985 | 28.41846 | 30.49344 | 24.86287 | 25.80355 | 6.795967 |
| Viet Nam | 0.004947 | 0.081052 | 0.238607 | 0.649219 | 1.885332 | 4.667871 | 8.641807 | 13.08352 | 16.24948 | 17.71373 | 22.65892 | 24.49499 | 29.02974 | 39.33008 | 34.94491 | 44.85037 | 44.97785 | 5.553101 |
| Yemen | 0.00204 | 0.016496 | 0.03997 | 0.106372 | 0.247036 | 0.580594 | 1.110264 | 2.299866 | 3.822553 | 5.883708 | 7.455613 | 9.042394 | 9.913963 | 12.96817 | 11.77919 | 14.12128 | 16.45309 | 0.603517 |
| Zambia | 0.017881 | 0.281828 | 0.793188 | 1.580356 | 3.628972 | 9.509333 | 16.41416 | 26.92221 | 40.05746 | 58.01766 | 78.71176 | 74.51587 | 84.07269 | 86.91734 | 86.50532 | 86.26249 | 82.8383 | 5.518356 |
| Zimbabwe | 0.009046 | 0.122021 | 0.361957 | 1.043505 | 2.051719 | 4.189855 | 8.078804 | 13.43609 | 18.76831 | 24.7154 | 32.00362 | 36.62988 | 38.3129 | 39.03525 | 39.81825 | 38.08567 | 36.32598 | 3.184789 |

**Supplementary table 8. Age distribution of DALYs rate for cirrhosis and other chronic liver diseases due to alcohol use in different countries in 2019.**

| **2019 DALYs rate** | **15 to 19** | **20 to 24** | **25 to 29** | **30 to 34** | **35 to 39** | **40 to 44** | **45 to 49** | **50 to 54** | **55 to 59** | **60 to 64** | **65 to 69** | **70 to 74** | **75 to 79** | **80 plus** | **80-84** | **85-89** | **90-94** | **all ages** |
| --- | --- | --- | --- | --- | --- | --- | --- | --- | --- | --- | --- | --- | --- | --- | --- | --- | --- | --- |
| Afghanistan | 0.2743 | 3.994827 | 3.994827 | 9.270514 | 19.52641 | 40.08217 | 68.79267 | 120.4636 | 169.7214 | 204.9762 | 204.2927 | 198.4581 | 168.9431 | 150.3499 | 151.7858 | 148.5011 | 143.8915 | 18.08651 |
| Albania | 0.410544 | 22.40231 | 22.40231 | 45.50806 | 66.72167 | 96.58394 | 127.7383 | 186.9579 | 226.8715 | 322.5763 | 361.9144 | 400.1361 | 413.4824 | 366.2243 | 399.1965 | 333.9112 | 282.0247 | 128.8926 |
| Algeria | 0.155051 | 2.043725 | 2.043725 | 4.777715 | 9.288384 | 16.19581 | 26.36681 | 44.922 | 65.51189 | 87.03059 | 94.97157 | 96.4634 | 95.89284 | 116.1607 | 97.23718 | 151.5747 | 179.2314 | 17.91956 |
| American Samoa | 0.4849 | 16.46454 | 16.46454 | 29.87973 | 72.52913 | 103.372 | 166.2779 | 193.3487 | 252.7969 | 236.7536 | 205.5685 | 163.3939 | 156.8275 | 121.8233 | 115.0598 | 129.1901 | 119.4162 | 65.64271 |
| Andorra | 0.050991 | 13.32568 | 13.32568 | 48.62087 | 109.0729 | 191.7505 | 271.6246 | 364.8189 | 425.1702 | 438.7773 | 404.603 | 350.2434 | 293.7984 | 240.2097 | 251.448 | 232.9706 | 226.4716 | 198.4746 |
| Angola | 1.006656 | 33.47895 | 33.47895 | 65.27771 | 135.3397 | 275.0626 | 417.9114 | 595.281 | 824.8907 | 1158.858 | 1184.919 | 972.7398 | 864.5223 | 571.0359 | 632.5519 | 486.2662 | 360.7808 | 115.1953 |
| Antigua and Barbuda | 0.148295 | 15.35604 | 15.35604 | 32.81668 | 85.40298 | 129.1915 | 217.769 | 351.3771 | 504.0348 | 637.7556 | 547.3963 | 521.344 | 463.0194 | 413.8903 | 472.5441 | 354.1964 | 308.6859 | 167.3194 |
| Argentina | 0.178206 | 8.515171 | 8.515171 | 24.68831 | 66.16635 | 138.7576 | 251.2695 | 404.1845 | 554.5373 | 606.5388 | 609.9009 | 508.7468 | 370.2478 | 254.6539 | 276.6917 | 239.4265 | 213.1139 | 155.1846 |
| Armenia | 0.383191 | 20.28247 | 20.28247 | 60.38307 | 159.4297 | 357.4651 | 535.9874 | 657.0271 | 738.4138 | 825.1432 | 1028.441 | 1156.955 | 1297.784 | 1543.736 | 1497.131 | 1796.384 | 1246.767 | 360.7836 |
| Australia | 0.019656 | 1.696016 | 1.696016 | 6.518926 | 18.32798 | 41.57928 | 67.03901 | 106.8095 | 138.5537 | 126.7615 | 100.9257 | 85.64135 | 59.77836 | 53.39265 | 51.92597 | 51.33475 | 58.8843 | 44.08166 |
| Austria | 0.056497 | 11.12407 | 11.12407 | 43.80299 | 93.43044 | 192.3828 | 333.5154 | 500.8485 | 683.9758 | 730.722 | 653.6598 | 511.1761 | 342.6918 | 238.9984 | 273.322 | 225.0045 | 185.2928 | 263.6953 |
| Azerbaijan | 1.024481 | 46.80067 | 46.80067 | 103.5904 | 197.9232 | 327.3354 | 460.1008 | 664.4555 | 948.3238 | 1251.652 | 1571.156 | 1646.437 | 1627.781 | 1729.51 | 1629.062 | 2104.316 | 1781.072 | 336.416 |
| Bahamas | 0.223417 | 25.28493 | 25.28493 | 79.17381 | 185.0694 | 299.9504 | 410.0559 | 614.6567 | 613.198 | 672.4542 | 626.7134 | 499.6123 | 410.9139 | 295.9646 | 333.0662 | 261.606 | 234.8727 | 217.5711 |
| Bahrain | 0.104493 | 1.833828 | 1.833828 | 4.206906 | 8.313599 | 15.13561 | 33.99644 | 57.19936 | 88.98536 | 94.47374 | 125.3343 | 140.3114 | 159.6488 | 178.2005 | 172.4347 | 189.8811 | 207.4954 | 24.00944 |
| Bangladesh | 0.412397 | 25.05002 | 25.05002 | 50.50285 | 102.4573 | 150.1775 | 237.8198 | 415.1944 | 537.6959 | 460.466 | 428.8622 | 412.2846 | 275.9461 | 485.3767 | 461.5496 | 532.7343 | 497.3491 | 117.2559 |
| Barbados | 0.166679 | 12.6208 | 12.6208 | 44.09737 | 81.64388 | 150.4257 | 246.4902 | 312.7301 | 425.5513 | 500.5924 | 470.4398 | 432.8956 | 331.2659 | 270.2266 | 294.2935 | 258.138 | 217.94 | 183.537 |
| Belarus | 0.166343 | 55.66373 | 55.66373 | 185.7773 | 367.7817 | 488.3559 | 588.0555 | 707.0087 | 876.1767 | 890.2767 | 642.2855 | 368.1271 | 218.4788 | 146.9615 | 161.0252 | 133.9956 | 120.1851 | 359.4276 |
| Belgium | 0.067203 | 10.29353 | 10.29353 | 31.53715 | 74.63126 | 168.0115 | 284.5658 | 454.6201 | 578.7784 | 622.6538 | 547.6808 | 423.0184 | 335.9029 | 282.0684 | 282.2581 | 275.5853 | 284.4197 | 224.8268 |
| Belize | 0.382059 | 35.26398 | 35.26398 | 105.8804 | 198.1947 | 339.1963 | 581.6259 | 801.1483 | 1038.752 | 1003.547 | 860.7044 | 772.0345 | 588.7394 | 452.1622 | 525.7712 | 398.0782 | 324.4461 | 208.0005 |
| Benin | 0.861436 | 34.04514 | 34.04514 | 84.11477 | 159.6253 | 309.031 | 381.5626 | 566.6382 | 834.0785 | 1199.85 | 1021.241 | 825.3983 | 672.1279 | 557.8913 | 625.0988 | 464.3974 | 383.6194 | 111.6293 |
| Bermuda | 0.113887 | 5.697086 | 5.697086 | 15.51565 | 39.32414 | 86.36457 | 152.9061 | 217.7922 | 268.9026 | 289.8349 | 296.7564 | 263.8547 | 240.8187 | 194.6188 | 214.7591 | 184.7091 | 154.6971 | 131.9669 |
| Bhutan | 1.212765 | 57.85546 | 57.85546 | 121.4463 | 215.6808 | 319.1223 | 431.1812 | 561.5621 | 663.7971 | 730.1481 | 747.1339 | 748.3889 | 672.6256 | 573.0295 | 615.6695 | 546.5765 | 425.1481 | 186.3929 |
| Bolivia (Plurinational State of) | 0.329887 | 24.16152 | 24.16152 | 68.21506 | 160.6659 | 330.034 | 590.4133 | 962.1788 | 1367.22 | 1770.963 | 1886.136 | 1763.346 | 1516.856 | 1129.728 | 1232.748 | 1046.358 | 783.8234 | 304.086 |
| Bosnia and Herzegovina | 0.23841 | 13.34459 | 13.34459 | 33.23592 | 73.3955 | 146.6298 | 246.1486 | 357.0058 | 493.1721 | 593.5479 | 551.5318 | 491.6752 | 441.5432 | 262.2876 | 309.9075 | 190.49 | 137.639 | 222.8549 |
| Botswana | 0.434961 | 21.20874 | 21.20874 | 71.13083 | 146.0328 | 271.0131 | 415.5594 | 610.7137 | 764.6977 | 781.7162 | 754.3474 | 611.9729 | 481.641 | 354.89 | 390.9777 | 307.6521 | 242.88 | 150.3085 |
| Brazil | 0.311186 | 37.14216 | 37.14216 | 103.5386 | 216.2522 | 366.1868 | 526.8259 | 696.2928 | 788.2815 | 768.0068 | 680.7231 | 568.9529 | 436.0139 | 311.9121 | 344.6518 | 290.3538 | 240.9415 | 253.5276 |
| Brunei Darussalam | 0.10846 | 5.329096 | 5.329096 | 19.45514 | 49.52279 | 110.7173 | 173.0458 | 196.2071 | 235.8512 | 240.6379 | 230.9939 | 196.281 | 152.7874 | 140.874 | 138.1215 | 149.9246 | 138.8166 | 65.17113 |
| Bulgaria | 0.708831 | 50.55211 | 50.55211 | 160.0026 | 328.6655 | 534.8605 | 849.9971 | 1152.362 | 1347.449 | 1319.318 | 1078.646 | 767.9212 | 563.2818 | 285.4587 | 333.6954 | 234.5413 | 167.38 | 558.4812 |
| Burkina Faso | 0.804832 | 25.2794 | 25.2794 | 55.81161 | 102.2727 | 209.9927 | 236.7907 | 392.4334 | 552.7507 | 1034.608 | 1000.005 | 776.5697 | 725.6722 | 613.7421 | 679.11 | 527.8921 | 433.9877 | 89.49509 |
| Burundi | 0.683452 | 26.09189 | 26.09189 | 49.11068 | 116.2174 | 291.1229 | 431.6851 | 644.4113 | 838.9753 | 1191.494 | 1278.916 | 984.8951 | 819.5615 | 541.4575 | 640.8389 | 387.4175 | 286.6688 | 117.2649 |
| Cabo Verde | 0.697115 | 22.96325 | 22.96325 | 58.35111 | 114.3392 | 251.6943 | 344.3769 | 512.4918 | 762.7671 | 937.5037 | 726.2559 | 621.4829 | 479.3621 | 459.5371 | 487.7569 | 448.3664 | 371.2701 | 163.2259 |
| Cambodia | 1.575657 | 70.28077 | 70.28077 | 186.9364 | 365.1171 | 606.7271 | 837.4541 | 1071.231 | 1333.711 | 1463.586 | 1442.511 | 1309.592 | 1087.391 | 828.1649 | 904.1879 | 756.815 | 566.2022 | 334.0468 |
| Cameroon | 0.869431 | 30.27894 | 30.27894 | 74.9999 | 147.2355 | 286.7117 | 339.5854 | 500.271 | 725.8661 | 1075.504 | 916.0188 | 726.848 | 575.3874 | 475.7478 | 530.4755 | 395.2418 | 326.5192 | 108.8078 |
| Canada | 0.033021 | 5.757303 | 5.757303 | 16.63907 | 36.22272 | 72.04985 | 124.4111 | 200.7035 | 285.0931 | 324.3643 | 287.2068 | 242.7432 | 194.9825 | 152.7822 | 161.2378 | 142.2285 | 143.6755 | 114.164 |
| Central African Republic | 0.965323 | 22.58746 | 22.58746 | 45.52781 | 112.8858 | 289.6154 | 483.7649 | 718.0015 | 996.9887 | 1285.795 | 1154.446 | 827.0323 | 635.6092 | 350.6261 | 394.2569 | 272.0196 | 192.7607 | 132.767 |
| Chad | 1.128482 | 30.36998 | 30.36998 | 74.88195 | 154.7807 | 333.4969 | 429.7986 | 643.2988 | 973.5591 | 1411.885 | 1203.669 | 933.3766 | 685.2424 | 536.7828 | 607.5895 | 441.0407 | 354.9857 | 109.3234 |
| Chile | 0.110756 | 10.30801 | 10.30801 | 35.98901 | 101.9023 | 211.9212 | 357.4776 | 547.9358 | 724.422 | 817.6396 | 847.5883 | 689.9972 | 521.4732 | 308.8437 | 356.3056 | 275.0098 | 223.4775 | 243.9048 |
| China | 0.046272 | 3.871238 | 3.871238 | 13.98697 | 36.68236 | 66.19612 | 85.99884 | 114.5048 | 140.5562 | 153.2566 | 150.3705 | 146.2944 | 132.8536 | 109.2313 | 114.3235 | 111.4695 | 68.57827 | 61.69727 |
| Colombia | 0.115848 | 7.729804 | 7.729804 | 21.0457 | 37.75551 | 65.5933 | 96.16643 | 147.3753 | 216.6768 | 290.3942 | 316.3159 | 324.571 | 306.0097 | 243.3353 | 270.2461 | 227.6411 | 199.2159 | 75.63566 |
| Comoros | 0.410973 | 20.08814 | 20.08814 | 37.10001 | 83.68986 | 191.2033 | 283.4516 | 429.3414 | 568.5036 | 809.3011 | 920.3692 | 741.3749 | 676.2032 | 466.8673 | 548.6794 | 353.7326 | 266.1037 | 131.1607 |
| Congo | 0.671849 | 25.12439 | 25.12439 | 51.529 | 113.5471 | 242.0666 | 373.5181 | 523.372 | 719.0146 | 958.7089 | 971.9724 | 790.365 | 723.1331 | 477.4897 | 504.6255 | 441.6108 | 363.985 | 129.5993 |
| Cook Islands | 0.132441 | 8.555008 | 8.555008 | 14.96637 | 49.51156 | 71.93346 | 139.9419 | 172.1807 | 234.9462 | 233.3179 | 192.299 | 145.044 | 129.2285 | 92.86876 | 82.81223 | 106.5787 | 93.55226 | 76.5992 |
| Costa Rica | 0.179804 | 17.82752 | 17.82752 | 56.93285 | 123.0053 | 229.0368 | 358.2721 | 539.6145 | 726.9337 | 838.8063 | 802.2922 | 711.2381 | 620.7277 | 405.052 | 455.0865 | 391.3202 | 306.0296 | 218.9856 |
| Côte d'Ivoire | 0.914931 | 31.03948 | 31.03948 | 76.63626 | 151.9962 | 302.2087 | 368.0423 | 526.2817 | 771.0261 | 1131.101 | 989.9746 | 768.199 | 619.5205 | 527.6915 | 595.5289 | 437.0391 | 353.0853 | 119.2018 |
| Croatia | 0.105809 | 12.35914 | 12.35914 | 47.27771 | 114.9559 | 232.9398 | 398.5668 | 615.4927 | 811.7936 | 906.7522 | 844.1861 | 709.9211 | 509.7958 | 334.7935 | 392.8049 | 276.7087 | 201.6861 | 346.9829 |
| Cuba | 0.144727 | 9.217138 | 9.217138 | 29.67228 | 78.06457 | 196.2666 | 327.5243 | 473.8567 | 576.6896 | 593.1257 | 499.6261 | 434.0946 | 329.716 | 200.0085 | 237.0928 | 179.388 | 140.9693 | 224.7497 |
| Cyprus | 0.032282 | 7.271746 | 7.271746 | 21.80134 | 50.8854 | 79.48463 | 120.0534 | 194.1865 | 257.6346 | 314.9816 | 329.4606 | 320.293 | 337.4811 | 394.8831 | 386.0972 | 394.269 | 473.2009 | 113.0514 |
| Czechia | 0.169599 | 20.19151 | 20.19151 | 69.13623 | 152.4774 | 261.7207 | 447.9765 | 629.7364 | 816.0809 | 848.4963 | 695.4547 | 484.112 | 323.8783 | 180.7197 | 208.4249 | 167.4463 | 120.3211 | 309.3214 |
| Democratic People's Republic of Korea | 0.167459 | 11.91977 | 11.91977 | 35.91903 | 80.23073 | 144.3762 | 227.6412 | 315.2788 | 386.6618 | 392.1696 | 356.744 | 287.7032 | 219.2274 | 147.271 | 163.6471 | 123.0833 | 96.76866 | 129.9149 |
| Democratic Republic of the Congo | 0.781297 | 26.93418 | 26.93418 | 54.51549 | 119.3911 | 249.2161 | 367.3419 | 534.6928 | 711.6468 | 926.8711 | 867.1001 | 673.1866 | 555.0123 | 343.2287 | 382.1212 | 294.6817 | 227.3118 | 101.156 |
| Denmark | 0.041015 | 8.858563 | 8.858563 | 29.14457 | 67.66843 | 150.3121 | 282.8872 | 457.4104 | 609.6429 | 644.6427 | 474.1785 | 353.4326 | 219.0208 | 149.3892 | 160.9791 | 134.9101 | 135.6826 | 207.0678 |
| Djibouti | 0.567555 | 21.39508 | 21.39508 | 38.15405 | 86.55853 | 206.7426 | 323.2959 | 508.1936 | 689.3886 | 976.4817 | 1095.546 | 904.7532 | 765.853 | 529.4085 | 611.1221 | 391.4013 | 286.2473 | 128.6195 |
| Dominica | 0.221791 | 15.59594 | 15.59594 | 44.5234 | 115.2732 | 183.5458 | 270.8812 | 412.3658 | 528.5265 | 560.0426 | 561.3668 | 502.3676 | 395.5249 | 294.484 | 328.481 | 258.0625 | 236.7508 | 179.9095 |
| Dominican Republic | 0.673696 | 43.38344 | 43.38344 | 117.2241 | 234.9748 | 368.4741 | 568.3021 | 867.488 | 1023.268 | 1321.379 | 1200.343 | 1283.374 | 1005.555 | 1056.248 | 990.0413 | 1197.469 | 1038.543 | 290.7226 |
| Ecuador | 0.283599 | 24.75564 | 24.75564 | 68.94875 | 149.8816 | 252.0838 | 413.2744 | 623.7616 | 878.2411 | 1095.726 | 1125.598 | 1070.654 | 951.1764 | 912.6759 | 916.2333 | 900.9572 | 913.5627 | 229.7771 |
| Egypt | 0.337024 | 5.908361 | 5.908361 | 14.87688 | 30.14409 | 57.81522 | 113.9824 | 243.2563 | 396.5454 | 557.2686 | 680.9083 | 832.5862 | 872.5196 | 944.559 | 907.5316 | 990.2378 | 1046.37 | 88.74871 |
| El Salvador | 0.303188 | 53.02015 | 53.02015 | 177.7701 | 311.2049 | 438.5928 | 558.1959 | 722.9155 | 866.2953 | 940.0943 | 859.6153 | 814.0751 | 679.5108 | 507.1043 | 569.6941 | 504.2385 | 381.5249 | 252.6553 |
| Equatorial Guinea | 0.487321 | 15.75834 | 15.75834 | 30.01855 | 62.16542 | 123.4239 | 190.1352 | 267.4493 | 366.3935 | 506.9812 | 559.7284 | 460.6836 | 449.2035 | 310.8117 | 334.9943 | 286.138 | 214.6467 | 48.01511 |
| Eritrea | 0.8048 | 35.76412 | 35.76412 | 67.25727 | 164.9317 | 393.4737 | 600.812 | 848.0116 | 1097.234 | 1413.075 | 1540.463 | 1123.814 | 1018.247 | 710.9651 | 799.5002 | 545.3303 | 364.6446 | 162.8277 |
| Estonia | 0.180826 | 31.74262 | 31.74262 | 130.3604 | 252.9668 | 460.123 | 647.0134 | 804.387 | 907.4734 | 801.5496 | 587.1834 | 325.3114 | 175.8965 | 88.83989 | 99.93665 | 78.63649 | 69.98738 | 328.629 |
| Eswatini | 0.567943 | 25.85784 | 25.85784 | 76.47671 | 157.8448 | 303.5149 | 489.5855 | 696.9975 | 852.7359 | 839.324 | 797.7175 | 610.688 | 497.2192 | 378.3396 | 405.7474 | 329.3106 | 270.286 | 135.6165 |
| Ethiopia | 0.527494 | 17.38163 | 17.38163 | 34.19326 | 75.96811 | 173.9374 | 277.0036 | 446.9178 | 631.6613 | 922.065 | 1131.253 | 987.5292 | 915.8756 | 627.5092 | 720.8834 | 498.6738 | 363.1258 | 87.01323 |
| Fiji | 0.414723 | 14.43481 | 14.43481 | 22.66813 | 62.729 | 90.90157 | 144.3146 | 175.9839 | 218.6696 | 235.6716 | 216.1973 | 168.0758 | 163.6785 | 125.8091 | 123.7168 | 133.7553 | 106.2906 | 60.96273 |
| Finland | 0.039475 | 14.16226 | 14.16226 | 52.10198 | 129.6666 | 259.9248 | 488.4058 | 716.5533 | 848.9742 | 840.539 | 604.1921 | 351.3875 | 197.0756 | 99.83758 | 116.4021 | 88.88678 | 74.91536 | 288.7085 |
| France | 0.043922 | 8.047575 | 8.047575 | 26.31979 | 69.5249 | 157.0771 | 279.8946 | 413.6461 | 511.0844 | 536.5371 | 456.6273 | 344.5659 | 265.0759 | 184.8438 | 196.9629 | 172.6446 | 171.5341 | 191.2276 |
| Gabon | 0.78411 | 26.45709 | 26.45709 | 53.50434 | 118.6939 | 237.3725 | 360.5821 | 505.623 | 728.5475 | 980.6306 | 1063.771 | 830.587 | 721.1112 | 467.6789 | 499.5327 | 444.6184 | 339.5584 | 149.1556 |
| Gambia | 0.846767 | 28.82921 | 28.82921 | 71.52565 | 142.508 | 283.6455 | 359.981 | 549.8178 | 856.0741 | 1256.999 | 1072.417 | 843.9528 | 655.9174 | 543.1218 | 585.4363 | 480.6327 | 389.4314 | 117.0611 |
| Georgia | 0.625936 | 50.25604 | 50.25604 | 175.1731 | 493.4774 | 810.0888 | 1172.256 | 1327.577 | 1285.331 | 1120.973 | 1053.366 | 975.8324 | 852.32 | 476.3196 | 611.1269 | 380.5684 | 261.6886 | 552.9229 |
| Germany | 0.06279 | 14.99731 | 14.99731 | 50.03816 | 120.0608 | 241.058 | 386.7019 | 576.1602 | 695.399 | 738.773 | 638.4517 | 516.4369 | 395.5862 | 290.3288 | 313.5578 | 271.4908 | 240.253 | 298.9283 |
| Ghana | 0.94171 | 35.25499 | 35.25499 | 90.39598 | 169.0842 | 316.6907 | 375.1763 | 587.2747 | 845.2738 | 1264.99 | 1131.529 | 880.7597 | 736.2073 | 614.6474 | 684.3398 | 526.8662 | 390.4511 | 154.8144 |
| Greece | 0.05682 | 7.579376 | 7.579376 | 20.46028 | 45.88973 | 94.73827 | 173.6787 | 246.4868 | 290.4443 | 303.2507 | 296.729 | 270.967 | 247.6531 | 189.7869 | 197.3983 | 181.8768 | 177.0509 | 136.7459 |
| Greenland | 0.033573 | 11.83794 | 11.83794 | 43.15815 | 99.49198 | 187.8627 | 279.7369 | 387.1848 | 448.7371 | 396.5626 | 280.55 | 189.6336 | 116.063 | 68.01475 | 75.14642 | 58.50735 | 58.51062 | 148.2634 |
| Grenada | 0.222183 | 15.72061 | 15.72061 | 61.10064 | 149.5196 | 250.5822 | 401.5536 | 561.3005 | 718.5669 | 854.2994 | 880.122 | 722.3582 | 556.7101 | 314.7564 | 382.012 | 246.8462 | 179.853 | 235.2991 |
| Guam | 0.663754 | 19.97752 | 19.97752 | 48.95733 | 127.9169 | 143.8208 | 177.5025 | 271.7973 | 333.3975 | 298.3349 | 261.6726 | 185.2256 | 185.7817 | 163.0361 | 147.9604 | 166.6327 | 177.6337 | 100.7708 |
| Guatemala | 0.68203 | 117.4469 | 117.4469 | 327.3684 | 640.1408 | 935.4485 | 1093.29 | 1458.92 | 1570.41 | 1593.423 | 1506.808 | 1174.802 | 878.2494 | 841.2319 | 839.1982 | 854.3023 | 828.9995 | 359.2369 |
| Guinea | 1.095349 | 32.64422 | 32.64422 | 79.07143 | 153.8761 | 309.4382 | 384.845 | 556.3688 | 863.3823 | 1264.719 | 1062.006 | 850.2368 | 640.1637 | 513.9715 | 595.7784 | 428.9545 | 352.9817 | 121.3232 |
| Guinea-Bissau | 1.430619 | 45.39081 | 45.39081 | 113.6608 | 238.4481 | 516.6256 | 658.4672 | 911.0969 | 1396.782 | 1894.171 | 1566.232 | 1171.965 | 858.0882 | 675.965 | 745.743 | 577.6006 | 445.0345 | 179.5396 |
| Guyana | 0.533485 | 55.81835 | 55.81835 | 224.9832 | 474.6303 | 840.5946 | 1176.053 | 1503.088 | 1770.788 | 1903.38 | 1533.345 | 1238.109 | 889.3135 | 575.3755 | 644.7405 | 474.2291 | 467.3663 | 464.9652 |
| Haiti | 0.332451 | 23.81629 | 23.81629 | 75.724 | 185.2296 | 389.7064 | 629.2137 | 926.6816 | 1164.902 | 1306.872 | 1188.107 | 1042.984 | 791.9024 | 550.8713 | 618.4493 | 463.9298 | 358.5809 | 206.8624 |
| Honduras | 0.472399 | 30.99089 | 30.99089 | 87.93301 | 207.5603 | 409.808 | 707.753 | 1131.601 | 1599.31 | 1888.161 | 1812.774 | 1712.203 | 1601.379 | 1240.621 | 1291.276 | 1191.104 | 1046.01 | 286.9135 |
| Hungary | 0.164523 | 23.35722 | 23.35722 | 82.75574 | 183.5347 | 332.8409 | 678.4362 | 1069.211 | 1241.213 | 1329.147 | 1123.11 | 731.7619 | 449.7 | 173.5037 | 222.4277 | 134.5337 | 85.7326 | 475.4707 |
| Iceland | 0.020079 | 3.379592 | 3.379592 | 11.91186 | 30.82815 | 62.03518 | 95.93635 | 141.8611 | 183.1955 | 190.1489 | 166.89 | 131.4534 | 96.69851 | 57.33566 | 69.51284 | 50.56816 | 43.54394 | 61.97507 |
| India | 0.733919 | 43.30735 | 43.30735 | 111.105 | 207.8544 | 267.733 | 349.2534 | 409.9987 | 482.7822 | 491.2252 | 472.7149 | 423.3193 | 349.5135 | 292.509 | 316.1929 | 252.5617 | 241.4092 | 145.654 |
| Indonesia | 0.799635 | 24.26024 | 24.26024 | 61.58015 | 111.6759 | 175.5539 | 255.9055 | 359.4653 | 505.4543 | 681.4757 | 734.359 | 744.2061 | 700.8947 | 618.7125 | 640.5917 | 600.8399 | 524.5321 | 157.8415 |
| Iran (Islamic Republic of) | 0.104731 | 1.442576 | 1.442576 | 3.52599 | 6.730391 | 11.60591 | 19.50153 | 34.22555 | 54.06193 | 69.96965 | 74.11801 | 74.31673 | 76.69578 | 72.67629 | 70.89917 | 73.10928 | 78.19671 | 14.8211 |
| Iraq | 0.13 | 1.460513 | 1.460513 | 3.630147 | 7.491875 | 17.74997 | 33.02891 | 55.17636 | 86.97661 | 99.05656 | 103.2223 | 97.91593 | 83.37978 | 66.50981 | 68.95574 | 62.75304 | 63.34144 | 13.71448 |
| Ireland | 0.059555 | 8.612071 | 8.612071 | 25.0887 | 61.51733 | 115.7529 | 172.6521 | 254.9223 | 288.0868 | 280.4349 | 254.5299 | 187.2603 | 172.9647 | 126.1628 | 129.7543 | 121.5889 | 121.7577 | 103.3786 |
| Israel | 0.054416 | 6.480432 | 6.480432 | 17.43995 | 32.23427 | 58.54003 | 99.29171 | 163.4685 | 182.4302 | 216.9765 | 241.9988 | 249.2762 | 272.6995 | 246.4152 | 248.294 | 242.2219 | 238.2071 | 67.82783 |
| Italy | 0.033519 | 2.905476 | 2.905476 | 9.566689 | 24.32261 | 52.97959 | 94.75131 | 138.6007 | 172.7681 | 194.1179 | 207.1878 | 204.7283 | 185.6615 | 146.4408 | 164.5891 | 139.2474 | 110.3127 | 92.01403 |
| Jamaica | 0.155297 | 10.18185 | 10.18185 | 25.5565 | 43.40891 | 70.05707 | 109.2959 | 170.251 | 243.3215 | 262.4471 | 244.4213 | 271.6335 | 226.1096 | 155.9355 | 189.8737 | 145.8032 | 109.6967 | 69.35842 |
| Japan | 0.041211 | 1.478885 | 1.478885 | 5.377324 | 15.16153 | 36.84963 | 67.34066 | 108.9769 | 143.7371 | 157.5349 | 145.6099 | 132.9433 | 124.0651 | 114.7799 | 117.4471 | 112.5173 | 105.7773 | 71.21177 |
| Jordan | 0.084311 | 1.165508 | 1.165508 | 3.123887 | 5.923206 | 11.93497 | 21.05417 | 38.89312 | 55.74026 | 79.07272 | 101.3675 | 90.05462 | 90.97306 | 87.24447 | 86.29607 | 86.76161 | 89.9653 | 11.45971 |
| Kazakhstan | 0.606895 | 86.24044 | 86.24044 | 273.0539 | 549.5918 | 789.8719 | 922.9161 | 1136.617 | 1452.184 | 1631.192 | 1747.669 | 1531.715 | 1169.291 | 867.0794 | 961.6887 | 695.1441 | 601.8087 | 488.8777 |
| Kenya | 0.581973 | 34.36842 | 34.36842 | 69.68934 | 160.0614 | 358.1855 | 583.8075 | 905.8582 | 1247.034 | 1764.981 | 2046.605 | 1728.106 | 1618.486 | 1118.561 | 1295.168 | 895.2249 | 592.1437 | 197.5392 |
| Kiribati | 1.213797 | 45.91347 | 45.91347 | 96.20051 | 238.0209 | 309.5471 | 486.2769 | 516.546 | 652.498 | 590.8632 | 497.1713 | 383.3073 | 385.6169 | 293.64 | 302.1482 | 284.2783 | 214.4401 | 141.0448 |
| Kuwait | 0.047308 | 0.47293 | 0.47293 | 1.464232 | 3.810108 | 8.553241 | 18.46532 | 36.66134 | 53.39496 | 70.57342 | 65.01979 | 68.08825 | 59.70418 | 62.23943 | 60.25855 | 64.92177 | 59.75817 | 10.76353 |
| Kyrgyzstan | 1.074586 | 108.2352 | 108.2352 | 343.7747 | 716.3371 | 1080.679 | 1249.967 | 1474.997 | 1761.957 | 1722.806 | 1738.83 | 1311.103 | 949.5664 | 574.0045 | 621.774 | 537.7957 | 457.1306 | 466.8112 |
| Lao People's Democratic Republic | 0.361867 | 17.82952 | 17.82952 | 57.17585 | 125.202 | 232.5297 | 343.4925 | 477.4209 | 605.263 | 670.0775 | 583.8502 | 489.787 | 359.3456 | 271.0512 | 292.2265 | 249.6796 | 204.869 | 121.6523 |
| Latvia | 0.172853 | 32.20091 | 32.20091 | 135.9941 | 285.5395 | 473.2089 | 597.9972 | 707.1544 | 682.9717 | 633.3231 | 464.1178 | 314.0186 | 208.5563 | 104.2716 | 117.3597 | 94.89388 | 72.3107 | 300.5357 |
| Lebanon | 0.165863 | 1.91401 | 1.91401 | 3.999951 | 7.373594 | 12.74094 | 21.45906 | 37.55688 | 58.64588 | 77.9396 | 89.46301 | 95.5097 | 88.42181 | 88.13745 | 86.22693 | 89.34173 | 92.78758 | 18.40992 |
| Lesotho | 0.514068 | 21.06556 | 21.06556 | 60.95766 | 130.7431 | 268.9385 | 462.3342 | 702.045 | 901.4637 | 909.1949 | 814.8863 | 602.9017 | 465.324 | 330.381 | 363.0763 | 279.7686 | 229.3483 | 153.8758 |
| Liberia | 1.140952 | 37.86059 | 37.86059 | 87.11294 | 170.4684 | 332.3932 | 414.0457 | 613.5668 | 891.2793 | 1315.007 | 1146.054 | 967.6357 | 815.5457 | 727.1571 | 798.8627 | 649.8897 | 553.4363 | 142.9977 |
| Libya | 0.138457 | 1.908992 | 1.908992 | 4.999117 | 10.61933 | 18.85836 | 33.78349 | 55.6302 | 83.0559 | 109.5507 | 121.6966 | 120.2206 | 119.9998 | 97.44891 | 98.40319 | 95.22978 | 92.04293 | 21.07104 |
| Lithuania | 0.144819 | 63.67116 | 63.67116 | 241.5905 | 549.2346 | 826.2907 | 951.7625 | 1029.105 | 1064.851 | 1000.104 | 752.8702 | 377.4345 | 217.2141 | 109.9022 | 124.8535 | 96.91639 | 79.75004 | 471.3654 |
| Luxembourg | 0.07879 | 15.79261 | 15.79261 | 50.90106 | 103.731 | 179.7591 | 269.1274 | 390.0702 | 518.897 | 530.3995 | 490.5552 | 394.5436 | 316.3363 | 230.7129 | 250.78 | 214.9407 | 202.7739 | 194.1247 |
| Madagascar | 0.641703 | 28.04277 | 28.04277 | 50.59355 | 108.4266 | 250.5888 | 378.9748 | 544.4628 | 733.3073 | 1001.16 | 1131.488 | 882.9181 | 764.4594 | 536.1794 | 617.6962 | 418.9274 | 324.1971 | 113.1639 |
| Malawi | 0.970656 | 35.86451 | 35.86451 | 62.63782 | 138.5232 | 314.0884 | 475.0903 | 693.8533 | 910.4669 | 1258.026 | 1460.096 | 1167.777 | 1049.038 | 667.2518 | 803.2437 | 517.0328 | 322.3746 | 130.2938 |
| Malaysia | 0.149707 | 5.336174 | 5.336174 | 20.17311 | 49.06261 | 86.36281 | 141.2045 | 202.0451 | 297.4312 | 350.3741 | 343.1325 | 353.9356 | 269.1291 | 206.1653 | 216.352 | 191.8145 | 185.2251 | 76.13662 |
| Maldives | 0.086873 | 5.329471 | 5.329471 | 19.91504 | 43.59265 | 66.48734 | 93.23036 | 118.7848 | 159.5174 | 180.4506 | 180.2789 | 177.1522 | 183.7119 | 180.8905 | 187.7424 | 179.4982 | 152.6006 | 40.60781 |
| Mali | 0.800845 | 26.87273 | 26.87273 | 66.94661 | 134.1409 | 272.1704 | 334.3779 | 489.2367 | 724.5057 | 1056.132 | 904.0039 | 735.1039 | 555.6152 | 448.0069 | 504.2091 | 377.1716 | 310.3974 | 96.2152 |
| Malta | 0.04642 | 9.914641 | 9.914641 | 30.9455 | 69.16821 | 114.0689 | 165.5278 | 200.6023 | 230.0449 | 248.2285 | 241.9107 | 206.404 | 155.9583 | 111.268 | 128.1786 | 97.522 | 83.66732 | 110.6291 |
| Marshall Islands | 0.74365 | 37.20089 | 37.20089 | 72.86638 | 173.8499 | 245.4573 | 373.9211 | 426.7581 | 526.8624 | 491.0776 | 434.5625 | 330.6228 | 298.5219 | 241.7325 | 240.3199 | 250.4597 | 221.7569 | 121.6694 |
| Mauritania | 0.555479 | 21.16887 | 21.16887 | 53.16578 | 100.135 | 193.855 | 239.376 | 357.6246 | 543.662 | 822.3713 | 719.576 | 615.3453 | 526.9821 | 460.0915 | 500.9203 | 416.5516 | 346.8708 | 94.50199 |
| Mauritius | 0.229903 | 15.94291 | 15.94291 | 54.50458 | 118.2695 | 194.3591 | 268.6019 | 359.8632 | 351.2309 | 410.2459 | 331.2496 | 232.237 | 184.0672 | 125.5886 | 127.6035 | 123.1525 | 120.7402 | 151.6378 |
| Mexico | 0.209087 | 44.78314 | 44.78314 | 160.7124 | 373.364 | 629.2875 | 842.8477 | 1149.099 | 1414.395 | 1491.902 | 1454.569 | 1242.261 | 1056.378 | 769.9102 | 852.5887 | 711.7915 | 583.5433 | 406.8495 |
| Micronesia (Federated States of) | 0.750683 | 36.63466 | 36.63466 | 69.63619 | 148.5455 | 217.4466 | 381.9122 | 451.9969 | 568.6653 | 508.6162 | 425.7622 | 326.5389 | 296.3337 | 220.9909 | 219.9784 | 226.4741 | 202.7054 | 129.7095 |
| Monaco | 0.068769 | 16.14249 | 16.14249 | 60.39368 | 125.0109 | 216.1101 | 291.3553 | 378.4202 | 432.4075 | 431.4874 | 388.8215 | 332.8726 | 272.9449 | 211.2643 | 230.0959 | 195.9268 | 188.2942 | 218.7471 |
| Mongolia | 1.043509 | 133.2503 | 133.2503 | 341.2962 | 653.8137 | 1028.92 | 1328.61 | 1696.728 | 2178.901 | 2671.624 | 2845.25 | 2697.994 | 2632.466 | 3001.149 | 2825.051 | 3415.604 | 3146.717 | 633.3608 |
| Montenegro | 0.102339 | 10.86492 | 10.86492 | 30.78733 | 67.36648 | 105.2703 | 162.7627 | 240.1757 | 306.4092 | 324.697 | 283.0613 | 189.0369 | 141.6081 | 90.49246 | 94.20685 | 85.64216 | 75.90429 | 112.8863 |
| Morocco | 0.150417 | 1.632579 | 1.632579 | 3.828081 | 8.272717 | 16.75405 | 31.84621 | 58.48429 | 91.81932 | 120.6167 | 132.1685 | 140.5977 | 124.5484 | 124.6745 | 123.4497 | 124.052 | 122.427 | 24.86595 |
| Mozambique | 0.843372 | 23.56733 | 23.56733 | 23.77497 | 46.32377 | 107.3208 | 209.5747 | 360.7833 | 353.1122 | 381.028 | 606.9426 | 504.553 | 421.9617 | 393.7744 | 455.2199 | 296.3424 | 240.2249 | 51.35485 |
| Myanmar | 0.666505 | 59.70875 | 59.70875 | 175.6938 | 366.7599 | 627.4572 | 888.1872 | 799.758 | 896.5218 | 765.0997 | 568.5906 | 389.0618 | 229.0024 | 133.1356 | 155.5785 | 108.1933 | 88.24134 | 274.2515 |
| Namibia | 0.400365 | 16.74079 | 16.74079 | 51.1079 | 109.3524 | 201.4889 | 335.1725 | 524.5889 | 673.0866 | 698.4277 | 673.5676 | 577.6802 | 482.0724 | 356.3741 | 399.3934 | 313.5879 | 242.7152 | 115.455 |
| Nauru | 0.808716 | 40.06243 | 40.06243 | 77.98656 | 188.7366 | 270.8543 | 416.1481 | 468.131 | 561.7715 | 507.7571 | 404.9432 | 319.2194 | 311.6682 | 323.5693 | 330.8478 | 335.0995 | 234.743 | 103.7223 |
| Nepal | 1.86649 | 86.38676 | 86.38676 | 177.1015 | 348.5941 | 529.7219 | 759.6358 | 996.0589 | 1210.836 | 1352.241 | 1387.674 | 1419.105 | 1270.812 | 1062.617 | 1128.941 | 978.3583 | 876.6753 | 312.7555 |
| Netherlands | 0.037236 | 5.896957 | 5.896957 | 14.64652 | 27.04545 | 55.29231 | 99.25356 | 161.8161 | 218.3268 | 242.6074 | 224.4536 | 198.9518 | 187.2887 | 225.1388 | 191.491 | 229.754 | 293.07 | 97.2699 |
| New Zealand | 0.02214 | 1.08705 | 1.08705 | 2.959969 | 7.468585 | 17.28441 | 33.16853 | 51.70532 | 72.23184 | 81.14059 | 73.62851 | 60.10801 | 50.4063 | 49.33836 | 44.15968 | 48.86566 | 61.21134 | 27.10992 |
| Nicaragua | 0.35181 | 34.94475 | 34.94475 | 93.54823 | 204.8033 | 388.8566 | 638.5117 | 987.3199 | 1322.688 | 1460.792 | 1545.705 | 1482.534 | 1263.073 | 929.4105 | 1045.036 | 807.0826 | 635.2858 | 276.567 |
| Niger | 0.874221 | 28.83239 | 28.83239 | 72.21198 | 143.2021 | 284.5999 | 346.3239 | 519.8843 | 803.7475 | 1182.783 | 981.0501 | 799.7442 | 629.5378 | 504.2257 | 561.8964 | 412.6541 | 349.961 | 91.6717 |
| Nigeria | 0.754387 | 29.36053 | 29.36053 | 77.23729 | 154.9257 | 284.9671 | 365.8143 | 550.5737 | 821.8723 | 1232.841 | 1162.924 | 1036.856 | 897.8966 | 786.0497 | 861.6209 | 702.8927 | 589.1925 | 122.5243 |
| Niue | 0.712461 | 21.39763 | 21.39763 | 36.57761 | 97.3228 | 147.0387 | 234.7891 | 302.3707 | 412.1719 | 414.2843 | 347.8324 | 270.7258 | 260.711 | 203.5585 | 204.5509 | 206.993 | 181.3498 | 135.1731 |
| North Macedonia | 0.272454 | 15.53502 | 15.53502 | 34.06524 | 74.05625 | 138.4562 | 230.7453 | 333.9575 | 485.1911 | 531.0117 | 527.4271 | 442.4475 | 424.9993 | 265.958 | 285.3534 | 232.541 | 178.2451 | 186.5213 |
| Northern Mariana Islands | 0.687691 | 21.2635 | 21.2635 | 45.87896 | 121.6609 | 136.5917 | 323.6734 | 286.4785 | 478.1857 | 413.0628 | 408.4943 | 316.1277 | 340.6999 | 276.9208 | 253.7767 | 304.5357 | 299.4194 | 175.7119 |
| Norway | 0.040075 | 3.679066 | 3.679066 | 9.892848 | 25.91916 | 55.51155 | 89.28321 | 136.4209 | 173.1986 | 202.204 | 187.7111 | 159.352 | 131.9561 | 109.7596 | 106.2887 | 106.9705 | 114.6991 | 70.16269 |
| Oman | 0.127511 | 1.300387 | 1.300387 | 3.644921 | 9.064754 | 14.27478 | 28.8147 | 58.78407 | 102.4072 | 128.497 | 171.2333 | 171.1149 | 150.7979 | 145.2642 | 159.9249 | 116.8377 | 117.5176 | 14.15972 |
| Pakistan | 0.645864 | 23.45746 | 23.45746 | 47.8906 | 86.11354 | 125.5105 | 181.5642 | 246.6656 | 320.8185 | 379.1256 | 400.0878 | 413.9727 | 361.9075 | 322.3989 | 342.6568 | 292.7569 | 271.3386 | 61.64841 |
| Palau | 0.578756 | 27.97075 | 27.97075 | 49.92432 | 131.0952 | 182.7283 | 270.5718 | 303.5385 | 360.2241 | 344.1363 | 290.4312 | 236.8946 | 219.3666 | 186.1688 | 174.561 | 208.6866 | 177.1846 | 148.899 |
| Palestine | 0.121552 | 1.373871 | 1.373871 | 2.805331 | 6.814838 | 11.12689 | 23.58683 | 49.61843 | 88.046 | 118.2406 | 142.8047 | 157.6504 | 146.3476 | 130.0273 | 138.6101 | 119.0763 | 120.0561 | 13.40297 |
| Panama | 0.258977 | 11.81661 | 11.81661 | 33.55349 | 55.38047 | 110.1561 | 173.3529 | 290.2357 | 427.8617 | 493.7056 | 517.4129 | 501.4803 | 445.9225 | 335.5099 | 356.6999 | 326.6606 | 305.1437 | 116.2942 |
| Papua New Guinea | 0.401402 | 16.28902 | 16.28902 | 33.94991 | 84.59626 | 129.4122 | 187.0032 | 196.0159 | 231.0781 | 206.2567 | 186.1331 | 132.0178 | 99.75834 | 64.57857 | 72.80151 | 56.93069 | 38.48884 | 46.9657 |
| Paraguay | 0.357132 | 22.52015 | 22.52015 | 60.20795 | 121.4149 | 240.6865 | 363.9456 | 526.221 | 611.3062 | 671.9212 | 637.3944 | 550.6066 | 429.1693 | 320.7207 | 348.0438 | 323.3006 | 245.4201 | 149.833 |
[truncated: 13,378 more chars]
